# Microbial lung-to-blood translocation associates with systemic inflammation in severe pneumonia: evidence from paired plasma and lower respiratory tract metagenomics

**DOI:** 10.1186/s40635-026-00862-z

**Published:** 2026-02-03

**Authors:** Haopu Yang, Matthew K. Hensley, Vi D.-B. Nguyen, Nameer S. Al-Yousif, Noel Britton, Ghady Haidar, Libing Yang, Faraaz Shah, William Bain, Xiaohong Wang, Shulin Qin, Asim A. Ahmed, Tim Blauwkamp, Sivan Bercovici, Brett A. Kaufman, Kevin M. Redding, Adam Fitch, Barbara Methé, Panayiotis V. Benos, Bryan J. McVerry, Alison Morris, Georgios D. Kitsios

**Affiliations:** 1 Division of Pulmonary, Allergy, Critical Care and Sleep Medicine, University of Pittsburgh Medical Center, 3459 Fifth Avenue, Pittsburgh, PA 15213 USA; 2 Québec Heart and Lung Institute Research Centre, Québec City, QC G1V 4G5 Canada; 3 Department of Medicine, Olive View-UCLA Medical Center, Sylmar, CA 91342 USA; 4 Department of Medicine, University of Pittsburgh Medical Center Mercy, Pittsburgh, PA 15219 USA; 5 Center for Shock, Trauma and Anesthesiology Research, Department of Anesthesiology, University of Maryland School of Medicine, Baltimore, MD 21201 USA; 6 Department of Medicine, Division of Infectious Diseases, University of Pittsburgh School of Medicine, Pittsburgh, PA 15213 USA; 7 Department of Thoracic Surgery, Peking Union Medical College Hospital, Beijing, 100730 China; 8 Veterans Affairs Pittsburgh Healthcare System, Pittsburgh, PA 15240 USA; 9 Acute Lung Injury and Infection Center for Excellence, University of Pittsburgh Medical Center, Pittsburgh, PA 15213 USA; 10 Karius Inc, Redwood City, CA 94065 USA; 11 Heart, Lung, and Blood Vascular Medicine Institute, University of Pittsburgh, Pittsburgh, PA 15213 USA; 12 Center for Medicine and the Microbiome, University of Pittsburgh, Pittsburgh, PA 15213 USA; 13 Department of Epidemiology, University of Florida, Gainesville, FL 32603 USA; 14 Rutgers Robert Wood Johnson Medical School, Rutgers University, New Brunswick, NJ 07103 USA; 15 Department of Critical Care Medicine, University of Pittsburgh, Pittsburgh, PA USA

**Keywords:** Severe pneumonia, Metagenomics, Host–microbe interaction

## Abstract

**Background:**

Biological heterogeneity in host inflammatory responses to severe pneumonia predicts clinical outcomes and may influence the effectiveness of immunomodulatory therapy. The upstream drivers of this heterogeneity remain poorly defined. We hypothesized that microbial translocation from the lungs to the bloodstream, detectable via multi-compartment metagenomic analysis, contributes to divergent host responses in pneumonia.

**Methods:**

In this nested case–control study of mechanically ventilated patients with severe pneumonia, we collected paired plasma and endotracheal aspirate samples at baseline. Plasma samples underwent microbial cell-free DNA (mcfDNA) sequencing, and endotracheal aspirates were analyzed by Nanopore metagenomic sequencing. Host-response biomarkers were measured in both plasma and endotracheal aspirate samples. Microbial translocation of pulmonary origin was defined by the genus-level concordance of detectable taxa between matched endotracheal aspirate and plasma samples.

**Results:**

Among 98 patients (76 pneumonia, 22 controls), plasma mcfDNA was markedly higher in microbiologically confirmed pneumonia compared with culture-negative pneumonia (median 4015 vs. 210 molecules/μL, *p* = 0.0006). Pulmonary microbial translocation was identified in 31 (41%) pneumonia patients and correlated significantly with plasma soluble ST2 levels, independent of clinical severity. Patients classified into the prognostically adverse hyperinflammatory subphenotype exhibited greater translocating microbial DNA levels compared to hypoinflammatory patients (*p* = 0.04), further linking translocation to host-response heterogeneity.

**Conclusions:**

Microbial lung-to-blood translocation is a measurable biological process associated with systemic inflammatory heterogeneity in severe pneumonia. This pathway may represent a novel mechanistic target for precision therapeutic strategies aimed at mitigating immune dysregulation.

**Supplementary Information:**

The online version contains supplementary material available at 10.1186/s40635-026-00862-z.

## Background

Pneumonia is a major public health threat, as the leading communicable cause of death globally [1], and one of the major causes of hospitalization and mortality in the United States even prior to the COVID-19 pandemic [2, 3]. Clinical outcomes for severe pneumonia remain suboptimal despite considerable advances in the supportive care of patients admitted to the hospital with respiratory failure due to pneumonia. Currently, accurate and timely identification of the causative pathogen with microbiological cultures—the conventional gold standard—remains a formidable challenge for pneumonia, and most patients with severe pneumonia remain undiagnosed with current microbiologic methods [4–6]. As a result, clinicians must often make empiric decisions about antimicrobials, targeting plausible but undetected pathogens, a practice that contributes to antibiotic resistance, potential drug toxicity, increased cost, and secondary infections such as *Clostridioides difficile* colitis [7].

Recent advances in sequencing technologies have transformed our ability to identify pathogens directly from clinical samples, without the need for prior culture or organism isolation. Amplicon sequencing targets conserved microbial genes (e.g., the bacterial 16S rRNA gene) and typically achieves genus-level resolution. Shotgun metagenomics can sequence all DNAs present in a sample, enabling species- or strain-level identification. Nanopore sequencing further extends these capabilities by providing long-read, real-time sequencing with minimal library preparation. These culture-independent approaches may offer faster turnaround time [8], provide more sensitive diagnostic yield [9], and potentially improve decision-making by intensivists [10]. A recent clinical trial in patients with severe pneumonia found that metagenomics-based treatment decisions resulted in a shorter time to clinical improvement compared to conventional microbiological testing [11].

While pathogen identification is central to treatment decisions, the host response plays a critical role in determining the severity and trajectory of illness in pneumonia [12]. In severe cases, pneumonia can trigger immune dysregulation, leading to respiratory complications, including acute respiratory failure and acute respiratory distress syndrome (ARDS), as well as extrapulmonary organ dysfunction manifesting as multi-system organ failure [13–15]. The use of systemic immunomodulators, such as corticosteroids, has shown promise in improving outcomes in pneumonia, although the optimal agent, dosing, timing, and patient selection are unclear [16, 17]. In that context, the biological basis for the wide inter-individual variability in host response remains poorly understood, limiting the ability to personalize immunomodulatory therapies.

Recent studies [18–21] have consistently identified two biologically distinct subphenotypes among patients with acute respiratory failure, including those with pneumonia: a hyperinflammatory subphenotype characterized by elevated systemic biomarkers of inflammation and innate immunity activation, and a hypoinflammatory subphenotype with more favorable outcomes [18]. Our group has previously shown that microbial community disruption (dysbiosis) in the lower respiratory tract is associated with the hyperinflammatory subphenotype and worse clinical outcomes [22]; we and others have also demonstrated that increased levels of circulating microbial cell-free DNA (mcfDNA)—a potential pathogen-associated molecular pattern (PAMP)—are associated with the prognostically adverse hyperinflammatory subphenotype [12, 23]. However, the mechanistic drivers of these subphenotype differences remain poorly understood. It is unknown whether systemic inflammation results from the translocation of microbial fragments across the alveolar–endothelial barrier that directly trigger circulating immune cells or from local host–microbe interactions within the alveolar space and subsequent secretion of inflammatory mediators into the bloodstream.

In the Rapid Pneumonia Pathogen Identification with DNA Sequencing (RaPPID-Seq) study, we performed a multi-compartment analysis of microbial metagenomics and host inflammation profiles, in mechanically ventilated patients with and without pneumonia. Using next-generation DNA sequencing, we profiled microbial communities in paired lower respiratory tract and plasma samples. In parallel, we quantified host inflammatory responses in both compartments using a targeted panel of protein biomarkers. This nested case–control study was designed with two primary objectives: 1) to systematically screen for microbial DNA translocation (defined as the same taxa identified in the plasma and respiratory compartments), and 2) to evaluate the relationship between microbial translocation and host inflammatory responses in the setting of severe pneumonia.

## Methods

*Cohort enrollment.* From June 2018 through January 2020, we prospectively enrolled adult hospitalized patients admitted to medical or cardiac ICUs requiring invasive mechanical ventilation via endotracheal intubation at UPMC Presbyterian Hospital. These patients were enrolled into the Acute Lung Injury Registry and Biospecimen Repository (ALIR), a prospective cohort study [19, 24, 25]. Eligible patients were adults (aged 18–90 years) admitted to the ICU requiring invasive mechanical ventilation via endotracheal intubation from diverse etiologies including pneumonia, sepsis, aspiration, cardiogenic pulmonary edema, exacerbations of chronic lung diseases, and airway protection needs. We excluded patients with chronic ventilator dependence via tracheostomy. For this analysis, we selected 98 participants using a nested case–control design. Participants were retrospectively classified as patients with pneumonia (*n* = 76) or as uninfected controls without evidence of infection at any body site (*n* = 22). The study was approved by the University of Pittsburgh Institutional Review Board (protocol STUDY19050099), and written informed consent for participation in the study was obtained from all participants or their surrogates in accordance with the Declaration of Helsinki.

*Sample collection.* At study enrollment and within 72 h of intubation, we collected blood samples for plasma separation and quantification of both inflammatory biomarkers and mcfDNA metagenomic sequencing, as previously described [12]. Simultaneously, we obtained endotracheal aspirate samples for the study of lower respiratory tract host responses and microbiota [26, 27].

*Clinical classifications.* Three physicians (GDK, MH, NA-Y) independently reviewed clinical, microbiologic, and antimicrobial treatment data within ± 3 days of baseline sampling to classify patients into one of the three clinical groups, a pragmatic framework that was independent of respiratory viral panel (RVP) results:

- Microbiologically confirmed pneumonia [MCP]: positive bacterial or fungal pathogen identification from conventional microbiological testing (blood or lower respiratory tract), along with a clinical diagnosis of pneumonia based on radiographic airspace disease and compatible signs/symptoms [4].
- Clinically diagnosed pneumonia [CDP]: clinical features of pneumonia without identifiable bacterial or fungal pathogen by conventional microbiological testing. Viral pneumonia confirmed by RVP was also classified as CDP if no bacterial or fungal co-infection was detected, because such patients often receive empiric antimicrobials regardless of virus identification.
- Uninfected controls: intubated for non-infectious reasons, such as airway protection or cardiogenic pulmonary edema.

Adjudication was blinded to sequencing and biomarker data, and disagreements were resolved through consensus (GK, MH, NA-Y). Radiographic severity was scored with the Radiographic Assessment of Lung Edema (RALE) score, as previously described [28]. We followed patients prospectively for the outcomes of ventilator-free days (VFDs) at day 28 and 60-day mortality.

*Host-response assays.* We measured inflammatory and injury-related biomarkers in plasma, and when available, in endotracheal aspirate supernatants, using a 10-plex Luminex multi-analyte panel (R&D Systems, Minneapolis, MN, United States): interleukin(IL)-6, IL-8, IL-10, soluble tumor necrosis factor receptor 1 (sTNFR1), soluble growth stimulation expressed gene 2 (sST2), fractalkine, soluble receptor of advanced glycation end-products (sRAGE), angiopoietin-2, procalcitonin, and pentraxin-3 [26]. We applied a previously derived and externally validated hyperinflammatory/hypoinflammatory classifier that was originally developed in patients with acute hypoxemic respiratory failure [25], including individuals overlapping with the present cohort. We selected this model rather than ARDS-specific subphenotype classifiers because less than half of our cohort met Berlin ARDS criteria (Table 1), making a broader acute respiratory failure framework more appropriate. In addition to feasibility and prior validation, the classifier has strong construct validity [21, 29]: its component biomarkers (sTNFR1, procalcitonin, angiopoietin-2, and bicarbonate) reflect innate immune activation, host response to infection, endothelial injury, and metabolic acidosis—key biological pathways relevant in severe pneumonia.

**Table 1:** Baseline characteristics, physiologic variables, illness severity and outcomes of cohort participants, stratified by clinical diagnosis

	Microbiologically confirmed pneumonia (*n* = 30)	Clinically diagnosed pneumonia (*n* = 46)	Uninfected controls (*n* = 22)	P value for comparisons between all 3 subgroups
Demographics				
Age (median [IQR], yrs)	55.8 (50.9–61.9)	55.3 (45.3–72.9)	60.1 (41.8–68.0)	0.69
Male, N (%)	20 (66.7)	23 (51.1)	16 (76.2)	0.12
Race, N (% Caucasian)	26 (86.7)	38 (84.4)	19 (90.5)	0.73
COPD, N (%)	7 (23.3)	6 (13.3)	1 (4.8)	0.17
Immunosuppression, N (%)	11 (36.7)	9 (19.6)	3 (13.6)	0.18
Diabetes, N (%)	10 (33.3)	20 (44.4)	2 (9.5)	0.016
BMI (median [IQR], kg/m^2^)	25.7 (22.1–32.5)	29.4 (26.7–35.0)	27.5 (25.1–29.4)	0.028
HAP, N %	6 (20)	5 (11)	NA	0.32
Steroid use prior to sampling, N (%)	11 (61.1)	21 (70.0)	2 (25.0)	0.07
Physiologic variables				
Plateau pressure (cmH_2_O, median [IQR])	19.0 (14.8–24.2)	23.0 (16.0–25.2)	17.0 (13.0–19.2)	0.016
Normalized elastance (cmH₂O/(L·kg), median [IQR])	1.4 [1.1, 2.0]	2.0 [1.5, 2.6]	1.4 [1.2, 1.6]	< 0.001
PaO_2_/FiO_2_ ratio (mmHg, median [IQR])	225.0 [159.9, 277.1]	187.8 [135.6, 231.2]	283.5 [249.4, 341.5]	< 0.01
Ventilatory ratio (median [IQR])	1.5 [1.2, 1.8]	1.8 [1.6, 2.3]	1.3 [1.2, 1.6]	0.02
Illness severity				
Vasopressor-dependent shock, N (%)	17 (56.7)	29 (63.0)	11 (50.0)	0.58
SOFA score^$^ (median [IQR])	7 (4–8)	6 (5–9)	5 (4–9)	0.21
RALE score (median [IQR])	19.8 [12.9, 23.4]	22.5 [16.6, 28.5]	12.7 [10.2, 15.1]	< 0.01
ARDS, N(%)	5 (16.7)	19 (41.3)	0 (0)	< 0.001
Hyperinflammatory subphenotype, N (%)	8 (24.1)	5 (8.9)	2 (9.1)	0.189
Outcomes				
VFD (median [IQR], days)	20 (13–24)	21 (7–25)	25 (22–27)	0.001
Mortality at 60 days, N (%)	4 (13.3)	14 (30.4)	1 (4.5)	0.028

Values are shown as median (interquartile range) or number (percentage). P values for comparisons across all subgroups were obtained from Kruskal–Wallis test for continuous variables and from Fisher’s exact test for categorical variables

Abbreviations: ARDS, acute respiratory distress syndrome. BMI, body mass index. HAP: hospital acquired pneumonia, defined as intubation for the index acute respiratory failure > 48 h post-hospital admission, with the remaining cases classified as community-acquired pneumonia. RALE, radiographic assessment of lung edema. IQR, inter-quartile range. SOFA$, Sequential Organ Failure Assessment Score, excluding the neurologic component. VFD, ventilator-free days

*Microbiota profiling strategy.* To comprehensively characterize microbial communities in patients with severe pneumonia, we profiled both the lower respiratory tract (endotracheal aspirate samples) and blood (plasma samples) using complementary sequencing techniques. This dual-compartment approach allowed us to identify which microbes were present in the lungs and which had translocated into the bloodstream.

*Lower respiratory tract sequencing.* We used two complementary methods to profile bacteria in endotracheal aspirate samples: i) Nanopore metagenomic sequencing sequences all microbial DNA present in a sample without selectively amplifying specific genes. This unbiased approach identifies bacteria, fungi, and viruses and provides species-level identification (e.g., *Staphylococcus aureus* rather than just *Staphylococcus*). It also estimates the total microbial burden in a sample. ii) 16S rRNA gene sequencing specifically targets a conserved bacterial gene that serves as a "barcode" for identifying bacteria. While this method provides genus-level rather than species-level resolution, it is more sensitive for detecting bacteria in samples with low microbial abundance, making it complementary to Nanopore metagenomics. We also used qPCR of this same 16S gene to estimate total bacterial load in each sample.

*Plasma sequencing.* McfDNA sequencing (Karius Test, Karius Inc., Redwood City, CA) detects fragments of microbial DNA circulating freely in plasma. This non-invasive blood test identifies microbes at the species level and quantifies their abundance (reported as DNA molecules per microliter-μL). Detection of microbial DNA in blood may indicate translocation from infected tissue sites into the systemic circulation.

*Human DNA quantification.* We quantified human-derived DNA in both compartments as a marker of tissue damage. In plasma, we measured human cell-free DNA using both the Karius test and targeted qPCR assays. In respiratory samples, we quantified residual human DNA remaining after depletion steps during sequencing preparation.

*Pathogen classification.* We classified all detected microbes into three categories based on their likelihood of causing respiratory infection: (i) established pathogens—microbes well-documented as causes of pneumonia (e.g., *Streptococcus pneumoniae*, *S. aureus*); (ii) potential pathogens—microbes occasionally reported in pneumonia but less clearly pathogenic; and (iii) unlikely pathogens or commensals—microbes that typically colonize airways without causing disease. This classification combined automated literature review (searching PubMed for pneumonia associations) with expert adjudication by physicians specializing in critical care, pulmonary, and infectious diseases (see details in Supplemental Methods). For analysis, we focused on established pathogens by summing their abundance across respiratory or plasma samples.

*Microbial community ecology metrics.* To describe the complexity and composition of lung microbial communities, we calculated diversity metrics commonly used in ecological studies:

**Alpha diversity** measures the variety of microbes within a single sample, analogous to counting both the number of different species in an ecosystem and how evenly distributed they are. We used the Shannon index, where higher values indicate more diverse communities (many different microbes at similar abundances), while lower values indicate dominance by one or a few microbes.

**Beta diversity** measures how different the microbial communities are between samples. We used Bray–Curtis dissimilarity, which ranges from 0 (identical communities) to 1 (completely different communities). Statistical testing (permutational analysis of variance -PERMANOVA) determined whether patient groups had significantly different community compositions.

*Definition of microbial translocation:*

 Because no standard definition exists for microbial translocation from lungs to blood, we developed an operational classification using our paired metagenomics data. We reasoned that detecting the same microbe in both the respiratory tract and plasma likely indicates movement across the lung barrier, given that pneumonia pathogens typically enter via the airways rather than the bloodstream. We defined three translocation categories:

1. **Pulmonary translocation**—detection of matching microbial genera in both endotracheal aspirate (relative abundance > 0.1%) and plasma (any detectable concentration).
2. **Non-pulmonary translocation**—detection of microbes in plasma without matching genus in the respiratory sample, suggesting origin from other body sites.
3. **No translocation**—no detectable microbial DNA in plasma.

We quantified "pulmonary translocation burden" by summing the plasma concentrations of all microbes also detected in the lung. We then examined whether translocation burden correlated with plasma inflammatory biomarkers using regression models.

*Statistical analyses.* We performed comparisons between continuous variables with the Kruskal–Wallis test and categorical variables with the Fisher’s exact test. P-values from the Kruskal–Wallis tests across all biomarkers were adjusted for multiple testing using the Benjamini–Hochberg method. We considered p value less than 0.05 as statistically significant. This was an exploratory analysis designed to generate mechanistic hypotheses. No a priori effect size estimates or formal sample size calculations were performed.

To account for potential mechanistic differences related to viral infection, we performed exploratory analyses stratifying the CDP group into three subgroups based on RVP results: (1) viral CDP (positive RVP), (2) non-viral CDP (negative RVP), and (3) CDP without RVP testing. Clinical characteristics, biomarkers, and translocation patterns were compared between these subgroups.

## Results

### Cohort clinical and biological characteristics at baseline

Of the 98 patients selected, 76 were diagnosed with pneumonia (*n* = 30 MCP and *n* = 46 CDP), while 22 had no evidence of infection and served as uninfected controls. Baseline demographics were similar between groups (Table 1), whereas comorbidity profiles differed, with more prevalent diabetes and higher body mass index in the CDP group. Ten (22%) of CDP patients were diagnosed with a viral pneumonia (*n* = 5 Rhinovirus/Enterovirus, *n* = 2 Coronavirus [non-SARS-CoV-2], *n* = 2 Influenza A, and *n* = 1 RSV B), but had no evidence of bacterial or fungal superinfection by available conventional microbiological testing. Most pneumonia cases represented community-acquired pneumonia (CAP, 85%), with the remaining cases classified as hospital-acquired pneumonia (HAP) prior to intubation. CDP patients had a higher incidence of ARDS (41.3%) compared to MCP patients (16.7%, *p* < 0.01), along with worse respiratory mechanics (higher plateau and normalized elastance), worse gas exchange (hypoxemia and CO_2_ clearance), and increased 60-day mortality (30.4%) compared to MCP (13.3%) and controls (4.5%, *p* = 0.02). Radiographic severity by RALE scores was also significantly higher in CDP patients, with a median RALE of 22.5 [16.6–28.5] (median [inter-quartile range]), *p* < 0.05. Biospecimens were obtained in 1 [1–1.8] days post-ICU admission and 1 [1–2] day post-intubation with no difference in sampling timing between groups. There were no significant differences in systemic corticosteroid use prior to biospecimen sampling between MCP (61%) and CDP patients (70%; Table 1). CDP patients showed markedly higher endotracheal aspirate levels of sRAGE and sST2 compared to MCP or controls (Figure S1A-C, Table S1). Plasma biomarkers were similarly distributed between MCP and CDP groups, with both showing significantly higher levels compared to controls (Figures S1D-F, Table S1). The proportion of patients with the hyperinflammatory subphenotype did not differ significantly across the three groups (24%, 9%, and 9% classified as hyperinflammatory in MCP, CDP, and controls, respectively; Figure S1G).

### Distinct metagenomic profiles in the lung and blood compartments by clinical diagnosis

Details on microbial sequencing yields and filtering of outputs for each compartment and platform are provided in the supplement (Table S2; Figures S2-S3). 

**Fig. 1:**
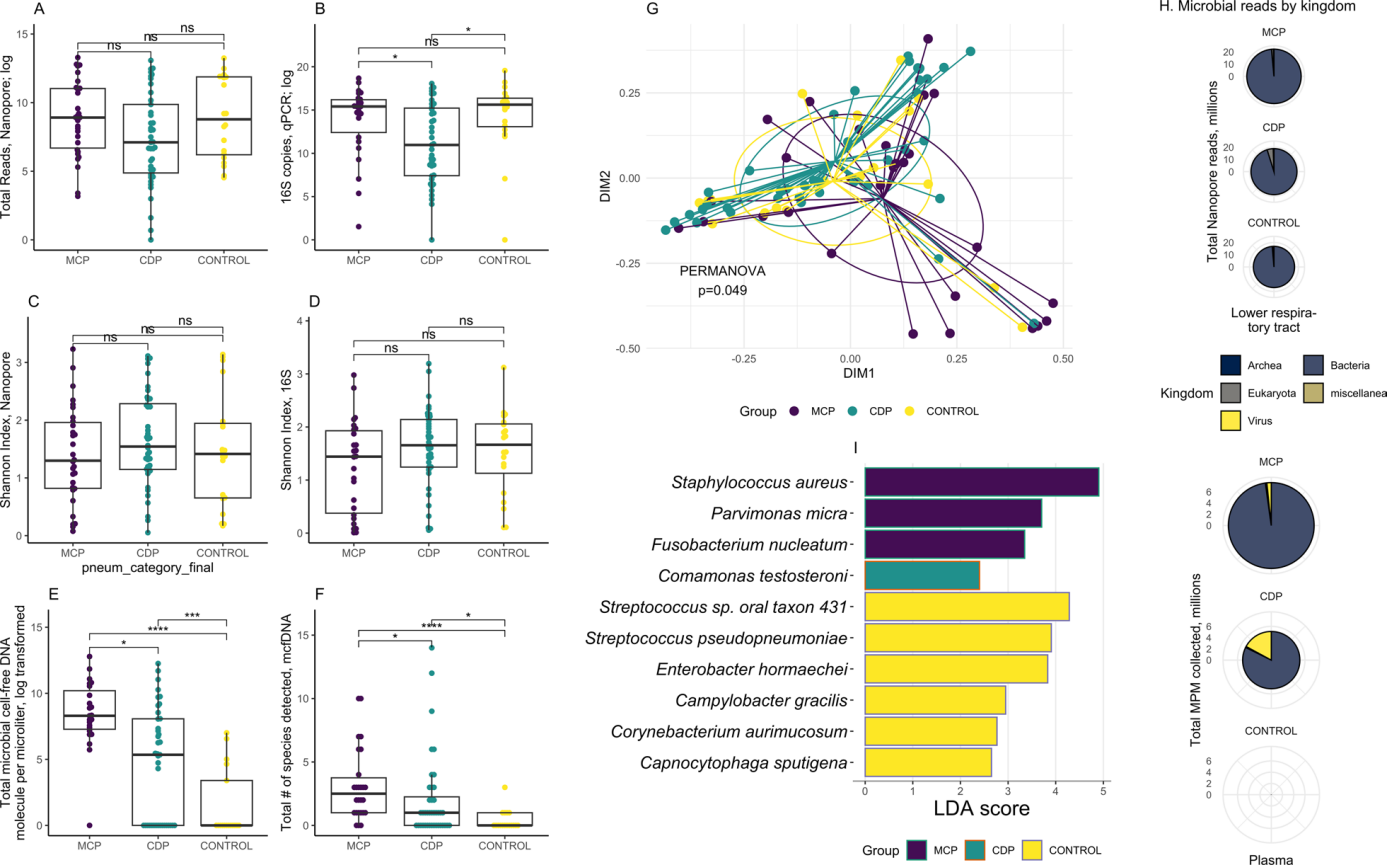
Microbial load, diversity, and composition in lower respiratory tract and plasma compartments. Lower respiratory tract microbiota were profiled by Nanopore metagenomic sequencing and 16S rRNA gene qPCR and sequencing of endotracheal aspirates, and circulating microbial DNA was assessed using Karius microbial cell-free DNA (mcfDNA) sequencing of plasma. **A**, **C** Nanopore sequencing of endotracheal aspirate samples showed similar total microbial reads across clinical groups, with microbiologically confirmed pneumonia (MCP) showing relatively reduced Shannon diversity compared to the clinically diagnosed pneumonia (CDP) subgroup (post hoc *p* = 0.047). **B, D** By 16S rRNA gene qPCR and sequencing, endotracheal aspirate samples demonstrate decreased microbial load in the CDP subgroup and similar Shannon diversity across all subgroups. *, *p* < 0.05; ns, non-significant pairwise comparisons. **E**, **F** Plasma mcfDNA profiling revealed significantly higher total microbial load as well as the number of species detected in MCP subgroups compared to CDP and to controls. **G**–**I** Microbial community composition (**G**) differed between clinical groups (Nanopore; PERMANOVA *p* = 0.049) with specific taxa enriched in each group as identified by linear discriminant analysis effect size (LEfSe; panel **I**). **H** Across clinical groups and compartments, bacterial reads constituted the majority of detected microbial signal, with fungi and viruses contributing a smaller but variable proportion. In plasma from CDP patients, the relative contribution of viral reads was higher compared to MCP, though absolute viral loads remained low. Pie chart size reflects total microbial reads within each clinical subgroup. *, *p* < 0.05; ***, *p* < 0.005; ****, *p* < 0.001; ns, non-significant

Microbial load and diversity patterns differed markedly by compartment and by clinical diagnosis. In the lower respiratory tract, total bacterial load was similar in MCP and uninfected controls (by 16S qPCR or Nanopore total reads; Fig. 1A–B), whereas Shannon diversity indices were comparable between MCP and CDP patients (by both 16S sequencing and Nanopore metagenomics; Fig. 1C–D). In contrast, plasma mcfDNA profiling revealed striking differences by clinical groups. MCP patients exhibited markedly higher plasma mcfDNA burden (4015 [1461–28436] molecules/μL) compared to CDP (210 [0–3190], p = 0.0006) and controls (0 [0–30], *p* < 0.0001; Fig. 1E). The number of detected microbial species in plasma followed the same pattern, with higher numbers detected in MCP (median 2.5 [1–4] species) relative to CDP (1 [0–2]) and controls (0 [0–1], *p* < 0.05; Fig. 1F). In terms of diagnostic yield, metagenomic results from the lower respiratory tract and plasma were largely concordant with conventional microbiological testing in MCP cases (65–67% concordance; Figure S4A), whereas in CDP cases, combining two compartment metagenomics enabled detection of additional plausible respiratory pathogens in 64% of patients (Figure S4B).

Parallel analyses of human DNA yielded a similar compartment-specific pattern. In the lung, human DNA abundance by Nanopore sequencing was comparable across diagnostic groups (*p* = 0.30). Conversely, plasma human cell-free DNA levels (hcfDNA, by the Karius Test) were substantially elevated in both MCP and CDP patients relative to controls (Figure S5A). Nuclear DNA levels (nDNA, by qPCR) showed a similar pattern, whereas mitochondrial DNA (mtDNA) did not differ significantly (Figure S5B). Importantly, plasma hcfDNA and nDNA concentrations were significantly higher in patients with elevated lactate (> 2 mmol/L), and correlated positively with markers of epithelial and endothelial injury (sRAGE and Angiopoietin-2, Figures S5C–D, S6). These findings suggest that circulating human cell-free DNA reflects diffuse cellular and endothelial injury, complementing microbial metagenomic signals.

Beyond overall microbial load and diversity, the composition of microbial communities differed markedly between compartments and clinical groups. Beta diversity of endotracheal aspirate samples showed significant differences by both Nanopore and 16S rRNA sequencing (PERMANOVA *p* = 0.049 and *p* = 0.0004, respectively, Fig. 1G and Figure S7). Tests of homogeneity of dispersion for Nanopore-derived beta-diversity indicated no significant differences in variability across groups (ANOVA *p* = 0.87), suggesting that the PERMANOVA signal reflects differences in centroids rather than dispersion. A subset of MCP samples driving separation along ordination axes by Nanopore were enriched for *S. aureus* and several lower abundance taxa, indicating biological rather than technical sources of variation. Across all groups, bacterial reads accounted for > 95% of total microbial Nanopore reads in the lower respiratory tract (Fig. 1H), followed by fungal reads (notably 4.7% in CDP patients). In plasma, MCP patients had predominantly bacterial mcfDNA (97%), whereas viral DNA constituted a greater proportion in CDP (18%) and controls (53%).

**Fig. 2:**
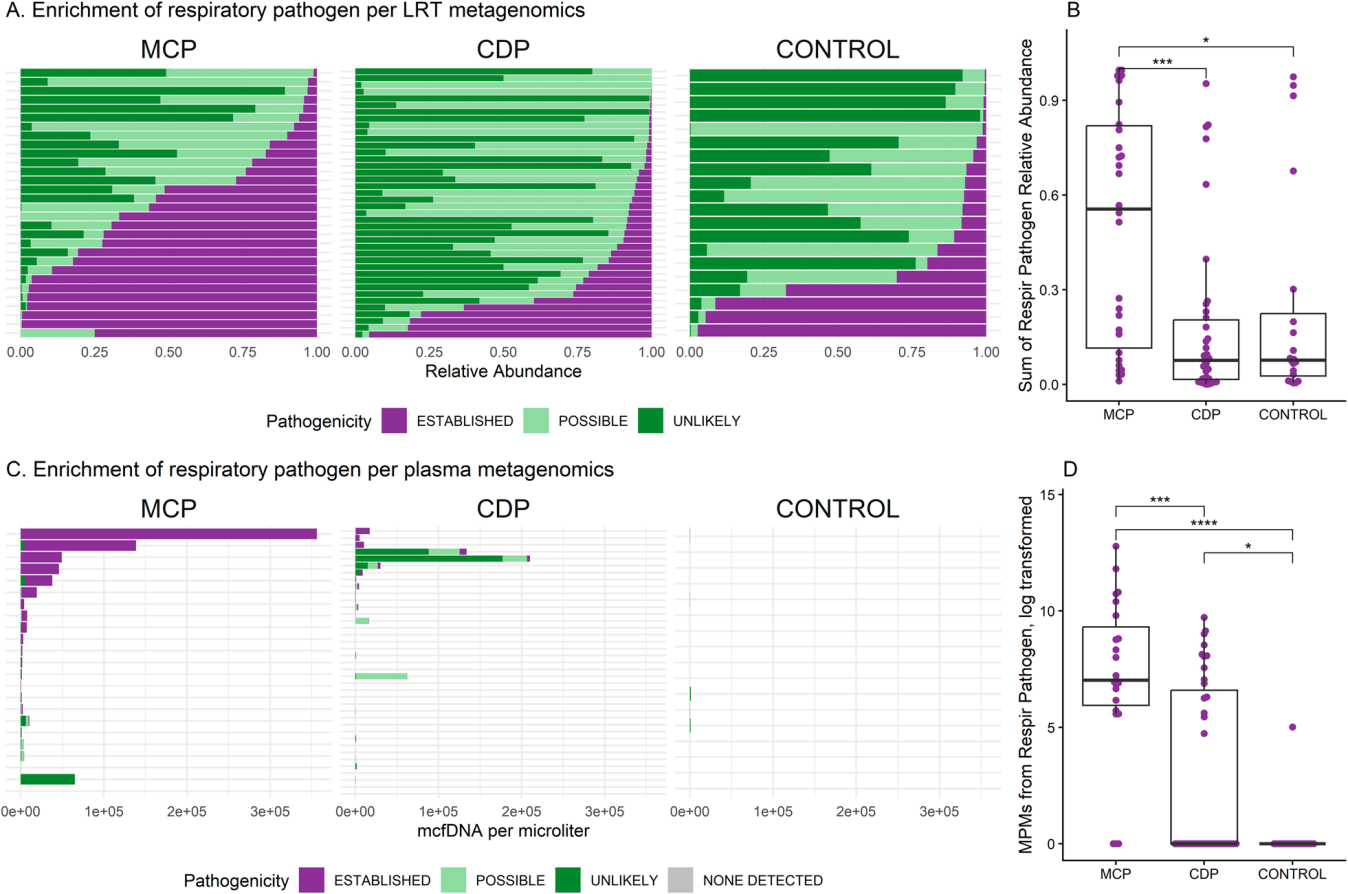
Enrichment of pathogens in the lower respiratory tract and plasma in patients with microbiologically confirmed pneumonia. **A** Bar plot breakdown within each lower respiratory tract sample showing the proportion of established pathogens, possible pathogens, and unlikely pathogenic taxa per metagenomics; the summary box plot showed significantly more (relative) abundance of pathogens identified within MCPs compared to clinically diagnosed pneumonia (CDP) and non-pneumonia controls. Detailed classification for each taxon is listed in Table S2. B. Sum of respiratory pathogen abundance in the lower respiratory tract for each patient, stratified by clinical groups. *, Benjamini–Hochberg post hoc adjusted p value from Wilcox comparison less than 0.05; **, *p* < 0.01; ***, *p* < 0.005; ****, *p* < 0.001. Non-significant comparisons are not marked. **C** Bar plot breakdown within each plasma sample showing the proportion of taxa detected by metagenomics; the summary box plot showed significantly more absolute abundance of pathogens observed in MCPs over CDPs or controls. **D** Sum of respiratory pathogen abundance in plasma in each patient, stratified by clinical groups. CDP, clinically diagnosed pneumonia. LRT, lower respiratory tract. MCP, microbiologically confirmed pneumonia. MPM, molecule(s) (of microbial cell-free DNA) per microliter. *, *p* < 0.05; ***, *p* < 0.005; ****, *p* < 0.001; ns, non-significant

Pathogen-specific analysis revealed distinct enrichment patterns between groups. Differential abundance analysis of endotracheal aspirate profiles by Nanopore metagenomics revealed enrichment of established respiratory pathogens in MCP patients (e.g., *S. aureus*; Fig. 2A); potential pathogens in CDP patients (e.g., *Campylobacter* sp., C*apnocytophaga* sp.), and mixed commensals (e.g., *Streptococcus* sp.) and potential pathogens (e.g., *Enterobacter hormaechei*) in controls (all *p* < 0.05; by linear discriminant analysis effect size [LEfSe], Fig. 1I and S7). When grouped by pathogenicity classifications, endotracheal aspirate samples from MCP patients demonstrated significantly higher abundance of respiratory pathogens compared to CDP (*p* = 0.0002, Fig. 2B). Among CDP patients, those with a viral pneumonia diagnosis had lower pathogen abundance in the lower respiratory tract compared to those without RVP testing (*p* = 0.04, Figure S8). Correspondingly, plasma mcfDNA analysis showed that MCP patients had markedly higher mcfDNA burden attributable to respiratory pathogens compared to CDP patients (*p* = 0.0003, Fig. 2C, D). Notably, among all pneumonia cases, HAP patients exhibited a significantly higher plasma pathogen mcfDNA burden compared with CAP patients (*p* = 0.045; Figure S9).

Taken together, these analyses delineate compartment-specific patterns of microbial diversity and pathogen abundance in the lower respiratory tract and plasma. We next compared sequencing results across compartments to investigate evidence of microbial translocation from lung to blood.

### Genus-level concordance across compartments indicates microbial translocation

We next focused on the 76 patients with pneumonia (MCP or CDP; controls excluded due to minimal translocation and limited relevance to the target population) with paired metagenomic data to investigate microbial translocation based on taxonomic overlap between compartments. Using our operational framework, we classified patients into three groups: 31 with pulmonary translocation (i.e., shared taxa detected in both lower respiratory tract and plasma metagenomics), 15 with non-pulmonary translocation (plasma taxa not detected in lower respiratory tract), and 30 without detectable translocation.

Translocation patterns were associated with distinct clinical features. Demographics and overall illness severity were comparable across groups (Table 2). Patients with pulmonary translocation were significantly more likely to belong to the MCP group (58.1% vs. 46.7% in the non-pulmonary translocation group and 6.7% in the no translocation group, *p* = 0.003; Figure S10). Although we initially hypothesized that pulmonary translocation would be more frequent in patients with ARDS due to presumed lung–blood barrier disruption, ARDS prevalence did not differ significantly among groups (pulmonary 16.1% vs. non-pulmonary 40.0% and no translocation 46.7%, *p* = 0.06). Similarly, we hypothesized that vasopressor-dependent shock would be more common in patients with non-pulmonary translocation due to gut ischemia and presumed impaired intestinal barrier integrity, but vasopressor use at enrollment was similar across groups (*p* = 0.87; Table 2). These findings suggest that translocation patterns are not explained solely by overt barrier disruption at either the lung or gut interface (Table 3).

**Table 2:** Baseline characteristics, physiologic variables, illness severity and outcomes of patients with pneumonia, stratified by translocation groups

	No. translocation (*n* = 15)	Non-pulmonary translocation (*n* = 15)	Pulmonary translocation (*n* = 31)	*P* value for comparisons between all 3 subgroups
Demographics				
Age (median [IQR], yrs)	49.3 [40.1, 68.1]	55.9 [42.0, 73.7]	58.5 [53.6, 64.2]	0.45
Male, N (%)	6 (40.0)	12 (80.0)	18 (58.1)	0.09
Race, N (% Caucasian)	14 (93.3)	12 (80.0)	26 (83.9)	0.77
COPD, N (%)	3 (20.0)	3 (20.0)	7 (22.6)	1.00
Immunosuppression, N (%)	1 (6.7)	6 (40.0)	9 (29.0)	0.09
Diabetes	5 (33.3)	6 (40.0)	13 (41.9)	0.94
BMI (median [IQR], kg/m^2^)	29.0 [26.8, 35.8]	25.7 [23.3, 32.2]	26.4 [22.5, 33.7]	0.20
HAP, N %	1 (7)	2 (13)	6 (19)	0.52
Steroid use prior to sampling, N (%)	6 (60.0)	8 (72.7)	12 (63.2)	0.81
Physiologic variables				
Plateau pressure (cmH_2_O, median [IQR])	18.0 [15.5, 23.0]	18.5 [15.5, 25.0]	23.0 [16.0, 27.0]	0.49
Normalized elastance (cmH₂O/(L·kg), median [IQR])	1.8 [1.3, 2.7]	1.9 [1.4, 2.7]	1.7 [1.3, 2.3]	0.69
PaO_2_/FiO_2_ ratio, mmHg (median [IQR])	209.3 [177.7, 230.8]	214.0 [117.0, 267.9]	201.7 [147.5, 277.1]	0.97
Ventilatory ratio (median [IQR])	1.8 [1.1, 2.2]	1.6 [1.6, 1.7]	1.7 [1.5, 2.3]	0.76
Illness severity				
Vasopressor-dependent shock, N (%)	8 (53.3)	9 (60.0)	19 (61.3)	0.87
SOFA score^$^ (median [IQR])	5.5 [4.7, 8.0]	6.0 [5.0, 6.5]	7.0 [4.0, 9.0]	0.73
RALE score (median [IQR])	21.5 [15.3, 27.8]	23.0 [20.2, 25.6]	20.5 [16.3, 24.0]	0.50
ARDS, N(%)	7 (46.7)	6 (40.0)	5 (16.1)	0.06
Hyperinflammatory subphenotype, N (%)	0 (0)	2 (13)	6 (19)	0.19
Outcomes				
VFD (median [IQR], days)	22.5 [17.2, 25.8]	22.0 [3.5, 25.0]	18.0 [7.5, 24.0]	0.26
Mortality at 60 days, N (%)	2 (13.3)	3 (20.0)	9 (29.0)	0.47

Values are shown as median (interquartile range) or number (percentage). P values for comparisons across all subgroups were obtained from Kruskal–Wallis test for continuous variables and from Fisher’s exact test for categorical variables

ARDS, acute respiratory distress syndrome. BMI, body mass index. HAP, hospital acquired pneumonia, defined as intubation for the index acute respiratory failure > 48 h post-hospital admission, with the remaining cases classified as community-acquired pneumonia. RALE, radiographic assessment of lung edema. IQR, inter-quartile range. SOFA$, Sequential Organ Failure Assessment Score, excluding the neurologic component. VFD, ventilator-free days

**Table 3:** Comparisons of lower respiratory tract and plasma biomarker concentrations between microbial translocation groups

	No translocation (*n* = 15)	Non-pulmonary translocation (*n* = 15)	Pulmonary translocation (*n* = 31)	Unadjusted *p*-value	Adjusted *P* value* for multiple comparisons
Lower respiratory tract, median [inter-quartile range (IQR)], pg/mL					
Ang-2	494.9 [324.6, 913.1]	758.6 [267.3, 940.8]	349.6 [116.0, 710.0]	0.3114	0.445
Fractalkine	1069.8 [84.1, 2331.2]	619.7 [24.9, 1070.5]	6.6 [3.0, 352.2]	**0.0149**	0.149
IL-6	599.3 [160.3, 1867.4]	521.5 [421.5, 4306.0]	391.7 [108.1, 4694.8]	0.8576	0.857
IL-8	27,937.0 [10732.2, 83,463.5]	83,463.5 [22228.4, 83,463.5]	83,463.5 [83462.7, 83,463.5]	0.249	0.445
IL-10	0.5 [0.4, 2.0]	0.3 [0.3, 1.2]	1.2 [0.3, 4.8]	0.3972	0.461
Pentraxin-3	31,727.8 [5673.3, 80,920.8]	121,430.7 [20809.5, 368,677.5]	108,333.4 [20444.1, 547,521.4]	0.1813	0.445
Procalcitonin	49.1 [1.8, 137.3]	37.6 [3.5, 112.6]	13.7 [1.8, 51.9]	0.2739	0.445
sRAGE	6320.3 [915.0, 20,258.8]	2150.7 [963.1, 3547.5]	963.1 [487.4, 6810.6]	0.1663	0.445
sST2	3443.6 [2250.4, 6226.7]	4956.4 [2489.2, 6234.1]	2566.6 [1621.7, 11,127.5]	0.4145	0.461
sTNFR1	1264.9 [540.9, 3114.4]	4495.6 [754.7, 7028.7]	3996.7 [849.0, 12,365.1]	0.237	0.445
Plasma, median [IQR], pg/mL					
Ang-2	4466.3 [3505.6, 6753.9]	6975.4 [3152.8, 12,661.1]	7381.5 [3890.2, 13,195.2]	0.4576	0.572
Fractalkine	410.1 [410.1, 1328.7]	1346.2 [805.1, 1974.0]	2022.3 [1277.4, 2547.3]	**0.0084**	**0.042**
IL-6	38.9 [12.9, 102.2]	37.9 [11.2, 78.7]	47.6 [14.7, 183.9]	0.5592	0.621
IL-8	9.1 [5.7, 22.0]	17.0 [7.3, 29.9]	22.5 [11.2, 37.4]	0.2055	0.343
IL-8	1.3 [1.3, 3.9]	1.3 [1.3, 8.8]	1.4 [1.3, 11.8]	0.8405	0.841
Pentraxin-3	2280.2 [1375.8, 4479.2]	8338.6 [2318.6, 16,545.4]	5571.9 [2769.6, 9889.0]	**0.0325**	0.081
Procalcitonin	339.1 [183.3, 721.8]	486.8 [240.9, 2638.2]	2333.1 [592.3, 4040.7]	**0.0142**	**0.047**
sRAGE	2196.2 [1537.2, 5470.7]	3700.9 [2405.6, 6891.9]	4356.4 [2320.8, 5819.0]	0.2963	0.423
sST2	83,477.1 [38207.1, 155,242.8]	108,502.9 [49643.6, 217,188.7]	229,141.5 [120421.4, 535,018.6]	**0.0037**	**0.037**
sTNFR1	2534.8 [1684.6, 3398.3]	3637.1 [2355.3, 5452.9]	4597.8 [2348.8, 8275.9]	0.1043	0.209

Biomarkers were measured in paired lower respiratory tract (endotracheal aspirate) and plasma specimens. Group comparisons were performed using the Kruskal–Wallis test. For LRT measurements, fractalkine and IL-10 concentrations were normalized to total protein content due to low absolute values near assay detection limits. P values were adjusted for multiple testing using the Benjamini–Hochberg method across biomarkers within each specimen type. Adjusted P values < 0.05 were considered statistically significant and are shown in bold. Ang-2, angiopoietin-2; IL, interleukin; sRAGE, soluble receptor for advanced glycation end-product; sST2, soluble growth stimulation expressed gene 2; sTNFR1, soluble tumor necrosis factor receptor-1

**Fig. 3:**
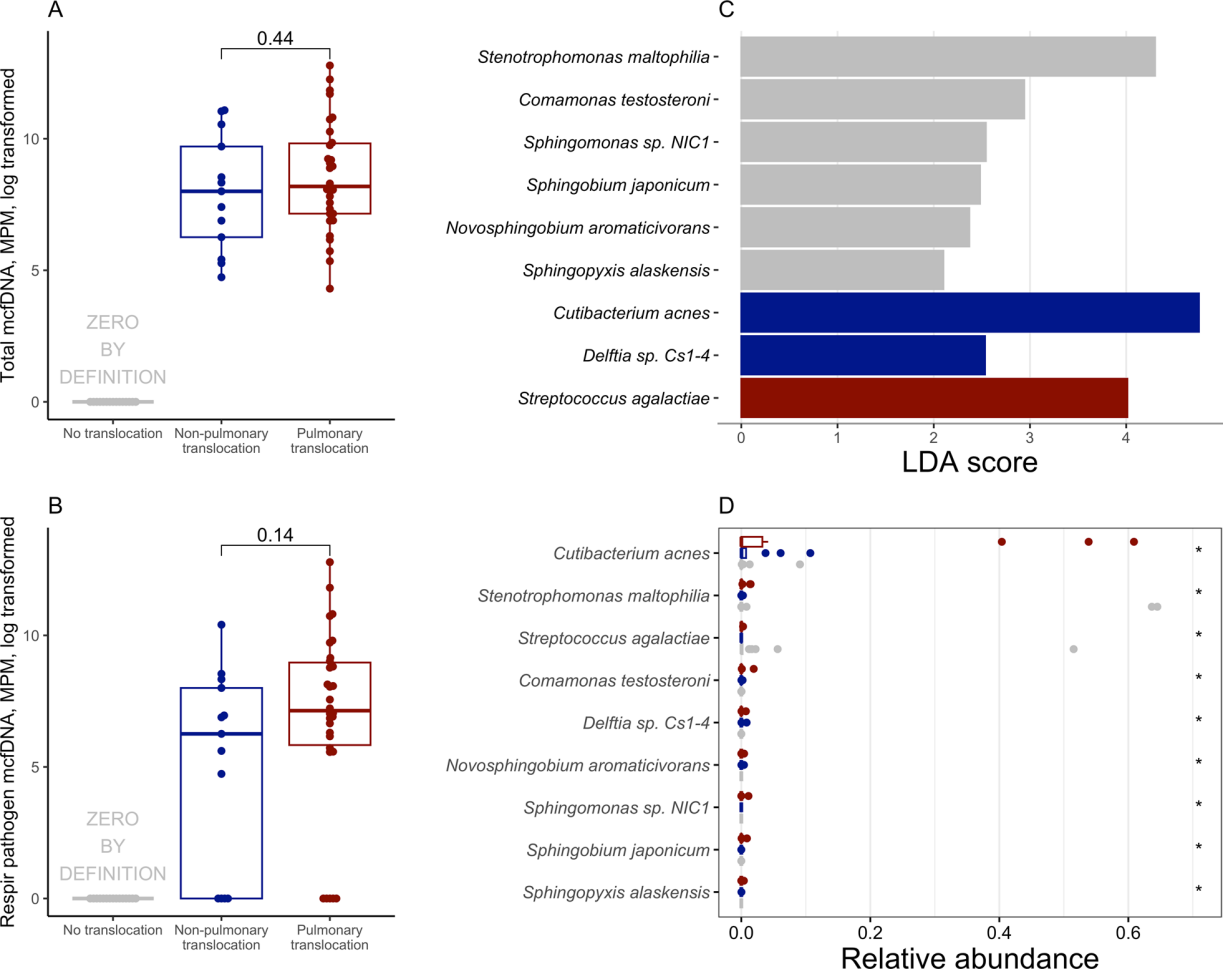
Distinct microbial signatures between translocation subgroups. Pneumonia patients were categorized into 3 subgroups (see Methods): (i) those with no translocation, (ii) those with translocation from a non-pulmonary source, and (iii) those with pulmonary translocation. **A** Comparable amounts of total microbial cell-free DNA (mcfDNA) identified in plasma between pulmonary translocation group versus non-pulmonary translocation group. **B** Non-significant enrichment of mcfDNA belonging to respiratory pathogen taxa was observed in patients with pulmonary translocation. **C** Linear discriminant analysis (LDA) of lower respiratory tract metagenomic data by Nanopore showed enrichment of *Streptococcus agalactiae* in the pulmonary translocation group. **D** Wilcox test identified top differential taxa between groups, including *Cutibacterium acnes, Stenotrophomonas maltophilia,* and *Streptococcus agalactiae*

Plasma mcfDNA burden was similar between the pulmonary (4246 [1278–18758] molecules/μL) and non-pulmonary translocation groups (2986 [522–16396] molecules/μL; *p* = 0.44, Fig. 3A). Similarly, plasma mcfDNA burden attributed to respiratory pathogens was comparable between these two groups (Fig. 3B, Figure S11). Among the 31 microbial genera classified as involved in pulmonary translocation, six (19%, *Staphylococcus*, *Streptococcus*, *Pseudomonas*, *Escherichia*, *Enterobacter*, and *Aspergillus* spp.) were identified by conventional microbiological testing and implicated as causative pathogens in at least one patient. The remaining genera were not detected by standard conventional microbiological testing and likely represent co-pathogens or commensal organisms that translocated into the bloodstream. We next investigated whether the presence and composition of these translocating microbes were associated with the magnitude of the host inflammatory response.

### Microbial translocation is associated with compartment-specific and systemic host-response heterogeneity

**Fig. 4:**
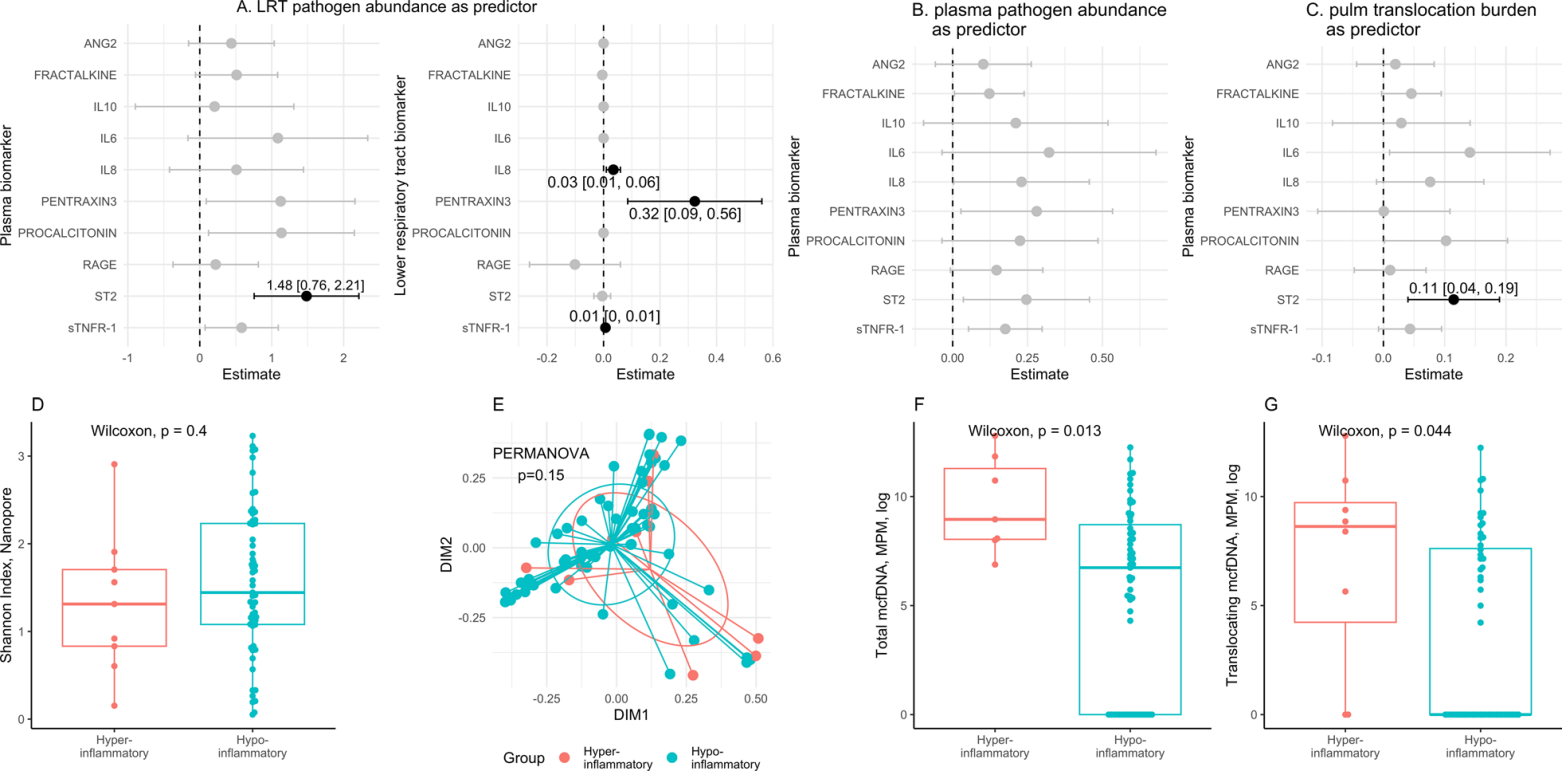
Microbial translocation is associated with host inflammatory responses. We built univariate regression models to predict ten individual biomarker levels within pneumonia patients, using the following predictors: **A** sum of respiratory pathogen abundance in lower respiratory tract (LRT) per Nanopore sequencing, **B** sum of plasma concentration of microbial cell-free DNA (mcfDNA) assigned to pathogens per Karius test, and **C** pulmonary translocation burden, defined as sum of plasma mcfDNA concentration belonging to taxa present in plasma and corresponding LRT sample from each patient. Biomarker levels were log transformed. To limit the risk of false discovery, regression results are reported as coefficient estimates, 95% confidence interval only when the Benjamini–Hochberg-adjusted *p* value was < 0.05. Further comparisons within pneumonia patients stratified into hyper-inflammatory versus hypo-inflammatory subphenotypes are reported as follows: **D** Shannon diversity in LRT per Nanopore sequencing, **E** microbial composition per principal coordinate analysis, **F** total mcfDNA in plasma, and G translocating microbial loads. Ang-2, angiopoietin-2; IL, interleukin; LRT, lower respiratory tract; sRAGE, soluble receptor for advanced glycation end-product; sST2, soluble growth stimulation expressed gene 2; sTNFR1, soluble tumor necrosis factor receptor-1

Pathogen abundance in the lower respiratory tract was associated primarily with local inflammatory responses. Elevated lower respiratory tract pathogen load correlated with increased IL-8, pentraxin-3, sTNFR-1 levels in endotracheal aspirate samples (all adjusted *p* < 0.05; Fig. 4A), reflecting a compartmentalized pulmonary host response. Among systemic biomarkers, only plasma sST2 correlated with lower respiratory tract pathogen abundance (coefficient 1.48, 95%CI: 0.76–2.21, multiple testing adjusted *p* = 0.0014).

By plasma metagenomics, pathogen mcfDNA abundance showed significant and directionally consistent associations with plasma pentraxin-3 (coefficient 0.28, 95%CI: 0.03–0.53) and sST2 (coefficient 0.25, 95%CI: 0.04–0.46; multiple-testing adjusted *p* = 0.09 for each, Fig. 4B).

Focusing on microbial DNA detected in both compartments (pulmonary translocation), significant systemic associations emerged. Plasma IL-6 (coefficient 0.14, 95% CI: 0.01–0.27), procalcitonin (coefficient 0.10, 95% CI: 0.0–0.2), and sST2 (coefficient 0.11, 95% CI: 0.04–0.19) were elevated in patients with pulmonary translocation (Fig. 4C, S12). Notably, sST2 remained significant after multiple-testing adjustment.

Finally, systemic host-response subphenotypes were independent of local microbial ecology: alpha diversity (Shannon index) and beta diversity of lower respiratory tract samples did not differ between hyperinflammatory and hypoinflammatory patients (*p* = 0.4 and *p* = 0.175, respectively; Fig. 4D, E, Figure S13). In contrast, hyperinflammatory patients had higher total plasma mcfDNA (*p* = 0.01) and, importantly, higher pulmonary translocating mcfDNA (*p* = 0.04) than hypoinflammatory patients (Fig. 4F, G). These results emphasize that systemic immune heterogeneity relates to microbial translocation rather than local pulmonary community composition.

### Sensitivity analysis: viral vs non-viral CDP comparisons

Among CDP patients, 10 had positive RVP findings (viral CDP), 20 had negative RVP results (non-viral CDP), and 16 were never tested. Patients with viral CDP demonstrated similar clinical characteristics, comparable inflammatory biomarker levels, and numerically similar proportions and burdens of microbial translocation compared to other CDP subgroups (Table S3).

## Discussion

In this nested case–control study of critically ill patients with and without pneumonia, we leveraged multi-compartment metagenomic sequencing and host biomarker profiling to investigate microbial translocation from the lungs to the bloodstream. We found that overlapping microbial taxa were frequently detected in paired lower respiratory tract and plasma specimens of patients with pneumonia, consistent with lung-to-blood translocation. Importantly, translocation was associated with elevated systemic inflammatory biomarkers, despite similar clinical severity indices across translocation-defined subgroups, highlighting its potential role in driving host-response heterogeneity.

Our analyses revealed compartment-specific microbial and host patterns. In the lower respiratory tract, total bacterial load and alpha diversity were comparable across diagnostic groups, including MCP and uninfected controls, consistent with a detectable baseline lower respiratory tract microbiome in all mechanically ventilated patients [22, 27, 30]. Broad ecological metrics such as overall load or alpha diversity were therefore insufficient to distinguish infection status. However, microbial composition differed markedly, with MCP patients exhibiting enrichment for canonical respiratory pathogens, reflecting a qualitative shift in community structure rather than changes in total abundance. This pattern suggests that the signal of pneumonia emerges from pathogen-specific enrichment within an existing microbial community, rather than from absolute increases in bacterial load detectable by metagenomics or 16S qPCR.

In contrast to the lower respiratory tract, plasma metagenomic profiles revealed striking differences by clinical diagnosis. MCP patients had markedly higher pathogen-specific mcfDNA and greater taxonomic richness than both CDP and control groups, reflecting systemic dissemination of microbial material. Notably, these patterns of lung-to-blood translocation were not explained solely by overt lung barrier injury. ARDS prevalence, radiographic severity, and the alveolar epithelial injury biomarker sRAGE were similar across translocation-defined subgroups. These findings suggest that microbial DNA can enter the circulation even in the absence of clinically apparent alveolar–capillary disruption. Instead, our findings support a model in which qualitative features of the lung microbiome, specifically the enrichment of pathogenic taxa, predispose to translocation, with plasma mcfDNA serving as a downstream signature of microbial–host interaction rather than a direct reflection of local microbial load. The strong correlation between plasma mcfDNA and inflammatory biomarkers further reinforces this concept, suggesting that circulating microbial fragments act as PAMPs amplifying inflammatory host responses in severe pneumonia.

Patients with evidence of lung-to-plasma microbial translocation had elevated levels of sST2, fractalkine, and procalcitonin, biomarkers previously associated with poor outcomes in pneumonia and acute respiratory failure [12, 26]. Among these, sST2 showed the strongest and most consistent association with microbial translocation burden after adjustment for multiple comparisons. sST2, a soluble decoy receptor for IL-33, reflects tissue-level stress and systemic inflammation and has been linked to extrapulmonary organ dysfunction in acute respiratory failure. Its relationship with circulating pathogen-derived DNA suggests that translocating microbial fragments may activate the IL-33/ST2 axis, amplifying systemic immune responses and contributing to multi-organ injury. These findings align with prior multicohort analyses showing that plasma sST2, but not lower respiratory tract sST2, correlates with extrapulmonary organ failure and predicts mortality in ARF. [23]

The detection of shared microbial DNA in the lower respiratory tract and plasma supports the concept that microbial fragments entering the bloodstream create a hyperinflammatory state that exacerbates organ dysfunction. Microbial translocation, the movement of microbes or microbial products across epithelial barriers, has long been recognized in pneumonia, particularly in cases with both respiratory and blood culture positivity. Although such cases are rare, occurring in fewer than 10% of hospitalized patients with pneumonia, dual culture positivity is associated with higher mortality, longer hospital stays, and increased healthcare costs [31]. These data collectively suggest that microbial dissemination from the lung into the bloodstream contributes to disease severity, yet culture-based detection likely underestimates its true prevalence.

By leveraging high-resolution metagenomic techniques, our study extends this concept and demonstrates that microbial DNA translocation is far more common than previously appreciated, detected in 41% of pneumonia cases even in the absence of bacteremia. Similar to prior sepsis studies identifying "bad blood" signatures [32]—where circulating microbial DNA profiles tracked with hyperinflammatory host phenotypes [23]—our data indicate that lung-to-plasma translocation in pneumonia is accompanied by systemic immune activation. We also identified a smaller subset of patients with apparent non-pulmonary translocation, in whom plasma mcfDNA did not match lower respiratory tract taxa. Because other anatomic reservoirs (e.g., gut, oropharynx, urinary tract) were not sampled, the precise source of these signatures remains uncertain. We hypothesized that gut barrier dysfunction during vasopressor-dependent shock might contribute, but vasopressor use did not differ between translocation groups, arguing against this as the sole explanation. In several non-pulmonary cases, the lower respiratory tract signal was dominated by low read counts of taxa such as *Cutibacterium acnes*, which could reflect either skin contamination or true low-biomass colonization [33, 34]. To avoid biasing analyses, we did not remove *C. acnes* a priori; however, when such low-abundance LRT signals coincided with distinct plasma microbial DNA profiles, we interpreted these as more consistent with extrapulmonary rather than lung-derived translocation.

Our study has limitations. First, although the presence of overlapping taxa in the lower respiratory tract and plasma supports the concept of microbial translocation, the sensitivity of detection can be influenced by methodological constraints. Inter-compartment comparisons were performed at the genus level due to platform-specific differences in taxonomic resolution. While strain-resolved approaches, such as whole genome sequencing of bloodstream isolates, can definitively trace translocation in bacteremic cases [35], most severe pneumonia cases occur without bacteremia, and plasma mcfDNA fragments are typically too short to permit full genome reconstruction. Therefore, future advances enabling strain-level metagenomics directly from plasma would be required to more definitively map microbial movement across compartments. Second, our operational threshold for defining translocation was necessarily permissive, as no standardized criteria currently exist for mcfDNA-based translocation detection [36]. Third, we did not evaluate pathogen-specific host responses, which may contribute to the biological heterogeneity observed in bloodstream infections and pneumonia [37]. Moreover, because our sequencing platforms detect only microbial DNA, the translocation findings in the few cases with RNA virus infection should be interpreted with caution, as we could not assess translocation of viral nucleic acids. Finally, the single-center case–control design and modest sample size—particularly within the hyperinflammatory subgroup—limit generalizability and reduce statistical power, rendering several findings hypothesis-generating rather than confirmatory. Despite these limitations, the consistent association between translocation and systemic inflammation provides a compelling signal that warrants validation in larger, prospective cohorts.

## Conclusions

Our study demonstrates that microbial DNA originating from the lung can be detected in the bloodstream of patients with severe pneumonia, supporting the concept of lung-to-blood microbial translocation. This translocation was associated with a heightened systemic inflammatory response, suggesting that microbial fragments crossing into the systemic circulation can contribute to immune dysregulation and extrapulmonary organ damage. By integrating multi-compartment metagenomics with host biomarker profiling, we provide a novel framework for understanding the biology of microbial translocation in pneumonia. Our findings position microbial translocation as both a potential marker of disease severity and a mechanistic link between local infection and systemic inflammation. Identifying patients with this signature of microbial translocation may enable more precise therapeutic approaches, ranging from targeted anti-inflammatory interventions to enhanced antimicrobial strategies for those at highest risk.

## Supplementary Information

Additional file1 (DOCX 4179 kb)

## Data Availability

To ensure transparency and reproducibility of our study, we have released our code for analyses on GitHub: https://github.com/MicrobiomeALIR/RappidSeq. Lower respiratory tract sequencing raw data are accessible in PRJNA554461 and PRJNA595346 at the Sequencing Resource Archive.
