# Supplementary material for: Microbial lung-to-blood translocation associates with systemic inflammation in severe pneumonia: evidence from paired plasma and lower respiratory tract metagenomics
Source: Intensive Care Med Exp. 2026 Feb 3;14:10. doi: 10.1186/s40635-026-00862-z (PMC12868331; doi:10.1186/s40635-026-00862-z)
Supplement: Supplementary file 1 — Additional file1 (DOCX 4179 kb) [file 40635_2026_862_MOESM1_ESM.docx]

# Supplemental Methods

## Nanopore Metagenomic Sequencing for Respiratory Samples

Following human DNA depletion in endotracheal aspirate samples, we performed metagenomic Nanopore sequencing with a rapid PCR barcoding kit (SQK-RPB004) on the MinION device (Oxford Nanopore Technologies-ONT, Oxford, UK).[[1, 2]](https://sciwheel.com/work/citation?ids=7110036,7945334&pre=&pre=&suf=&suf=&sa=0,0&dbf=0&dbf=0) We analyzed microbial metagenomic sequences with the EPI2ME platform (ONT) and the "What's In My Pot" (WIMP) workflow to quantify the abundance of microbial species. We filtered FASTQ files with a mean quality (q-score) below a minimum threshold of 7 and used the centrifuge classification engine of WIMP to assign each read to a taxonomy unit in the NCBI taxonomy database. To reduce spurious signals, we removed taxa if they (i) had relative abundance less than 0.005% across all samples or (ii) appeared in only one sample (singletons), or (iii) were never ranked among the top three most abundant taxa in any sample. The final taxa table was then generated at the species level.

## Amplicon Sequencing in Respiratory Samples (16S rRNA Gene Sequencing)

We extracted genomic DNA from endotracheal aspirates for bacterial load quantification using quantitative PCR (qPCR) of the V3-V4 region of the 16S rRNA gene.[[3–5]](https://sciwheel.com/work/citation?ids=9365225,14818913,16531108&pre=&pre=&pre=&suf=&suf=&suf=&sa=0,0,0&dbf=0&dbf=0&dbf=0) The 16S rRNA gene is a component of the bacterial ribosome that contains both highly conserved regions (allowing universal bacterial amplification) and hypervariable regions (enabling taxonomic discrimination). Amplicon sequencing targeting the V4 hypervariable region of the 16S rRNA gene (16S-Seq) was performed on the Illumina MiSeq platform. We used extensive experimental negative controls in all processing steps to rule out contamination, as well as mock microbial community positive controls (Zymo) to ensure successful target amplification.[[5]](https://sciwheel.com/work/citation?ids=16531108&pre=&suf=&sa=0&dbf=0)

Comparison of 16S and Nanopore Sequencing:

- Scope: 16S sequencing targets only bacteria, while Nanopore metagenomics detects bacteria, fungi, and viruses
- Resolution: 16S typically identifies to genus level; Nanopore can achieve species-level identification
- Sensitivity: 16S is more sensitive in low-biomass samples due to targeted amplification
- Quantification: 16S provides relative abundances; Nanopore can estimate absolute microbial load
- Interpretation: Concordance between methods strengthens confidence in bacterial findings; discordance may reflect differences in biomass, DNA quality, or presence of non-bacterial microbes.

## Plasma Metagenomic Sequencing (mcfDNA-Seq)

We conducted plasma microbial cell-free DNA (mcfDNA) metagenomic sequencing with the Karius Test (Karius Inc., Redwood City, CA).[[6]](https://sciwheel.com/work/citation?ids=6466461&pre=&suf=&sa=0&dbf=0) This commercially available clinical assay performs next-generation sequencing on plasma samples and classifies derived metagenomic sequences as human-derived cell-free DNA (hcfDNA) versus microbial-derived cell-free DNA (mcfDNA).[[7, 8]](https://sciwheel.com/work/citation?ids=15826202,12208451&pre=&pre=&suf=&suf=&sa=0,0&dbf=0&dbf=0) All microbes were reported at the species level and included a quantitative measure of abundance expressed as DNA molecules per microliter of plasma. Negative controls and decontamination procedures were performed as previously described, and taxon-specific limits of detection have been reported elsewhere.[[6]](https://sciwheel.com/work/citation?ids=6466461&pre=&suf=&sa=0&dbf=0)

## Human DNA Quantification

In plasma biospecimens, we measured: (i) human cell-free DNA (hcfDNA) concentration as reported by the Karius test, and (ii) nuclear and mitochondrial cell-free DNA (nDNA and mtDNA, respectively) using quantitative PCR assays.[[9]](https://sciwheel.com/work/citation?ids=15929469&pre=&suf=&sa=0&dbf=0) Both serve as markers of host cellular damage, with mtDNA particularly reflecting mitochondrial injury.

In lower respiratory tract biospecimens, human DNA was depleted prior to Nanopore sequencing using a saponin-based method to enrich microbial reads.[[1]](https://sciwheel.com/work/citation?ids=7110036&pre=&suf=&sa=0&dbf=0) Despite this depletion, human-derived DNA remains the predominant source in many low-biomass samples. We quantified remnant reads mapping to the human genome after depletion as an estimate of human DNA content. Although this approach is not a direct or perfect measure of epithelial injury, samples with persistently high human DNA are likely to reflect increased pleocytosis or epithelial disruption. Thus, residual human DNA serves as a measurable proxy for local tissue injury or inflammation in the respiratory tract.

## Pathogenicity Classification for Microorganisms

We aggregated all microbial taxa identified through metagenomics from both anatomical compartments and utilized the NCBI taxonomy database to create unique identifiers using taxa ID. We summarized taxa at the species level and classified them into three categories of plausible respiratory pathogenicity[[4, 10]](https://sciwheel.com/work/citation?ids=6297506,14818913&pre=&pre=&suf=&suf=&sa=0,0&dbf=0&dbf=0):

- Established pathogens – well-documented causes of respiratory infection
- Potential pathogens – organisms occasionally implicated in respiratory disease
- Unlikely pathogens or commensals – organisms that typically colonize without causing infection.

Classification Methodology:

This classification followed a two-step process:

1. Step 1: Automated Literature Review. We used the CAP-China tool, which systematically screens PubMed abstracts (indexed through March 1, 2022) for mentions of each taxon in the context of pneumonia. Taxa with >50 mentions were tentatively designated as established pathogens, those with 4-50 mentions as potential pathogens, and those with ≤3 mentions as unlikely pathogens. These thresholds were selected a priori based on prior work characterizing pneumonia-associated organisms.
2. Step 2: Expert Adjudication. We performed independent review by a panel of three physicians with expertise in critical care, pulmonary medicine, and infectious diseases (GDK, MKH, GH) to validate and refine the tentative classifications generated by automated literature review. This adjudication of microbial pathogenicity was performed agnostic of specific human cases in our cohort. Discordant classifications were resolved by consensus discussion.

For downstream analyses, we aggregated plasma mcfDNA concentration or endotracheal aspirate relative abundance values across all taxa classified as established pathogens, creating summary metrics of pathogen burden in each compartment.

## Diversity Analyses

Microbial diversity metrics were calculated to describe the ecological structure of the lower respiratory tract microbiome. These methods, derived from community ecology, provide complementary information about microbial community composition:

- Alpha Diversity (Within-Sample Diversity): We measured alpha diversity using the Shannon Index, which incorporates both richness (number of different taxa) and evenness (how uniformly abundance is distributed across taxa). Higher values indicate more diverse communities with many taxa at similar abundances; lower values indicate dominance by one or few taxa. In the clinical context of pneumonia, high diversity may reflect polymicrobial infection or colonization, while low diversity may indicate single-pathogen dominance.
- Beta Diversity (Between-Sample Compositional Differences): We assessed beta diversity using Bray-Curtis dissimilarity, which measures compositional difference between pairs of samples based on both presence/absence and relative abundance of taxa. The metric ranges from 0 (identical communities) to 1 (completely different communities).

We used permutational multivariate ANOVA (PERMANOVA) to test whether microbial community composition differed significantly between patient groups (e.g., survivors vs. non-survivors). PERMANOVA is a non-parametric test that assesses whether group centroids differ in multivariate space while accounting for within-group variability. We also used betadisper ANOVA to evaluate differences in dispersion (variability) between groups. This tests whether one group has more heterogeneous microbial communities than another, independent of differences in central tendency. All diversity analyses were performed using the vegan package in R v4.4.3.

## Definition and Quantification of Microbial Translocation

As no standardized definition of microbial translocation exists in the critical care literature, we developed an operational classification leveraging our two-compartment metagenomic profiling approach.

Biological Rationale: We reasoned that concurrent detection of the same microbial taxon in both the lower respiratory tract and plasma may indicate translocation across the alveolar-endothelial barrier in the context of pneumonia. This assumption is justified because pneumonia pathogens almost exclusively initiate infection via the airways (air-borne or bronchogenic route) rather than through hematogenous dissemination, which is a very rare mechanism in pneumonia. Therefore, finding a lung pathogen in the bloodstream likely represents barrier breach rather than primary bacteremia.

Translocation Categories: Based on this framework, we defined three mutually exclusive categories:

1. **Pulmonary Translocation** – Concurrent detection of a genus in both plasma (mcfDNA concentration >0 molecules/μL by Karius test) and endotracheal aspirate (relative abundance >0.1% by Nanopore sequencing). The 0.1% threshold in respiratory samples was chosen to exclude trace contaminants while capturing biologically relevant colonizers or pathogens.
2. **Non-pulmonary Translocation** – Detection of mcfDNA in plasma without any matching genera in endotracheal aspirate at >0.1% relative abundance. This pattern suggests microbial translocation from other anatomical sites (e.g., gut, oral cavity, skin) or potentially from lung microbes below our detection threshold.
3. **No Translocation** – No detectable mcfDNA in plasma (concentration = 0 molecules/μL), irrespective of endotracheal aspirate findings. These patients may have pneumonia confined to the respiratory compartment without systemic dissemination.

Pulmonary Translocation Burden Metric:

We quantified overall pulmonary translocation burden by summing the plasma mcfDNA concentration (molecules/μL) for all genera that were also detected in the endotracheal aspirate. This continuous metric captures both the number of translocating taxa and their abundance in the bloodstream, providing a more nuanced measure than categorical classification alone.

## Regression Analyses

We examined associations between microbial translocation and systemic inflammation using multivariable regression models. Specifically, we predicted individual plasma biomarker levels (after natural log transformation to normalize distributions) using three complementary microbial exposure metrics:

1. Lung pathogen burden: Sum of relative abundances in lower respiratory tract for all taxa classified as established respiratory pathogens (Nanopore sequencing)
2. Plasma pathogen burden: Sum of mcfDNA concentration in plasma for all taxa classified as established respiratory pathogens (Karius test)
3. Pulmonary translocation burden: Sum of plasma mcfDNA concentration for genera also detected in endotracheal aspirate (as defined above)

This analytic approach allowed us to disentangle whether systemic inflammation was driven primarily by: (a) high pathogen burden in the lung itself, (b) high pathogen burden in the blood regardless of source, or (c) specifically by lung-to-blood translocation.

## Statistical Analysis

We performed comparisons between continuous variables using the Kruskal-Wallis test (a non-parametric alternative to ANOVA appropriate for non-normally distributed data) and categorical variables using Fisher's exact test (appropriate for small sample sizes and contingency tables).

P-values from Kruskal-Wallis tests across all biomarkers were adjusted for multiple testing using the Benjamini-Hochberg false discovery rate method to control for Type I error inflation. We considered adjusted p-values less than 0.05 as statistically significant.

This was an exploratory analysis designed to generate mechanistic hypotheses about the role of microbial translocation in pneumonia pathophysiology. No a priori effect size estimates or formal sample size calculations were performed. All statistical analyses were conducted using R version 4.4.3.

# Supplementary Tables

## Table S1. Baseline inflammatory protein biomarkers stratified by clinical diagnosis in systemic circulation (plasma) and in lower respiratory tract (endotracheal aspirate).

|  | Uninfected controls (n=22) | Clinically diagnosed pneumonia (CDP; n=46) | Microbiologically confirmed pneumonia (MCP; n=30) | P value for across all 3 subgroups | P value between CDP vs MCP |
| --- | --- | --- | --- | --- | --- |
| Lower respiratory tract biomarker (median [IQR], pg/mL) | | | | | |
| Angiopoeitin-2 | 647.9 [415.8, 920.0] | 438.4 [267.3, 940.8] | 494.9 [101.8, 758.6] | 0.356 | 0.48 |
| Interleukin-6 | 552.0 [102.3, 3373.1] | 1135.2 [172.3, 4294.4] | 434.7 [116.3, 4055.9] | 0.433 | 0.55 |
| Interleukin-8 | 83463.5 [83463.5, 83463.5] | 83463.5 [19131.4, 83463.5] | 83463.5 [83463.5, 83463.5] | **0.044** | 0.07 |
| Interleukin-10 | 2.58 [2.58, 2.58] | 2.58 [2.58, 15.31] | 2.58 [2.58, 2.58] | 0.201 | 0.66 |
| Procalcitonin | 31.7 [3.5, 54.6] | 52.9 [1.8, 132.6] | 1.8 [1.8, 49.1] | **0.040** | **0.034** |
| sST-2 | 2583.6 [1780.3, 3637.4] | 4223.9 [2489.2, 8701.7] | 2455.3 [1565.2, 4495.6] | **0.006** | **0.014** |
| Fractalkine | 3424.8 [26.9, 6486.2] | 3190.8 [26.9, 7715.4] | 26.9 [26.9, 4239.0] | 0.067 | 0.08 |
| Pentraxin-3 | 116687.1 [26048.0, 451532.7] | 53089.4 [8046.1, 210553.5] | 145764.3 [26073.5, 766363.1] | 0.101 | 0.14 |
| sRAGE | 1059.7 [724.0, 2140.4] | 3547.5 [963.1, 21730.9] | 769.0 [489.0, 1156.6] | **0.001** | **0.001** |
| sTNFR-1 | 4403.2 [1505.5, 9513.3] | 2051.9 [580.9, 4495.6] | 7460.3 [908.1, 12911.0] | 0.05 | 0.09 |
| Plasma biomarker (median [IQR], pg/mL) | | | | | |
| Angiopoeitin-2 | 3924.4 [2381.0, 4675.4] | 5844.4 [3463.3, 9788.2] | 8662.1 [3867.1, 14058.9] | **0.003** | 0.38 |
| Interleukin-6 | 21.5 [10.5, 52.4] | 36.5 [12.7, 105.6] | 94.9 [32.7, 192.9] | **0.015** | 0.11 |
| Interleukin-8 | 7.86 [4.73, 11.55] | 14.51 [7.20, 23.50] | 23.72 [9.44, 43.37] | **0.003** | 0.14 |
| Interleukin-10 | 1.3 [0.9, 2.2] | 1.3 [1.3, 8.5] | 1.3 [0.8, 10.7] | 0.522 | 0.77 |
| Procalcitonin | 240.0 [89.7, 381.6] | 609.2 [194.8, 2783.0] | 1496.4 [402.2, 4040.7] | **0.002** | 0.15 |
| sST-2 | 62737.5 [29475.6, 120873.9] | 121137.3 [53231.0, 376352.8] | 177396.6 [81628.5, 483093.2] | **0.007** | 0.35 |
| Fractalkine | 787.7 [410.1, 1170.7] | 1305.2 [410.1, 2261.2] | 1591.3 [797.4, 2720.6] | **0.01** | 0.42 |
| Pentraxin-3 | 1156.5 [673.1, 7079.4] | 4367.7 [2007.6, 8338.6] | 6873.9 [2375.1, 11662.7] | **0.009** | 0.23 |
| sRAGE | 1692.0 [1325.9, 3705.9] | 3175.7 [2060.4, 5963.5] | 3744.3 [2279.0, 7060.2] | **0.004** | 0.29 |
| sTNFR-1 | 2398.7 [1113.9, 3645.6] | 3710.6 [2237.1, 6803.0] | 3864.1 [2261.6, 7504.5] | **0.023** | 0.74 |

P values for comparisons across all subgroups were obtained from Kruskal-Wallis test for continuous variables. P value between CDP vs MCP subgroup were adjusted by Benjamini-Hochberg method. IQR, inter-quartile range; sRAGE, soluble receptor for advanced glycation end-product; sST-2, soluble growth stimulation expressed gene 2; sTNFR, soluble tumor necrosis factor receptor.

## Table S2. Diagnostic validity of metagenomics sequencing versus conventional microbiologic testing.

Detailed comparison between respiratory and blood culture results, plasma mcfDNA and respiratory tract metagenomics sequencing. Samples for mcfDNA sequencing were collected within 72 hours of intubation. We recorded respiratory and/or blood cultures obtained within the time frame from 72 hours (-2 days) prior to the earliest available research plasma sample acquisition(denoted as Day 0) for mcfDNA sequencing and/or ETA metagenomic sequencing, till 72 hours (+2 days) after the final longitudinal plasma or ETA sample was obtained for metagenomics, denoted as Day -2, -1, 0, +1, +2 etc below. The columns of respiratory and/or blood cultures are marked as Non-Available (“N/A”) when no corresponding clinical microbiology sample was acquired within the time span. Given the signal-to-noise ratio of Nanopore sequencing despite our effort on trimming out singleton or rare taxa, we only listed taxa which a) rank top 3 within each sample, or b) show concordance with plasma mcfDNA result or microbiological culture results and exceed a relative abundance threshold of 0.1%. ETA samples with less than 50 reads collected from Nanopore sequencing were excluded from quantitative analyses due to low quality of sequencing results. We also recorded results of Respiratory Viral Panel (RVP) tests conducted in upper or lower respiratory tract specimens, as ordered by the clinical providers from the timing of hospital admission up to 2 days post the final research sample acquisition for each participant.

BAL, bronchoalveolar lavage; Cx, culture; ETA, Endotracheal aspirate; GNR, Gram-negative rod; GPC, Gram-positive cocci; GPCCH, Gram-positive cocci in chain; GPCCL, Gram-positive cocci in cluster; GPCP, Gram-positive cocci in pair; GPR, Gram-positive rod; GS, Gram stain; MPM, mcfDNA molecules per microliter; MRSA, methicillin resistant *Staphylococcus aureus*; MSSA, methicillin sensitive *Staphylococcus aureus*; NRF, normal respiratory flora; QP, sequencing passed qualitatively; RSV, respiratory syncytial virus; RVP, respiratory viral panel.

| Participant ID | Clinical subgroup | McfDNA Sequencing Summary (Organism, MPMs) | ETA metagenomics summary  (Organism, total reads detected by kingdom [copies], relative abundance [percentage]) | Respiratory Cultures% | Blood Cultures^$^ |
| --- | --- | --- | --- | --- | --- |
| 1 | Control | Day 0:  *Escherichia coli*, 150 | Day 1  Bacterial total: 328  *Staphylococcus aureus*, 32.01%;   *Moraxella catarrhalis*, 25.61%;   *Prevotella melaninogenica*, 7.01%  Fungal total: 837  *Malassezia globosa*, 95.22%;   *Metarhizium brunneum*, 2.27%;   *Candida albicans*, 1.08%  Day 5  Bacterial total: 1653  *Streptococcus parasanguinis*, 44.89;   *Streptococcus salivarius*, 9.98;   *Streptococcus oralis*, 8.95  Fungal total: 9  *Malassezia globosa*, 66.67;   *Candida albicans*, 11.11;   *Lobosporangium transversale*, 11.11;   *[Candida] glabrata*, 11.11 | Day 0: N/A  Day +5: N/A | Day -1: No Growth  Day +5: No Growth |
| 2 | Control | No Organism Detected, 0 | Day 1  Bacterial total: 8571  *Rothia mucilaginosa*, 89.38;   *Streptococcus salivarius*, 3.69;   *Streptococcus parasanguinis*, 2.32  Fungal total: 78  *Anthracocystis flocculosa,* 53.85;   *Candida tropicalis*, 21.79;   *Candida dubliniensis*, 20.51 | N/A | Day -1: No Growth |
| 3 | Control | No Organism Detected, 0 | Day 1  Bacterial total: 180  *Rothia mucilaginosa*, 86.67;   *Streptococcus salivarius*, 3.89;   *Streptococcus thermophilus*, 2.78  Fungal total: 3  *Anthracocystis flocculosa*, 33.33;   *Candida dubliniensis*, 33.33;   *Candida tropicalis*, 33.33 | N/A | N/A |
| 4 | Control | No Organism Detected, 0 | NA | N/A | Day -2: No Growth |
| 5 | Control | Day 0:  No Organism Detected, 0 | Day 1  Bacterial total: 431  *Streptococcus oralis*, 48.26;   *Rothia mucilaginosa*, 32.02;   *Gemella* sp. oral taxon 928, 2.78  Fungal total: 14  *Malassezia globosa*, 64.29;   *Candida albicans*, 28.57;   *Candida tropicalis*, 7.14 Day 5  Bacterial total: 1124  *Staphylococcus aureus*, 97.24;   *Streptococcus salivarius*, 1.42;   *Staphylococcus epidermidis*, 0.53  Fungal total: 1  *Malassezia globosa*, 100 | Sample Type: ETA  Day -1: (9/4/18)  GS: Many WBCs, Few GPCCH, Few GPCP  Cx: Moderate NRF | Day -1: No Growth |
| 6 | Control | *Helicobacter pylori*, 104 | Day 1  Bacterial total: 239764  *Streptococcus mitis*, 60.83;   *Streptococcus pneumoniae*, 15.7;   *Streptococcus pseudopneumoniae*, 10.43  Fungal total: 75  *Lobosporangium transversale*, 25.33;   *Malassezia globosa*, 25.33;   *Metarhizium brunneum*, 16  Viral total: 465  Streptococcus phage spp., 100 | N/A | N/A |
| 7 | Control | No Organism Detected, 0 | Day 1  Bacterial total: 375  *Corynebacterium striatum*, 64.53;   *Corynebacterium simulans*, 13.6;   *Klebsiella pneumoniae*, 5.33  Fungal total: 0  NA | Sample Type: ETA  Day -1:  GS: Many WBCs, Few GNRs, Rare GPCCL  Cx: Moderate NRF | Day -2: No Growth |
| 8 | Control | No Organism Detected, 0 | Day 1  Bacterial total: 70325  *Staphylococcus aureus*, 90.33;   *Streptococcus oralis*, 1.11;   *Lancefieldella parvula*, 0.59  Fungal total: 483  *Candida albicans*, 64.39;   *Candida dubliniensis*, 18.01;   *Candida tropicalis*, 9.32 | Sample Type: Nasopharyngeal Swab  Day -11:  RVP: negative | Day -1: No Growth |
| 9 | Control | No Organism Detected, 0 | Day 1  Bacterial total: 533723  ***Klebsiella pneumoniae***, 96.7;   *Tropheryma whipplei*, 1.94;   ***Klebsiella variicola***, 0.21  Fungal total: 39  *Metarhizium brunneum*, 58.97;   *[Candida] glabrata*, 17.95;   *Candida orthopsilosis*, 10.26 | Sample Type: ETA  Day +2:  GS: Many WBCs, Moderate GNRs, Moderate GPCPs  Cx: Heavy ***Klebsiella* sp**, Light NRF^ | Day +2: No Growth |
| 10 | Control | Day0:  No Organism Detected, 0  Day 5:  *Escherichia coli, 5650;*  *Citrobacter koseri, 3606* | Day 1  Bacterial total: 110965  *Streptococcus pseudopneumoniae*, 17.32;   *Streptococcus* sp. A12, 13.35;   *Streptococcus parasanguinis*, 12.94  Fungal total: 161  *Malassezia globosa*, 63.35;   *Lobosporangium transversale*, 14.91;   *Metarhizium brunneum*, 7.45  Viral total: 108  Streptococcus phage, 100 | Sample Type: Sputum  Day +2  GS: Many WBCs, Moderate GPCPs  Cx: Moderate NRF | Day -1: No Growth  Day +2: No Growth |
| 11 | Control | No Organism Detected, 0 | Day 1  Bacterial total: 74  *Klebsiella pneumoniae*, 41.89;   *Enterobacter cloacae*, 21.62;   *Enterobacter hormaechei*, 13.51  Fungal total: 4186  *[Candida] glabrata*, 98.54;   *Candida albicans*, 1.31 | Sample Type: ETA  Day -1:  GS: Few WBCs  Cx: Rare NRF  Sample Type: Nasopharyngeal Swab  Day 0:  RVP: positive for Rhinovirus / Enterovirus | Day -1: No Growth |
| 12 | Control | No Organism Detected, 0 | Day 1  Bacterial total: 116429  *Streptococcus pseudopneumoniae*, 13.33;   *Escherichia coli*, 12.88;   *Haemophilus parainfluenzae*, 8.87  Fungal total: 50  *Lobosporangium transversale*, 52;   *Candida albicans*, 10;   *Candida tropicalis*, 10 | N/A | N/A |
| 13 | Control | No Organism Detected, 0 | Day 1  Bacterial total: 3722  *Streptococcus oralis*, 24.31;   *Streptococcus sanguinis*, 9.89;   *Rothia dentocariosa*, 9.24  Fungal total: 5661  *Candida albicans*, 98.87;   *[Candida] glabrata*, 0.39;   *Candida tropicalis*, 0.32 | N/A | N/A |
| 14 | Control | Day 0:  *Prevotella melaninogenica*, 437,  *Veillonella dispar*, 146,  *Rothia mucilaginosa*, 123  Day 5:  *Bacteroides ovatus*, 105 | Day 1  Bacterial total: 69  *Staphylococcus aureus*, 40.58;   *Klebsiella pneumoniae*, 20.29;   *Cutibacterium acnes*, 10.14  Fungal total: 1  *Anthracocystis flocculosa*, 100 | Sample Type: ETA  Day -1:  GS: Few WBCs  Cx: No growth | Day -2: No Growth |
| 15 | Control | *Prevotella melaninogenica*, 30 | Day 1  Bacterial total: 55517  *Streptococcus mitis*, 50.98;   *Streptococcus pneumoniae*, 15.51;   *Streptococcus pseudopneumoniae*, 15.02  Fungal total: 496  *Candida albicans*, 40.73;   *Malassezia globosa*, 40.32;   *[Candida] glabrata*, 16.53  Viral total: 1046  Streptococcus phage, 100 | N/A | N/A |
| 16 | Control | Epstein-Barr virus (EBV), 1106 | Day 1  Bacterial total: 163  *Cutibacterium acnes*, 65.64;   *Actinomyces oris*, 7.36;   *Neisseria sicca*, 6.75  Fungal total: 7  *Candida dubliniensis*, 42.86;   *Lobosporangium transversale*, 28.57;   *Malassezia globosa*, 28.57 | N/A | Day -2: No Growth |
| 17 | Clinically-diagnosed pneumonia | ***Escherichia col****i*, 546 | Day 1  Bacterial total: 52  *Streptococcus parasanguinis*, 40.38;   *Streptococcus oralis*, 13.46;   *Streptococcus salivarius*, 13.46  ***Escherichia coli****,* 1.92  Fungal total: 0  NA | Sample Type: ETA  Day -2:  GS: Moderate WBCs  Cx: Rare NRF | Day 0: No Growth |
| 18 | Clinically-diagnosed pneumonia | *Enterococcus faecium*, 1154,  Torque teno virus 15, 892,  ***Candida glabrata*, 340**,  ***Streptococcus mitis*, 330,**  *Bacteroides vulgatus*, 259,  ***Streptococcus salivarius*, 185** | Day 1  Bacterial total: 4205  ***Streptococcus salivarius***, 42.47;   *Lactobacillus rhamnosus*, 20.5;   *Lactobacillus paracasei*, 12.15  Fungal total: 40769  ***Candida dubliniensis*, 44.78;   *[Candida] glabrata*, 39.05;   *Candida albicans*, 16** | Sample Type: BAL  Day -2:  GS: Many WBCs, Few yeast  Cx: **Yeast**, not cryptococcus species, NRF  Day 0:  GS: Moderate WBCs  Cx: No growth  Sample Type: Nasopharyngeal Swab  Day +2:  RVP: negative | Day -2: No Growth |
| 19 | Clinically-diagnosed pneumonia | No Organism Detected, 0 | NA | Sample Type: BAL  Day +2:  GS: Few WBCs  Cx: No growth | Day 0: No Growth |
| 20 | Clinically-diagnosed pneumonia | No Organism Detected, 0 | Day 1 LOW BACTERIA  Fungal total: 53  *Malassezia globosa*, 84.91;   *Candida albicans*, 5.66;   *[Candida] glabrata*, 5.66 | Sample Type: mini-BAL  Day -1:  GS: Many WBCs  Cx: Yeast, not cryptococcus  Day 0:  GS: Few WBCs  Cx: No growth  Sample Type: BAL  Day 0:  RVP: positive for Rhinovirus / Enterovirus  Sample Type: Nasopharyngeal Swab  Day -1:  RVP: positive for Rhinovirus / Enterovirus | Day -1: No Growth |
| 21 | Clinically-diagnosed pneumonia | Day 0 :  *Lactobacillus fermentum,* 37296,  ***Streptococcus salivarius,* 25570,**  ***Streptococcus parasanguinis*, 8492**,  *Rothia mucilaginosa,* 8429,  *Veillonella parvula,* 7695,  *Prevotella melaninogenica,* 7084,  *Haemophilus parainfluenzae,* 6695,  *Veillonella dispar,* 4772,  *Rothia dentocariosa,* 4716,  *Megasphaera micronuciformis,* 2990,  *Neisseria sicca,* 2044,  *Campylobacter concisus,* 2008,  ***Escherichia coli,* 1561**,  *Fusobacterium nucleatum,* 1315  Day 5:  ***Escherichia coli*, 863** | Day 5  Bacterial total: 52  ***Escherichia coli*, 67.31**;   ***Streptococcus agalactiae*, 9.62;   *Streptococcus mitis*, 7.69**  Fungal total: 0  NA | Sample Type: BAL  Day -1:  GS: Many WBCs  Cx: No growth  Day +2:  GS: Many WBCs  Cx: No growth  Day +4  GS: Many WBCs  Cx: No growth  Day +6:  GS: Many WBCs  Cx: No growth  Sample Type: Nasopharyngeal Swab  Day -1:  RVP: positive for RSV B virus | Day -1: No Growth  Day +3: No Growth |
| 22 | Clinically-diagnosed pneumonia | Epstein-Barr virus (EBV), 224 | Day 1 Bacterial total: 4339  Streptococcus sp. A12, 48.33;  Rothia mucilaginosa, 8.46;  Bradyrhizobium japonicum, 6.82 Fungal total: 29  Malassezia globosa, 27.59;  [Candida] glabrata, 27.59;  Candida dubliniensis, 17.24 | Sample Type: BAL  Day -2:  GS: Few WBC  Cx: NRF  Sample Type: Bronchial Wash  Day -2:  RVP: negative | N/A |
| 23 | Clinically-diagnosed pneumonia | Day 0:  No Organism Detected, 0  Day 5:  No Organism Detected, 0 | Day 1  Bacterial total: 284  *Streptococcus salivarius*, 45.07;   *Lactobacillus rhamnosus*, 17.61;   *Lactobacillus paracasei*, 11.62  Fungal total: 3057  *Candida dubliniensis*, 45.11;   *[Candida] glabrata*, 38.4;   *Candida albicans*, 16.45 | Sample Type: ETA  Day 0:  GS: Many WBCs  Cx: Rare NRF  Day 5: N/A  Sample Type: Nasopharyngeal Swab  Day 0:  RVP: negative | Day 0: No Growth  Day 5: N/A |
| 24 | Clinically-diagnosed pneumonia | ***Prevotella melaninogenica****,* 1287,  *Veillonella dispar,* 151,  ***Campylobacter concisus****,* 89 | Day 1  Bacterial total: 591  *Rothia mucilaginosa*, 69.2;   *Staphylococcus aureus*, 8.63;   *Lancefieldella parvula*, 7.11  ***Campylobacter concisus,*** 1.35  ***Prevotella melaninogenica***, 0.67    Fungal total: 1141  *Candida albicans*, 99.3;   *Candida tropicalis*, 0.26 | Sample Type: BAL  Day -1:  GS: Few WBCs  Cx: No growth  RVP: negative  Sample Type: ETA  Day -1:  GS: Rare WBCs  Cx: No growth  Sample Type: Nasopharyngeal Swab  Day -1:  RVP: negative | Day -1: No Growth |
| 25 | Clinically-diagnosed pneumonia | *Morococcus cerebrosus,* 142,  ***Veillonella parvula****,* 68 | Day 1  Bacterial total: 66925  ***Veillonella parvula*, 21.92;**   *Streptococcus oralis*, 13.35;   *Streptococcus intermedius*, 9.59  Fungal total: 468  *Malassezia globosa*, 48.93;   *Candida dubliniensis*, 30.77;   *Candida albicans*, 6.62 | Sample Type: BAL  Day 0:  GS: Many WBCs  Cx: No growth  RVP: negative  Sample Type: ETA  Day 0:  GS: Many WBCs  Cx: Rare NRF  Sample Type: Nasopharyngeal Swab  Day 0:  RVP: negative | Day -1: No Growth  Day 0: No Growth |
| 26 | Clinically-diagnosed pneumonia | *Streptococcus pneumoniae,* 8537,  *Klebsiella pneumoniae,* 8179,  Herpes simplex virus type 1 (HSV-1), 486 | NA | Sample Type: ETA  Day -1:  GS: Few WBCs  Cx: Rare NRF  Day +2:  GS: Few WBCs  Cx: Rare NRF | Day +2: No Growth |
| 27 | Clinically-diagnosed pneumonia | Day 0 :  *Pseudomonas aeruginosa,* 1920,  ***Candida dubliniensis,* 1062**,  ***Rothia mucilaginosa,* 195,**  *Torque teno virus,* 42  Day 5 :  *Escherichia coli*, 483;  *Enterococcus faecium*, 121 | Day 1  Bacterial total: 3741  ***Rothia mucilaginosa*, 70.89;**   *Streptococcus salivarius*, 10.69;   *Campylobacter concisus*, 4.95  Fungal total: 437  ***Candida dubliniensis***, 96.11;   *Malassezia globosa*, 2.75;   ***Candida albicans***, 0.69  Viral total: 559*  Human polyomavirus 3, 84 | Sample Type: BAL  Day 0:  GS: Many WBCs  Cx: No growth  RVP: Coronavirus (non-SARS-CoV-2) | N/A |
| 28 | Clinically-diagnosed pneumonia | *Helicobacter pylori,* 5451,  ***Streptococcus pneumoniae,* 2957**,  *Citrobacter koseri,* 169,  ***Haemophilus haemolyticus,* 169** | Day 1  Bacterial total: 236095  ***Streptococcus pneumoniae*, 72.29**;   ***Haemophilus influenzae*, 9.8;**  ***Streptococcus mitis*, 5.07**  Fungal total: 287  *[Candida] glabrata*, 58.89;   *Malassezia globosa*, 14.29;   *Lobosporangium transversale*, 9.06  Viral total: 46  Streptococcus phage spp., 100 | Sample Type: ETA  Day 0:  GS: Few WBCs, Moderate GPCR, Moderate GPCCH,  Rare GNR  Cx: Moderate NRF | Day -1: No Growth |
| 29 | Clinically-diagnosed pneumonia | ***Candida dubliniensis*, 74** | Day 1 LOW BACTERIA  Fungal total: 1191  ***Candida dubliniensis***, 79.51;   ***[Candida] glabrata***, 13.01;   ***Candida albicans***, 6.63 | Sample Type: BAL  Day -1:  GS: Few WBCs  Cx: No Growth  RVP: negative  Day +2:  GS: Few WBCs  Cx: No Growth  RVP: negative | Day 0: No Growth |
| 30 | Clinically-diagnosed pneumonia | Day 0:  *Escherichia coli*, 978  Day 5:  No Organism Detected, 0 | Day 1  LOW BACTERIAL READS  Fungal total: 78  *Candida albicans*, 57.69;   *[Candida] glabrata*, 33.33;   *Malassezia globosa*, 5.13 | Sample Type: ETA  Day -1:  GS: Many WBCs  Cx: Light NRF  Sample Type: Nasopharyngeal Swab  Day -1:  RVP: positive for Influenza A virus, subtype 2009H1N1 | Day 0: No Growth |
| 31 | Clinically-diagnosed pneumonia | No Organism Detected, 0 | Day 1  Bacterial total: 252290  *Rothia mucilaginosa*, 49.51;   *Streptococcus mitis*, 26.69;   *Streptococcus pneumoniae*, 6.47  Fungal total: 180  *Malassezia globosa*, 46.67;   *Anthracocystis flocculosa*, 21.67;   *Lobosporangium transversale*, 13.89  Viral total : 160  Streptococcus phage spp., 100 | Sample Type: Nasopharyngeal Swab  Day -1:  RVP: positive for Influenza A virus, subtype 2009H1N1 | Day -1: No Growth |
| 32 | Clinically-diagnosed pneumonia | Day 0:  Human herpesvirus 6A, 844  Day 5:  Human herpesvirus 6A, 385  Day 10:  Cytomegalovirus (CMV), QP;  Human herpesvirus 6A, QP | Day 5  Bacterial total: 50  *Streptococcus pneumoniae*, 40;   *Staphylococcus aureus*, 28;   *Rothia mucilaginosa*, 10  Fungal total: 1690  *Candida albicans*, 97.57;   *Malassezia globosa*, 1.6 | Sample Type: BAL  Day -1:  GS: Few WBCs  Cx: NRF  Sample Type: Sputum  Day 9 (4/26):  GS: Few WBCs  Cx: Moderate NRF  Sample Type: Nasopharyngeal Swab  Day -1:  RVP: positive for Coronavirus (non-SARS-CoV-2) | Day 0: No Growth  Day +12: No Growth |
| 33 | Clinically-diagnosed pneumonia | Day 0:  No Organism Detected, 0  Day5:  Cytomegalovirus (CMV), 195  Day 10:  Herpes simplex virus type 1 (HSV-1), QP;  Cytomegalovirus (CMV), QP | Day 1  Bacterial total: 91440  *Sphingomonas melonis*, 23.9;   *Sphingomonas koreensis*, 18.08;   *Sphingomonas hengshuiensis*, 10.96  Fungal total: 3273  *Sordaria macrospora*, 89.18;   *Candida albicans*, 9.5;   *Anthracocystis flocculosa*, 0.55  Day 5  Bacterial total: 3348  *Sphingomonas melonis*, 24.22;   *Sphingomonas koreensis*, 18.97;   *Sphingomonas hengshuiensis*, 10.57  Fungal total: 109  *Sordaria macrospora*, 95.41;   *Candida albicans*, 4.59  Day 10  Bacterial total: 66624  *Sphingomonas melonis*, 21.77;   *Sphingomonas koreensis*, 16.93;   *Sphingomonas hengshuiensis*, 12.15  Fungal total: 5549  *Candida albicans*, 61.6;   *Sordaria macrospora*, 37.14;   *Anthracocystis flocculosa*, 0.27  Viral total: 6650  Human alphaherpesvirus, 100 | Sample Type: ETA  Day 0:  GS: Few WBCs  Cx: Rare NRF  Sample Type: BAL  Day +1:  GS: Few WBCs  Cx: No growth  RVP: positive for Rhinovirus / Enterovirus | Day +6: No Growth  Day +7: No Growth  Day +12: No Growth |
|  |  |  |  |  |  |
| 35^#^ | Clinically-diagnosed pneumonia | Kaposi sarcoma-associated herpesvirus, 16123,  Cytomegalovirus (CMV), 273 | Day 1  Bacterial total: 133541  *Rothia mucilaginosa*, 91.26;   *Streptococcus oralis*, 1.73;   *Streptococcus parasanguinis*, 1.42  Fungal total: 136  *Malassezia globosa*, 76.47;   *Metarhizium brunneum*, 8.09;   *Lobosporangium transversale*, 6.62 | Sample Type: ETA  Day -1:  GS: Few WBCs, Rare GPC, Rare GPR, Rare GNR  Cx: Heavy NRF  Sample Type: BAL  Day 0:  RVP: positive for Rhinovirus / Enterovirus  CMV detection: negative  Sample Type: Nasopharyngeal Swab  Day -1:  RVP: positive for Rhinovirus / Enterovirus | Day -1: No Growth  Day 0: No Growth |
| 36 | Clinically-diagnosed pneumonia | No Organism Detected, 0 | Day 1  Bacterial total: 121  *Streptococcus oralis*, 23.14;   *Streptococcus parasanguinis*, 12.4;   *Streptococcus anginosus*, 8.26  Fungal total: 3  *Candida albicans*, 66.67;   *Malassezia globosa*, 33.33 | Sample Type: BAL  Day -2:  Cx: No growth | N/A |
| 37 | Clinically-diagnosed pneumonia | No Organism Detected, 0 | Day 1  Bacterial total: 21893  *Streptococcus parasanguinis*, 42.45;   *Rothia mucilaginosa*, 18.76;   *Streptococcus salivarius*, 11.43  Fungal total: 21  *Metarhizium brunneum*, 28.57;   *Malassezia globosa*, 28.57;   *Candida orthopsilosis*, 19.05 | Sample Type: ETA  Day -1:  GS: Moderate WBCs, Few GPCP  Cx: Rare NRF  Sample Type: BAL  Day 0:  GS: Moderate WBCs  Cx: No growth  RVP: negative  Sample Type: Nasopharyngeal Swab  Day -1:  RVP: negative | Day -1: No Growth |
| 38 | Clinically-diagnosed pneumonia | Cytomegalovirus (CMV), 5113 | Day 1  Bacterial total: 577  *Rothia mucilaginosa*, 27.04;   *Klebsiella pneumoniae*, 16.12;   *Streptococcus parasanguinis*, 15.25  Fungal total: 123  *[Candida] glabrata*, 89.43;   *Candida tropicalis*, 4.07;   *Candida dubliniensis*, 3.25 | Sample Type: ETA  Day 0:  GS: Few WBCs, Rare GNR  Cx: Light NRF  Sample Type: Nasopharyngeal Swab  Day 0:  RVP: negative | Day 0: No Growth |
| 39 | Clinically-diagnosed pneumonia | *Bacteroides vulgatus*, 194 | Day 1  Bacterial total: 211  *Rothia dentocariosa*, 24.64;   *Streptococcus parasanguinis*, 16.11;   *Rothia mucilaginosa*, 13.27  Fungal total: 4728  *Candida albicans*, 99.2;   *Candida dubliniensis*, 0.38;   *Candida tropicalis*, 0.27 | Sample Type: BAL  Day -2:  GS: Few WBCs  Cx: No growth  RVP: negative  Sample Type: Nasopharyngeal Swab  Day -2:  RVP: negative | N/A |
| 40 | Clinically-diagnosed pneumonia | BK polyomavirus, 522 | Day 1  Bacterial total: 597  *Lactobacillus rhamnosus*, 54.44;   *Lactiplantibacillus plantarum*, 18.59;   *Lactobacillus paracasei*, 16.75  Fungal total: 23  *[Candida] glabrata*, 82.61;   *Malassezia globosa*, 8.7 | Sample Type: BAL  Day -3:  RVP: negative  Day 0:  GS: Few WBCs  Cx: No growth  Sample Type: Nasopharyngeal Swab  Day -7:  RVP: positive for Rhinovirus / Enterovirus | N/A |
| 41 | Clinically-diagnosed pneumonia | *Escherichia coli*, 114 | Day 1  LOW BACTERIA  Fungal total: 719  *Malassezia globosa*, 99.44;   *Anthracocystis flocculosa*, 0.28 | Sample Type: BAL  Day -1:  GS: Many WBCs, Moderate GPC  Cx: NRF | Day -1: No Growth |
| 42 | Clinically-diagnosed pneumonia | Day 0:  No Organism Detected, 0  Day 5:  *Candida tropicalis*, 849;  Epstein-Barr virus (EBV), 48 | Day 1  Bacterial total: 51  *Cutibacterium acnes*, 39.22;   *Corynebacterium kroppenstedtii*, 21.57;   *Escherichia coli*, 11.76  Fungal total: 1  *Malassezia globosa*, 100  Day 5  Bacterial total: 9811  *Veillonella parvula*, 42.46;   *Staphylococcus haemolyticus*, 34.32;   *Lactobacillus rhamnosus*, 12.03  Fungal total: 649  *Malassezia globosa*, 61.79;   ***[Candida] glabrata***, 37.75;   *Lobosporangium transversale*, 0.31 | Sample Type: BAL  Day -2:  GS: Few WBCs  Cx: No growth  RVP: negative | Day -2: No Growth |
| 43 | Clinically-diagnosed pneumonia | No Organism Detected, 0 | Day 1  Bacterial total: 150  *Cutibacterium acnes*, 31.33;   *Lactobacillus paracasei*, 30;   *Lactobacillus casei*, 7.33  Fungal total: 1015  *[Candida] glabrata*, 79.51;   *Candida albicans*, 19.61;   *Malassezia globosa*, 0.49 | Sample Type: ETA  Day -1:  GS: Many WBCs, Moderate GPCP, Few yeast  Cx: Moderate NRF  Sample Type: BAL  Day 0:  GS: Few WBCs  Cx: No growth  Sample Type: Nasopharyngeal Swab  Day -2:  Rapid influenza / RSV PCR: negative | Day 0: No Growth |
| 44 | Clinically-diagnosed pneumonia | No Organism Detected, 0 | NA | Sample Type: ETA  Day -1:  GS: Rare WBCs  Cx: No growth  Sample Type: BAL  Day 0:  GS: Rare WBCs  Cx: No growth | Day 0: No Growth |
| 45* | Clinically-diagnosed pneumonia | ***Lactobacillus gasseri***, 102949,  ***Lactobacillus fermentum***, 55713,  ***Streptococcus mitis*, 8674,**  ***Streptococcus tigurinus*, 7663,**  ***Streptococcus dentisani*, 7435,**  ***Streptococcus oralis*, 7093,**  ***Streptococcus salivarius*, 4709,**  ***Streptococcus parasanguinis*, 4592,**  ***Streptococcus anginosus*, 4362**,  *Escherichia coli*, 3412,  ***Streptococcus vestibularis*, 3207,**  *Saccharomyces cerevisiae*, 96 | Day 1  Bacterial total: 648  ***Streptococcus salivarius*, 14.81;   *Streptococcus parasanguinis*, 14.66;   *Streptococcus anginosus*, 11.42**  Fungal total: 2  *Malassezia globosa*, 100 | Sample Type: ETA  Day -1:  GS: Many GPCP  Cx: Heavy NRF  Sample Type: Nasopharyngeal Swab  Day 0:  RVP: negative | Day -1:  GS: GPR  Cx: ***Lactobacillus gasseri*** (considered contaminant by clinical team) (1/4 bottles) |
| 46 | Clinically-diagnosed pneumonia | No Organism Detected, 0 | Day 1  Bacterial total: 96488  *Rothia mucilaginosa*, 60.86;   *Streptococcus oralis*, 5.53;   *Rothia dentocariosa*, 3.77  Fungal total: 42  *Lobosporangium transversale*, 61.9;   *Candida dubliniensis*, 9.52 | Sample Type: ETA  Day -1:  GS: Many WBCs, Many GPC, Many GNR, Many GPR  Cx: Moderate NRF  Sample Type: Nasopharyngeal Swab  Day -2:  RVP: negative | Day -2: No Growth |
| 47 | Clinically-diagnosed pneumonia | Herpes simplex virus type 2 (HSV-2), 61551,  Adeno-associated dependoparvovirus A, 977 | Day 1  Bacterial total: 236  *Prevotella melaninogenica*, 60.59;   *Cutibacterium acnes*, 11.02  Fungal total: 3  *Lobosporangium transversale*, 33.33;   *Malassezia globosa*, 33.33;   *Candida dubliniensis*, 33.33 | Sample Type: ETA  Day -1:  Cx: No growth  Day 0:  GS: Many WBCs  Cx: NRF  Sample Type: BAL  Day 0:  GS: Moderate WBCs, Yeast  Cx: No growth  RVP: negative  Sample Type: Nasopharyngeal Swab  Day -1:  RVP: negative | Day -2: No Growth  Day -1: No Growth |
| 48 | Clinically-diagnosed pneumonia | *Pseudomonas aeruginosa*, 233 | NA | Sample Type: ETA  Day 0:  GS: Many WBCs  Cx: Light NRF  Sample Type: BAL  Day 0:  GS: Few WBCs  Cx: No growth | Day -1: No Growth |
| 49 | Clinically-diagnosed pneumonia | No Organism Detected, 0 | Day 1  Bacterial total: 646  *Moraxella osloensis*, 45.98;   *Rothia mucilaginosa*, 40.09;   *Cutibacterium acnes*, 8.36  Fungal total: 2145  *Candida dubliniensis*, 99.49;   *Anthracocystis flocculosa*, 0.28;   *Malassezia globosa*, 0.19 | N/A | Day -1: No Growth |
| 50 | Clinically-diagnosed pneumonia | No Organism Detected, 0 | Day 1  Bacterial total: 159656  *Streptococcus mitis*, 17.15;   *Streptococcus salivarius*, 15.69;   *Streptococcus pseudopneumoniae*, 14.95  Fungal total: 2110  *Malassezia globosa*, 71.33;   *[Candida] glabrata*, 20.28;   *Candida tropicalis*, 6.16  Viral total : 149  Streptococcus phage spp., 100 | Sample Type: BAL  Day 0:  GS: Moderate WBCs, Many GPCs  Cx: NRF | Day 0: No Growth |
| 51 | Clinically-diagnosed pneumonia | *Streptococcus salivarius*, 448,  *Streptococcus parasanguinis*, 253,  *Bacteroides distasonis*, 169,  *Corynebacterium striatum*, 151,  *Bacteroides thetaiotaomicron*, 134,  *Bacteroides merdae*, 125 | Day 1  Bacterial total: 18041  *Haemophilus influenzae*, 79.77;   *Rothia mucilaginosa*, 6.52;   *Lancefieldella parvula*, 3.5  *Streptococcus parasanguinis,* 2.08  *Streptococcus salivarius,* 0.57  Fungal total: 211  *Malassezia globosa*, 98.1 | Sample Type: ETA  Day 0:  GS: Rare WBCs, Many GPR, Moderate GPC  Cx: Heavy NRF | Day 0: No Growth |
| 52 | Clinically-diagnosed pneumonia | Day 0:  ***Haemophilus influenzae*, QP**  Day 5:  ***Haemophilus influenzae,* QP** | Day 5  Bacterial total: 2058  ***Haemophilus influenzae***, 82.07;   *Bifidobacterium longum*, 4.42;   *Cutibacterium acnes*, 2.43  Fungal total: 4326  *Candida albicans*, 96.97;   *Malassezia globosa*, 2.52;   *Candida dubliniensis*, 0.14 | Sample Type: BAL  Day 0:  GS: Moderate WBCs  Cx: No growth  RVP: negative  Sample Type: Nasopharyngeal Swab  Day -1:  RVP: negative  Sample Type: BAL  Day 3:  GS: Moderate WBCs  Cx: No growth | Day -1: No Growth |
| 53 | Clinically-diagnosed pneumonia | No Organism Detected, 0 | NA | Sample Type: BAL  Day -1:  GS: Few WBCs  Cx: No growth  RVP: negative  Sample Type: Nasopharyngeal Swab  Day -1:  RVP: negative | Day -1: No Growth |
| 54 | Clinically-diagnosed pneumonia | No Organism Detected, 0 | Day 1  Bacterial total: 254  *Staphylococcus aureus*, 74.02;   *Cutibacterium acnes*, 11.02;   *Haemophilus parainfluenzae*, 3.54  Fungal total: 2560  *Candida albicans*, 69.8;   *Malassezia globosa*, 16.76;   *[Candida] glabrata*, 13.05 | Sample Type: BAL  Day 0:  RVP: negative  Sample Type: Nasopharyngeal Swab  Day -1:  RVP: negative | Day -1: No Growth |
| 55 | Clinically-diagnosed pneumonia | *Enterococcus faecium*, 8888,  ***Staphylococcus aureus*, 481,**  ***Rothia mucilaginosa*, 472** | Day 1  Bacterial total: 447067  ***Staphylococcus aureus*, 96.97;   *Rothia mucilaginosa*, 2.35;   *Staphylococcus epidermidis*, 0.51**  Fungal total: 11206  *Candida dubliniensis*, 98.06;   *Malassezia globosa*, 1.28;   *Candida tropicalis*, 0.46  Viral total : 549  Staphylococcus virus, 100 | N/A | Day -1: No Growth |
| 56 | Clinically-diagnosed pneumonia | No Organism Detected, 0 | Day 1  Bacterial total: 58  *Cutibacterium acnes*, 63.79;   *Escherichia coli,* 5.17  Fungal total: 8  *Malassezia globosa*, 62.5;   *Candida albicans*, 25;   *Candida dubliniensis*, 12.5 | Sample Type: Nasopharyngeal Swab  Day -13:  RVP: negative | Day 0: No Growth |
| 57 | Clinically-diagnosed pneumonia | ***Streptococcus thermophilus***, 10810,  *Nesseria mucosa*, 9000,  ***Streptococcus salivarius***, 2818,  *Escherichia coli*, 1841,  *Haemophilus parainfluenzae*, 1375,  *Rothia mucilaginosa*, 1253,  *Veillonella dispar*, 760,  *Prevotella melaninogenica*, 600,  *Bacteroides uniformis*, 385 | Day 1  Bacterial total: 4830  *Enterobacter cloacae*, 33.52;   ***Streptococcus salivarius***, 30.83;   ***Streptococcus parasanguinis***, 6.75  Fungal total: 1  *Candida albicans*, 100 | Sample Type: Nasopharyngeal Swab  Day -1:  RVP: negative | Day -1:  Cx: *Enterococcus faecalis* (considered contaminant by clinical team) (1/4 bottles)  Day 0: No Growth |
| 58 | Microbiologically-confirmed pneumonia | Day 0 Sample : ^$$^  ***Streptococcus mitis***, 2775,  ***Prevotella melaninogenica***, 318,  *Haemophilus parainfluenzae*, 262,  *Gemella haemolysans*, 213,  *Veillonella dispar*, 163,  *Haemophilus parahaemolyticus*, 161,  *Haemophilus haemolyticus*, 123  Day 5:  *Veillonella parvula*, 1898;  ***Klebsiella michiganensis***, 1401  Day 10:  No Organism Detected, 0 | Day 1  Bacterial total: 2502  ***Streptococcus parasanguinis*,** 18.51;   ***Streptococcus pneumoniae***, 12.51;   ***Prevotella melaninogenica***, 7.91  Fungal total: 174  *Malassezia globosa*, 81.61;   *Metarhizium brunneum*, 6.9;   *Candida albicans*, 4.02  Day 5  Bacterial total: 35631  ***Klebsiella oxytoca***, 47.41;   *Staphylococcus aureus*, 17.23;   Delftia tsuruhatensis, 11.64  Fungal total: 1056  *[Candida] glabrata*, 84;   *Candida albicans*, 15.15;   *Malassezia globosa*, 0.57  Day 10  Bacterial total: 131161  *Delftia tsuruhatensis*, 78.47;   *Delftia acidovorans*, 9.83;   *Stenotrophomonas maltophilia*, 3.6  Fungal total: 22  *Candida albicans*, 36.36;   *[Candida] glabrata*, 31.82;   *Anthracocystis flocculosa*, 22.73 | Sample Type: BAL  Day 0:  GS: Rare WBCs, Rare GPCP  Cx: NRF  Day +1:  GS: Many WBCs, Many GNRs, Many GPCPCL  Cx: NRF, >100,00 CFU *Staphylococcus aureus,* >60,000 CFU ***Klebsiella oxytoca***  Day +5:  GS: Rare WBCs  Cx: No Growth | Day 1: No Growth  Day 2: No Growth  Day +5(06/18/2018):  No Growth |
| 59 | Microbiologically-confirmed pneumonia | *Prevotella oris*, 6145,  *Fusobacterium nucleatum*, 3158,  ***Streptococcus intermedius***, 944 | Day 1  Bacterial total: 55928  ***Streptococcus intermedius***, 74.36;   *Parvimonas micra*, 15.19;   ***Staphylococcus aureus***, 2.53  Fungal total: 3348  *[Candida] glabrata*, 74.34;   *Candida albicans*, 25.39;   *Candida tropicalis*, 0.12 | Sample Type: ETA  Day 0:  GS: Few WBCs, Few GPCP, Few GPCCH, Rare Yeast  Cx: Light ***Staphylococcus aureus***, Light NRF  Sample Type: Pleural Fluid  Day 0:  GS: Many WBCs, Many GPC  Cx; Heavy ***Streptococcus intermedius***, Moderate ***Streptococcus anginosus***, Rare ***Staphylococcus aureus*** | Day 0: No Growth  Day +1: No Growth |
| 60 | Microbiologically-confirmed pneumonia | Cytomegalovirus (CMV), 12144,  *Pseudomonas aeruginosa*, 4308,  *Enterococcus faecalis*, 830,  *Staphylococcus haemolyticus*, 800,  Herpes simplex virus type 1 (HSV-1), 424,  *Rothia mucilaginosa*, 385 | Day 1  Bacterial total: 4579  ***Stenotrophomonas maltophilia***, 64.18;   Escherichia coli, 24.5;   *Klebsiella pneumoniae,* 3.56  *Enterococcus faecalis*, 1.35  *Pseudomonas aeruginosa,* 0.46  *Staphylococcus haemolyticus*, 0.046  Fungal total: 0  NA | Sample Type: ETA  Day -1:  GS: Moderate WBCs, Few GPCP, Few GPCCL  Cx: Heavy ***Stenotrophomonas maltophilia***, Heavy NRF | Day -1: No Growth |
| 61 | Microbiologically-confirmed pneumonia | ***Staphylococcus aureus***, 475 | Day 1  Bacterial total: 1124  ***Staphylococcus aureus*,** 97.24;   *Streptococcus salivarius*, 1.42;   ***Staphylococcus epidermidis***, 0.53  Fungal total: 1  *Malassezia globosa*, 100 | Sample Type: ETA  Day +1:  GS: Many WBCs, Many GPC  Cx: Heavy ***Staphylococcus aureus*** | Day 0: No Growth |
| 62 | Microbiologically-confirmed pneumonia | Day 0:  ***Streptococcus pneumoniae***, 777  *Aggregatibacter segnis*, 209  Day 5:  ***Streptococcus pneumoniae***, 223 | Day 5  Bacterial total: 29377  *Rothia mucilaginosa*, 44.71;   *Lancefieldella parvula*, 9.83;   ***Streptococcus oralis***, 6.77  Fungal total: 21  *Malassezia globosa*, 33.33;   *Lobosporangium transversale*, 19.05;   *Anthracocystis flocculosa*, 14.29 | Sample Type: ETA  Day +1:  GS: Many WBCs; Many GPCPs, Few GNRs  Cx: Heavy ***Streptococcus pneumoniae***, Heavy *Staphylococcus aureus*  N/A | Day -1: No Growth  Day +1: No Growth |
| 63* | Microbiologically-confirmed pneumonia | ***Staphylococcus aureus***, 45941 | Day 1  Bacterial total: 7929  ***Staphylococcus aureus***, 99.29;   ***Staphylococcus epidermidis***, 0.43  Fungal total: 185  *Malassezia globosa*, 75.68;   *Candida dubliniensis*, 7.03;   Metarhizium brunneum, 5.95 | Sample Type: BAL  Day 0:  GS: Moderate WBCs  Cx: Heavy***Staphylococcus aureus*** (**MRSA)**, NRF | Day -2:  GS: GPCCL;  Cx: ***Staphylococcus aureus***; (2/4 bottles) |
| 64 | Microbiologically-confirmed pneumonia | ***Aspergillus niger***, 2986 | Day 1  Bacterial total: 66  *Escherichia coli*, 37.88;   *Staphylococcus aureus*, 18.18;   *Bacillus subtilis*, 10.61  Fungal total: 973  *Malassezia globosa*, 46.25;   *Candida orthopsilosis*, 38.13;   *Candida dubliniensis*, 4.32 | Sample Type: BAL  Day -1:  GS: Many WBCs, Few yeasts  Cx: ***Aspergillus fumigatus***  RVP: negative | Day +2: No Growth |
| 65 | Microbiologically-confirmed pneumonia | Day 0:  *Pseudomonas aeruginosa*, 4155  Day 5:  *Pseudomonas aeruginosa*, 219 | Day 1  Bacterial total: 6136  ***Staphylococcus aureus***, 44.46;   *Parvimonas micra*, 10.06;   *Olsenella uli*, 9.55  Fungal total: 197  *Malassezia globosa*, 88.83;   *Metarhizium brunneum*, 5.08;   *Lobosporangium transversale*, 4.57 | Sample Type: ETA  Day -2:  GS: Many WBCs, Few GPC, Few yeast  Cx: Light NRF, Moderate ***Staphylococcus aureus***  Sample Type: BAL  Day -1:  GS: Many WBCs  Cx: Moderate ***Staphylococcus aureus***  RVP & viral culture: negative  Sample Type: Nasopharyngeal Swab  Day -1:  RVP: negative | Day -2: No Growth |
| 66 | Microbiologically-confirmed pneumonia | **Day 0:**  ***Escherichia coli***, 6461,  *Bacteroides vulgatus*, 751  Day 5:  ***Escherichia coli***, 1135  Day 10:  Epstein-Barr virus (EBV), 238 | Day 1  Bacterial total: 326377  ***Escherichia coli*, 98.99**;   ***Escherichia albertii***, 0.16;   Shigella flexneri, 0.16  Fungal total: 241  *[Candida] glabrata*, 61;   *Malassezia globosa*, 24.07;   *Metarhizium brunneum*, 8.3 | Sample Type: ETA  Day 0:  GS: Moderate WBCs  Cx: Light ***Escherichia coli***  Sample Type: Nasopharynx  Day +4:  RVP: RSV B | Day 0: No Growth  Day +9: No Growth  Day +12: ***Escherichia coli*** |
| 67 | Microbiologically-confirmed pneumonia | ***Streptococcus agalactiae***, 656,  ***Haemophilus influenzae***, 347,  *Lactobacillus crispatus*, 274 | Day 1  Bacterial total: 50994  ***Streptococcus agalactiae***, 58.35;   ***Haemophilus influenzae***, 31.91;   *Prevotella melaninogenica*, 3.84  Fungal total: 6658  *[Candida] glabrata*, 92.72;   *Candida tropicalis,* 4.07;   *Malassezia globosa*, 2.51 | Sample Type: ETA  Day +1:  GS: Moderate WBCs, Rare yeast  Cx: Light Group B ***Streptococci*** (***Streptococcus agalactiae***), Light NRF | Day -1: No Growth |
| 68 | Microbiologically-confirmed pneumonia | *Enterococcus faecalis*, 1035,  *Morococcus cerebrosus*, 348,  *Corynebacterium striatum*, 343,  *Veillonella parvula*, 142,  *Nesseria sicca*, 132,  *Campylobacter curvus*, 116,  ***Staphylococcus aureus***, 95,  *Actinomyces viscosus*, 93,  *Malassezia furfur*, 85,  *Fusobacterium nucleatum*, 84 | Day 1  Bacterial total: 339  *Limosilactobacillus fermentum*, 39.53;   *Lactobacillus rhamnosus*, 26.55;   *Escherichia coli,* 12.39  Fungal total: 2359  *[Candida] glabrata*, 95;   *Candida dubliniensis*, 3.77;   *Candida albicans*, 1.14 | Sample Type: ETA  Day -1:  GS: Moderate WBCs  Cx: Moderate ***Staphylococcus aureus* (MRSA)**, Moderate NRF | Day -1: No Growth |
| 69 | Microbiologically-confirmed pneumonia | ***Streptococcus parasanguinius,*** QP | Day 1  Bacterial total: 186649  ***Streptococcus mitis*,** 34.9;   *Rothia mucilaginosa*, 28.36;   *Streptococcus oralis*, 8.51  Fungal total: 114  *Candida albicans,* 58.77;   *Lobosporangium transversale*, 15.79;   *Metarhizium brunneum*, 7.02 | Sample Type: BAL  Day 0:  GS: Few WBCs, Few GPCs  Cx: Light *Klebsiella* sp, NRF  RVP & viral culture: negative  Sample Type: Nasopharyngeal Swab  Day -1:  RVP: negative | N/A |
| 70 | Microbiologically-confirmed pneumonia | **Day 0:**  ***Pseudomonas aeruginosa***, 49392,  ***Aspergillus fumigatus***, 107  Day 10:  No Organism Detected, 0 | Day 1  Bacterial total: 501  ***Pseudomonas aeruginosa***, 83.63;   *Rothia mucilaginosa*, 3.39;   Streptococcus mitis, 2.59  Fungal total: 127  *Malassezia globosa,* 84.25;   *Metarhizium brunneum*, 7.09;   *Candida albicans*, 7.09 | Sample Type: BAL  ~~Day –4(2/21/2019):~~  ~~GS: Few WBCs, Few GPCP, Few GPCCl~~  ~~Cx: Moderate~~ ***~~Pseudomonas aeruginosa~~***~~,~~ ***~~Aspergillus fumigatus,~~*** ~~Heavy NRF~~  Sample Type: BAL  Day -2:  GS: Many WBCs  Cx: Light ***Pseudomonas aeruginosa***,Yeast Heavy NRF;  RVP: Positive for Influenza A Virus, subtype 2009 H1N1  Viral culture: negative  Sample Type: Nasopharyngeal Swab  Day -4:  RVP: Positive for Influenza A Virus, subtype 2009 H1N1  Influenza / RSV Rapid PCR: Influenza A positive  Day +10:  RVP: Positive for Influenza A Virus, subtype 2009 H1N1 | N/A |
| 71 | Microbiologically-confirmed pneumonia | ***Pseudomonas aeruginosa***, 1051,  *Bacteroides vulgatus*, 414,  *Bacteroides ovatus*, 180 | Day 1  Bacterial total: 5297  *Streptococcus gordonii*, 19.39;   *Rothia dentocariosa*, 15.46;   *Veillonella parvula*, 10.5  Fungal total: 1406  *[Candida] glabrata,* 95.52;   *Malassezia globosa*, 3.56;   *Candida albicans*, 0.43 | Sample Type: ETA  Day -1:  GS: Many WBCs;  Cx: Light ***Pseudomonas aeruginosa***  Sample Type: BAL  Day 0:  GS: Moderate WBCs  Cx: Yeast, not cryptococcus species, NRF  Sample Type: Bronchial wash  Day +1:  GS: Few WBCs  Cx: No Growth  RVP and viral culture: negative  Sample Type: Nasopharyngeal Swab  Day -2:  RVP: negative | Day 0: No Growth |
| 72 | Microbiologically-confirmed pneumonia | QC failure during sequencing | Day 1  Bacterial total: 89  *Rothia mucilaginosa*, 51.69;   ***Streptococcus mitis***, 17.98;   ***Streptococcus pneumoniae***, 10.11  Fungal total: 259  *Malassezia globosa*, 96.14;   *Metarhizium brunneum*, 2.7;   *Lobosporangium transversale*, 0.77 | Sample Type: BAL  Day -2:  Cx: Few *Candida* sp, Few **Alpha Hemolytic** ***Streptococci***  RVP: Positive for Influenza A Virus, subtype 2009 H1N1  Day +2:  Cx: No growth  RVP: Positive for Influenza A Virus, subtype 2009 H1N1  Sample Type: Nasopharyngeal Swab  Day -5:  Influenza / RSV rapid PCR: negative  Day -3:  RVP: positive for influenza A, subtype 2009H1N1  Day -1:  RVP: negative | Day -1: No Growth  Day +2: No Growth |
| 73 | Microbiologically-confirmed pneumonia | QC failure during sequencing | Day 1  Bacterial total: 99881  ***Staphylococcus aureus***, 80.35;   *Rothia mucilaginosa*, 13.67;   *Streptococcus parasanguinis*, 1.78  Fungal total: 261  *Candida albicans*, 79.31;   *Malassezia globosa*, 15.71;   *Metarhizium brunneum*, 3.07 | Sample Type: ETA  Day -1:  GS: Moderate WBCs, Moderate GPC  Cx: Heavy ***Staphylococcus aureus*,** Moderate NRF | Day -1: No Growth |
| 74 | Microbiologically-confirmed pneumonia | No Organism Detected, 0 | Day 1  Bacterial total: 9268  *Streptococcus mitis*, 51.12;   *Rothia mucilaginosa*, 18.31;   *Streptococcus pneumoniae*, 8.07  Fungal total: 10  *Malassezia globosa*, 60;   *Metarhizium brunneum*, 30;   *Candida albicans*, 10 | Sample Type: ETA  Day -1:  GS: Few WBCs, Moderate GPR, Moderate GPCCL  Cx: Light *Klebsiella pneumoniae*, Moderate NRF | Day -1: No Growth  Day 0: No Growth |
| 75 | Microbiologically-confirmed pneumonia | **Day 0 :**  ***Staphylococcus aureus*** (MSSA), 1375,  *Streptococcus mitis*, 123,  ***Candida albicans*, 101**,  Herpes simplex virus type 1 (HSV-1), 98,  *Gardnerella vaginalis*, 93,  *Streptococcus salivarius*, 83,  *Klebsiella variicola*, 59  Day 5 :  *Bacteroides ovatus*, 1043;  *Bacteroides thetaiotaomicron,* 443 | Day 1  Bacterial total: 9485  *Stenotrophomonas maltophilia*, 95.14;   *Stenotrophomonas* sp. WZN-1, 3;   *Pseudomonas aeruginosa*, 0.94  Fungal total: 4698  ***Candida albicans***, 99.68;   ***Candida dubliniensis***, 0.17 | Sample Type: ETA  Day -2:  GS: Many WBCs, Many GPC  Cx: Heavy ***Staphylococcus aureus***, Light NRF  Sample Type: Nasopharyngeal Swab  Day -2:  RVP: positive for Metapneumovirus | Day -2: No Growth |
| 76 | Microbiologically-confirmed pneumonia | *Streptococcus mitis*, 1312,  ***Candida tropicalis***, 372,  ***Staphylococcus aureus***, 305,  *Prevotella melaninogenica*, 264,  *Gemella morbillorum*, 228,  *Gemella sanguinis*, 197,  *Nesseria flavescens*, 108,  *Haemophilus haemolyticus*, 92,  *Nesseria mucosa*, 81,  *Gemella haemolysans*, 77 | Day 1  Bacterial total: 43181  ***Staphylococcus aureus***, 95.97;   ***Streptococcus oralis***, 0.6;   ***Streptococcus anginosus***, 0.39  Fungal total: 89  *Malassezia globosa*, 67.42;   ***Candida albicans***, 21.35;   *Metarhizium brunneum*, 7.87 | Sample Type: ETA  Day 0:  GS: Many WBCs, Many GPCP, Many GPCCL  Cx: Heavy ***Staphylococcus aureus***, Moderate NRF | Day -1: No Growth |
| 77 | Microbiologically-confirmed pneumonia | ***Enterobacter cloacae* complex**, 331992,  ***Staphylococcus aureus*** (MSSA), 24360 | Day 1  Bacterial total: 314287  ***Enterobacter cloacae***, 54.16;   ***Enterobacter hormaechei***, 42.5;   ***Staphylococcus aureus***, 1.11  Fungal total: 31  *Metarhizium brunneum*, 38.71;   *Candida albicans*, 25.81 | Sample Type: BAL  Day -1:  GS: Many GPCP, Many GNR, Few GPCCL, Few GPCCH  Cx: Heavy Carbapenem resistant ***Enterobacter cloacae* complex**. Confirmed as a carbapenemase producer. Moderate ***Staphylococcus aureus*** | Day 0: No Growth |
| 78* | Microbiologically-confirmed pneumonia | Day 0 :  *Escherichia coli*, 122894,  ***Streptococcus agalactiae*,** 6735,  ***Staphylococcus aureus***, 5151,  *Bacteroides thetaiotaomicron*, 4005  Day 5 :  *Escherichia coli*, 7565;  *Lactobacillus gasseri*, 6731;  *Streptococcus agalactiae*, 6259;  *Veillonella parvula*, 3106;  ***Staphylococcus aureus***, 2105;  Herpes simplex virus type 2 (HSV-2), 1740;  *Porphyromonas gingivalis*, 362 | Day 1  Bacterial total: 6395  **Staphylococcus aureus**, 94.92;   ***Streptococcus agalactiae***, 1.36;   *Corynebacterium kroppenstedtii*, 0.91  Fungal total: 593  Candida albicans, 84.82;   *Clavispora lusitaniae*, 14.5;   *Candida orthopsilosis*, 0.51  Day 5  Bacterial total: 196909  *Enterobacter asburiae*, 36.94;   *Enterobacter cloacae*, 24.38;   *Enterobacter* sp. E20, 17.18  Fungal total: 19927  *Candida albicans*, 92.3;   *Clavispora lusitaniae*, 6.46;   *Candida orthopsilosis*, 0.43 | Day +6: Enterobacter cloacae | Day -1:  GS: GPCCL;  Cx: ***Staphylococcus aureus*** *(1/2 bottles); Streptococcus constellatus* (2/4 bottle)  Day +1: No Growth  Day +2: No Growth  Day +6: No Growth |
| 79 | Microbiologically-confirmed pneumonia | *Enterobacter cloacae* complex, 31429,  *Atopobium vaginae*, 4946,  ***Staphylococcus aureus*** (MSSA), 1605 | Day 1  Bacterial total: 555323  *Klebsiella pneumoniae*, 96.57;   *Tropheryma whipplei*, 2.2;   *Escherichia coli*, 0.24  Fungal total: 51  *Metarhizium brunneum*, 54.9;   *Candida albicans*, 23.53;   *Candida orthopsilosis*, 15.69 | Sample Type: BAL  Day -1:  GS: Many WBCs, Few GPCP, Few GPCCL  Cx: Light *Proteus mirabilis*, Moderate ***Staphylococcus aureus*** (MRSA), NRF  RVP & viral culture: negative | Day -2: No Growth  Day -1: No Growth |
| 80 | Microbiologically-confirmed pneumonia | ***Escherichia coli***, 6733,  *Bacteroides fragilis*, 705,  *Enterococcus avium*, 293 | Day 1  Bacterial total: 3271  ***Escherichia coli***, 43.78;   Bifidobacterium breve, 27.39;   ***Klebsiella pneumoniae***, 12.29  Fungal total: 122  *[Candida] glabrata*, 50;   *Candida dubliniensis*, 46.72;   *Malassezia globosa*, 2.46 | Sample Type: ETA  Day -1:  GS: Moderate WBCs, Moderate GPR, Few GNR, Few GPCCL  Cx: Moderate ***Escherichia coli***, Moderate *Staphylococcus aureus* (MRSA), Light ***Klebsiella pneumoniae***, Moderate Group B Streptococci (Streptococcus agalactiae), Light NRF | Day -1: No Growth |
| 81 | Microbiologically-confirmed pneumonia | *Bacteroides vulgatus*, 261,  *Clostridium butyricum*, 215,  *Enterobacter cloacae* complex, 152,  *Lactobacillus gasseri*, 131,  *Escherichia coli*, 113,  *Clostridium innocuum*, 101 | Day 1  Bacterial total: 150  *Streptococcus oralis*, 15.33;   *Streptococcus parasanguinis*, 11.33;   *Klebsiella pneumoniae*, 10  ***Staphylococcus aureus,*** 7.33  Fungal total: 5  *Candida albicans*, 100 | Sample Type: ETA  Day 0:  GS: Moderate WBCs, Few GPCCH, Few GPCP  Cx: Rare ***Staphylococcus aureus*** (MRSA), Light NRF  Sample Type: BAL  Day 0:  RVP & viral culture: negative | Day 0: No Growth |
| 82 | Microbiologically-confirmed pneumonia | Day 0:  *Bifidobacterium longum*, 54711,  *Bacteroides vulgatus*, 5554,  *Sutterella wadsworthensis*, 4777  Day 5:  *Escherichia coli*, 677;  *Bacteroides thetaiotaomicron*, 435;  *Bacteroides fragilis*, 350;  *Bacteroides uniformis*, 226 | Day 1  Bacterial total: 59207  Neisseria sicca, 47.58;   ***Staphylococcus aureus***, 15.91;   Rothia mucilaginosa, 9.11  Fungal total: 11  *Malassezia globosa*, 45.45;   *Candida albicans*, 18.18;   *[Candida] glabrata*, 18.18  Day 5  Bacterial total: 135567  ***Staphylococcus aureus***, 68.33;   *Neisseria sicca*, 23.18;   *Haemophilus influenzae*, 1.94  Fungal total: 111  *Malassezia globosa*, 61.26;   *Metarhizium brunneum*, 13.51;   *Lobosporangium ransversal*, 10.81 | Sample Type: ETA  Day –1:  GS: Moderate WBCs, Many GNR, Many GPCCL, Moderate GPCP  Cx : ***Staphylococcus aureus***, NRF  Sample Type : ETA  Day +3:  GS: Many WBCs, Many GPCP and GPCL, Few GNR  Cx: Moderate **MSSA,** Light NRF | Day -1: No Growth  Day +3: No Growth |
| 83* | Microbiologically-confirmed pneumonia | ***Streptococcus mitis*, 307** | Day 1  Bacterial total: 295993  ***Staphylococcus aureus***, 60.43;   ***Streptococcus mitis***, 7.86;   *Rothia mucilaginosa*, 6.1  Fungal total: 530  *Malassezia globosa*, 68.87;   *Anthracocystis flocculosa*, 14.91;   *Candida albicans*, 5.28  Viral total : 460  *Streptococcus* phage spp., 100 | Sample Type: Nasopharyngeal Swab  Day +2:  RVP: negative | Day +2:  GS: GPCCL;  Cx: ***Staphylococcus aureus***(2/4 bottles) |
| 34 | Control | Day 0:  No Organism Detected, 0  Day 5:  No Organism Detected, 0 | Day 1  Bacterial total: 453  *Rothia mucilaginosa*, 33.33;   *Streptococcus gordonii*, 9.49;   *Streptococcus parasanguinis*, 6.62  Fungal total: 39  *Malassezia globosa*, 92.31;   *Candida albicans*, 5.13;   *Candida tropicalis*, 2.56 Day 5  Bacterial total: 11870  *Neisseria sicca*, 38.17;   *Streptococcus oralis*, 25.85;   Haemophilus influenzae, 5.27  Fungal total: 6  *Candida orthopsilosis*, 33.33;   *Candida albicans*, 33.33 | Sample Type: ETA  Day 5:  GS: Few WBCs, Rare GNR, Rare GPCP  Cx: Light NRF | Day 5:  *Staphylococcus epidermidis* |
| 84 | Control | NA | Day 1  Bacterial total: 105559  *Capnocytophaga gingivalis*, 38.49;   *Streptococcus gordonii*, 7.64;   *Rothia dentocariosa*, 6.94  Fungal total: 515  *Candida dubliniensis*, 100 | Sample Type: ETA  Day 0:  GS: WBCs  Cx: Rare NRF | Day 0: No Growth |
| 85 | Control | NA | Day 1  Bacterial total: 164245  *Moraxella catarrhalis*, 93.67;   *Moraxella bovoculi*, 1.6;   *Streptococcus mitis*, 1.26  Fungal total: 50  *Metarhizium brunneum*, 30;   *Lobosporangium transversale*, 28 | N/A  RVP: Influenza | N/A |
| 86 | Control | NA | SEQUENCING FAILURE | N/A | N/A |
| 87 | Control | Day 0:  NA | Day 1 Low bacterial reads yielded  Fungal total: 96  *Malassezia globosa*, 98.96;   *Candida albicans*, 1.04 | Sample Type: BAL  Day -2:  GS: Many WBCs, Many GPC  Cx: NRF  Sample Type: BAL  Day +1:  GS: Many WBCs  Cx: No Growth  Sample Type: nasopharyngeal swab  Day -2:  RVP: Rhino/enterovirus | Day –2: No Growth  Day –1: No Growth |
| 88 | Control | Day 0:  NA | Day 1  Bacterial total: 963  *Rothia mucilaginosa*, 45.48;  Streptococcus salivarius, 21.91;  Streptococcus parasanguinis, 8 Fungal total: 2440  Malassezia globosa, 97.17;  Candida albicans, 2.17 | N/A | Day 0(8/26/2019): No Growth  Day +1(08/27/2019): No Growth  Day +4(08/30/2019): No Growth |
| 90 | Clinically-diagnosed pneumonia | NA | Day 1  Bacterial total: 74  *Tropheryma whipplei*, 28.38;   *Staphylococcus epidermidis*, 14.86;   *Escherichia coli*, 8.11  Fungal total: 225  *Candida albicans*, 100 | Sample Type: ETA  Day -1:  GS: Rare WBCs  Cx: Light NRF | Day –1: No Growth |
| 91 | Clinically-diagnosed pneumonia | NA | Day 1  LOW BACTERIA  Fungal total: 54  *Malassezia globosa*, 94.44;   *Candida albicans*, 1.85;   *Candida tropicalis*, 1.85;   *Saccharomyces cerevisiae*, 1.85  Day 5  Bacterial total: 85745  *Escherichia coli*, 20.7;   *Staphylococcus aureus*, 19.66;   *Listeria monocytogenes*, 19.23  Fungal total: 1968  *Saccharomyces cerevisiae*, 76.98;   *Cryptococcus neoformans*, 22.97 | Sample Type: Nasopharyngeal swab  Day -1:  RVP: Rhino/Enterovirus  Sample Type: BAL  Day 0:  RVP: Negative  GS: No WBCs or organism  Cx: No Growth | Day –1: No Growth  Day +5: No Growth  Day +6:No Growth |
| 92 | Clinically-diagnosed pneumonia | NA | OVERWHELMING HUMAN DNA; SEQUENCING FAILURE (out of 367 reads collected, 27 unclasssifiable and 295 human) | Sample Type: BAL  Day -2:  GS: Many WBCs, Many GPC, GPR and GNR  Cx: Moderate NRF  Day +1 (10/10/2018):  GS: Rare WBCs  Cx: No Growth  Sample Type: ETA  Day +3 (10/12/2018):  GS: Rare WBCs  Cx: No Growth | Day +3: No Growth  Day +5: No Growth |
| 93 | Clinically-diagnosed pneumonia | Day 5:  No Organism Detected, 0 | Day 1  Bacterial total: 143  *Escherichia coli*, 33.57;   *Veillonella parvula*, 11.19;   *Lancefieldella parvula*, 9.09  Fungal total: 2  *Malassezia globosa*, 100 | Sample Type: Nasopharynx  Day -3:  RVP: Negative  Day 0:  RVP: Negative  Sample Type: BAL  Day -2:  GS: Few GPC  Cx: NRF  Day 0:  RVP: Negative  Sample Type: ETA  Day -2:  GS: Numerous GPC, few GNR  Cx: NRF  Sample Type: Bronchial washing  Day 0:  GS: Few WBCs  Cx: No Growth | Day –3: No Growth  Day +9: No Growth |
| 94 | Clinically-diagnosed pneumonia | Day 0:  *Bartonella henselae,* QP  *Streptococcus pneumoniae,* QP  Day 5:  *Bartonella henselae,* 521 | Day 5  Bacterial total: 123  *Lactococcus lactis*, 30.89;   *Enterobacter asburiae*, 14.63;   *Enterobacter cloacae*, 8.13  Fungal total: 9086  *Candida albicans*, 79.83;   *Candida tropicalis*, 19.37;   *[Candida] glabrata*, 0.56 | Sample Type: ETA  Day 0:  GS: Many WBC, Many GPC, few GPR, rare yeast  Cx: Light NRF | Day 0: No Growth |
| 95 | Clinically-diagnosed pneumonia | NA | OVERWHLEMING HUMAN DNA (51 human out of 59 reads collected) | Sample Type: ETA  Day 0:  GS: Rare WBC  Cx: No organism | Day –1: No Growth  Day 0: No Growth |
| 96 | Microbiologically-confirmed pneumonia | NA | OVERWHLEMING HUMAN DNA (18032 human out of 18120 reads collected) | Sample Type: ETA  Day -2:  GS: Many WBC  Cx: Moderate *Staphylococcus aureus*  Sample Type: ETA  Day 0:  GS: Many WBC  Cx: No organism | Day –2: No Growth  Day 0: No Growth |
| 97^+^ | Microbiologically-confirmed pneumonia | NA | Day 1  Bacterial total: 74  *Tropheryma whipplei*, 28.38;   *Staphylococcus epidermidis*, 14.86;   *Escherichia coli*, 8.11  Fungal total: 225  *Candida albicans*, 100 | Sample Type: BAL  Day 0:  RVP: Negative  Sample Type: Nasopharynx  Day 0:  RVP: Negative  Sample Type: BAL  Day 0:  GS: Moderate WBC  Cx: No Growth | N/A |
| 98 | Microbiologically-confirmed pneumonia | NA | Day 1  Bacterial total: 431  *Streptococcus oralis*, 48.26;   *Rothia mucilaginosa*, 32.02;   *Gemella* sp. oral taxon 928, 2.78  ***Staphylococcus aureus***, 0.23  Fungal total: 14  *Malassezia globosa*, 64.29;   *Candida albicans*, 28.57;   *Candida tropicalis*, 7.14 | Sample Type: ETA  Day 0 (09/21/2018):  GS: Many WBC, Many GPCCL and GPCP  Cx: Moderate **MSSA**, Light NRF | Day 0: No Growth  Day +2: No Growth |
| 99 | Microbiologically-confirmed pneumonia | NA | Day 1  Bacterial total: 211  *Rothia mucilaginosa*, 46.92;   *Streptococcus mitis*, 21.8;   *Streptococcus pneumoniae*, 5.21  Fungal total: 283  *Malassezia globosa*, 98.23;   *Metarhizium brunneum*, 0.71;   *Candida albicans*, 0.71 | Sample Type: ETA  Day -2 (03/09/2019):  GS: Moderate WBC, Few GPC  Cx: Moderate MRSA, Rare NRF | NA |

$, Blood culture has been consistently monitored for 5 days if marked as “No Growth”;

%, respiratory culture has been consistently monitored for 2 days if marked as “No Growth”;

*, subjects with bacteremia;

#, subject with subsequently biopsy-confirmed Kaposi-sarcoma, as previously reported [[11]](https://sciwheel.com/work/citation?ids=10228302&pre=&suf=&sa=0&dbf=0).

^ Positive respiratory culture considered colonization in a subject with unremarkable CXR and low Clinical Pulmonary Infection Score (CPIS<6), who was not treated with antibiotics for this positive ETA culture.

+ Patient previously diagnosed with pneumocystis pneumonia.

^$$^ Day 0 sample data included in analyses. In this subject, we had obtained a follow-up mcfDNA sample on Day 5, which revealed mcfDNA of *Klebsiella michinanesis* (1401 MPMs), likely corresponding to the cultured *Klebsiella oxytoca* by BAL, as well as mcfDNA of *Veillonella parvula* (1898 MPMs)

## Table S3 – Pathogenicity classification of microbes detected by metagenomics.

| SPECIES | Pathogenicity per NLP | Number of Manuscripts | Pathogenicity, human adjudicated | References  Journal (YEAR) [PMID] |
| --- | --- | --- | --- | --- |
| *Aspergillus fumigatus* | Established pathogen | 934 | **established** | Proc R Soc Med(1958)[13567714]; Gruzlica (1926)(1959)[14447236]; Am Rev Respir Dis(1959)[14431678]; Klin Med Osterr Z Wiss Prakt Med(1959)[13665948]; Schweiz Med Wochenschr(1961)[13699822]; Gruzlica (1926)(1962)[14459260]; Osp Maggiore(1963)[14116794]; Mykosen(1965)[5886454]; Mykosen(1965)[14298541]; Gruzlica(1966)[5979196]; Pol Med J(1967)[4965969]; Prax Pneumol(1967)[4989437]; J Clin Pathol(1967)[5614068]; Gruzlica(1968)[5663103]; Pol Przegl Chir(1968)[5701026]; Gruzlica(1969)[5804868]; Thorax(1970)[5441996]; J Assoc Physicians India(1971)[4947842]; Clin Allergy(1971)[4950529]; Folia Med Neerl(1971)[4938718]; Poumon Coeur(1971)[4341414]; Rev Immunol (Paris)(1972)[4200036]; J Thorac Cardiovasc Surg(1972)[4564491]; Scand J Respir Dis(1972)[4629597]; N Engl J Med(1973)[4565759]; Clin Allergy(1973)[4571691]; IMJ Ill Med J(1973)[4149101]; Sabouraudia(1973)[4584203]; Dimens Health Serv(1974)[4154256]; J Clin Pathol(1974)[4613736]; Medicine (Baltimore)(1975)[1186493]; Gruzlica(1975)[1089079]; Mycologia(1975)[765815]; Sabouraudia(1975)[1099699]; Respiration(1976)[778964]; Scand J Respir Dis(1976)[821142]; Am Rev Respir Dis(1976)[816237]; Dtsch Med Wochenschr(1976)[821738]; Arkh Patol(1976)[779726]; Clin Allergy(1976)[58741]; Kokyu To Junkan(1976)[794963]; Khirurgiia (Sofiia)(1976)[794564]; Br Med J(1977)[338111]; J Allergy Clin Immunol(1977)[319138]; Proc R Soc Med(1977)[122648]; Scand J Respir Dis(1977)[329408]; Schweiz Med Wochenschr(1977)[834993]; Rev Clin Esp(1977)[343198]; Acta Cytol(1977)[324215]; J Occup Med(1978)[569690]; Am Rev Respir Dis(1978)[350109]; Nihon Naika Gakkai Zasshi(1978)[351105]; Respiration(1978)[351756]; Klin Med (Mosk)(1978)[368427]; Scand J Respir Dis Suppl(1978)[98837]; J Can Assoc Radiol(1978)[363725]; J Antimicrob Chemother(1979)[385573]; J Med Soc N J(1979)[370393]; Postgrad Med J(1979)[392479]; Eur J Pediatr(1979)[436850]; South Med J(1979)[290047]; JAMA(1979)[372590]; Pol Tyg Lek(1980)[7027207]; Union Med Can(1980)[6990577]; Thorax(1980)[6992331]; Pneumonol Pol(1980)[6997834]; Poumon Coeur(1980)[6770358]; J Clin Microbiol(1980)[7372799]; Br J Dis Chest(1980)[7426370]; Arch Intern Med(1980)[7396594]; Nihon Kyobu Shikkan Gakkai Zasshi(1980)[7009943]; Am J Med(1980)[7424951]; Dtsch Med Wochenschr(1980)[6988190]; Zentralbl Bakteriol A(1980)[6779440]; Pneumonol Pol(1981)[7031619]; J Pediatr(1981)[7252678]; Chest(1981)[6788456]; Pneumonol Pol(1981)[7027194]; Pneumonol Pol(1981)[7027195]; Nihon Kyobu Shikkan Gakkai Zasshi(1981)[7026850]; Pneumonol Pol(1981)[7027197]; Rev Clin Esp(1981)[7036250]; Dtsch Med Wochenschr(1982)[6280948]; Thorax(1982)[6760449]; Nervenarzt(1982)[7110459]; Rev Clin Esp(1982)[6750707]; Laryngoscope(1982)[6953293]; Eur J Respir Dis(1982)[6754414]; Ann Intern Med(1982)[7059089]; Probl Tuberk(1982)[6760187]; Acta Pathol Microbiol Immunol Scand B(1982)[6814182]; Mykosen(1983)[6346091]; South Med J(1983)[6353603]; Histopathology(1983)[6229467]; Dakar Med(1983)[6352216]; Probl Tuberk(1983)[6356126]; Radiat Med(1984)[6400454]; J Clin Pathol(1984)[6368604]; Zh Mikrobiol Epidemiol Immunobiol(1984)[6431721]; J Allergy Clin Immunol(1984)[6438210]; Thorax(1984)[6204396]; G Ital Chemioter(1984)[6381204]; Med Clin (Barc)(1984)[6371398]; Thorax(1984)[6390774]; Diagn Microbiol Infect Dis(1984)[6443202]; Acta Pathol Microbiol Immunol Scand B(1984)[6397961]; J Asthma(1984)[6423615]; Rinsho Ketsueki(1984)[6381793]; Chest(1984)[6373174]; Internist (Berl)(1984)[6373657]; Allergol Immunopathol (Madr)(1985)[3909794]; Eur J Respir Dis(1985)[4018188]; J Infect Dis(1985)[3928772]; J Am Coll Cardiol(1985)[3894476]; Probl Tuberk(1985)[3900990]; Mykosen(1985)[3911067]; Rev Ig Bacteriol Virusol Parazitol Epidemiol Pneumoftiziol Pneumoftiziol(1985)[3012754]; Chest(1985)[3880535]; Vestn Khir Im I I Grek(1985)[3911542]; Klin Khir (1962)(1985)[3934455]; Pneumonol Pol(1985)[3914634]; Transplantation(1985)[3883596]; Klin Wochenschr(1985)[3925220]; Internist (Berl)(1985)[3897098]; Acta Pathol Microbiol Immunol Scand B(1985)[3893030]; Acta Cytol(1985)[3863422]; Schweiz Med Wochenschr(1986)[3775339]; Nihon Kyobu Shikkan Gakkai Zasshi(1986)[3543449]; Br J Dis Chest(1986)[3539169]; J Thorac Cardiovasc Surg(1986)[3097424]; Med Clin (Barc)(1986)[3540481]; Arch Intern Med(1986)[3516103]; Rev Clin Esp(1986)[3526429]; Cancer(1986)[3510710]; Isr J Med Sci(1986)[3528050]; Tubercle(1986)[3715986]; Zentralbl Bakteriol Mikrobiol Hyg A(1986)[3532632]; Vestn Khir Im I I Grek(1986)[3529583]; Rev Mal Respir(1987)[3671863]; Z Erkr Atmungsorgane(1987)[3673125]; Chest(1987)[3556066]; Morphol Embryol (Bucur)(1987)[2958697]; Thorax(1987)[3438896]; South Med J(1987)[3306949]; Dtsch Med Wochenschr(1987)[3595464]; Radiology(1987)[3317503]; J Allergy Clin Immunol(1987)[3316345]; Postgrad Med J(1987)[3328191]; Scand J Infect Dis(1987)[3303306]; Ugeskr Laeger(1987)[3330331]; Med Clin (Barc)(1987)[3312858]; N Y State J Med(1987)[3295611]; Thorax(1987)[2827334]; Am Fam Physician(1988)[3276099]; Ann Med Interne (Paris)(1988)[3056177]; Mem Inst Oswaldo Cruz(1988)[3152275]; Int Arch Allergy Appl Immunol(1988)[3281913]; Eur J Clin Microbiol Infect Dis(1988)[3132374]; Pediatr Pulmonol(1988)[2845341]; Rev Med Chil(1988)[3077208]; Schweiz Med Wochenschr(1988)[3175570]; Neurol Clin(1988)[3047541]; Pneumonol Pol(1988)[3075740]; J Hosp Infect(1988)[2896699]; Dtsch Med Wochenschr(1988)[3292187]; Rev Mal Respir(1988)[3293130]; Thorax(1989)[2595632]; Thorax(1989)[2678581]; Thorax(1989)[2648644]; Nihon Kyobu Shikkan Gakkai Zasshi(1989)[2681910]; Eur J Epidemiol(1989)[2651146]; Nihon Kyobu Shikkan Gakkai Zasshi(1989)[2681908]; Trans R Soc Trop Med Hyg(1989)[2617633]; Klin Med (Mosk)(1989)[2697785]; Allergy Proc(1989)[2647584]; Arch Fr Pediatr(1989)[2653261]; Am J Clin Pathol(1989)[2642636]; J Hosp Infect(1989)[2564014]; Kekkaku(1989)[2651750]; Am J Med(1989)[2543220]; Med Clin (Barc)(1989)[2691773]; An Med Interna(1990)[2129388]; Nihon Kyobu Shikkan Gakkai Zasshi(1990)[2290234]; Am J Cardiovasc Pathol(1990)[2095835]; Pneumologie(1990)[2367437]; Nihon Rinsho(1990)[2192129]; Lancet(1990)[1978097]; Rev Infect Dis(1990)[2385767]; Pneumologie(1990)[2367358]; Nihon Kyobu Shikkan Gakkai Zasshi(1990)[2232375]; Scand J Infect Dis(1990)[2284583]; Ter Arkh(1990)[2128860]; Kekkaku(1990)[2198374]; Arch Dis Child(1990)[2078237]; Br J Clin Pract(1990)[2282302]; Can J Infect Dis(1990)[22553447]; BMJ(1991)[2021725]; N Engl J Med(1991)[1994248]; Zhonghua Yi Xue Za Zhi(1991)[1660774]; Chest(1991)[1864142]; Chest(1991)[1889295]; N Engl J Med(1991)[1997841]; Allergy(1991)[1928661]; J Allergy Clin Immunol(1991)[1890268]; Nihon Kyobu Shikkan Gakkai Zasshi(1991)[1895586]; Ir J Med Sci(1991)[1769811]; Chest(1991)[1647938]; Kyobu Geka(1991)[2051688]; Hum Pathol(1991)[1748430]; Transplantation(1991)[1949183]; Radiology(1991)[2068304]; Kansenshogaku Zasshi(1991)[1761900]; Enferm Infecc Microbiol Clin(1991)[1932238]; J Infect Dis(1991)[1940482]; Nihon Kyobu Shikkan Gakkai Zasshi(1992)[1289634]; Nihon Kyobu Shikkan Gakkai Zasshi(1992)[1405108]; Biull Eksp Biol Med(1992)[1421313]; Clin Infect Dis(1992)[1420684]; Ann Ital Med Int(1992)[1457257]; Arerugi(1992)[1554325]; Nihon Kyobu Shikkan Gakkai Zasshi(1992)[1405113]; Eur J Pediatr(1992)[1396889]; Clin Lab Haematol(1992)[1633682]; Neth J Med(1992)[1579187]; Crit Care Med(1992)[1729033]; Pediatr Radiol(1992)[1491941]; Nihon Naika Gakkai Zasshi(1992)[1402236]; Arch Environ Health(1993)[8357277]; Thorax(1993)[8434358]; Chest(1993)[8449106]; Rev Rhum Ed Fr(1993)[8242026]; Pathol Biol (Paris)(1993)[8332393]; Ann Fr Anesth Reanim(1993)[8273926]; Rev Rhum Ed Fr(1993)[8242025]; Am J Clin Pathol(1993)[8394049]; Rev Assoc Med Bras (1992)(1993)[8281206]; Am J Med(1993)[8395142]; Nihon Kyobu Shikkan Gakkai Zasshi(1993)[8515594]; Nihon Rinsho(1993)[8492455]; Rev Pneumol Clin(1993)[8296152]; J Paediatr Child Health(1993)[8387801]; J Ky Med Assoc(1993)[8254235]; Clin Pharm(1993)[8428430]; Am Rev Respir Dis(1993)[8368653]; Nihon Kyobu Shikkan Gakkai Zasshi(1993)[8366618]; Spine (Phila Pa 1976)(1994)[7899969]; Clin Infect Dis(1994)[8011849]; Br J Neurosurg(1994)[8011191]; Clin Investig(1994)[7894223]; Nihon Kyobu Shikkan Gakkai Zasshi(1994)[8114374]; J Heart Lung Transplant(1994)[7803417]; J Allergy Clin Immunol(1994)[8064081]; Eur J Clin Microbiol Infect Dis(1994)[7915233]; Thorax(1994)[8016796]; Jpn J Antibiot(1994)[7990256]; J Rheumatol(1994)[7966071]; J Clin Pathol(1994)[7962648]; Kekkaku(1995)[7884995]; J Med Assoc Thai(1995)[8868015]; Kansenshogaku Zasshi(1995)[8586896]; Eur Respir J(1995)[7656967]; Thorax(1995)[7570421]; Arch Bronconeumol(1995)[7704395]; Arch Pediatr(1995)[7581783]; Harefuah(1995)[7744343]; Probl Tuberk(1995)[7761386]; Arch Bronconeumol(1995)[8542187]; Rev Clin Esp(1995)[7481013]; Gaoxiong Yi Xue Ke Xue Za Zhi(1995)[7674424]; Rev Pneumol Clin(1995)[7569578]; Thorax(1995)[7570424]; J Assoc Physicians India(1995)[9282671]; J Intensive Care Med(1995)[10155170]; Ann Thorac Surg(1995)[7887732]; Eur J Clin Microbiol Infect Dis(1995)[7588826]; Nihon Kyobu Geka Gakkai Zasshi(1995)[7769345]; Respir Med(1995)[7709000]; Nihon Kyobu Shikkan Gakkai Zasshi(1995)[8822006]; Ann Pharmacother(1995)[8672828]; Arch Pediatr(1995)[7640736]; Nihon Kyobu Shikkan Gakkai Zasshi(1995)[7739180]; Rev Inst Med Trop Sao Paulo(1995)[8729749]; Pneumologie(1995)[8584525]; Nephrol Dial Transplant(1996)[8672030]; Kekkaku(1996)[8753017]; Nihon Kyobu Shikkan Gakkai Zasshi(1996)[8622279]; Crit Rev Diagn Imaging(1996)[8993947]; Br J Dermatol(1996)[8763465]; Ann Pharmacother(1996)[8826557]; Mycoses(1996)[8767281]; Kansenshogaku Zasshi(1996)[8741716]; Ann Intern Med(1996)[8686977]; Mycoses(1996)[8767273]; Ter Arkh(1996)[8744115]; Eur Respir J(1996)[8834350]; An Med Interna(1996)[8948813]; Eur Respir J(1996)[8834351]; Thorax(1996)[8795683]; Int J Antimicrob Agents(1996)[18611704]; Rinsho Byori(1996)[8752728]; Mycoses(1996)[8786758]; Med Pediatr Oncol(1996)[8700001]; Enferm Infecc Microbiol Clin(1996)[8744368]; Genitourin Med(1996)[9038641]; Can Assoc Radiol J(1996)[8943916]; Pneumonol Alergol Pol(1996)[8630468]; Nihon Kyobu Geka Gakkai Zasshi(1996)[8666881]; Am J Respir Crit Care Med(1997)[9001291]; Kansenshogaku Zasshi(1997)[9248266]; Rev Med Interne(1997)[9161576]; Chest(1997)[9118736]; Ugeskr Laeger(1997)[9206859]; Nihon Kyobu Shikkan Gakkai Zasshi(1997)[9379561]; Pneumonol Alergol Pol(1997)[9489415]; Pneumonol Alergol Pol(1997)[9489424]; J Heart Lung Transplant(1997)[9154954]; Neurosurg Rev(1997)[9226675]; Kekkaku(1997)[9071093]; Nihon Kyobu Shikkan Gakkai Zasshi(1997)[9103864]; Nihon Kyobu Shikkan Gakkai Zasshi(1997)[9103855]; Pneumologie(1998)[9557055]; Allergy(1998)[9534923]; Chest(1998)[9824030]; Eur Respir J(1998)[9657566]; East Afr Med J(1998)[9803639]; Nihon Kokyuki Gakkai Zasshi(1998)[9893429]; Rev Clin Esp(1998)[9774886]; Arch Bronconeumol(1998)[9803276]; Chest(1998)[9554660]; Trop Med Int Health(1998)[9484966]; Presse Med(1998)[9856131]; Presse Med(1998)[9798465]; AJR Am J Roentgenol(1998)[9456936]; Biochem Mol Biol Int(1998)[9818093]; Pneumologie(1998)[9654974]; Med Clin (Barc)(1998)[9922954]; Enferm Infecc Microbiol Clin(1998)[9646561]; Ann Allergy Asthma Immunol(1998)[9609605]; Nihon Kokyuki Gakkai Zasshi(1998)[9754002]; J Infect(1998)[9661945]; Eur Respir J(1999)[10836345]; Ryoikibetsu Shokogun Shirizu(1999)[10201210]; Contrib Microbiol(1999)[10523262]; Mycoses(1999)[10680448]; Ryoikibetsu Shokogun Shirizu(1999)[10201209]; Ryoikibetsu Shokogun Shirizu(1999)[10201212]; Nihon Kokyuki Gakkai Zasshi(1999)[18217308]; J Med Microbiol(1999)[9989647]; Nihon Kokyuki Gakkai Zasshi(1999)[18217319]; Arch Bronconeumol(1999)[10618759]; Respirology(1999)[10489676]; Dtsch Med Wochenschr(1999)[10605422]; Intensive Care Med(1999)[10470583]; Radiology(1999)[10540673]; Ann Allergy Asthma Immunol(1999)[10353584]; Intern Med(1999)[10480308]; Med Clin (Barc)(1999)[10220762]; Med Klin (Munich)(1999)[10081288]; Pneumologie(2000)[11072723]; Arch Bronconeumol(2000)[10726189]; Clin Transplant(2000)[10945197]; Am J Gastroenterol(2000)[11051412]; Eur Radiol(2000)[10823622]; Intern Med(2000)[10732844]; Clin Microbiol Infect(2000)[11168048]; Int J Mol Med(2000)[10719062]; Arch Bronconeumol(2000)[10726182]; Asian Pac J Allergy Immunol(2000)[11316037]; Mycoses(2000)[11291572]; Med Clin (Barc)(2000)[11141396]; Ann Acad Med Singap(2000)[11056787]; Pneumonol Alergol Pol(2000)[11276977]; Ugeskr Laeger(2000)[10689950]; Transplantation(2000)[10919584]; Med Clin (Barc)(2000)[10786341]; AJR Am J Roentgenol(2000)[10882278]; Cochrane Database Syst Rev(2000)[11034746]; Tunis Med(2000)[11026827]; Intern Med(2000)[10772121]; Allergy Asthma Proc(2000)[11191100]; Vnitr Lek(2001)[11505722]; Clin Exp Allergy(2001)[11737042]; Nihon Kokyuki Gakkai Zasshi(2001)[11729687]; Indian J Med Microbiol(2001)[17664833]; Pneumonol Alergol Pol(2001)[11575005]; Transpl Infect Dis(2001)[11429033]; Pneumonol Alergol Pol(2001)[11475559]; Singapore Med J(2001)[11764059]; Rev Pneumol Clin(2001)[11416805]; Mycoses(2001)[11486459]; Rev Chir Orthop Reparatrice Appar Mot(2001)[11685151]; Nihon Kokyuki Gakkai Zasshi(2001)[11855083]; Ann Thorac Surg(2001)[11269424]; Thorax(2001)[11312407]; Med Clin (Barc)(2001)[11412696]; Rev Med Interne(2001)[11817128]; Rev Iberoam Micol(2001)[15487910]; Leuk Lymphoma(2002)[12002779]; Infect Dis Clin North Am(2002)[12512185]; Mycoses(2002)[12421281]; Jpn J Antibiot(2002)[12664928]; Braz J Med Biol Res(2002)[12131918]; Ann Thorac Surg(2002)[12643413]; Pediatr Pulmonol(2002)[11747264]; Ann Pathol(2002)[12124493]; Allergy(2002)[12121192]; Chest(2002)[11948049]; Nihon Kokyuki Gakkai Zasshi(2002)[12692945]; Indian J Med Microbiol(2002)[17657053]; South Med J(2002)[12190228]; Clin Infect Dis(2002)[12173150]; Clin Infect Dis(2002)[11753826]; Semin Respir Infect(2002)[12070828]; Nihon Kokyuki Gakkai Zasshi(2003)[12833848]; Am J Nurs(2003)[12677133]; Int J Surg Pathol(2003)[12598920]; Clin Microbiol Infect(2003)[14686988]; Am J Hematol(2003)[12508264]; Respirology(2003)[14528890]; Nihon Kokyuki Gakkai Zasshi(2003)[12931675]; Front Biosci(2003)[12456338]; Presse Med(2003)[14576582]; Allergol Immunopathol (Madr)(2003)[14572421]; Intensive Care Med(2003)[12768234]; Clin Infect Dis(2003)[12975753]; Indian J Chest Dis Allied Sci(2003)[12683713]; Chest(2003)[12970049]; Ann Hematol(2004)[14661114]; Transplant Proc(2004)[15621130]; Respiration(2004)[15316220]; Mycoses(2004)[15310341]; Mayo Clin Proc(2004)[15065620]; AJR Am J Roentgenol(2004)[15505281]; Rev Pneumol Clin(2004)[15292826]; Intern Med(2004)[15575252]; Nihon Kokyuki Gakkai Zasshi(2004)[15228135]; Korean J Intern Med(2004)[15053042]; Eur J Cardiothorac Surg(2004)[15541981]; Eur J Haematol(2005)[15946314]; J Hosp Infect(2005)[15823659]; Eur J Pediatr(2005)[15926067]; Zhonghua Er Ke Za Zhi(2005)[16191290]; J Chemother(2005)[16167522]; J Heart Lung Transplant(2005)[15653389]; J Antimicrob Chemother(2005)[15563516]; J Cyst Fibros(2005)[15914093]; Br J Haematol(2005)[15755275]; Mycoses(2005)[15826287]; An Med Interna(2005)[16386078]; Intensive Care Med(2005)[15782314]; J Heart Lung Transplant(2005)[16297812]; Mycoses(2005)[15826282]; Transpl Infect Dis(2005)[16390406]; Transplant Proc(2005)[16386634]; Rev Mal Respir(2005)[16272970]; Ter Arkh(2005)[16116913]; Kyobu Geka(2005)[15776733]; J Heart Lung Transplant(2005)[16143243]; Rev Mal Respir(2005)[16272984]; Eur J Clin Microbiol Infect Dis(2005)[16177885]; Respir Med(2005)[15939251]; MedGenMed(2005)[16369233]; Med Mycol(2005)[16110792]; Curr Opin Infect Dis(2005)[15985827]; Med Mycol(2006)[16966174]; Nihon Kokyuki Gakkai Zasshi(2006)[17087345]; Eur Respir J(2006)[16772392]; J Clin Forensic Med(2006)[16356753]; Can J Cardiol(2006)[16568159]; J Med Microbiol(2006)[17005798]; Int J Immunogenet(2006)[16893395]; Paediatr Respir Rev(2006)[16473820]; Respirology(2006)[17052313]; Indian J Med Res(2006)[16575119]; Int J Antimicrob Agents(2006)[17034993]; Nihon Kokyuki Gakkai Zasshi(2006)[16841714]; Am J Respir Crit Care Med(2006)[16959918]; Med Clin (Barc)(2006)[16602189]; Chest(2006)[17035449]; Nihon Ishinkin Gakkai Zasshi(2006)[16940951]; Ann Allergy Asthma Immunol(2006)[16802778]; Curr Opin Pulm Med(2006)[16582678]; Nihon Ishinkin Gakkai Zasshi(2006)[16465137]; Kansenshogaku Zasshi(2007)[17564118]; J Infect Chemother(2007)[17334729]; Intern Med(2007)[17541225]; Kansenshogaku Zasshi(2007)[17447480]; Pediatr Blood Cancer(2007)[16429409]; Tuberk Toraks(2007)[17602344]; Nihon Kokyuki Gakkai Zasshi(2007)[17929474]; Eur J Pediatr(2007)[16804696]; Acta Radiol(2007)[17611879]; Immunol Lett(2007)[17321603]; Transplant Proc(2007)[17954195]; Int J Obstet Anesth(2007)[16945517]; J Investig Allergol Clin Immunol(2007)[17460953]; Scand J Infect Dis(2007)[17454898]; Scand J Infect Dis(2007)[17454905]; J Infect Chemother(2007)[17721689]; Cancer(2007)[17351938]; J Med Assoc Thai(2007)[17596043]; Zhonghua Nei Ke Za Zhi(2007)[17547802]; Rev Med Liege(2007)[18286944]; J Allergy Clin Immunol(2007)[17335882]; Nihon Kokyuki Gakkai Zasshi(2007)[18186242]; Cancer(2008)[18521930]; Lupus(2008)[18413410]; Allergol Int(2008)[18493169]; Med Mycol(2008)[17885955]; Monaldi Arch Chest Dis(2008)[18507197]; Kansenshogaku Zasshi(2008)[18306679]; Nihon Kokyuki Gakkai Zasshi(2008)[18516997]; Pediatr Blood Cancer(2008)[18478572]; J Neurosurg Sci(2008)[18500218]; Mikrobiyol Bul(2008)[18444575]; Transpl Infect Dis(2008)[18194369]; Arch Bronconeumol(2008)[18559224]; Rev Iberoam Micol(2008)[18785789]; Infection(2008)[19011743]; Pharmacotherapy(2008)[18657024]; J Pak Med Assoc(2008)[18846804]; Pneumonol Alergol Pol(2008)[19003771]; Rev Alerg Mex(2008)[19058490]; Zhonghua Nei Ke Za Zhi(2008)[19134308]; Transplant Proc(2008)[19010204]; AIDS Patient Care STDS(2008)[18095836]; J Cyst Fibros(2008)[17693140]; Chest(2009)[19265090]; Infection(2009)[19730788]; J Heart Lung Transplant(2009)[19416784]; Pneumonol Alergol Pol(2009)[19722146]; Respirology(2009)[19909460]; Nihon Kokyuki Gakkai Zasshi(2009)[19514502]; Nihon Kokyuki Gakkai Zasshi(2009)[19348267]; Ned Tijdschr Geneeskd(2009)[20051169]; Br J Radiol(2009)[19592397]; Nihon Kokyuki Gakkai Zasshi(2009)[19514508]; Infect Control Hosp Epidemiol(2009)[19848605]; J Bras Pneumol(2009)[19547859]; Singapore Med J(2009)[19421672]; Emerg Infect Dis(2009)[19751595]; Arch Bronconeumol(2009)[19303524]; Nihon Kokyuki Gakkai Zasshi(2009)[19455964]; Nihon Kokyuki Gakkai Zasshi(2009)[19827582]; Clin Infect Dis(2009)[19489714]; Clin J Gastroenterol(2009)[26192799]; Zhonghua Jie He He Hu Xi Za Zhi(2009)[19957779]; Arch Bronconeumol(2009)[19376624]; Nihon Kokyuki Gakkai Zasshi(2010)[21141064]; Intern Med(2010)[20558936]; J Indian Med Assoc(2010)[21661466]; Ir Med J(2010)[21560502]; Clin Chest Med(2010)[20692546]; Acta Derm Venereol(2010)[21057748]; Mikrobiyol Bul(2010)[20549962]; Clin Microbiol Infect(2010)[19673966]; Nihon Kokyuki Gakkai Zasshi(2010)[20803983]; Eur J Pediatr(2010)[19657670]; J Med Microbiol(2010)[20299503]; Med Mycol(2010)[20055745]; Am J Trop Med Hyg(2010)[20207859]; Emerg Infect Dis(2010)[20507748]; Diagn Pathol(2010)[20205795]; Minerva Anestesiol(2010)[21102391]; Rev Iberoam Micol(2010)[20346298]; Antimicrob Agents Chemother(2010)[20439610]; Intern Med J(2011)[22435902]; Clin Dev Immunol(2011)[21603163]; J Chemother(2011)[22005062]; Zhonghua Jie He He Hu Xi Za Zhi(2011)[22177489]; Clin Infect Dis(2011)[21467016]; S Afr Med J(2011)[22272860]; J Korean Neurosurg Soc(2011)[22200027]; Eur J Clin Microbiol Infect Dis(2011)[21541671]; Eur Respir J(2011)[20595150]; Clin Microbiol Infect(2011)[21668573]; Eur Spine J(2011)[20596734]; J Res Med Sci(2011)[22091235]; Pathol Res Pract(2011)[21978481]; Scand J Work Environ Health(2011)[21327319]; Zhonghua Nei Ke Za Zhi(2011)[21600085]; Acta Paediatr(2011)[20722636]; Transplant Proc(2011)[21440756]; Nihon Rinsho(2011)[21838048]; Respir Med(2011)[21824758]; Transplant Proc(2011)[21839284]; Transpl Infect Dis(2011)[21457421]; Acta Clin Belg(2011)[21837936]; Asian Cardiovasc Thorac Ann(2011)[21357323]; Eur J Clin Microbiol Infect Dis(2012)[21997774]; Rev Med Chil(2012)[23096664]; Pediatr Transplant(2012)[22489792]; Transpl Infect Dis(2012)[22988985]; Mycopathologia(2012)[22457034]; Clin Infect Dis(2012)[22247307]; Antimicrob Agents Chemother(2012)[22123701]; Enferm Infecc Microbiol Clin(2012)[22079232]; Allergol Int(2012)[22722814]; Ann Biol Clin (Paris)(2012)[22294141]; Toxicol Ind Health(2012)[22082828]; Chin Med J (Engl)(2012)[22613594]; Euro Surveill(2012)[23218390]; Medicine (Baltimore)(2012)[22932790]; J Cyst Fibros(2012)[22342403]; Rev Pneumol Clin(2012)[22305135]; Rev Mal Respir(2012)[22440310]; Enferm Infecc Microbiol Clin(2012)[22341751]; Rev Peru Med Exp Salud Publica(2012)[22858775]; Oman Med J(2012)[23226822]; J Infect(2012)[22898389]; Med Mycol(2012)[22149972]; Rev Iberoam Micol(2012)[22463784]; Rev Iberoam Micol(2012)[22108606]; BMC Infect Dis(2012)[23145899]; J Bronchology Interv Pulmonol(2012)[23207537]; Transplantation(2012)[22805441]; Rev Chilena Infectol(2012)[23096474]; Acta Clin Belg(2012)[22480040]; Monaldi Arch Chest Dis(2012)[22662646]; Cochrane Database Syst Rev(2012)[22696329]; Respir Care(2012)[22417634]; Eur J Cardiothorac Surg(2012)[21601471]; PLoS One(2012)[22558432]; Zhonghua Jie He He Hu Xi Za Zhi(2012)[22455941]; Zhonghua Er Ke Za Zhi(2012)[23302613]; Antimicrob Agents Chemother(2013)[23403435]; Intern Med(2013)[23903514]; Scientifica (Cairo)(2013)[24278780]; Chin Med J (Engl)(2013)[24033938]; Adv Exp Med Biol(2013)[23835992]; Thorax(2013)[23513028]; Mycopathologia(2013)[23615821]; Case Rep Transplant(2013)[23984170]; PLoS Pathog(2013)[24348239]; Infect Chemother(2013)[24265971]; Eur Respir J(2013)[23100491]; Clin Exp Allergy(2013)[23889240]; Rev Iberoam Micol(2013)[22986228]; Antimicrob Agents Chemother(2013)[23669377]; Chest(2013)[23276848]; Pediatr Transplant(2013)[23489441]; J Bronchology Interv Pulmonol(2013)[23609249]; J Allergy Clin Immunol(2013)[23726262]; Transplant Proc(2013)[23953550]; Future Microbiol(2013)[24199804]; Balkan Med J(2013)[25207153]; An Pediatr (Barc)(2013)[23116554]; N Engl J Med(2013)[23514292]; PLoS Pathog(2013)[24204250]; Transplant Proc(2013)[24314931]; Zhonghua Jie He He Hu Xi Za Zhi(2013)[24433801]; Mycoses(2013)[23560622]; Intern Med(2013)[24042528]; Infection(2013)[23463186]; Acta Clin Belg(2013)[24579244]; Occup Med (Lond)(2013)[23975883]; Onkologie(2013)[24107913]; Curr Opin Pulm Med(2013)[23411576]; IDCases(2014)[26839766]; BMJ Case Rep(2014)[25385562]; J Clin Diagn Res(2014)[24959447]; BMJ Case Rep(2014)[25371437]; Mycoses(2014)[24673772]; Zhonghua Jie He He Hu Xi Za Zhi(2014)[25533690]; J Clin Microbiol(2014)[24829237]; Lung India(2014)[24778481]; Lung India(2014)[25125822]; Med Mycol Case Rep(2014)[25379388]; Kansenshogaku Zasshi(2014)[25764808]; BMJ Case Rep(2014)[24862414]; Radiologia(2014)[24735895]; Cochrane Database Syst Rev(2014)[25431975]; Mymensingh Med J(2014)[24858168]; Clin Infect Dis(2014)[25342502]; Cold Spring Harb Perspect Med(2014)[25377144]; Mycopathologia(2014)[24947170]; Clin Microbiol Infect(2014)[24303995]; Ann Pharmacother(2014)[24939635]; PLoS One(2014)[25072733]; Curr Protein Pept Sci(2014)[24818760]; BMJ Case Rep(2014)[24850547]; J Thorac Dis(2014)[24624284]; Ups J Med Sci(2014)[24195576]; J Infect Chemother(2014)[24477329]; Allergy Asthma Proc(2014)[24717781]; Chest(2014)[24687706]; Exp Clin Transplant(2014)[24702140]; Med Mycol(2014)[24719455]; J Med Case Rep(2014)[25412755]; Int J Biochem Cell Biol(2014)[24625547]; Zhonghua Wei Zhong Bing Ji Jiu Yi Xue(2014)[25230864]; J Glob Antimicrob Resist(2015)[27873672]; Diagn Interv Imaging(2015)[25753544]; Expert Rev Respir Med(2015)[25547335]; Thorac Cardiovasc Surg Rep(2015)[26693131]; J Bras Pneumol(2015)[26578140]; Semin Respir Crit Care Med(2015)[25826588]; Int J Clin Exp Med(2015)[26884969]; Clin Transplant(2015)[25604399]; Indian J Med Res(2015)[26112836]; Expert Rev Hematol(2015)[25959740]; J Clin Diagn Res(2015)[26816937]; Med Mycol Case Rep(2015)[26199867]; Med Mycol Case Rep(2015)[25834787]; Tuberc Respir Dis (Seoul)(2015)[25861349]; J Infect Chemother(2015)[25828927]; Drug Resist Updat(2015)[26282594]; Med Mycol(2015)[25851262]; Mycoses(2015)[26214496]; Respirology(2015)[25819403]; Eur J Clin Microbiol Infect Dis(2015)[26003310]; Antimicrob Agents Chemother(2015)[25987612]; BMC Pulm Med(2015)[26420256]; Exp Clin Transplant(2015)[25894190]; Infect Dis Clin Pract (Baltim Md)(2015)[26392737]; J Clin Pharm Ther(2015)[26248976]; Exp Clin Transplant(2015)[25894189]; Ann Am Thorac Soc(2015)[25513736]; Ned Tijdschr Geneeskd(2015)[25714763]; Arab J Gastroenterol(2015)[26206431]; Surg Neurol Int(2015)[26600985]; Transplant Proc(2015)[26036497]; BMJ Case Rep(2015)[26123468]; Exp Clin Transplant(2015)[25894185]; PLoS One(2015)[26629994]; Arch Pediatr(2016)[28231891]; Med Mycol Case Rep(2016)[28053849]; Respir Investig(2016)[26879476]; Can Respir J(2016)[27445566]; Allergol Int(2016)[26740298]; Monaldi Arch Chest Dis(2016)[27374215]; Intern Med(2016)[27041173]; Eur Ann Allergy Clin Immunol(2016)[27152607]; Ann Am Thorac Soc(2016)[27348071]; Cochrane Database Syst Rev(2016)[27820955]; Am J Transplant(2016)[27105907]; Contemp Oncol (Pozn)(2016)[27095942]; Pneumonol Alergol Pol(2016)[27238177]; Antimicrob Agents Chemother(2016)[26574014]; Med Mycol(2016)[27364649]; Eur Ann Allergy Clin Immunol(2016)[27608478]; Egypt J Immunol(2016)[28502150]; Respirol Case Rep(2016)[26839700]; Exp Clin Transplant(2016)[27805531]; Lung India(2016)[26933300]; Nihon Rinsho Meneki Gakkai Kaishi(2016)[27181240]; Surg Neurol Int(2016)[27308089]; Int J Mycobacteriol(2016)[28043539]; PLoS One(2016)[27861524]; Bone Marrow Transplant(2017)[27941772]; J Infect Chemother(2017)[28109740]; Respir Med Case Rep(2017)[28053855]; Rev Esp Enferm Dig(2017)[28229616]; Mycoses(2017)[28139853]; Arch Bronconeumol(2017)[28024667]; Med Mycol Case Rep(2017)[28761803]; Medicine (Baltimore)(2017)[29245249]; Medicine (Baltimore)(2017)[29390575]; Int J Hyg Environ Health(2017)[28529020]; Allergy(2017)[28513848]; Am J Case Rep(2017)[28377567]; Otolaryngol Head Neck Surg(2017)[28440108]; Mycoses(2017)[28660636]; J Infect Dis(2017)[28911045]; Arch Bronconeumol(2017)[28495076]; Mycoses(2017)[28504471]; J Cyst Fibros(2017)[28185887]; J Med Case Rep(2017)[28797271]; Int J Health Sci (Qassim)(2017)[28539863]; Cent Eur J Immunol(2017)[28680340]; Int J Tuberc Lung Dis(2017)[28826456]; Respir Med Case Rep(2017)[28393004]; Medicine (Baltimore)(2017)[29049191]; Rev Esp Quimioter(2017)[28176520]; J Clin Diagn Res(2017)[28384860]; Case Rep Cardiol(2017)[28316844]; Ann Thorac Med(2017)[28469716]; BMC Pulm Med(2017)[28431569]; J Cyst Fibros(2017)[27356848]; J Thorac Dis(2017)[28449484]; Mycoses(2018)[29920796]; Indoor Air(2018)[29082624]; Mycoses(2018)[29687483]; Allergol Int(2018)[29773475]; Mycoses(2018)[30015364]; Surg Infect (Larchmt)(2018)[30227087]; Microb Pathog(2018)[29410233]; J Allergy Clin Immunol Pract(2018)[28939137]; MMWR Morb Mortal Wkly Rep(2018)[30260939]; Mycopathologia(2018)[29858759]; Eur J Clin Microbiol Infect Dis(2018)[29754210]; BMC Infect Dis(2018)[29996788]; J Infect Public Health(2018)[28780309]; J Mycol Med(2018)[30366642]; Iran J Public Health(2018)[30181998]; Clin Infect Dis(2018)[29684106]; Respirol Case Rep(2018)[30065838]; Eur Respir J(2018)[29496757]; Front Microbiol(2018)[29666610]; Respirology(2018)[29502335]; Front Microbiol(2018)[30083151]; Front Microbiol(2018)[29686661]; Mycoses(2018)[29570855]; Med Mycol(2018)[28525619]; Medicine (Baltimore)(2018)[30412142]; Am J Transplant(2018)[29790292]; Intern Med(2018)[29607975]; Rev Chil Pediatr(2018)[29799893]; Clin Case Rep(2018)[30564352]; Mycopathologia(2018)[29067630]; BMC Infect Dis(2018)[30541477]; J Infect(2018)[29248586]; Med Mycol Case Rep(2018)[29188178]; Rev Iberoam Micol(2019)[30503225]; Turk Patoloji Derg(2019)[29630082]; Clin Infect Dis(2019)[30307492]; Allergy Asthma Proc(2019)[31690385]; Respirol Case Rep(2019)[31406579]; Intern Med(2019)[30930349]; Allergol Int(2019)[30670338]; J Infect Chemother(2019)[30503017]; Pediatr Pulmonol(2019)[30838817]; J Cyst Fibros(2019)[30638825]; Transpl Infect Dis(2019)[31529558]; Transplant Proc(2019)[30879588]; J Infect Chemother(2019)[30824299]; Rev Port Cardiol (Engl Ed)(2019)[31495716]; J Fungi (Basel)(2019)[31266196]; Int J Neurosci(2019)[30430892]; Case Rep Oncol Med(2019)[31534809]; Clin Microbiol Infect(2019)[30986554]; Clin Lab(2019)[31232022]; Mymensingh Med J(2019)[31599261]; HIV AIDS (Auckl)(2019)[31920403]; Allergy(2019)[30793327]; J Lab Physicians(2019)[31929707]; Klin Mikrobiol Infekc Lek(2019)[31971247]; Pathogens(2019)[31877884]; Chin Neurosurg J(2019)[32922917]; Pediatr Infect Dis J(2019)[29912843]; Front Microbiol(2019)[31447794]; Clin Microbiol Rev(2019)[31722890]; J Pediatr Hematol Oncol(2019)[30095689]; Am J Transl Res(2019)[31105860]; Environ Res(2020)[31902481]; BMC Infect Dis(2020)[32703183]; Am J Case Rep(2020)[33361740]; Curr Opin Infect Dis(2020)[32657965]; Med Mycol(2020)[30877757]; J Hosp Infect(2020)[31585141]; J Clin Microbiol(2020)[33087440]; J Mycol Med(2020)[32008965]; Mycoses(2020)[32677131]; Intern Med(2020)[31866624]; Monaldi Arch Chest Dis(2020)[32945640]; BMC Infect Dis(2020)[32807082]; J Med Microbiol(2020)[32459615]; J Diabetes Investig(2020)[31758642]; bioRxiv(2020)[33173866]; Med Mycol Case Rep(2020)[31890489]; J Fungi (Basel)(2020)[32443672]; Antimicrob Resist Infect Control(2020)[32393344]; Microorganisms(2020)[33379247]; Asia Pac Allergy(2020)[32789112]; Acta Clin Belg(2020)[31179880]; BMC Cancer(2020)[31906982]; Pathogens(2020)[32878014]; Medicine (Baltimore)(2020)[31914069]; Pathogens(2020)[32781694]; Int J Mycobacteriol(2020)[33323668]; Am J Respir Crit Care Med(2020)[32191838]; J Thorac Dis(2020)[32274153]; J Fungi (Basel)(2020)[33114653]; Mycoses(2020)[32648614]; J Fungi (Basel)(2020)[33371198]; J Antimicrob Chemother(2020)[32862231]; Mycoses(2020)[32749040]; Respirol Case Rep(2020)[32025305]; BMC Infect Dis(2020)[31906888]; Mycoses(2020)[32918365]; Int Med Case Rep J(2020)[32210640]; Cureus(2020)[32923277]; Front Microbiol(2020)[32849346]; J Fungi (Basel)(2020)[33291706]; mBio(2021)[33436426]; Mycopathologia(2021)[34490551]; Ann Clin Microbiol Antimicrob(2021)[34174895]; Rev Port Cardiol (Engl Ed)(2021)[33303301]; Med Mycol Case Rep(2021)[34804785]; Medicine (Baltimore)(2021)[34397685]; Mycoses(2021)[33210776]; BMC Infect Dis(2021)[34126952]; BMJ Case Rep(2021)[34340987]; J Microbiol Immunol Infect(2021)[33012653]; Transpl Infect Dis(2021)[33185971]; WMJ(2021)[33974773]; Med Mycol(2021)[33418565]; BMJ Case Rep(2021)[33837026]; N Z Med J(2021)[34140712]; Am J Respir Crit Care Med(2021)[33021398]; Mycopathologia(2021)[34052941]; Mycoses(2021)[34181773]; J Fungi (Basel)(2021)[34209322]; Rev Iberoam Micol(2021)[33775537]; Med Mycol Case Rep(2021)[32837879]; J Fungi (Basel)(2021)[33808931]; Transplant Direct(2021)[34113715]; J Fungi (Basel)(2021)[34682297]; Cureus(2021)[33824833]; Microorganisms(2021)[33669831]; Scand J Clin Lab Invest(2021)[34278893]; Surg Case Rep(2021)[33796902]; J Fungi (Basel)(2021)[33435452]; Cureus(2021)[34527462]; J Mycol Med(2021)[34428666]; Diagnostics (Basel)(2021)[34359309]; Cureus(2021)[34268029]; J Mycol Med(2021)[34147761]; BMJ Open Respir Res(2021)[34385150]; Respirol Case Rep(2021)[34194813]; Front Immunol(2021)[34239516]; Infection(2021)[33709380]; Mycoses(2021)[33569857]; Antibiotics (Basel)(2021)[33800658]; Respir Med(2021)[33611086]; J Fungi (Basel)(2021)[33670173]; J Fungi (Basel)(2021)[33418997]; Eur J Clin Microbiol Infect Dis(2021)[33140176]; Intern Med(2022)[34373373]; J Cyst Fibros(2022)[34332906]; Fetal Pediatr Pathol(2022)[32619123]; J Pharm Pract(2022)[33084474]; Respirol Case Rep(2022)[34888059]; Retin Cases Brief Rep(2022)[32004182] |
| *Aspergillus niger* | Established pathogen | 118 | **established** | Am J Clin Pathol(1975)[1199978]; Cancer(1975)[1060508]; Scand J Respir Dis(1976)[821142]; Dtsch Med Wochenschr(1976)[821738]; Acta Cytol(1979)[285553]; J Clin Microbiol(1980)[7372799]; Chest(1981)[6788456]; Am J Clin Pathol(1981)[7293976]; Am Rev Respir Dis(1984)[6703507]; Zh Mikrobiol Epidemiol Immunobiol(1984)[6431721]; Mykosen(1984)[6521752]; Allergol Immunopathol (Madr)(1985)[3909794]; Med Clin (Barc)(1985)[4033255]; Probl Tuberk(1985)[3900990]; Am Rev Respir Dis(1985)[4003949]; Acta Cytol(1985)[3863422]; Arch Pathol Lab Med(1986)[3778147]; Vestn Khir Im I I Grek(1986)[3529583]; Nihon Kyobu Shikkan Gakkai Zasshi(1987)[3599574]; J Allergy Clin Immunol(1988)[3123541]; Biomed Pharmacother(1989)[2790149]; Klin Med (Mosk)(1989)[2697785]; Thorax(1989)[2763249]; Crit Care Med(1990)[2328603]; Ter Arkh(1990)[2128860]; Med Clin (Barc)(1991)[1766284]; Pneumonol Alergol Pol(1991)[1843924]; Chest(1992)[1541168]; Pathol Biol (Paris)(1993)[8332393]; Kansenshogaku Zasshi(1994)[8051447]; Ter Arkh(1995)[8571247]; Am J Hematol(1995)[7771465]; Bull Soc Pathol Exot(1995)[8555766]; Rinsho Byori(1996)[8752728]; Kansenshogaku Zasshi(1997)[9248266]; J Infect Chemother(1999)[11810498]; Thorax(1999)[10525567]; Nihon Kokyuki Gakkai Zasshi(1999)[18217308]; Ann Allergy Asthma Immunol(1999)[10353584]; Arch Bronconeumol(2000)[10726199]; Clin Transplant(2000)[10945197]; Jpn J Thorac Cardiovasc Surg(2000)[10824482]; Transplantation(2000)[10919584]; Respir Med(2001)[11392574]; Nihon Kokyuki Gakkai Zasshi(2001)[11481826]; Med Lav(2001)[11515153]; Wien Klin Wochenschr(2001)[15503626]; Mycoses(2002)[12421281]; Ann Pathol(2002)[12124493]; Semin Respir Infect(2002)[12070828]; Int J Surg Pathol(2003)[12598920]; Nihon Kokyuki Gakkai Zasshi(2004)[15357273]; Mycoses(2004)[15078435]; J Cyst Fibros(2005)[15914093]; Zhonghua Er Ke Za Zhi(2005)[15833167]; Med Mycol(2005)[16110792]; Ter Arkh(2005)[16116913]; Intern Med(2005)[15805716]; J Heart Lung Transplant(2005)[16143277]; Med Mycol(2006)[16966174]; Respirology(2006)[17052313]; Eur J Intern Med(2006)[16762784]; Acta Biomed(2006)[17370569]; Tokai J Exp Clin Med(2006)[21302231]; Nihon Ishinkin Gakkai Zasshi(2006)[16940951]; J Infect Chemother(2006)[17235646]; Niger Postgrad Med J(2006)[17278326]; Tuberk Toraks(2007)[17602344]; Clin Med Case Rep(2008)[24179349]; J Asthma(2009)[19191146]; Nihon Kokyuki Gakkai Zasshi(2009)[19348267]; Mycoses(2009)[18643917]; Mikrobiyol Bul(2010)[20549962]; Respir Med(2011)[21824758]; Am J Respir Crit Care Med(2012)[23155220]; Rev Iberoam Micol(2012)[22108606]; J Infect(2012)[22898389]; Rev Chilena Infectol(2012)[23096474]; BMC Infect Dis(2012)[23031334]; J Med Case Rep(2012)[22333492]; J Thorac Dis(2013)[23991333]; Am J Emerg Med(2013)[22795409]; Ear Nose Throat J(2014)[25025412]; J Infect Chemother(2014)[24477329]; J Trop Pediatr(2014)[24531376]; BMC Infect Dis(2015)[26567015]; PLoS One(2015)[26230666]; PLoS One(2015)[26629994]; Med Mycol(2016)[26773134]; Bull Soc Pathol Exot(2016)[27848101]; Mycopathologia(2016)[26666549]; Int J Mycobacteriol(2016)[28043539]; J Clin Diagn Res(2016)[27790435]; Medicine (Baltimore)(2017)[29245249]; Int J Health Sci (Qassim)(2017)[28539863]; J Clin Diagn Res(2017)[28384860]; Med Mycol Case Rep(2017)[28560131]; Allergol Int(2018)[29773475]; BMC Res Notes(2018)[29776430]; Front Microbiol(2018)[29666610]; Front Vet Sci(2018)[30370271]; Respir Med Case Rep(2018)[29276675]; Pan Afr Med J(2018)[30574254]; Rev Mal Respir(2019)[31010756]; Health Sci Rep(2019)[31768420]; Indian J Thorac Cardiovasc Surg(2019)[33061034]; Pan Afr Med J(2019)[31489098]; BMC Infect Dis(2020)[32703183]; Case Rep Pediatr(2020)[33014499]; Antimicrob Resist Infect Control(2020)[32393344]; West Afr J Med(2020)[32150628]; Pediatr Pulmonol(2020)[32130796]; Mycoses(2020)[32918365]; Case Rep Infect Dis(2020)[33457026]; Mycopathologia(2021)[34052941]; Clin Lab(2021)[33616322]; Intern Emerg Med(2021)[33751395]; Respir Med Case Rep(2021)[33619451] |
| *BK polyomavirus* | Possible patho, intermediate # of evidence | 17 | **established** | Recenti Prog Med(2002)[11989130]; Transplantation(2004)[15087764]; Hum Pathol(2005)[16153469]; Clin Infect Dis(2006)[17083023]; Pediatr Transplant(2007)[17910663]; Am J Transplant(2010)[20840474]; Clin Exp Nephrol(2010)[20714773]; Transpl Infect Dis(2012)[22998078]; J Clin Virol(2012)[22959064]; Saudi J Kidney Dis Transpl(2014)[25193905]; Br J Cancer(2016)[27632373]; J Heart Lung Transplant(2017)[27914897]; Transpl Infect Dis(2018)[29064138]; Ann Transplant(2018)[29748530]; J Infect Chemother(2019)[31027885]; BMC Infect Dis(2020)[32795251]; Transplantation(2021)[33323766] |
| *Citrobacter freundii* | Possible patho, intermediate # of evidence | 33 | **established** | Chest(1975)[1149508]; Am J Clin Pathol(1978)[696671]; Can J Microbiol(1979)[526888]; Rev Clin Esp(1985)[4001494]; Am J Perinatol(1989)[2730730]; DICP(1991)[2008783]; Nihon Kyobu Shikkan Gakkai Zasshi(1992)[1630056]; Nihon Kyobu Shikkan Gakkai Zasshi(1994)[7815761]; Kansenshogaku Zasshi(1994)[8151148]; Nihon Kyobu Shikkan Gakkai Zasshi(1994)[7799554]; Indian Pediatr(1995)[8613295]; Microb Drug Resist(1995)[9158804]; Ann Urol (Paris)(1996)[8766145]; Antimicrob Agents Chemother(1998)[9687398]; Crit Care Med(2001)[11246310]; Recenti Prog Med(2004)[15032335]; Pharmacotherapy(2007)[17253909]; Eur J Clin Microbiol Infect Dis(2007)[17587073]; J Antimicrob Chemother(2007)[17540673]; J Clin Microbiol(2007)[17715376]; J Infect(2007)[16815552]; Eur J Clin Microbiol Infect Dis(2009)[18682995]; Clin Ther(2009)[19302902]; Acta Med Iran(2010)[21287478]; Clin Microbiol Infect(2011)[20345467]; Transplantation(2014)[24162251]; PLoS One(2015)[26407326]; Zhonghua Nei Ke Za Zhi(2015)[26675021]; Infect Disord Drug Targets(2017)[27658860]; Pak J Pharm Sci(2017)[29175778]; Antimicrob Agents Chemother(2018)[29203488]; J Cardiovasc Echogr(2020)[33282654]; Biomed Res Int(2021)[34136573] |
| *Citrobacter koseri* | Possible patho, intermediate # of evidence | 5 | **established** | Isr J Med Sci(1984)[6511332]; Eur J Clin Microbiol Infect Dis(2009)[18682995]; Nihon Hinyokika Gakkai Zasshi(2018)[31006742]; Antimicrob Resist Infect Control(2018)[29541448]; Biomed Res Int(2021)[34136573] |
| *Cryptococcus neoformans* | Established pathogen | 397 | **established** | Bras Med(1952)[14954067]; Am Rev Tuberc(1956)[13354928]; Prensa Med Argent(1956)[13359305]; Dtsch Med Wochenschr(1971)[5088868]; Am Rev Respir Dis(1972)[4562360]; Arch Monaldi(1972)[4615649]; Nihon Kyobu Shikkan Gakkai Zasshi(1973)[4588356]; J Indiana State Med Assoc(1973)[4589050]; South Med J(1976)[1246649]; Schweiz Med Wochenschr(1976)[1036794]; Schweiz Arch Neurol Neurochir Psychiatr(1977)[341300]; Cancer(1977)[322854]; N Engl J Med(1977)[337142]; Clin Allergy(1978)[152171]; Virchows Arch A Pathol Anat Histol(1979)[157594]; Am Rev Respir Dis(1980)[6992663]; Ann Intern Med(1981)[7235394]; Dtsch Med Wochenschr(1981)[7261926]; Rev Inst Med Trop Sao Paulo(1981)[7345538]; Med Trop (Mars)(1982)[7154905]; Aust N Z J Med(1982)[6758749]; Clin Allergy(1982)[6749330]; Ann Pediatr (Paris)(1983)[6600905]; N Engl J Med(1983)[6217423]; Am J Med Sci(1984)[6517091]; J Infect(1984)[6527047]; Medicine (Baltimore)(1984)[6325849]; Ann Intern Med(1985)[2986505]; Del Med J(1985)[3987945]; Am Rev Respir Dis(1985)[4003948]; Am J Med(1985)[3985041]; J Thorac Cardiovasc Surg(1986)[3784593]; Chest(1986)[3709257]; Radiology(1986)[3489955]; Diagn Cytopathol(1986)[3021408]; Semin Respir Infect(1987)[2825316]; Arch Pathol Lab Med(1987)[3541845]; Nervenarzt(1988)[3185845]; Infect Dis Clin North Am(1988)[3060529]; Rev Med Liege(1988)[3060933]; West J Med(1988)[3266812]; Neurol Clin(1988)[3047541]; Clin Chest Med(1988)[3044680]; Clin Pharm(1988)[3383545]; J Neurol(1989)[2915224]; Klin Wochenschr(1989)[2545969]; Plucne Bolesti(1989)[2798562]; Semin Respir Infect(1989)[2652235]; Nihon Kyobu Shikkan Gakkai Zasshi(1989)[2696803]; Chest(1989)[2721249]; Kansenshogaku Zasshi(1989)[2509595]; J Acquir Immune Defic Syndr (1988)(1990)[2324944]; Mycoses(1990)[2352543]; Schweiz Rundsch Med Prax(1990)[2187221]; N C Med J(1990)[2342607]; Chest(1990)[2225944]; J Thorac Imaging(1990)[2325190]; Semin Respir Infect(1990)[2188321]; Mycoses(1990)[1965550]; Chest(1990)[2361410]; Rev Med Chil(1990)[2131506]; Rev Mal Respir(1991)[1775717]; Diagn Cytopathol(1991)[1935520]; Am J Med Sci(1991)[2021155]; Tidsskr Nor Laegeforen(1991)[1957287]; Nihon Kyobu Shikkan Gakkai Zasshi(1991)[1770686]; J Protozool(1991)[1818223]; Rev Med Chil(1991)[9723082]; Med Clin (Barc)(1991)[1766280]; Int J STD AIDS(1992)[1616962]; J Thorac Imaging(1992)[1404545]; Rev Clin Esp(1992)[1475474]; Med Trop (Mars)(1992)[1494313]; Chest(1992)[1735305]; Dtsch Med Wochenschr(1992)[1425273]; Clin Infect Dis(1992)[1600018]; Enferm Infecc Microbiol Clin(1992)[1292602]; Nihon Kyobu Shikkan Gakkai Zasshi(1993)[8121088]; Nihon Kyobu Shikkan Gakkai Zasshi(1993)[8230897]; J Infect(1993)[8454891]; Aust N Z J Med(1993)[8141697]; Radiol Med(1993)[8386841]; Neurosurgery(1993)[8327080]; Thorax(1993)[8511742]; Nihon Kyobu Shikkan Gakkai Zasshi(1993)[8515594]; Radiographics(1993)[8356267]; J Clin Lab Anal(1993)[8389846]; Thorax(1993)[8434359]; Ann Intern Med(1993)[8480960]; Infection(1993)[8365820]; Pneumologie(1993)[8309925]; Semin Respir Infect(1993)[8278679]; J Thorac Imaging(1994)[8207784]; J Med Assoc Thai(1994)[7869019]; Clin Nephrol(1994)[8026116]; Ugeskr Laeger(1994)[7941090]; Am J Med(1994)[7985710]; Indian J Chest Dis Allied Sci(1994)[7737703]; Chest(1994)[8082342]; Radiographics(1995)[7761633]; Arch Bronconeumol(1995)[7627427]; Arch Otolaryngol Head Neck Surg(1995)[7546590]; J Clin Microbiol(1995)[8586730]; Clin Radiol(1995)[7489624]; Ann Dermatol Venereol(1995)[8687055]; Semin Thorac Cardiovasc Surg(1995)[7612761]; Clin Infect Dis(1995)[8527555]; Enferm Infecc Microbiol Clin(1995)[7779903]; Nihon Kyobu Shikkan Gakkai Zasshi(1995)[8821998]; Aust N Z J Med(1995)[7605295]; Am J Respir Crit Care Med(1995)[7767533]; Clin Infect Dis(1995)[7620032]; J Clin Microbiol(1995)[7650192]; Acta Cytol(1995)[7483983]; Gastroenterol Hepatol(1996)[8754417]; Zentralbl Bakteriol(1996)[8837378]; Clin Infect Dis(1996)[8909849]; Semin Respir Infect(1996)[8883177]; Enferm Infecc Microbiol Clin(1996)[8714157]; Clin Infect Dis(1996)[8824970]; J Med Assoc Thai(1996)[8855629]; Int J Antimicrob Agents(1996)[18611705]; Australas J Dermatol(1997)[9046650]; Eur J Med Res(1997)[9110925]; Clin Infect Dis(1997)[9142791]; Semin Perinatol(1997)[9298723]; Acta Cytol(1997)[9100786]; South Med J(1997)[9404918]; Rev Mal Respir(1997)[9480480]; Schweiz Med Wochenschr(1997)[9446191]; Praxis (Bern 1994)(1997)[9480518]; Gastroenterol Hepatol(1997)[9072199]; J Med Assoc Thai(1998)[9803090]; Kansenshogaku Zasshi(1998)[9916411]; Zentralbl Bakteriol(1998)[9638878]; Arch Inst Pasteur Alger(1998)[11256309]; J La State Med Soc(1998)[9926705]; Med Clin (Barc)(1998)[9922954]; Diagn Cytopathol(1999)[10352914]; Dermatology(1999)[10559593]; Chest(1999)[10084461]; J Formos Med Assoc(1999)[10560238]; Hosp Med(1999)[10621798]; Transpl Infect Dis(1999)[11428996]; J Thorac Imaging(1999)[9894953]; J Assoc Physicians India(1999)[10778661]; Ryoikibetsu Shokogun Shirizu(1999)[10201223]; Rev Iberoam Micol(1999)[18473558]; Clin Infect Dis(2000)[10770733]; Nihon Kokyuki Gakkai Zasshi(2000)[11244725]; Am J Respir Crit Care Med(2000)[10712349]; Bull Soc Pathol Exot(2000)[10774496]; Nihon Kokyuki Gakkai Zasshi(2000)[10879035]; Br J Dermatol(2000)[10971343]; Transplantation(2000)[10919584]; J Formos Med Assoc(2000)[10770026]; Sex Transm Infect(2000)[10858714]; Chest(2000)[10936151]; Ther Umsch(2001)[11695094]; Ther Umsch(2001)[11695093]; AIDS(2001)[11698718]; J Int Med Res(2001)[11393347]; J Endocrinol Invest(2001)[11765053]; Hunan Yi Ke Da Xue Xue Bao(2002)[12575241]; Infection(2002)[12382094]; Med Mycol(2002)[11860015]; J Korean Med Sci(2002)[11850600]; Br J Neurosurg(2003)[14756487]; Transpl Infect Dis(2003)[14617302]; Salud Publica Mex(2003)[14974289]; Zhonghua Yi Xue Za Zhi(2003)[12820917]; Postgrad Med(2003)[12545593]; Indian J Pathol Microbiol(2004)[16295487]; South Med J(2004)[15301128]; Med Mycol(2004)[15124872]; HIV Med(2004)[15236619]; Arq Bras Endocrinol Metabol(2004)[15640890]; Korean J Intern Med(2004)[15053045]; Eur J Clin Microbiol Infect Dis(2004)[15278730]; Respir Care(2004)[15165294]; Nihon Kokyuki Gakkai Zasshi(2004)[15069786]; Rev Iberoam Micol(2004)[15709789]; J Clin Microbiol(2004)[15297551]; Rev Iberoam Micol(2004)[15709792]; J Infect(2005)[16230188]; Zhonghua Jie He He Hu Xi Za Zhi(2005)[16115395]; Mycoses(2005)[15982204]; Nihon Kokyuki Gakkai Zasshi(2005)[15770944]; AJR Am J Roentgenol(2005)[16120909]; Mycoses(2005)[15679661]; Yonsei Med J(2005)[15744824]; QJM(2006)[16504989]; Chest(2006)[16478849]; Can Respir J(2006)[16896431]; J Trop Pediatr(2006)[16735366]; J Miss State Med Assoc(2006)[17941200]; J Pediatr Surg(2006)[16516607]; J Appl Res(2006)[19816551]; Respir Med(2006)[16239102]; Respiration(2007)[16864987]; Rev Inst Med Trop Sao Paulo(2007)[17823759]; Medicine (Baltimore)(2007)[17435588]; Med Mycol(2007)[17654275]; Int J Infect Dis(2008)[18054512]; Enferm Infecc Microbiol Clin(2008)[18208772]; Respirology(2008)[18339023]; Semin Respir Crit Care Med(2008)[18365996]; Clin Infect Dis(2008)[18171241]; Med Mycol(2008)[18654919]; Intern Med(2008)[19015612]; J Infect Chemother(2008)[18622672]; Rev Med Chir Soc Med Nat Iasi(2008)[18677910]; Cases J(2008)[19116029]; Lung India(2008)[21264083]; Transplant Proc(2008)[18455028]; Neurol Sci(2008)[18379738]; Rev Laryngol Otol Rhinol (Bord)(2009)[20597419]; Jpn J Infect Dis(2009)[19168962]; Arerugi(2009)[20168072]; J Microbiol Immunol Infect(2009)[19812855]; Paediatr Respir Rev(2009)[19879505]; New Microbiol(2009)[19579702]; Kyobu Geka(2009)[19764490]; Clin J Gastroenterol(2009)[26192799]; HIV Med(2009)[19207599]; Nihon Kokyuki Gakkai Zasshi(2009)[19198229]; Nihon Kokyuki Gakkai Zasshi(2009)[19637803]; Tidsskr Nor Laegeforen(2009)[19997137]; Clin Invest Med(2009)[19178882]; Int J Organ Transplant Med(2010)[25013584]; Int J Infect Dis(2010)[20932485]; Nihon Kokyuki Gakkai Zasshi(2010)[21226309]; Int J Infect Dis(2010)[19477671]; Am J Med Sci(2010)[20581653]; Epidemiol Infect(2010)[19796452]; Int J Ophthalmol(2010)[22553527]; J UOEH(2010)[20857817]; J Int Assoc Physicians AIDS Care (Chic)(2011)[21460353]; Scand J Infect Dis(2011)[21271944]; Semin Respir Crit Care Med(2011)[22167400]; Clin Infect Dis(2011)[21220771]; Respir Care(2011)[21255489]; Nihon Kokyuki Gakkai Zasshi(2011)[21591463]; Inflamm Bowel Dis(2011)[21674724]; Ultrastruct Pathol(2011)[21214404]; Med Health R I(2011)[21456379]; Clin Rheumatol(2012)[22476206]; BMJ Case Rep(2012)[23104630]; Einstein (Sao Paulo)(2012)[23386093]; Enferm Infecc Microbiol Clin(2012)[22197013]; Lung(2012)[22246551]; Singapore Med J(2012)[22337198]; Case Rep Pathol(2012)[22953139]; Rev Fac Cien Med Univ Nac Cordoba(2012)[22917073]; Zhonghua Lao Dong Wei Sheng Zhi Ye Bing Za Zhi(2012)[23257048]; Trop Doct(2012)[22290112]; BMC Infect Dis(2012)[22436174]; PLoS One(2013)[23613973]; Chest(2013)[23648912]; Int Urol Nephrol(2013)[22627787]; Indian J Med Microbiol(2013)[23508435]; Coll Antropol(2013)[23941014]; Pneumologie(2013)[24006197]; Dtsch Med Wochenschr(2013)[23860684]; Am J Dermatopathol(2013)[22892474]; J Clin Diagn Res(2013)[24298497]; Case Rep Pathol(2013)[23936710]; Int J Infect Dis(2013)[23777597]; J Thorac Dis(2013)[23991332]; Clin Radiol(2013)[23809268]; J Acquir Immune Defic Syndr(2013)[23542636]; BMC Res Notes(2013)[23510524]; J Venom Anim Toxins Incl Trop Dis(2014)[25180029]; J Assoc Physicians India(2014)[25327100]; Respir Med Case Rep(2014)[26029550]; Int J Clin Exp Pathol(2014)[25197354]; Can J Ophthalmol(2014)[25284106]; Intern Med(2014)[24930663]; Folia Microbiol (Praha)(2014)[24947767]; PLoS One(2014)[25360596]; PLoS One(2014)[24586423]; Rev Pneumol Clin(2014)[24210157]; Int J Infect Dis(2014)[24589681]; Rev Iberoam Micol(2014)[23462234]; Mycopathologia(2015)[25563716]; Lancet Infect Dis(2015)[25467646]; S Afr Med J(2015)[26636155]; J Family Med Prim Care(2015)[26985424]; Transpl Infect Dis(2015)[25689604]; Ned Tijdschr Geneeskd(2015)[25827149]; Infect Drug Resist(2015)[25999744]; Am J Orthop (Belle Mead NJ)(2015)[26665255]; Mycoses(2015)[26444438]; Mikrobiyol Bul(2016)[27525403]; Rinsho Shinkeigaku(2016)[26616484]; Zhongguo Dang Dai Er Ke Za Zhi(2016)[27530787]; Med Mycol(2016)[27118805]; Rev Med Inst Mex Seguro Soc(2016)[27428339]; Open Forum Infect Dis(2016)[27704021]; Transplant Proc(2016)[27742281]; J Rheumatol(2016)[27307524]; Medicine (Baltimore)(2016)[27583871]; Clin Immunol(2016)[26732859]; Intern Med(2016)[27086819]; BMC Infect Dis(2017)[28558705]; Mycoses(2017)[28857298]; Clin Chest Med(2017)[28797489]; Clin Chest Med(2017)[28797488]; Indian J Ophthalmol(2017)[28643722]; Medicine (Baltimore)(2017)[28700464]; BJR Case Rep(2017)[30363287]; J La State Med Soc(2017)[28414687]; BMJ Case Rep(2017)[28188169]; Exp Clin Transplant(2018)[27143150]; Mycopathologia(2018)[29086143]; Medicine (Baltimore)(2018)[30278580]; Transpl Infect Dis(2018)[29359837]; Clin Lab(2018)[30549981]; Respir Med Case Rep(2018)[29619316]; Respir Med Case Rep(2018)[30302308]; Infect Drug Resist(2018)[30555247]; Case Reports Hepatol(2018)[30693118]; Exp Ther Med(2018)[30542394]; Eur J Case Rep Intern Med(2018)[30756009]; Respirol Case Rep(2018)[29796274]; BMC Nephrol(2018)[29374464]; Rev Iberoam Micol(2019)[31676212]; Laryngoscope(2019)[30152047]; BMC Infect Dis(2019)[31405376]; J Infect Chemother(2019)[31182330]; CEN Case Rep(2019)[31161376]; Medicine (Baltimore)(2019)[31083210]; Klin Mikrobiol Infekc Lek(2019)[31904103]; Future Microbiol(2019)[31992070]; Eur J Clin Microbiol Infect Dis(2019)[30684164]; Intern Med J(2019)[31713345]; Med Mycol(2019)[30329097]; Cureus(2019)[31316875]; World J Clin Cases(2019)[31799309]; Leuk Lymphoma(2019)[30188226]; Cureus(2019)[32025418]; Rev Iberoam Micol(2019)[31537469]; Case Rep Infect Dis(2019)[30915245]; HIV Med(2019)[30311440]; Afr J Thorac Crit Care Med(2019)[34286267]; Ann Pharmacother(2019)[31014083]; BMC Geriatr(2019)[30909914]; Am J Med(2019)[31077652]; Case Rep Infect Dis(2019)[31032130]; Infez Med(2019)[31205044]; Medicine (Baltimore)(2019)[31305427]; Rev Inst Med Trop Sao Paulo(2020)[32491141]; Transplant Proc(2020)[32641222]; Front Endocrinol (Lausanne)(2020)[33391186]; J Infect Chemother(2020)[32747210]; Exp Clin Transplant(2020)[30696395]; J Int Med Res(2020)[32070156]; Acta Trop(2020)[31580849]; J Neurovirol(2020)[31863401]; J Pediatric Infect Dis Soc(2020)[30339241]; BMC Infect Dis(2020)[32000709]; J Diabetes Investig(2020)[31758642]; World J Hepatol(2020)[32547692]; Infect Dis Health(2020)[31501070]; BMJ Case Rep(2020)[32295799]; BMJ Case Rep(2020)[31937629]; Germs(2020)[33489955]; Open Forum Infect Dis(2020)[33324722]; J Int Med Res(2020)[32527201]; Case Rep Infect Dis(2020)[32351746]; Can J Infect Dis Med Microbiol(2020)[33144899]; Ann Palliat Med(2020)[32434369]; Aging Male(2020)[31741419]; J Thorac Dis(2020)[32274153]; Chin Med J (Engl)(2020)[33273326]; Med Mal Infect(2020)[32777360]; Case Rep Infect Dis(2020)[32774953]; BMC Pulm Med(2020)[32503511]; IDCases(2020)[32055440]; Ann Otol Rhinol Laryngol(2021)[32627566]; Clin Nucl Med(2021)[33315665]; Clin Infect Dis(2021)[33772538]; J Nippon Med Sch(2021)[34471065]; BMJ Case Rep(2021)[34108156]; Medicine (Baltimore)(2021)[34260570]; Intern Med(2021)[34053982]; Med Mycol(2021)[34374784]; Exp Clin Transplant(2021)[29957160]; Transpl Infect Dis(2021)[33040432]; Mycopathologia(2021)[34181160]; Clin Case Rep(2021)[34306681]; Ther Adv Infect Dis(2021)[33996076]; Cureus(2021)[34178506]; Case Rep Dermatol(2021)[33708089]; Case Rep Med(2021)[33628263]; Open Med (Wars)(2021)[33681469]; Anaesthesist(2022)[34427689] |
| *Cytomegalovirus (CMV)* | Established pathogen |  | **established** |  |
| *Enterobacter asburiae* | Unlikely patho, no or very few evidence | 1 | **established** | J Med Virol(2022)[34812530] |
| *Enterobacter cloacae* | Established pathogen | 130 | **established** | Chest(1975)[1149508]; J Antimicrob Chemother(1981)[19803006]; Pathol Biol (Paris)(1982)[6214758]; Jpn J Antibiot(1983)[6674523]; Arch Intern Med(1985)[4004426]; Rev Invest Clin(1985)[4095402]; Drugs(1985)[3896741]; Am Surg(1986)[3729174]; Intensive Care Med(1987)[3655101]; South Med J(1988)[3406788]; No To Shinkei(1988)[3293638]; J Clin Microbiol(1989)[2681247]; Zhonghua Min Guo Xiao Er Ke Yi Xue Hui Za Zhi(1989)[2637614]; Wiad Lek(1990)[2275197]; Pneumologie(1990)[2367404]; Med Klin (Munich)(1990)[2377146]; Kansenshogaku Zasshi(1990)[2243194]; Rev Infect Dis(1991)[1925270]; Clin Pharm(1991)[1999086]; J Hosp Infect(1992)[1348068]; Ann Rheum Dis(1992)[1632668]; J Heart Lung Transplant(1992)[1610862]; Rev Pneumol Clin(1993)[8296141]; Chest(1993)[8222797]; Clin Infect Dis(1993)[8399893]; Acta Paediatr(1994)[7819699]; Clin Investig(1994)[7711407]; Transplantation(1995)[7570975]; Kansenshogaku Zasshi(1995)[8708405]; Anasthesiol Intensivmed Notfallmed Schmerzther(1997)[9498890]; Zhonghua Jie He He Hu Xi Za Zhi(1997)[10072803]; Clin Infect Dis(1997)[9332532]; Nihon Kokyuki Gakkai Zasshi(1998)[9656677]; Eur J Clin Microbiol Infect Dis(1998)[9758274]; Eur J Pediatr(1998)[9809826]; J Perinatol(1998)[9730200]; Kansenshogaku Zasshi(1998)[9545687]; Jpn J Antibiot(1999)[10695024]; Transplantation(1999)[10232564]; J Microbiol Immunol Infect(2000)[10917881]; Ann Fr Anesth Reanim(2000)[10976375]; Crit Care Med(2000)[10667501]; Can J Infect Dis(2000)[18159274]; J Microbiol Immunol Infect(2001)[11456359]; Eur J Clin Microbiol Infect Dis(2001)[11837636]; Pediatrics(2002)[12205248]; Clin Infect Dis(2002)[12173126]; Am J Infect Control(2004)[15175611]; Diagn Microbiol Infect Dis(2004)[15023432]; Di Yi Jun Yi Da Xue Xue Bao(2004)[15604082]; Int J Antimicrob Agents(2005)[16280243]; J Microbiol Immunol Infect(2006)[16440126]; J Chemother(2006)[17267336]; Zhongguo Dang Dai Er Ke Za Zhi(2006)[17052391]; J Antimicrob Chemother(2007)[17540673]; Pharmacotherapy(2007)[17253909]; Mali Med(2007)[19437827]; Med Mal Infect(2008)[18180124]; Microb Drug Resist(2008)[18321204]; Antimicrob Agents Chemother(2008)[18285482]; Zhonghua Er Ke Za Zhi(2008)[19099852]; Pediatr Blood Cancer(2008)[18240170]; Zhonghua Er Ke Za Zhi(2008)[19099875]; Respirology(2009)[19818053]; Zentralbl Chir(2009)[19382051]; Clin Ther(2009)[19302902]; Anestezjol Intens Ter(2009)[20201347]; Med Mal Infect(2010)[20172671]; Eur J Pediatr(2010)[20119725]; J Perinat Med(2010)[20297900]; Bull Soc Pathol Exot(2011)[21103965]; J Infect Chemother(2011)[21847518]; Clin Microbiol Infect(2011)[20345467]; Antimicrob Agents Chemother(2012)[22203599]; Zhongguo Dang Dai Er Ke Za Zhi(2012)[23234773]; Arch Bronconeumol(2012)[22858303]; Eur Spine J(2013)[23543389]; Pediatr Infect Dis J(2013)[23411624]; Sichuan Da Xue Xue Bao Yi Xue Ban(2013)[24059113]; Lung(2013)[23564195]; Diagn Microbiol Infect Dis(2014)[24268534]; Pulm Pharmacol Ther(2014)[24704389]; Medwave(2014)[25383685]; Zhonghua Liu Xing Bing Xue Za Zhi(2014)[25598264]; Microb Drug Resist(2014)[24716493]; Enferm Infecc Microbiol Clin(2014)[23587705]; PLoS One(2015)[26407326]; Turk J Med Sci(2015)[26775390]; Infect Chemother(2015)[26157593]; Transpl Infect Dis(2015)[25572932]; J Microbiol Immunol Infect(2015)[24239065]; Pneumonol Alergol Pol(2015)[25754051]; Khirurgiia (Mosk)(2015)[26271423]; Arch Pediatr(2015)[25466784]; Rev Med Inst Mex Seguro Soc(2016)[27197104]; Enferm Infecc Microbiol Clin(2016)[26139302]; Invest Clin(2016)[29938989]; Indian J Radiol Imaging(2016)[28104952]; Zhonghua Wai Ke Za Zhi(2016)[27373479]; Balkan Med J(2016)[27994915]; J Med Microbiol(2017)[27911257]; Pak J Pharm Sci(2017)[29175778]; Int J Environ Res Public Health(2017)[29149019]; Med Sci Monit(2017)[28135233]; Semergen(2017)[27773623]; Int J Antimicrob Agents(2018)[29277527]; Zhonghua Er Ke Za Zhi(2018)[29342994]; Antimicrob Agents Chemother(2018)[29203488]; Intractable Rare Dis Res(2018)[30560023]; Ann Clin Microbiol Antimicrob(2018)[29571291]; Monaldi Arch Chest Dis(2018)[29741076]; Antimicrob Resist Infect Control(2019)[31548884]; Antibiotics (Basel)(2019)[31766123]; Antimicrob Resist Infect Control(2019)[30976388]; Rev Esp Quimioter(2020)[32149487]; Int J Antimicrob Agents(2020)[32987104]; Zhonghua Xue Ye Xue Za Zhi(2020)[33445846]; Clin Microbiol Infect(2020)[32603803]; J Infect Public Health(2020)[32439355]; Endosc Int Open(2020)[33269309]; J Clin Microbiol(2020)[32075901]; J Med Microbiol(2021)[33587030]; Acta Med Indones(2021)[34251345]; J Antimicrob Chemother(2021)[33202023]; Zhonghua Wei Zhong Bing Ji Jiu Yi Xue(2021)[34053483]; PLoS One(2021)[33730101]; Infect Control Hosp Epidemiol(2021)[32703320]; ASAIO J(2022)[34264871]; Infect Control Hosp Epidemiol(2022)[33975668]; Infect Dis (Lond)(2022)[34382910] |
| *Enterobacter cloacae* complex | Established pathogen | 130 | **established** | Chest(1975)[1149508]; J Antimicrob Chemother(1981)[19803006]; Pathol Biol (Paris)(1982)[6214758]; Jpn J Antibiot(1983)[6674523]; Arch Intern Med(1985)[4004426]; Rev Invest Clin(1985)[4095402]; Drugs(1985)[3896741]; Am Surg(1986)[3729174]; Intensive Care Med(1987)[3655101]; South Med J(1988)[3406788]; No To Shinkei(1988)[3293638]; J Clin Microbiol(1989)[2681247]; Zhonghua Min Guo Xiao Er Ke Yi Xue Hui Za Zhi(1989)[2637614]; Wiad Lek(1990)[2275197]; Pneumologie(1990)[2367404]; Med Klin (Munich)(1990)[2377146]; Kansenshogaku Zasshi(1990)[2243194]; Rev Infect Dis(1991)[1925270]; Clin Pharm(1991)[1999086]; J Hosp Infect(1992)[1348068]; Ann Rheum Dis(1992)[1632668]; J Heart Lung Transplant(1992)[1610862]; Rev Pneumol Clin(1993)[8296141]; Chest(1993)[8222797]; Clin Infect Dis(1993)[8399893]; Acta Paediatr(1994)[7819699]; Clin Investig(1994)[7711407]; Transplantation(1995)[7570975]; Kansenshogaku Zasshi(1995)[8708405]; Anasthesiol Intensivmed Notfallmed Schmerzther(1997)[9498890]; Zhonghua Jie He He Hu Xi Za Zhi(1997)[10072803]; Clin Infect Dis(1997)[9332532]; Nihon Kokyuki Gakkai Zasshi(1998)[9656677]; Eur J Clin Microbiol Infect Dis(1998)[9758274]; Eur J Pediatr(1998)[9809826]; J Perinatol(1998)[9730200]; Kansenshogaku Zasshi(1998)[9545687]; Jpn J Antibiot(1999)[10695024]; Transplantation(1999)[10232564]; J Microbiol Immunol Infect(2000)[10917881]; Ann Fr Anesth Reanim(2000)[10976375]; Crit Care Med(2000)[10667501]; Can J Infect Dis(2000)[18159274]; J Microbiol Immunol Infect(2001)[11456359]; Eur J Clin Microbiol Infect Dis(2001)[11837636]; Pediatrics(2002)[12205248]; Clin Infect Dis(2002)[12173126]; Am J Infect Control(2004)[15175611]; Diagn Microbiol Infect Dis(2004)[15023432]; Di Yi Jun Yi Da Xue Xue Bao(2004)[15604082]; Int J Antimicrob Agents(2005)[16280243]; J Microbiol Immunol Infect(2006)[16440126]; J Chemother(2006)[17267336]; Zhongguo Dang Dai Er Ke Za Zhi(2006)[17052391]; J Antimicrob Chemother(2007)[17540673]; Pharmacotherapy(2007)[17253909]; Mali Med(2007)[19437827]; Med Mal Infect(2008)[18180124]; Microb Drug Resist(2008)[18321204]; Antimicrob Agents Chemother(2008)[18285482]; Zhonghua Er Ke Za Zhi(2008)[19099852]; Pediatr Blood Cancer(2008)[18240170]; Zhonghua Er Ke Za Zhi(2008)[19099875]; Respirology(2009)[19818053]; Zentralbl Chir(2009)[19382051]; Clin Ther(2009)[19302902]; Anestezjol Intens Ter(2009)[20201347]; Med Mal Infect(2010)[20172671]; Eur J Pediatr(2010)[20119725]; J Perinat Med(2010)[20297900]; Bull Soc Pathol Exot(2011)[21103965]; J Infect Chemother(2011)[21847518]; Clin Microbiol Infect(2011)[20345467]; Antimicrob Agents Chemother(2012)[22203599]; Zhongguo Dang Dai Er Ke Za Zhi(2012)[23234773]; Arch Bronconeumol(2012)[22858303]; Eur Spine J(2013)[23543389]; Pediatr Infect Dis J(2013)[23411624]; Sichuan Da Xue Xue Bao Yi Xue Ban(2013)[24059113]; Lung(2013)[23564195]; Diagn Microbiol Infect Dis(2014)[24268534]; Pulm Pharmacol Ther(2014)[24704389]; Medwave(2014)[25383685]; Zhonghua Liu Xing Bing Xue Za Zhi(2014)[25598264]; Microb Drug Resist(2014)[24716493]; Enferm Infecc Microbiol Clin(2014)[23587705]; PLoS One(2015)[26407326]; Turk J Med Sci(2015)[26775390]; Infect Chemother(2015)[26157593]; Transpl Infect Dis(2015)[25572932]; J Microbiol Immunol Infect(2015)[24239065]; Pneumonol Alergol Pol(2015)[25754051]; Khirurgiia (Mosk)(2015)[26271423]; Arch Pediatr(2015)[25466784]; Rev Med Inst Mex Seguro Soc(2016)[27197104]; Enferm Infecc Microbiol Clin(2016)[26139302]; Invest Clin(2016)[29938989]; Indian J Radiol Imaging(2016)[28104952]; Zhonghua Wai Ke Za Zhi(2016)[27373479]; Balkan Med J(2016)[27994915]; J Med Microbiol(2017)[27911257]; Pak J Pharm Sci(2017)[29175778]; Int J Environ Res Public Health(2017)[29149019]; Med Sci Monit(2017)[28135233]; Semergen(2017)[27773623]; Int J Antimicrob Agents(2018)[29277527]; Zhonghua Er Ke Za Zhi(2018)[29342994]; Antimicrob Agents Chemother(2018)[29203488]; Intractable Rare Dis Res(2018)[30560023]; Ann Clin Microbiol Antimicrob(2018)[29571291]; Monaldi Arch Chest Dis(2018)[29741076]; Antimicrob Resist Infect Control(2019)[31548884]; Antibiotics (Basel)(2019)[31766123]; Antimicrob Resist Infect Control(2019)[30976388]; Rev Esp Quimioter(2020)[32149487]; Int J Antimicrob Agents(2020)[32987104]; Zhonghua Xue Ye Xue Za Zhi(2020)[33445846]; Clin Microbiol Infect(2020)[32603803]; J Infect Public Health(2020)[32439355]; Endosc Int Open(2020)[33269309]; J Clin Microbiol(2020)[32075901]; J Med Microbiol(2021)[33587030]; Acta Med Indones(2021)[34251345]; J Antimicrob Chemother(2021)[33202023]; Zhonghua Wei Zhong Bing Ji Jiu Yi Xue(2021)[34053483]; PLoS One(2021)[33730101]; Infect Control Hosp Epidemiol(2021)[32703320]; ASAIO J(2022)[34264871]; Infect Control Hosp Epidemiol(2022)[33975668]; Infect Dis (Lond)(2022)[34382910] |
| *Enterobacter cloacae complex sp. ECNIH7* | Unlikely patho, no or very few evidence | 0 | **established** | NA |
| *Enterobacter kobei* | Unlikely patho, no or very few evidence | 1 | **established** | Pneumonol Alergol Pol(2015)[25754051] |
| *Enterobacter ludwigii* | Unlikely patho, no or very few evidence | 0 | **established** | NA |
| *Enterobacter roggenkampii* | Unlikely patho, no or very few evidence | 0 | **established** | NA |
| *Enterobacter sp. E20* | Unlikely patho, no or very few evidence | 0 | **established** | NA |
| *Enterobacter sp. HK169* | Unlikely patho, no or very few evidence | 0 | **established** | NA |
| *Enterobacter sp. ODB01* | Unlikely patho, no or very few evidence | 0 | **established** | NA |
| *Enterococcus faecalis* | Established pathogen | 83 | **established** | Antibiotiki(1969)[4391893]; Am J Dis Child(1979)[106722]; Pneumonol Pol(1979)[119214]; Am J Obstet Gynecol(1981)[6787924]; Jpn J Antibiot(1987)[3586339]; Jpn J Antibiot(1991)[1652653]; Enferm Infecc Microbiol Clin(1991)[1668361]; Kansenshogaku Zasshi(1991)[2071954]; Kansenshogaku Zasshi(1991)[1761891]; Clin Infect Dis(1992)[1617072]; Pediatriia(1992)[1475147]; J Assoc Physicians India(1992)[1634456]; N Engl J Med(1993)[8417392]; J Pediatr(1994)[8071746]; Enferm Infecc Microbiol Clin(1995)[7779904]; Clin Infect Dis(1995)[7742433]; Kansenshogaku Zasshi(1995)[8708405]; J Formos Med Assoc(1996)[9000815]; South Med J(1999)[10414486]; Arch Bronconeumol(2001)[11674943]; Am J Kidney Dis(2001)[11576907]; Actas Urol Esp(2001)[11692799]; Arch Esp Urol(2002)[12455285]; Arch Bronconeumol(2002)[12113748]; Chest(2003)[12853512]; An Med Interna(2004)[15283647]; Recenti Prog Med(2004)[15032335]; Kansenshogaku Zasshi(2004)[15678979]; Saudi Med J(2005)[16228055]; Clin Microbiol Infect(2005)[15649302]; J Chemother(2006)[17267336]; Am J Emerg Med(2007)[17276814]; Chest(2007)[17317736]; Med Intensiva(2007)[17306135]; Ann Thorac Surg(2007)[17954082]; Int J Antimicrob Agents(2008)[18160263]; G Ital Nefrol(2008)[19048577]; Pediatr Blood Cancer(2008)[18240170]; J Hosp Infect(2008)[18799242]; Medicine (Baltimore)(2009)[19282702]; Diagn Microbiol Infect Dis(2009)[19913683]; Respirology(2009)[19818053]; Int J Infect Dis(2009)[18775663]; Spine (Phila Pa 1976)(2009)[19139655]; Ugeskr Laeger(2010)[20696120]; Nihon Ronen Igakkai Zasshi(2012)[22466781]; Roum Arch Microbiol Immunol(2012)[23210322]; Clin Infect Dis(2012)[22700828]; Rev Soc Bras Med Trop(2012)[22767099]; PLoS Negl Trop Dis(2012)[22363829]; Infect Control Hosp Epidemiol(2012)[22961018]; Ann Fr Anesth Reanim(2013)[24211001]; Zhonghua Wai Ke Za Zhi(2013)[24256585]; Case Rep Med(2014)[24872819]; Transplant Proc(2014)[25420856]; J Infect Dev Ctries(2015)[26623628]; Pneumonol Alergol Pol(2015)[25754051]; Zhonghua Wai Ke Za Zhi(2016)[27373479]; Infez Med(2017)[28603227]; Turk Pediatri Ars(2017)[29062249]; Infect Control Hosp Epidemiol(2018)[29249216]; Infect Control Hosp Epidemiol(2018)[29486805]; Pan Afr Med J(2018)[29875941]; Medicine (Baltimore)(2019)[31096489]; Trop Med Infect Dis(2019)[30759812]; New Microbes New Infect(2019)[31763048]; Zhonghua Shao Shang Za Zhi(2019)[31154732]; Infect Control Hosp Epidemiol(2020)[31762428]; Einstein (Sao Paulo)(2020)[32725054]; Zhonghua Yu Fang Yi Xue Za Zhi(2020)[32842309]; Trop Med Infect Dis(2020)[32178241]; Zhonghua Yi Xue Za Zhi(2020)[33379842]; IDCases(2020)[32566481]; Infect Prev Pract(2020)[34316565]; Exp Ther Med(2020)[33101483]; Clin Microbiol Infect(2021)[34242804]; BMJ Case Rep(2021)[34479880]; Biomed Res Int(2021)[34136573]; Antimicrob Resist Infect Control(2021)[33407856]; BMJ Case Rep(2021)[34326118]; Surg Case Rep(2021)[34061274]; J Infect Dev Ctries(2021)[33690209]; Mil Med(2021)[33499465] |
| *Enterococcus faecium* | Established pathogen | 56 | **established** | Kansenshogaku Zasshi(1991)[1761891]; Pediatriia(1992)[1475147]; Med Clin (Barc)(1994)[8170232]; J Pediatr(1994)[8071746]; Clin Infect Dis(1995)[7742433]; Arch Intern Med(1996)[8678715]; Am J Respir Crit Care Med(2000)[10806182]; Am J Kidney Dis(2001)[11576907]; Crit Care Med(2001)[11801846]; Kansenshogaku Zasshi(2002)[12607351]; Mikrobiyol Bul(2005)[16358496]; J Chemother(2006)[17267336]; Endoscopy(2007)[17957611]; Transpl Int(2007)[17291223]; Infect Control Hosp Epidemiol(2008)[18947320]; Diagn Microbiol Infect Dis(2009)[19913683]; Kansenshogaku Zasshi(2011)[21861443]; Diagn Microbiol Infect Dis(2011)[21251573]; Chest(2011)[21659436]; Australas Med J(2012)[23289047]; W V Med J(2012)[25134190]; Chemotherapy(2012)[23548324]; ScientificWorldJournal(2012)[22619600]; Clin Infect Dis(2012)[22700828]; Anaesthesiol Intensive Ther(2012)[23801507]; Zhonghua Wai Ke Za Zhi(2013)[24256585]; Zhonghua Nei Ke Za Zhi(2013)[23856111]; Ann Nucl Med(2014)[24481822]; Pan Afr Med J(2014)[25018799]; J Clin Diagn Res(2014)[25584223]; Case Rep Infect Dis(2015)[26605096]; Transplant Proc(2015)[26518949]; Presse Med(2016)[26874909]; Zhonghua Wai Ke Za Zhi(2016)[27916031]; Case Rep Cardiol(2017)[28316844]; Infect Control Hosp Epidemiol(2018)[29486805]; Cureus(2018)[30410833]; J Infect Public Health(2018)[28780309]; Acute Crit Care(2018)[31723866]; Int J Gen Med(2018)[29317845]; JMM Case Rep(2018)[30425838]; Antimicrob Resist Infect Control(2018)[30455867]; Open Forum Infect Dis(2019)[30838226]; Crit Care Med(2019)[30407949]; BMC Infect Dis(2020)[32087689]; Antimicrob Resist Infect Control(2020)[32962759]; J Clin Pharm Ther(2020)[31657870]; Acute Crit Care(2020)[32907310]; Zhonghua Yi Xue Za Zhi(2020)[33379842]; Eur J Clin Microbiol Infect Dis(2021)[32909085]; J Infect Dev Ctries(2021)[33690209]; Mil Med(2021)[33499465]; Int J Environ Res Public Health(2021)[33923992]; Antimicrob Resist Infect Control(2021)[33407856]; Respir Med Case Rep(2021)[33732614]; Clin Infect Dis(2021)[32822465] |
| *Escherichia albertii* | Unlikely patho, no or very few evidence | 0 | **established** | NA |
| *Escherichia coli* | Established pathogen | 1082 | **established** | Beitr Klin Tuberk Spezif Tuberkuloseforsch(1950)[14777749]; Montp Med(1953)[13119723]; Athena(1953)[13058795]; Medizinische(1953)[13071212]; Sven Lakartidn(1956)[13311778]; Acta Microbiol Acad Sci Hung(1957)[13424151]; Antibiotiki(1962)[13911728]; Langenbecks Arch Klin Chir Ver Dtsch Z Chir(1963)[14120185]; Vopr Okhr Materin Det(1963)[14074898]; Gig Tr Prof Zabol(1963)[14109780]; Antibiotiki(1963)[14124734]; Acta Chir Scand(1964)[14169778]; Union Med Can(1964)[14146629]; Acta Chir Scand(1964)[14253872]; Zh Mikrobiol Epidemiol Immunobiol(1964)[14295807]; Z Arztl Fortbild (Jena)(1964)[14240423]; Acta Chir Scand(1964)[14171727]; Maandschr Kindergeneeskd(1964)[14224082]; Boll Ist Sieroter Milan(1964)[14180470]; Gac Med Caracas(1964)[14184336]; Monatsschr Kinderheilkd (1902)(1965)[5326766]; J Hyg (Lond)(1965)[14308350]; Monatsschr Kinderheilkd (1902)(1965)[14266615]; Laryngoscope(1965)[14256354]; Ann Intern Med(1965)[14258349]; Antimicrob Agents Chemother (Bethesda)(1966)[4862164]; G Mal Infett Parassit(1966)[4873823]; Med Klin(1966)[4385142]; J Sci Med Lille(1966)[5328538]; Orv Hetil(1967)[4872094]; J Tenn Med Assoc(1967)[4861825]; Policlinico Prat(1967)[4879265]; Antibiotiki(1967)[4386255]; N Engl J Med(1967)[4861559]; Ter Arkh(1967)[4888255]; Pol Tyg Lek(1967)[4863355]; Srp Arh Celok Lek(1967)[4883809]; N S Med Bull(1968)[4880900]; Postgrad Med(1968)[4869879]; Monatsschr Kinderheilkd (1902)(1968)[4887801]; Mich Med(1968)[4865842]; Arch Fr Pediatr(1968)[4880390]; Rev Med Chir Soc Med Nat Iasi(1968)[4978391]; Sem Hop(1968)[4310571]; G Ital Chemioter(1969)[4935435]; J Infect Dis(1969)[4977998]; Rev Hosp Clin Fac Med Sao Paulo(1969)[4903579]; Am Surg(1969)[4893662]; Rev Hosp Clin Fac Med Sao Paulo(1969)[4981764]; G Ital Chemioter(1969)[4935431]; Am J Roentgenol Radium Ther Nucl Med(1969)[4898693]; Scott Med J(1969)[4980002]; Antibiotiki(1969)[4391893]; Minerva Pediatr(1969)[4929967]; Pediatria (Bucur)(1969)[4907855]; Arkh Patol(1969)[4308115]; JAMA(1970)[4910819]; Thorax(1970)[4907196]; Pol Tyg Lek(1970)[4923892]; Am J Med Sci(1970)[4990227]; Bull Fed Soc Gynecol Obstet Lang Fr(1970)[4916591]; Pediatriia(1971)[4397850]; G Mal Infett Parassit(1971)[4948667]; Lijec Vjesn(1971)[4949831]; Scand J Infect Dis(1971)[4943331]; Ann Intern Med(1971)[4944157]; Medicine (Baltimore)(1971)[4944120]; Acta Microbiol Acad Sci Hung(1971)[5006094]; J Infect Dis(1971)[4947220]; Zh Mikrobiol Epidemiol Immunobiol(1972)[4569261]; Pathol Eur(1972)[4559956]; Ann Intern Med(1972)[4628214]; Dtsch Med Wochenschr(1972)[4553767]; Med Interna (Bucur)(1973)[4602843]; Am Rev Respir Dis(1973)[4201016]; Radiology(1973)[4572080]; J Infect Dis(1973)[4577976]; Ann Thorac Surg(1973)[4575244]; Vrach Delo(1974)[4155551]; Acta Paediatr Acad Sci Hung(1974)[4619871]; Wiad Lek(1974)[4602730]; Jpn J Antibiot(1974)[4612188]; Am J Med Sci(1975)[1211410]; J Urol(1975)[808645]; Chest(1975)[1149508]; Minerva Pediatr(1975)[1095907]; Nurs Times(1975)[1096088]; Z Erkr Atmungsorgane(1975)[775815]; Med Pediatr Oncol(1975)[778573]; J Am Geriatr Soc(1975)[1117117]; Klin Padiatr(1975)[1102765]; Rev Pediatr Obstet Ginecol Pediatr(1976)[822493]; N Y State J Med(1976)[768836]; AJR Am J Roentgenol(1976)[793427]; Lancet(1976)[62923]; Scott Med J(1976)[1265474]; J Infect Dis(1977)[850096]; S Afr Med J(1977)[416503]; Am J Med(1977)[871128]; J Fam Pract(1977)[320285]; Urology(1978)[343345]; Rev Chil Pediatr(1978)[390651]; Blood(1978)[416862]; Zentralbl Chir(1978)[358672]; Arch Dis Child(1979)[475415]; Am J Dis Child(1979)[382838]; Rev Prat(1979)[379969]; MMW Munch Med Wochenschr(1979)[33333]; J Clin Pathol(1979)[381332]; Khirurgiia (Mosk)(1979)[109696]; Minerva Med(1979)[492551]; Pneumonol Pol(1979)[119951]; J Pediatr(1979)[758385]; Grudn Khir(1980)[6768644]; Med Clin North Am(1980)[6993805]; MMW Munch Med Wochenschr(1980)[6771633]; Scand J Infect Dis Suppl(1980)[7010535]; Pediatrie(1980)[7008016]; Pediatrics(1980)[6990374]; J Am Geriatr Soc(1980)[6993540]; Vrach Delo(1980)[6998127]; Arch Intern Med(1980)[6986130]; Monatsschr Kinderheilkd(1981)[7335093]; Monatsschr Kinderheilkd(1981)[7035876]; Aust N Z J Med(1981)[7036972]; Am J Med(1981)[7211896]; Grudn Khir(1981)[6783478]; Surgery(1981)[7025316]; MMW Munch Med Wochenschr(1981)[6785603]; Compr Ther(1981)[7018827]; Cancer(1981)[7226035]; Chest(1981)[7226908]; Klin Med (Mosk)(1981)[7017264]; N Y State J Med(1981)[7019776]; Pathol Biol (Paris)(1982)[6214758]; Rev Infect Dis(1982)[6760339]; Am Rev Respir Dis(1982)[6756235]; Sov Med(1982)[6750804]; Bol Med Hosp Infant Mex(1982)[6280735]; Anaesthesist(1982)[7091639]; Jpn J Antibiot(1982)[6306293]; Am J Med(1982)[7046433]; Ann Fr Anesth Reanim(1982)[6224444]; J Chronic Dis(1982)[7119080]; Jpn J Antibiot(1983)[6348341]; Jpn J Antibiot(1983)[6425535]; Am J Infect Control(1983)[6349427]; Pol Tyg Lek(1983)[6346293]; Infect Control(1983)[6354957]; Rev Clin Esp(1983)[6344156]; Rev Invest Clin(1983)[6346449]; Jpn J Antibiot(1983)[6348340]; Internist (Berl)(1983)[6360946]; J Antimicrob Chemother(1983)[6352615]; Eur J Respir Dis(1983)[6825749]; J Natl Med Assoc(1983)[6827607]; Gan To Kagaku Ryoho(1983)[6639099]; Dtsch Med Wochenschr(1983)[6360617]; Heart Lung(1984)[6381413]; Med Clin (Barc)(1984)[6363833]; Am J Dis Child(1984)[6372439]; J Infect(1984)[6501903]; Infect Control(1984)[6376400]; Arch Intern Med(1984)[6380441]; Am J Dis Child(1984)[6695867]; Lancet(1984)[6140352]; J Trauma(1984)[6481828]; Pediatr Pol(1984)[6387612]; Med Dosw Mikrobiol(1984)[6387329]; Jpn J Antibiot(1985)[3937915]; Pediatr Pol(1985)[3913893]; Medicine (Baltimore)(1985)[4033411]; Isr J Med Sci(1985)[3000984]; Am J Med(1985)[3859208]; Am J Med(1985)[3881947]; Am J Med(1985)[4025369]; Gan To Kagaku Ryoho(1985)[4004291]; Pathol Biol (Paris)(1985)[3897966]; J Infect(1985)[3891869]; Drugs(1986)[3488201]; Am J Epidemiol(1986)[3940431]; Infection(1986)[3759253]; Immun Infekt(1986)[3100428]; J Hyg Epidemiol Microbiol Immunol(1986)[3522729]; Southeast Asian J Trop Med Public Health(1986)[3738607]; J Trop Med Hyg(1986)[3773019]; Rev Clin Esp(1986)[3532231]; West J Med(1986)[3953085]; Med Pediatr Oncol(1986)[3023801]; Am Rev Respir Dis(1987)[3310768]; J Fam Pract(1987)[3546587]; Zh Mikrobiol Epidemiol Immunobiol(1987)[3296578]; Infection(1987)[3312030]; Med Clin (Barc)(1987)[3329276]; Pediatr Pulmonol(1987)[3501100]; Ann Trop Paediatr(1987)[2445266]; Tijdschr Gerontol Geriatr(1988)[3282344]; Lancet(1988)[2896879]; Kansenshogaku Zasshi(1988)[3138338]; Rev Infect Dis(1988)[3353630]; Am J Med(1988)[3400692]; Pediatrie(1989)[2797997]; Jpn J Antibiot(1989)[2695657]; Ann Pediatr (Paris)(1989)[2648938]; J Clin Lab Immunol(1989)[2534928]; Rev Argent Microbiol(1989)[2748851]; An Med Interna(1989)[2491494]; Arch Phys Med Rehabil(1989)[2647058]; Clin Pediatr (Phila)(1989)[2805557]; Medicina (B Aires)(1989)[2698435]; Rev Clin Esp(1989)[2772294]; Blut(1989)[2660926]; J Med Assoc Thai(1989)[2788692]; Plucne Bolesti(1989)[2636405]; Am J Med(1989)[2729338]; Am J Med(1989)[2929625]; J Med Assoc Thai(1989)[2788694]; Kansenshogaku Zasshi(1989)[2614103]; Enferm Infecc Microbiol Clin(1989)[2490642]; Enferm Infecc Microbiol Clin(1990)[2095272]; Br J Clin Pract(1990)[2126452]; Klin Med (Mosk)(1990)[2186211]; Jpn J Antibiot(1990)[2112207]; Pediatrie(1990)[1963941]; Med Clin (Barc)(1990)[2381245]; Pneumonol Pol(1990)[2191275]; Klin Khir (1962)(1990)[2280524]; Infection(1990)[2079369]; J Formos Med Assoc(1990)[1982123]; Pneumonol Pol(1990)[2216912]; Kansenshogaku Zasshi(1990)[2243193]; J Infect(1990)[2273280]; Zhonghua Min Guo Xiao Er Ke Yi Xue Hui Za Zhi(1990)[2275365]; Pneumologie(1990)[2367474]; J Clin Microbiol(1990)[2179256]; Kansenshogaku Zasshi(1990)[2243194]; Drugs(1991)[1724642]; Mycopathologia(1991)[1795732]; Pediatr Infect Dis J(1991)[2041674]; Rev Clin Esp(1991)[1780543]; Rev Pneumol Clin(1991)[1775874]; Bull Soc Pathol Exot(1991)[1666982]; Scand J Infect Dis(1991)[1957125]; Kansenshogaku Zasshi(1991)[1783813]; Nihon Hinyokika Gakkai Zasshi(1991)[1881014]; Orv Hetil(1991)[1861853]; Bull Soc Pathol Exot(1991)[1819407]; Clin Ther(1991)[1790546]; Clin Radiol(1991)[1760914]; Cancer(1991)[1913526]; J Ky Med Assoc(1992)[1556476]; An Esp Pediatr(1992)[1482029]; Tidsskr Nor Laegeforen(1992)[1412305]; Antimicrob Agents Chemother(1992)[1416892]; Eur J Obstet Gynecol Reprod Biol(1992)[1294416]; East Afr Med J(1992)[1505400]; Bol Med Hosp Infant Mex(1992)[1449625]; J Assoc Physicians India(1992)[1634456]; J Am Geriatr Soc(1992)[1401689]; Kansenshogaku Zasshi(1992)[1402099]; Bol Med Hosp Infant Mex(1992)[1492914]; Med Klin (Munich)(1993)[8437527]; Ann Thorac Surg(1993)[8452451]; J Paediatr Child Health(1993)[8387801]; Rev Clin Esp(1993)[8511376]; Rev Clin Esp(1993)[8259466]; Zhonghua Yi Xue Za Zhi (Taipei)(1993)[8299044]; Chest(1993)[8222797]; Cas Lek Cesk(1993)[8358766]; Am J Infect Control(1993)[8239048]; Med J Aust(1993)[8487685]; Pneumonol Alergol Pol(1993)[8148761]; Anaesthesist(1993)[8250207]; Klin Padiatr(1993)[8487485]; MMWR Recomm Rep(1994)[8164632]; Surg Today(1994)[8054795]; Acta Paediatr(1994)[7819699]; Isr J Med Sci(1994)[8045751]; Jpn J Antibiot(1994)[7933529]; Ann Fr Anesth Reanim(1994)[7992933]; Am J Respir Crit Care Med(1994)[8004324]; Harefuah(1994)[7813926]; Can J Infect Dis(1994)[22346488]; J Infect Dis(1994)[8035009]; Intensive Care Med(1994)[8201105]; Chest(1994)[8306775]; Ann N Y Acad Sci(1994)[7840468]; Enferm Infecc Microbiol Clin(1995)[7654835]; Kansenshogaku Zasshi(1995)[8708405]; Pediatr Pathol Lab Med(1995)[8597814]; J Med Microbiol(1995)[7629859]; J Diarrhoeal Dis Res(1995)[7657960]; J Med Microbiol(1995)[7752213]; Rev Clin Esp(1995)[8532925]; Anaesth Intensive Care(1995)[7573923]; Zhonghua Min Guo Wei Sheng Wu Ji Mian Yi Xue Za Zhi(1995)[9774999]; Can J Infect Dis(1995)[22550412]; Chest(1995)[7555163]; ASAIO J(1996)[8828792]; Hepatology(1996)[8666323]; J Perinatol(1996)[8817439]; Ann Urol (Paris)(1996)[8766145]; Am J Respir Crit Care Med(1996)[8564144]; Pediatr Pol(1996)[8803464]; Indian J Pediatr(1996)[10830046]; Int J Antimicrob Agents(1996)[18611723]; Bacteriol Virusol Parazitol Epidemiol(1996)[8963117]; Eur Respir J(1996)[8947096]; AIDS(1996)[8970683]; Ned Tijdschr Geneeskd(1996)[8668238]; Pediatr Infect Dis J(1996)[8970218]; Anaesthesist(1996)[9065253]; HNO(1996)[8852801]; J Trop Pediatr(1996)[8984216]; Laeknabladid(1996)[20065393]; Arch Pediatr(1996)[8763720]; Pediatrics(1996)[8784356]; J Trop Pediatr(1996)[8820615]; Gastroenterol Hepatol(1996)[8754417]; Pediatr Infect Dis J(1996)[8822283]; Laeknabladid(1996)[20065391]; QJM(1996)[9015487]; Pediatr Radiol(1997)[9028858]; Am Fam Physician(1997)[9012271]; Fukuoka Igaku Zasshi(1997)[9154716]; Curr Opin Pulm Med(1997)[9193863]; Scand J Infect Dis(1997)[9181654]; Radiol Med(1997)[9280934]; Ann Acad Med Singap(1997)[9494663]; Gastroenterol Hepatol(1997)[9410539]; Clin Infect Dis(1997)[9145732]; Rev Hosp Clin Fac Med Sao Paulo(1997)[9334470]; N Z Med J(1997)[9315030]; Med J Malaysia(1997)[10968108]; Kansenshogaku Zasshi(1997)[9248265]; Presse Med(1997)[9365488]; Rev Clin Esp(1997)[9558601]; Acta Cytol(1997)[9100789]; Ren Fail(1997)[9154664]; Eur J Clin Microbiol Infect Dis(1997)[9105839]; Med Clin (Barc)(1998)[9789224]; Clin Microbiol Infect(1998)[11864280]; Arch Bronconeumol(1998)[9656074]; Ann Med Interne (Paris)(1998)[9853050]; Adv Nurse Pract(1998)[9611491]; Eur J Clin Microbiol Infect Dis(1998)[9758274]; Hunan Yi Ke Da Xue Xue Bao(1998)[10681842]; Br J Theatre Nurs(1998)[9677888]; Clin Infect Dis(1998)[9524833]; Emerg Infect Dis(1998)[9716961]; Clin Microbiol Infect(1998)[11864281]; Ann Emerg Med(1998)[9832668]; Am J Cardiol(1998)[9761092]; Arch Pediatr(1998)[10223164]; Diagn Microbiol Infect Dis(1998)[9934546]; Zhonghua Min Guo Xiao Er Ke Yi Xue Hui Za Zhi(1998)[9823677]; Arch Intern Med(1998)[9570172]; AIDS(1998)[9631140]; Khirurgiia (Sofiia)(1998)[9974040]; J Perinatol(1998)[9730200]; J Microbiol Immunol Infect(1999)[10650491]; FEMS Immunol Med Microbiol(1999)[10443502]; J Infect Dis(1999)[10558935]; Semin Respir Infect(1999)[10638512]; QJM(1999)[10627862]; J Assoc Physicians India(1999)[10862331]; J Pediatr (Rio J)(1999)[14685562]; Klin Padiatr(1999)[10407810]; Rev Esp Quimioter(1999)[10855016]; Rev Esp Quimioter(1999)[10855018]; Med Arh(1999)[10758760]; Rev Esp Quimioter(1999)[10878529]; Pediatrics(1999)[10103331]; An Esp Pediatr(1999)[10083638]; Ryoikibetsu Shokogun Shirizu(1999)[10088419]; Acta Neurochir (Wien)(1999)[10672301]; Rev Esp Quimioter(1999)[10878531]; Crit Care Med(1999)[10470744]; Pediatr Infect Dis J(1999)[10530574]; Dtsch Med Wochenschr(1999)[10480011]; Infez Med(1999)[12748443]; Ugeskr Laeger(2000)[10860428]; Clin Infect Dis(2000)[10913417]; Exp Clin Endocrinol Diabetes(2000)[10826524]; Indian J Pediatr(2000)[10832217]; Diagn Microbiol Infect Dis(2000)[10794943]; J Pain Symptom Manage(2000)[11068154]; J Postgrad Med(2000)[10855072]; Can J Infect Dis(2000)[18159274]; Isr Med Assoc J(2000)[11344776]; Antibiot Khimioter(2000)[10768061]; Med Oncol(2000)[10871816]; Infect Control Hosp Epidemiol(2000)[10968716]; P N G Med J(2000)[11407623]; Eur J Clin Microbiol Infect Dis(2001)[11347669]; Thorac Cardiovasc Surg(2001)[11339448]; Med J Malaysia(2001)[11732071]; J Microbiol Immunol Infect(2001)[11456359]; J Trop Pediatr(2001)[11827304]; Eur J Clin Microbiol Infect Dis(2001)[11837636]; Arch Pediatr Adolesc Med(2001)[11483127]; An Med Interna(2001)[11594181]; Am J Infect Control(2001)[11172313]; Diagn Microbiol Infect Dis(2001)[11687309]; Eur J Clin Microbiol Infect Dis(2001)[11305468]; J Med Liban(2001)[12243418]; Acta Gastroenterol Latinoam(2001)[11873668]; Indian J Gastroenterol(2001)[11817784]; Ned Tijdschr Geneeskd(2001)[11455692]; J Pediatr Surg(2001)[11227003]; Rev Clin Esp(2001)[11387820]; Indian J Pediatr(2001)[11563247]; J Neurol(2001)[11757959]; Arch Dis Child(2001)[11159294]; Dan Med Bull(2001)[11767129]; Gac Med Mex(2001)[11432088]; Rev Esp Quimioter(2001)[11856984]; Indian J Pediatr(2001)[11770243]; East Afr Med J(2001)[11921576]; Emerg Infect Dis(2002)[11749750]; Pediatrics(2002)[12165593]; Paediatr Drugs(2002)[11888355]; Presse Med(2002)[12148255]; Pharmacotherapy(2002)[11794426]; Curr Treat Options Neurol(2002)[11931731]; J Perinat Med(2002)[12012636]; Southeast Asian J Trop Med Public Health(2002)[12757224]; Am J Med(2002)[11893347]; J Med Assoc Thai(2002)[12403233]; J Pediatr(2002)[12183723]; Tidsskr Nor Laegeforen(2002)[12448252]; East Afr Med J(2002)[12643233]; Dtsch Med Wochenschr(2002)[12432483]; Gerodontology(2002)[12542215]; Ugeskr Laeger(2002)[11838420]; Diagn Microbiol Infect Dis(2002)[12493178]; Chest(2003)[12853512]; Zhonghua Yi Xue Za Zhi(2003)[12820912]; Infect Control Hosp Epidemiol(2003)[14700406]; J Hosp Infect(2003)[12818590]; Mayo Clin Health Lett(2003)[14593993]; Rev Med Suisse Romande(2003)[15095700]; Tumori(2003)[12903572]; J Clin Microbiol(2003)[12791846]; Harefuah(2003)[12754875]; Clin Infect Dis(2003)[12652388]; Clin Microbiol Infect(2003)[14616699]; J Cardiothorac Vasc Anesth(2003)[12635056]; J Clin Microbiol(2003)[14662987]; BMC Nephrol(2004)[15318947]; P R Health Sci J(2004)[15631176]; Zhong Nan Da Xue Xue Bao Yi Xue Ban(2004)[16145922]; Diagn Microbiol Infect Dis(2004)[15023432]; Clin Microbiol Infect(2004)[15191383]; Tuberk Toraks(2004)[15558355]; Indian J Pediatr(2004)[15053375]; Eur Respir J(2004)[15358692]; Am J Infect Control(2004)[15175611]; Emerg Infect Dis(2004)[15030711]; Eur J Intern Med(2004)[15450992]; Chest(2004)[14769717]; Diagn Microbiol Infect Dis(2004)[15135498]; Pediatrics(2004)[14702495]; Ann Rheum Dis(2004)[15361392]; Crit Care Med(2004)[15071391]; Iran J Allergy Asthma Immunol(2004)[17301389]; Infect Control Hosp Epidemiol(2004)[15484801]; MMWR Surveill Summ(2004)[15499306]; Southeast Asian J Trop Med Public Health(2004)[15691151]; Zhonghua Liu Xing Bing Xue Za Zhi(2004)[15231150]; Undersea Hyperb Med(2004)[15568415]; BMC Microbiol(2004)[15320954]; Can J Infect Dis(2004)[18159439]; J Med Assoc Thai(2004)[15521239]; J Ayub Med Coll Abbottabad(2004)[15762062]; Recenti Prog Med(2004)[15032335]; Indian J Chest Dis Allied Sci(2004)[14870864]; Ned Tijdschr Geneeskd(2004)[15453124]; Med Mal Infect(2004)[15679235]; J Paediatr Child Health(2004)[15265191]; J Med Assoc Thai(2005)[16149693]; J Microbiol Immunol Infect(2005)[15843855]; Lakartidningen(2005)[16408703]; Eur J Dermatol(2005)[16048763]; J Infect(2005)[16230186]; Int J Antimicrob Agents(2005)[16280243]; Int J Clin Pract(2005)[15707463]; Respiration(2005)[15753643]; Antimicrob Agents Chemother(2005)[15673761]; Pediatr Nephrol(2005)[15834619]; Med Arh(2005)[15822680]; An Med Interna(2005)[15839825]; Saudi Med J(2005)[16228055]; Infect Control Hosp Epidemiol(2005)[16276959]; Chemotherapy(2005)[16103664]; Saudi Med J(2006)[16680246]; Zhongguo Dang Dai Er Ke Za Zhi(2006)[16923372]; Przegl Epidemiol(2006)[16964673]; Infect Control Hosp Epidemiol(2006)[16807862]; Med Princ Pract(2006)[16763401]; J Infect(2006)[16457891]; Pflege Z(2006)[16502999]; Afr J Med Med Sci(2006)[17312749]; J Infect(2006)[16343637]; Diagn Microbiol Infect Dis(2006)[16529901]; An Med Interna(2006)[16566654]; Int J Tuberc Lung Dis(2006)[17131790]; J Chemother(2006)[17267336]; Zhonghua Jie He He Hu Xi Za Zhi(2006)[16638292]; Clin Transplant(2006)[16842513]; Zhongguo Dang Dai Er Ke Za Zhi(2006)[17052391]; J Perinat Med(2006)[16519624]; Zhongguo Dang Dai Er Ke Za Zhi(2006)[17052392]; Med Intensiva(2006)[16938192]; Anesthesiology(2007)[17893477]; Kyobu Geka(2007)[18078093]; Rev Chilena Infectol(2007)[17369967]; Int J Surg Pathol(2007)[17172507]; Am J Emerg Med(2007)[17276814]; Infect Control Hosp Epidemiol(2007)[17926284]; Southeast Asian J Trop Med Public Health(2007)[17877230]; Zhong Nan Da Xue Xue Bao Yi Xue Ban(2007)[17478953]; Enferm Infecc Microbiol Clin(2007)[17261247]; Infection(2007)[17721736]; Ethiop Med J(2007)[17642174]; J Coll Physicians Surg Pak(2007)[17374297]; Saudi Med J(2007)[17206302]; Am J Infect Control(2007)[17433942]; Rev Gastroenterol Mex(2007)[17685201]; Ann Thorac Med(2007)[19727346]; Saudi Med J(2007)[17268702]; J Microbiol Immunol Infect(2007)[18087630]; Spine (Phila Pa 1976)(2007)[17268255]; Med Intensiva(2007)[17306135]; Lancet Infect Dis(2008)[18291338]; Skeletal Radiol(2008)[18496688]; Am J Infect Control(2008)[18468549]; Antimicrob Agents Chemother(2008)[18285482]; Int J Antimicrob Agents(2008)[18358701]; Rev Iberoam Micol(2008)[18338934]; Pediatr Hematol Oncol(2008)[18569840]; World J Pediatr(2008)[18661767]; Zhonghua Jie He He Hu Xi Za Zhi(2008)[19080534]; Kansenshogaku Zasshi(2008)[18975590]; Infect Control Hosp Epidemiol(2008)[18947320]; Zhonghua Er Ke Za Zhi(2008)[19099875]; Eur Respir J(2008)[18978148]; An Pediatr (Barc)(2008)[18928695]; Zhongguo Dang Dai Er Ke Za Zhi(2008)[18947473]; Arch Pediatr(2008)[18524552]; Zhongguo Dang Dai Er Ke Za Zhi(2008)[18289463]; Cent Afr J Med(2008)[21648126]; Indian J Community Med(2008)[19967031]; Med Princ Pract(2008)[18685276]; Nihon Kokyuki Gakkai Zasshi(2008)[18939411]; Southeast Asian J Trop Med Public Health(2008)[19058610]; Rev Med Interne(2009)[18450332]; Medicine (Baltimore)(2009)[19282702]; Transplant Proc(2009)[19249559]; Zentralbl Chir(2009)[19382051]; Int J Tuberc Lung Dis(2009)[19861005]; J Int Med Res(2009)[19589276]; Braz J Microbiol(2009)[24031336]; J Chin Med Assoc(2009)[19687002]; Praxis (Bern 1994)(2009)[19672827]; J Microbiol Immunol Infect(2009)[19597649]; J Infect Dev Ctries(2009)[19762955]; Vojnosanit Pregl(2009)[20017416]; Zhongguo Dang Dai Er Ke Za Zhi(2009)[20113598]; Trop Med Int Health(2009)[19772545]; Anestezjol Intens Ter(2009)[20201343]; Spine (Phila Pa 1976)(2009)[19139655]; Pediatr Infect Dis J(2009)[19106757]; Rev Chilena Infectol(2009)[19621141]; Pediatr Infect Dis J(2009)[19106758]; J Microbiol Immunol Infect(2009)[20182669]; Clin Infect Dis(2009)[19191615]; J Trauma(2009)[19430237]; Indian Pediatr(2009)[19213982]; Mymensingh Med J(2009)[19377438]; Am J Trop Med Hyg(2010)[20207859]; Int J Antimicrob Agents(2010)[20580534]; J Adv Pharm Technol Res(2010)[22247847]; Respirology(2010)[20546191]; Turk J Haematol(2010)[27265793]; Tuberk Biolezni Legkih(2010)[27529925]; Saudi Med J(2010)[21135998]; Clin Infect Dis(2010)[20597676]; Med Intensiva(2010)[20605269]; Afr J Infect Dis(2010)[23878695]; Lung India(2010)[20616935]; J Assoc Physicians India(2010)[21568008]; J Infect Dev Ctries(2010)[20818104]; Hip Int(2010)[20544658]; Infection(2010)[20878457]; S Afr Med J(2010)[20529438]; J Infect Dev Ctries(2010)[20440059]; Zhonghua Er Ke Za Zhi(2010)[21055271]; Zhongguo Dang Dai Er Ke Za Zhi(2010)[20350426]; Chin Med J (Engl)(2010)[21034629]; Pathol Biol (Paris)(2010)[19875244]; J Paediatr Child Health(2010)[20796185]; J Res Health Sci(2010)[22911918]; Med Pregl(2010)[21446092]; Srp Arh Celok Lek(2010)[20607980]; Zh Mikrobiol Epidemiol Immunobiol(2010)[21384588]; Ann Thorac Med(2011)[21572702]; Int J Infect Dis(2011)[21450505]; Afr J Med Med Sci(2011)[22783679]; Arch Pediatr(2011)[21269814]; Intern Med(2011)[21963747]; J Microbiol Immunol Infect(2011)[21524962]; QJM(2011)[20829191]; Med Intensiva(2011)[21334104]; J Indian Med Assoc(2011)[22315846]; Hong Kong Med J(2011)[21636872]; Hepatol Res(2011)[21951874]; Clin Transplant(2011)[22150886]; Clin Microbiol Infect(2011)[20491828]; Can J Ophthalmol(2011)[21995996]; J Pediatr Hematol Oncol(2011)[21285902]; Biomed Pap Med Fac Univ Palacky Olomouc Czech Repub(2011)[22336651]; Lancet Infect Dis(2011)[21126917]; Klin Mikrobiol Infekc Lek(2011)[22052100]; J Infect(2011)[21056057]; Med Sci Monit(2011)[21525819]; World J Emerg Med(2011)[25214995]; Iran J Pediatr(2011)[23056825]; Zhonghua Er Ke Za Zhi(2011)[22336359]; Zhongguo Dang Dai Er Ke Za Zhi(2011)[21251380]; BMJ Case Rep(2011)[22696742]; Indian J Med Microbiol(2011)[21654106]; GMS Krankenhhyg Interdiszip(2011)[22242095]; Int J Clin Pharm(2011)[21984226]; Rev Mal Respir(2011)[21482336]; Med Clin (Barc)(2011)[21514606]; Respir Care(2011)[21605476]; Eur J Clin Microbiol Infect Dis(2011)[20859753]; Saudi Med J(2011)[21556470]; Transplant Proc(2011)[21440756]; World J Pediatr(2011)[20549409]; Zhongguo Wei Zhong Bing Ji Jiu Yi Xue(2011)[22093315]; Antimicrob Agents Chemother(2011)[21263048]; Indian J Med Res(2012)[22825611]; J Med Assoc Thai(2012)[23513480]; Tohoku J Exp Med(2012)[22729220]; Am J Infect Control(2012)[21908073]; Rev Argent Microbiol(2012)[22610291]; J Microbiol Immunol Infect(2012)[22041167]; Saudi J Kidney Dis Transpl(2012)[22569460]; East Mediterr Health J(2012)[22764437]; Nihon Ronen Igakkai Zasshi(2012)[22466781]; Saudi J Kidney Dis Transpl(2012)[22982916]; Pathol Biol (Paris)(2012)[21719212]; J Infect Dev Ctries(2012)[22337840]; Am J Emerg Med(2012)[22030178]; Arch Iran Med(2012)[22924377]; J Med Assoc Thai(2012)[22435245]; Afr Health Sci(2012)[23056018]; Int Arch Med(2012)[23075077]; BMC Infect Dis(2012)[22436174]; Zhongguo Dang Dai Er Ke Za Zhi(2012)[22613105]; Mikrobiyol Bul(2012)[22951654]; Zhonghua Nei Ke Za Zhi(2012)[22943829]; Zhonghua Xin Xue Guan Bing Za Zhi(2012)[23302668]; J Med Assoc Thai(2012)[23130469]; J Glob Infect Dis(2012)[22529622]; Zhongguo Wei Zhong Bing Ji Jiu Yi Xue(2012)[23131289]; Eur Urol(2012)[22575912]; Zhongguo Dang Dai Er Ke Za Zhi(2012)[23234773]; BMC Infect Dis(2012)[23267668]; Rev Esp Quimioter(2012)[22488539]; Rev Soc Bras Med Trop(2012)[22767099]; Crit Care Resusc(2012)[22963214]; J Coll Physicians Surg Pak(2012)[22414355]; Eur Respir J(2013)[23100491]; Korean J Gastroenterol(2013)[24077630]; Pediatr Int(2013)[24330298]; Exp Ther Med(2013)[23251300]; J Clin Diagn Res(2013)[24179913]; Clin Pediatr (Phila)(2013)[23661790]; Int J Antimicrob Agents(2013)[24071027]; J Health Popul Nutr(2013)[24592596]; Crit Care Med(2013)[23660733]; J Formos Med Assoc(2013)[23332427]; Am J Respir Crit Care Med(2013)[23540875]; Transplant Proc(2013)[23972528]; Pharm Pract (Granada)(2013)[24155856]; Sichuan Da Xue Xue Bao Yi Xue Ban(2013)[24059113]; BMJ Open(2013)[24176795]; J R Army Med Corps(2013)[24109134]; Zhonghua Nei Ke Za Zhi(2013)[23856111]; J Family Med Prim Care(2013)[24479091]; Zhongguo Dang Dai Er Ke Za Zhi(2013)[23336167]; S Afr Med J(2013)[23725954]; J Hosp Infect(2013)[23374289]; MEDICC Rev(2013)[23686252]; BMJ Open(2013)[24091424]; Transplant Proc(2013)[24314931]; Chin Med J (Engl)(2013)[23786927]; Caspian J Intern Med(2013)[24009954]; Geriatr Gerontol Int(2013)[23170823]; J Res Pharm Pract(2013)[24991607]; PLoS One(2013)[23593267]; BMJ Case Rep(2013)[23946529]; J Clin Diagn Res(2013)[24392373]; Eur Spine J(2013)[23543389]; J Chin Med Assoc(2013)[23351417]; Zhongguo Dang Dai Er Ke Za Zhi(2013)[23336161]; Infect Control Hosp Epidemiol(2013)[23221186]; Lung(2013)[23564195]; N Am J Med Sci(2013)[23923107]; Australas Med J(2013)[24133535]; Zhonghua Yu Fang Yi Xue Za Zhi(2013)[24378133]; Pediatr Infect Dis J(2013)[23411624]; J Infect Dev Ctries(2013)[23592640]; Zhonghua Wei Zhong Bing Ji Jiu Yi Xue(2013)[24021044]; Hepatol Int(2013)[26201642]; Biomed Res Int(2013)[24175299]; Ann Pharmacother(2013)[23673532]; Pediatr Pulmonol(2014)[23794463]; J Vasc Interv Radiol(2014)[25255950]; Medicine (Baltimore)(2014)[24797169]; Infect Dis Rep(2014)[25276329]; J Chemother(2014)[24650326]; Transplantation(2014)[24162251]; Lupus(2014)[25078055]; Chin Med J (Engl)(2014)[24824237]; Infection(2014)[23709293]; Rev Esp Anestesiol Reanim(2014)[23706936]; Case Rep Med(2014)[24872819]; Oxf Med Case Reports(2014)[25988064]; Am J Med(2014)[24530952]; J Infect Dev Ctries(2014)[25212077]; J Med Microbiol(2014)[25168964]; Korean J Intern Med(2014)[24574833]; Diagn Microbiol Infect Dis(2014)[24582578]; Int J Antimicrob Agents(2014)[24630306]; Clin Nephrol(2014)[23380391]; J Thorac Cardiovasc Surg(2014)[25240522]; Int J Chron Obstruct Pulmon Dis(2014)[25298733]; Zhonghua Er Ke Za Zhi(2014)[24680408]; Nihon Jinzo Gakkai Shi(2014)[24956885]; Ann Intensive Care(2014)[25593750]; BMJ Open Respir Res(2014)[25478172]; J Infect Dev Ctries(2014)[24423714]; Ann Agric Environ Med(2014)[25292115]; BMC Res Notes(2014)[24996427]; Indian J Cancer(2014)[26842169]; Iran J Public Health(2014)[26175971]; Zhonghua Yu Fang Yi Xue Za Zhi(2014)[25619215]; Antimicrob Resist Infect Control(2014)[25237477]; Zhonghua Jie He He Hu Xi Za Zhi(2014)[25351266]; Cell Biochem Biophys(2015)[25347986]; Drug Resist Updat(2015)[26004211]; Transpl Infect Dis(2015)[25846286]; J Neurol Surg Rep(2015)[26251817]; Clin Microbiol Infect(2015)[25698659]; Am J Emerg Med(2015)[25498529]; Zhonghua Yi Xue Za Zhi(2015)[26704158]; Zhongguo Dang Dai Er Ke Za Zhi(2015)[26014687]; Iran Biomed J(2015)[26220641]; Epidemiol Mikrobiol Imunol(2015)[26099612]; Eur J Clin Microbiol Infect Dis(2015)[25926305]; Int J Antimicrob Agents(2015)[26358970]; MSMR(2015)[26115171]; PLoS One(2015)[26407326]; Fukuoka Igaku Zasshi(2015)[26021128]; Pan Afr Med J(2015)[27047618]; Medicine (Baltimore)(2015)[26107669]; Am J Trop Med Hyg(2015)[25548379]; Rev Esp Med Nucl Imagen Mol(2015)[25065971]; Medicine (Baltimore)(2015)[26632737]; Rev Med Suisse(2015)[25799655]; Pathol Biol (Paris)(2015)[25553645]; J Breath Res(2015)[25557917]; Virusdisease(2015)[26436125]; Klin Mikrobiol Infekc Lek(2015)[26098488]; J Thorac Dis(2015)[26543614]; Transplant Proc(2015)[25769594]; Int J Risk Saf Med(2015)[26639714]; Transplant Proc(2015)[26093756]; JNMA J Nepal Med Assoc(2015)[27746463]; Niger Med J(2015)[26759518]; Surg Infect (Larchmt)(2015)[25761081]; Case Rep Infect Dis(2015)[25628903]; J Ayub Med Coll Abbottabad(2015)[26182754]; PLoS One(2016)[27959904]; Hinyokika Kiyo(2016)[27624108]; Pediatr Int(2016)[27460398]; G Ital Dermatol Venereol(2016)[27824225]; Infect Dis (Lond)(2016)[26763410]; Infect Dis (Lond)(2016)[26577519]; Intern Emerg Med(2016)[26951186]; Przegl Epidemiol(2016)[27344468]; Infect Control Hosp Epidemiol(2016)[26456803]; Rev Med Inst Mex Seguro Soc(2016)[27197104]; Zhonghua Er Ke Za Zhi(2016)[26875468]; Pan Afr Med J(2016)[27642476]; Infect Control Hosp Epidemiol(2016)[27573805]; Medicine (Baltimore)(2016)[27015202]; Ann Clin Lab Sci(2016)[27466299]; Zhonghua Yi Xue Za Zhi(2016)[27266348]; J Microbiol Immunol Infect(2016)[25070279]; Medicine (Baltimore)(2016)[26886594]; Biomed Res Int(2016)[28044137]; Pulm Med(2016)[26998356]; Pak J Med Sci(2016)[27648021]; Prehosp Emerg Care(2016)[26024065]; J Med Case Rep(2016)[27821139]; Cureus(2016)[27800290]; Adv Med Sci(2016)[26583299]; Indian J Med Paediatr Oncol(2016)[27051152]; J Rheumatol(2016)[27307524]; Infection(2016)[26062812]; Childs Nerv Syst(2016)[27066799]; Gac Med Mex(2016)[27595249]; Semin Respir Crit Care Med(2016)[27960207]; Saudi Med J(2016)[27570854]; Transplant Proc(2016)[26915861]; Nan Fang Yi Ke Da Xue Xue Bao(2016)[27998866]; J Infect(2016)[27394401]; Pneumonia (Nathan)(2016)[28702283]; Biomed Pap Med Fac Univ Palacky Olomouc Czech Repub(2016)[27003315]; Respir Med Case Rep(2016)[27419065]; J Clin Diagn Res(2016)[26894065]; PLoS One(2016)[26872131]; Respirology(2016)[27417156]; MMWR Morb Mortal Wkly Rep(2016)[27559759]; J Clin Diagn Res(2016)[27790429]; Exp Clin Transplant(2016)[26789197]; J Infect Dev Ctries(2016)[27031457]; Med Dosw Mikrobiol(2016)[28146621]; Jundishapur J Microbiol(2016)[27942366]; Chin Med J (Engl)(2016)[26830989]; Am J Infect Control(2016)[27339791]; Transpl Infect Dis(2016)[27188439]; J Med Assoc Thai(2016)[27501600]; Eur J Clin Microbiol Infect Dis(2017)[27639859]; Med Mal Infect(2017)[27894516]; Microb Drug Resist(2017)[27096168]; J Chemother(2017)[27347770]; J Med Microbiol(2017)[27911257]; Eur J Clin Microbiol Infect Dis(2017)[27287765]; J Infect Public Health(2017)[28642140]; Pak J Pharm Sci(2017)[29175778]; Tohoku J Exp Med(2017)[28496015]; J Water Health(2017)[29040071]; Lett Appl Microbiol(2017)[28905401]; J Assoc Physicians India(2017)[28457027]; BMC Infect Dis(2017)[28683724]; Zhonghua Xue Ye Xue Za Zhi(2017)[28810329]; Chin Med J (Engl)(2017)[28836551]; J Hosp Infect(2017)[28007308]; Lung Cancer(2017)[29173772]; Afr J Infect Dis(2017)[28670639]; Surg Infect (Larchmt)(2017)[28475416]; East Afr Health Res J(2017)[34308158]; Zhonghua Er Ke Za Zhi(2017)[28881515]; Open Forum Infect Dis(2017)[29026867]; Med J Armed Forces India(2017)[28790779]; Antimicrob Agents Chemother(2017)[28069649]; Rev Med Inst Mex Seguro Soc(2017)[29792794]; Am J Infect Control(2017)[27856076]; Rev Med Inst Mex Seguro Soc(2017)[28212478]; BMC Med Imaging(2017)[28068928]; Am J Med Sci(2017)[29173358]; J Clin Diagn Res(2017)[28384858]; Rev Esp Quimioter(2017)[27898208]; J Nat Sci Biol Med(2017)[28250674]; Pediatr Infect Dis J(2017)[28005691]; Semergen(2017)[27773623]; BMC Infect Dis(2017)[28893195]; Ann Intensive Care(2017)[28447330]; Respirology(2017)[28681941]; Zhonghua Wei Zhong Bing Ji Jiu Yi Xue(2017)[28936955]; Zhongguo Dang Dai Er Ke Za Zhi(2017)[28899468]; J Ayub Med Coll Abbottabad(2017)[29076682]; Urologiia(2017)[28631909]; Hum Vaccin Immunother(2017)[28922613]; Zhonghua Yu Fang Yi Xue Za Zhi(2017)[29036990]; BMC Res Notes(2017)[28754148]; Tohoku J Exp Med(2018)[29618675]; J Nepal Health Res Counc(2018)[29717284]; Infect Control Hosp Epidemiol(2018)[29655388]; Intensive Care Med(2018)[29379992]; Saudi Med J(2018)[30520499]; Infect Control Hosp Epidemiol(2018)[29249216]; Wiad Lek(2018)[30737942]; Med Sci Monit(2018)[30482891]; Epidemiol Infect(2018)[30355372]; Nihon Hinyokika Gakkai Zasshi(2018)[31006742]; Clin Microbiol Infect(2018)[28559001]; Stroke(2018)[29915122]; Clin Infect Dis(2018)[29408951]; J Hosp Infect(2018)[29051092]; World Neurosurg(2018)[29191537]; Zhonghua Er Ke Za Zhi(2018)[29342994]; Med J Malaysia(2018)[30647223]; Respir Med Case Rep(2018)[30386722]; J Thorac Dis(2018)[29997928]; Ann Clin Microbiol Antimicrob(2018)[29571291]; Indian J Crit Care Med(2018)[29422728]; PLoS One(2018)[29953464]; Case Rep Pediatr(2018)[29888019]; J Antimicrob Chemother(2018)[30060117]; Antimicrob Agents Chemother(2018)[29203488]; Respir Med Case Rep(2018)[29977769]; Indian J Pediatr(2018)[29616405]; Infect Drug Resist(2018)[30519063]; Rev Fac Cien Med Univ Nac Cordoba(2018)[30296022]; Int J Gen Med(2018)[29317845]; Case Rep Infect Dis(2018)[29593918]; Afr J Lab Med(2018)[30568902]; Acute Crit Care(2018)[31723866]; East Afr Health Res J(2018)[34308168]; Infect Drug Resist(2018)[30464557]; Int J Environ Res Public Health(2018)[29324651]; Respir Med(2018)[29605219]; Antimicrob Resist Infect Control(2018)[29541448]; Turk J Pediatr(2018)[30102484]; Lung(2018)[29691645]; Emerg Med Int(2018)[30345116]; Ann Ib Postgrad Med(2018)[31217775]; Zhongguo Shi Yan Xue Ye Xue Za Zhi(2018)[29950242]; J Int Med Res(2018)[30027805]; Antimicrob Resist Infect Control(2018)[30455867]; Ann Agric Environ Med(2019)[31885243]; Biomed Res Int(2019)[31179337]; J Bronchology Interv Pulmonol(2019)[30908392]; Thorax(2019)[30420408]; Clin Infect Dis(2019)[30358811]; BMC Surg(2019)[31412850]; J Hosp Infect(2019)[31128970]; BMC Endocr Di |
| *Haemophilus influenzae* | Established pathogen | 1736 | **established** | J Am Med Assoc(1946)[21025619]; Proc Soc Exp Biol Med(1947)[20287366]; Proc Soc Exp Biol Med(1947)[20287365]; J Thorac Surg(1948)[18865486]; Lancet(1951)[14874413]; AMA Arch Intern Med(1954)[13157679]; Dis Chest(1954)[13127717]; Pediatrics(1955)[14394736]; Am J Med Sci(1959)[14444150]; Schweiz Med Wochenschr(1962)[13954195]; Ugeskr Laeger(1962)[13890573]; Allerg Asthma (Leipz)(1962)[13945214]; Am J Dis Child(1962)[13905103]; Ann Intern Med(1962)[14454874]; Am J Dis Child(1963)[13987791]; Cas Lek Cesk(1963)[14114579]; Med Thorac(1963)[14138588]; Gig Tr Prof Zabol(1963)[14109780]; Am J Dis Child(1963)[13987790]; Can Med Assoc J(1963)[14055830]; Poumon Coeur(1964)[14200889]; Am J Med Sci(1964)[14106884]; Acta Chir Scand(1964)[14171727]; Maandschr Kindergeneeskd(1964)[14224082]; Cesk Epidemiol Mikrobiol Imunol(1964)[14162969]; Med Sci(1964)[14118936]; Br J Dis Chest(1964)[14104970]; J Pathol Bacteriol(1964)[14194995]; Br Med J(1965)[5317929]; Lancet(1965)[4157848]; Ann Intern Med(1965)[14258349]; J Hyg (Lond)(1965)[14308350]; Laryngoscope(1965)[14256354]; Acta Paediatr Scand(1966)[4289665]; Indian Pediatr(1966)[5296655]; Arch Environ Health(1966)[5921283]; Am J Med(1966)[5297038]; Radiology(1966)[5295865]; Lancet(1967)[4165645]; Ann Intern Med(1967)[5297130]; Lancet(1967)[12389569]; Bronches(1968)[4386477]; Arch Intern Med(1968)[5645719]; Br Med J(1968)[5302853]; Anglo Ger Med Rev(1969)[5311686]; Med Lett Drugs Ther(1969)[5307043]; Naika(1970)[5312131]; Mykosen(1970)[5311015]; Poumon Coeur(1970)[4393036]; Am J Med(1971)[5314534]; Nihon Shonika Gakkai Zasshi(1971)[5314154]; Mars Med(1972)[4538919]; Br Med J(1972)[4537452]; Rev Med Suisse Romande(1972)[4537946]; Med J Aust(1972)[4404399]; Can Med Assoc J(1973)[4147709]; Plucne Bolesti Tuberk(1974)[4155512]; Schweiz Med Wochenschr(1975)[2971]; Pediatrics(1975)[1081672]; Am Rev Respir Dis(1975)[807138]; Acta Otolaryngol(1975)[885]; Med J Aust(1975)[1079300]; Nihon Kyobu Shikkan Gakkai Zasshi(1975)[813045]; Am J Dis Child(1975)[1079691]; Isr J Med Sci(1975)[1081984]; Bull Int Union Tuberc(1976)[31959]; Lancet(1976)[73690]; Clin Pediatr (Phila)(1976)[1083325]; Pediatrics(1976)[934743]; J Clin Pathol(1977)[325018]; Am J Dis Child(1977)[319655]; Arch Dis Child(1977)[343723]; J Fam Pract(1977)[320285]; Am J Med(1977)[835601]; JAMA(1977)[301574]; Can Med Assoc J(1977)[303933]; Pol Tyg Lek(1977)[917917]; Am J Med(1977)[302644]; South Med J(1977)[300177]; J Pediatr(1978)[690752]; Southeast Asian J Trop Med Public Health(1978)[375407]; Chest(1978)[738098]; Arch Dis Child(1978)[306225]; Am J Med Sci(1978)[307346]; Clin Pediatr (Phila)(1978)[630776]; South Med J(1978)[309660]; J Pediatr(1978)[621604]; Schweiz Med Wochenschr(1978)[305660]; J Pediatr(1978)[307594]; Acta Paediatr Scand(1978)[307897]; J Okla State Med Assoc(1978)[304475]; Am J Dis Child(1979)[312602]; Am Rev Respir Dis(1979)[316298]; J Pediatr(1979)[312930]; Medicine (Baltimore)(1979)[310943]; Am J Dis Child(1979)[443216]; J Pediatr(1979)[370354]; Medicina (B Aires)(1979)[316851]; Harefuah(1979)[317263]; Am J Dis Child(1979)[310640]; P N G Med J(1979)[299332]; Prim Care(1979)[392574]; Pneumonol Pol(1979)[119214]; Ann Med Interne (Paris)(1980)[6968526]; Ter Arkh(1980)[6972629]; Ugeskr Laeger(1980)[6250259]; Postgrad Med J(1980)[6969397]; P N G Med J(1980)[7008428]; South Med J(1980)[6966825]; Pediatrics(1980)[6967585]; Arch Intern Med(1980)[6966913]; Ter Arkh(1980)[6966837]; Clin Pediatr (Phila)(1980)[6965366]; Scand J Infect Dis(1980)[6966070]; J Antimicrob Chemother(1980)[6252154]; J Antimicrob Chemother(1980)[6967871]; Am Rev Respir Dis(1980)[6968168]; South Med J(1981)[6970414]; N Z Med J(1981)[6977748]; Nouv Presse Med(1981)[6259612]; Am Rev Respir Dis(1981)[7224344]; Laryngoscope(1981)[7005582]; Clin Radiol(1981)[6971206]; South Med J(1981)[6970416]; Perspect Biol Med(1981)[7038618]; Arch Intern Med(1981)[7235817]; Ter Arkh(1981)[7027504]; Am J Clin Pathol(1981)[6975035]; Thorax(1981)[7314031]; Arch Intern Med(1982)[6978116]; South Med J(1982)[7038889]; South Med J(1982)[6979798]; Scand J Infect Dis(1982)[7163783]; Medicine (Baltimore)(1982)[7038375]; Postgrad Med(1982)[6976567]; Zh Mikrobiol Epidemiol Immunobiol(1982)[6758431]; Otolaryngol Head Neck Surg(1982)[6810258]; Pediatr Infect Dis(1982)[6755404]; Arch Otolaryngol(1982)[6980642]; Diagn Microbiol Infect Dis(1983)[6199156]; Med Clin (Barc)(1983)[6602922]; Postgrad Med(1983)[6600837]; Pathol Biol (Paris)(1983)[6341938]; Jpn J Antibiot(1983)[6348340]; Br J Dis Chest(1983)[6605762]; Pathol Biol (Paris)(1983)[6341949]; Eur J Respir Dis(1983)[6825749]; Chest(1983)[6602694]; Am Fam Physician(1983)[6605672]; J Pediatr(1983)[6600278]; Am J Infect Control(1983)[6349427]; Rev Med Chir Soc Med Nat Iasi(1983)[6610192]; Pathol Biol (Paris)(1983)[6341937]; Am Rev Respir Dis(1983)[6412607]; Int J Pediatr Otorhinolaryngol(1983)[6609903]; J Clin Microbiol(1983)[6605362]; J Infect(1983)[6604106]; Postgrad Med(1983)[6600838]; Lab Delo(1983)[6194362]; Aust N Z J Med(1983)[6606417]; Arch Intern Med(1983)[6603201]; Kansenshogaku Zasshi(1983)[6443735]; Pediatr Infect Dis(1984)[6334843]; Pharmacotherapy(1984)[6371723]; Eur J Clin Microbiol(1984)[6332016]; Aust N Z J Med(1984)[6591905]; Rev Med Brux(1984)[6097981]; Hosp Pract (Off Ed)(1984)[6420325]; Lab Delo(1984)[6204117]; AIDS Res(1984)[6546007]; J Infect(1984)[6334118]; Gerontology(1984)[6333374]; Postgrad Med(1984)[6371752]; Radiology(1984)[6608117]; Zh Mikrobiol Epidemiol Immunobiol(1984)[6611002]; Ann Intern Med(1984)[6607006]; Can J Neurol Sci(1984)[6704799]; J Tenn Med Assoc(1984)[6334196]; Pediatr Infect Dis(1984)[6701101]; Am J Clin Pathol(1984)[6380270]; Clin Pediatr (Phila)(1984)[6705435]; Geriatrics(1984)[6332044]; Am J Clin Pathol(1984)[6606978]; An Esp Pediatr(1984)[6703532]; Pediatrics(1984)[6718113]; Antimicrob Agents Chemother(1985)[3875310]; Am J Med Sci(1985)[3872070]; Pathol Biol (Paris)(1985)[3897966]; Ann Intern Med(1985)[2996410]; Crit Care Med(1985)[4006503]; Bull Soc Pathol Exot Filiales(1985)[3836780]; Pathol Biol (Paris)(1985)[3897973]; Eur J Clin Microbiol(1985)[4018066]; Schweiz Med Wochenschr(1985)[3871965]; Indian J Pediatr(1985)[3879243]; Pediatr Infect Dis(1985)[4047960]; JAMA(1985)[3871869]; Jpn J Antibiot(1985)[3937915]; Clin Ther(1985)[4075365]; Cutis(1985)[3876911]; Chest(1985)[3979135]; Chest(1985)[3877618]; Indian J Chest Dis Allied Sci(1985)[3879887]; Pediatr Infect Dis(1985)[3876546]; Br J Dis Chest(1985)[3872675]; Zh Mikrobiol Epidemiol Immunobiol(1985)[3874504]; Infection(1985)[3905621]; Jpn J Antibiot(1985)[3912526]; J Antimicrob Chemother(1985)[3922936]; Monatsschr Kinderheilkd(1985)[3889594]; J Infect(1985)[3891869]; Bull World Health Organ(1986)[3490924]; Infection(1986)[3759253]; Medicine (Baltimore)(1986)[3486337]; Infection(1986)[3514472]; N Z Med J(1986)[3486391]; Presse Med(1986)[2950405]; J Antimicrob Chemother(1986)[3011725]; Ann Intern Med(1986)[3484420]; Nihon Kyobu Shikkan Gakkai Zasshi(1986)[3494152]; Presse Med(1986)[2947130]; An Esp Pediatr(1986)[3706923]; J Am Geriatr Soc(1986)[3489749]; Clin Pediatr (Phila)(1986)[3490945]; Monatsschr Kinderheilkd(1986)[3748039]; Arch Dis Child(1986)[3488712]; Drugs(1986)[3488188]; Schweiz Med Wochenschr(1986)[3492762]; Scand J Infect Dis(1986)[3526532]; Tex Heart Inst J(1986)[15226859]; Jpn J Antibiot(1986)[3735662]; Br Med J (Clin Res Ed)(1986)[3084017]; Z Erkr Atmungsorgane(1986)[3532586]; J Antimicrob Chemother(1986)[3771434]; Scand J Infect Dis(1986)[3532303]; Rev Infect Dis(1986)[3529308]; Scand J Infect Dis(1987)[2447637]; Pharm Weekbl Sci(1987)[3438152]; Dis Mon(1987)[3026760]; J Clin Microbiol(1987)[3493260]; Zh Mikrobiol Epidemiol Immunobiol(1987)[3307240]; Rev Mal Respir(1987)[3671863]; Pediatr Emerg Care(1987)[3497386]; Infection(1987)[3301684]; Tidsskr Nor Laegeforen(1987)[3496683]; Am J Dis Child(1987)[3812407]; Zh Mikrobiol Epidemiol Immunobiol(1987)[3105204]; Infection(1987)[3610332]; Pediatrics(1987)[3496581]; Am J Epidemiol(1987)[3812456]; Rev Infect Dis(1987)[3122301]; Arch Intern Med(1987)[3492980]; Am J Med(1987)[3578359]; Pediatr Pulmonol(1987)[3498925]; Pharm Weekbl Sci(1987)[3438151]; Indiana Med(1987)[3494056]; Chemioterapia(1987)[3308147]; Med Care(1987)[3807447]; Thorax(1987)[3660311]; J Infect(1987)[3819455]; Pediatr Infect Dis J(1987)[3324038]; Zentralbl Bakteriol Mikrobiol Hyg A(1987)[3115004]; Nihon Rinsho(1987)[3110462]; Arch Fr Pediatr(1987)[3502237]; Pharm Weekbl Sci(1987)[3501857]; Thorax(1987)[3321545]; Bol Med Hosp Infant Mex(1987)[3322317]; Lancet(1988)[2896980]; Eur J Clin Microbiol Infect Dis(1988)[3134210]; J Infect Dis(1988)[2834457]; J Clin Microbiol(1988)[2454937]; J Gerontol(1988)[3121716]; Arch Dis Child(1988)[3196056]; Eur Respir J(1988)[3265667]; Pathol Biol (Paris)(1988)[3136431]; Kansenshogaku Zasshi(1988)[3138368]; Acta Paediatr Jpn(1988)[3150208]; Pediatriia(1988)[3264401]; APMIS(1988)[3126783]; Am Rev Respir Dis(1988)[3257853]; Hosp Formul(1988)[10314280]; Clin Chest Med(1988)[3044680]; Chest(1988)[3293939]; J Med Microbiol(1988)[3260287]; Helv Paediatr Acta(1988)[3262604]; Pediatr Emerg Care(1988)[3263628]; Pediatr Emerg Care(1988)[3231554]; J Infect Dis(1989)[2677160]; Med J Aust(1989)[2739613]; Pharm Weekbl Sci(1989)[2677980]; Zh Mikrobiol Epidemiol Immunobiol(1989)[2532836]; Am J Med(1989)[2686425]; Semin Respir Infect(1989)[2652232]; Taiwan Yi Xue Hui Za Zhi(1989)[2507737]; Eur J Pediatr(1989)[2591401]; Zh Mikrobiol Epidemiol Immunobiol(1989)[2785321]; An Esp Pediatr(1989)[2698068]; CMAJ(1989)[2642395]; J Antimicrob Chemother(1989)[2759923]; Eur J Clin Microbiol Infect Dis(1989)[2495944]; Rev Clin Esp(1989)[2506614]; Semin Respir Infect(1989)[2496450]; J Natl Med Assoc(1989)[2786083]; Arch Fr Pediatr(1989)[2786712]; Kansenshogaku Zasshi(1989)[2506294]; Arch Intern Med(1989)[2730252]; An Med Interna(1989)[2562732]; Nihon Kyobu Geka Gakkai Zasshi(1989)[2768924]; Arch Intern Med(1989)[2508586]; J Antimicrob Chemother(1989)[2501265]; J Clin Lab Immunol(1989)[2534928]; Trans R Soc Trop Med Hyg(1989)[2617633]; N Z Med J(1989)[2784846]; Clin Ther(1989)[2663160]; Rev Prat(1989)[2749147]; Nihon Kyobu Shikkan Gakkai Zasshi(1989)[2747072]; Am J Med(1989)[2783357]; Pediatr Infect Dis J(1989)[2812913]; Medicina (B Aires)(1989)[2698435]; West J Med(1989)[2603419]; J Infect Dis(1989)[2783717]; Am J Med(1989)[2690620]; Arch Intern Med(1990)[2244774]; Pediatrie(1990)[1963941]; Kekkaku(1990)[2077264]; Rev Cubana Med Trop(1990)[2259780]; Lancet(1990)[1967678]; Lancet(1990)[1968564]; Rev Infect Dis(1990)[2237109]; Antimicrob Agents Chemother(1990)[2127342]; Med J Aust(1990)[2255282]; Bull World Health Organ(1990)[2364476]; Am J Med(1990)[2346162]; Southeast Asian J Trop Med Public Health(1990)[2237586]; Thorax(1990)[2356552]; Anaesthesia(1990)[2386280]; Pediatr Infect Dis J(1990)[2352816]; Br Heart J(1990)[2375897]; Clin Pediatr (Phila)(1990)[2155085]; Kansenshogaku Zasshi(1990)[2335751]; Can Assoc Radiol J(1990)[2328430]; Ann Trop Paediatr(1990)[1694651]; Rev Infect Dis(1990)[2201066]; Med Clin North Am(1990)[2186247]; Pediatr Neurosurg(1990)[2133404]; J Infect Dis(1990)[2313130]; Rev Infect Dis(1990)[2270413]; Clin Pediatr (Phila)(1990)[2265514]; Kansenshogaku Zasshi(1990)[2121857]; Rev Infect Dis(1990)[2270412]; Rev Infect Dis(1990)[2270411]; Emerg Med Clin North Am(1990)[2187678]; Eur J Clin Microbiol Infect Dis(1990)[2303063]; Thorax(1990)[2330550]; Kansenshogaku Zasshi(1990)[2243193]; J Natl Med Assoc(1990)[2332910]; Enferm Infecc Microbiol Clin(1990)[2090241]; Pediatr Emerg Care(1991)[1788118]; Pediatr Emerg Care(1991)[1788120]; Thorax(1991)[1907034]; Infect Dis Clin North Am(1991)[1955696]; Rev Infect Dis(1991)[1862284]; P N G Med J(1991)[2058297]; P N G Med J(1991)[1750265]; Semin Respir Infect(1991)[1810003]; Ugeskr Laeger(1991)[2028541]; Pediatr Infect Dis J(1991)[2041665]; Clin Ther(1991)[1709390]; Semin Respir Infect(1991)[1809999]; J Hosp Infect(1991)[1684594]; Semin Respir Infect(1991)[1810000]; J Antimicrob Chemother(1991)[1778856]; AIDS(1991)[1777177]; Pediatr Ann(1991)[1945539]; Chemotherapy(1991)[1884650]; J Hosp Infect(1991)[1684601]; Ann Pediatr (Paris)(1991)[1746849]; J Thorac Imaging(1991)[1861271]; Med J Aust(1991)[1988801]; Pediatrics(1991)[1984618]; Infect Dis Clin North Am(1991)[1869812]; BMJ(1991)[1912835]; Pediatr Infect Dis J(1991)[2062611]; An Esp Pediatr(1991)[1793187]; J Thorac Imaging(1991)[1942196]; Schweiz Med Wochenschr Suppl(1991)[1853179]; P N G Med J(1991)[1750262]; J Paediatr Child Health(1992)[1389445]; Med Clin (Barc)(1992)[1434998]; Am Rev Respir Dis(1992)[1416398]; Union Med Can(1992)[1539396]; Postgrad Med J(1992)[1287613]; Rev Pneumol Clin(1992)[1439460]; J Infect Dis(1992)[1588175]; Ann Pediatr (Paris)(1992)[1456675]; Br J Hosp Med(1992)[1617342]; Pneumoftiziologia(1992)[1299394]; Rev Mal Respir(1992)[1589629]; Am J Med(1992)[1605137]; West J Med(1992)[1475945]; Med Clin (Barc)(1992)[1573921]; Am J Med(1992)[1605138]; Clin Infect Dis(1992)[1420692]; Respir Med(1992)[1470708]; J Antimicrob Chemother(1992)[1624392]; Nihon Kyobu Shikkan Gakkai Zasshi(1992)[1405100]; Respir Med(1992)[1565823]; Curr Med Res Opin(1992)[1633721]; Pneumoftiziologia(1992)[1299403]; Chest(1992)[1424890]; Ann Intern Med(1992)[1314530]; Pediatr Infect Dis J(1992)[1528640]; Nihon Kyobu Shikkan Gakkai Zasshi(1992)[1318432]; Tidsskr Nor Laegeforen(1992)[1412305]; Rev Clin Esp(1992)[1620956]; J Infect(1992)[1548416]; South Med J(1992)[1411720]; Diabetes Care(1992)[1468268]; Ann Saudi Med(1992)[17587050]; Diagn Microbiol Infect Dis(1992)[1730186]; Am Rev Respir Dis(1992)[1731595]; Kansenshogaku Zasshi(1992)[1331264]; Can J Infect Dis(1992)[22451757]; Gan To Kagaku Ryoho(1992)[1371046]; Arch Intern Med(1992)[1444688]; Kansenshogaku Zasshi(1992)[1431388]; Infect Control Hosp Epidemiol(1992)[1430999]; N Y State J Med(1992)[1518586]; Indian J Pediatr(1992)[1340860]; Kansenshogaku Zasshi(1992)[1624830]; Beitr Gerichtl Med(1992)[1489325]; An Med Interna(1992)[1581452]; Br Med Bull(1992)[1281036]; J Antimicrob Chemother(1993)[8226438]; Pediatrics(1993)[8424026]; Postgrad Med(1993)[8493197]; Ann Pharmacother(1993)[8453168]; JAMA(1993)[8417240]; J Trop Pediatr(1993)[8492372]; Presse Med(1993)[8378279]; Pharmacotherapy(1993)[8361868]; Minerva Chir(1993)[8177440]; Eur J Pediatr(1993)[8444202]; An Esp Pediatr(1993)[8239205]; J Antimicrob Chemother(1993)[8407696]; Kansenshogaku Zasshi(1993)[8360519]; Am J Med(1993)[8356990]; Clin Infect Dis(1993)[8286635]; Chin Med J (Engl)(1993)[8504692]; Thorax(1993)[8303628]; Kokyu To Junkan(1993)[8210746]; Lancet(1993)[8094769]; Enferm Infecc Microbiol Clin(1993)[8324033]; Kansenshogaku Zasshi(1993)[8254215]; Postgrad Med(1993)[8415335]; Semin Respir Infect(1993)[8372274]; Clin Infect Dis(1993)[8218679]; Drug Investig(1993)[32287509]; Rev Pneumol Clin(1993)[8296141]; Semin Respir Infect(1993)[8278678]; Enferm Infecc Microbiol Clin(1993)[8324023]; Intensive Care Med(1993)[8408937]; West Indian Med J(1993)[8160460]; Recenti Prog Med(1993)[8488331]; Chest(1993)[8404198]; Med J Aust(1993)[8487685]; Rev Inst Med Trop Sao Paulo(1994)[7855490]; Eur Respir J(1994)[8162975]; Chest(1994)[8181342]; Eur Respir J(1994)[8143807]; Acta Paediatr Jpn(1994)[8203272]; Semin Respir Infect(1994)[7831538]; J Chemother(1994)[7861196]; Am J Epidemiol(1994)[8178792]; Southeast Asian J Trop Med Public Health(1994)[7667711]; Acta Paediatr(1994)[7981562]; Kansenshogaku Zasshi(1994)[7876669]; J Infect(1994)[7884221]; Zh Mikrobiol Epidemiol Immunobiol(1994)[8017127]; Eur J Clin Microbiol Infect Dis(1994)[7813492]; Ryoikibetsu Shokogun Shirizu(1994)[8152126]; Clin Infect Dis(1994)[8086559]; Scand J Infect Dis Suppl(1994)[8047855]; J Qual Clin Pract(1994)[8199754]; Semin Respir Infect(1994)[7831539]; Southeast Asian J Trop Med Public Health(1994)[7667713]; Mil Med(1994)[7870317]; Southeast Asian J Trop Med Public Health(1994)[7825002]; Clin Infect Dis(1994)[7803647]; Ann Trop Paediatr(1994)[7521637]; J Med Assoc Thai(1994)[7759965]; J Pediatr (Rio J)(1994)[14688857]; Enferm Infecc Microbiol Clin(1994)[8155750]; J Antimicrob Chemother(1994)[7829419]; East Afr Med J(1994)[7925039]; Kansenshogaku Zasshi(1994)[7996025]; Presse Med(1994)[7937643]; Arch Intern Med(1994)[8092913]; Kansenshogaku Zasshi(1994)[7829904]; Chest(1994)[8020263]; Rev Pneumol Clin(1994)[7839051]; Pediatr Infect Dis J(1994)[8190537]; Thorax(1995)[7597657]; Arch Bronconeumol(1995)[7743064]; Thorax(1995)[7597669]; Neth J Med(1995)[7643944]; Cutis(1995)[7729153]; Infection(1995)[8537133]; Eur Respir J(1995)[7789484]; Acta Paediatr(1995)[7756803]; Transplantation(1995)[7570975]; Am J Respir Crit Care Med(1995)[7551388]; Presse Med(1995)[7494845]; J Chemother(1995)[8568541]; Kansenshogaku Zasshi(1995)[8708405]; Rev Mal Respir(1995)[7899666]; Eur Respir J(1995)[8575582]; J Otolaryngol(1995)[8551540]; J Infect Dis(1995)[7594672]; J Antimicrob Chemother(1995)[8543488]; Ann Ig(1995)[8679163]; Clin Infect Dis(1995)[7795085]; J Infect Dis(1995)[7594685]; Rev Latinoam Microbiol(1995)[8850337]; Epidemiol Infect(1995)[7641841]; Thorax(1995)[7660337]; Monaldi Arch Chest Dis(1995)[8541820]; Kansenshogaku Zasshi(1995)[7751728]; Rev Mal Respir(1995)[7481050]; Rev Clin Esp(1995)[7569208]; Med Trop (Mars)(1995)[7637608]; Praxis (Bern 1994)(1995)[7481320]; Microb Drug Resist(1995)[9158749]; Infect Dis Obstet Gynecol(1995)[18475411]; J Egypt Public Health Assoc(1995)[17214208]; Thorax(1995)[8553294]; Can J Infect Dis(1995)[22550412]; Chest(1995)[7555163]; Chest(1995)[7497775]; Clin Infect Dis(1995)[8547517]; Med J Aust(1995)[7565207]; Presse Med(1995)[7899346]; Rev Mal Respir(1996)[8711237]; J Infect Dis(1996)[8940231]; Clin Infect Dis(1996)[8783712]; Clin Infect Dis(1996)[8816138]; Chemotherapy(1996)[8983892]; J Antimicrob Chemother(1996)[8961060]; Enferm Infecc Microbiol Clin(1996)[9011211]; J Formos Med Assoc(1996)[8870429]; Semin Respir Infect(1996)[8776779]; Semin Respir Infect(1996)[8776781]; Nihon Kyobu Shikkan Gakkai Zasshi(1996)[8810756]; Antimicrob Agents Chemother(1996)[8723461]; Clin Infect Dis(1996)[8783710]; Diagn Microbiol Infect Dis(1996)[8937842]; Clin Infect Dis(1996)[8922805]; Ann Acad Med Singap(1996)[8799002]; Pediatr Pulmonol(1996)[8893261]; Infez Med(1996)[14978374]; Clin Microbiol Infect(1996)[11866796]; Medscape Womens Health(1996)[9746716]; Int J Antimicrob Agents(1996)[18611723]; Enferm Infecc Microbiol Clin(1996)[8695681]; Arch Pediatr(1996)[8952771]; Semin Respir Infect(1996)[8883171]; Arch Bronconeumol(1996)[8634791]; Singapore Med J(1996)[8993135]; Clin Infect Dis(1996)[8824970]; Rev Prat(1996)[8978166]; Chest(1996)[8874254]; Laeknabladid(1996)[20065391]; Kansenshogaku Zasshi(1996)[8699095]; Pediatr Infect Dis J(1997)[9041619]; MMWR Morb Mortal Wkly Rep(1997)[9026713]; Postgrad Med(1997)[9046935]; Heart Lung(1997)[9013224]; Br J Oral Maxillofac Surg(1997)[9043003]; Commun Dis Intell(1997)[9222159]; Can Commun Dis Rep(1997)[9190089]; Scand J Infect Dis(1997)[9360255]; Ann Trop Paediatr(1997)[9578790]; Acta Clin Belg(1997)[9351291]; Pediatr Infect Dis J(1997)[9271041]; Changgeng Yi Xue Za Zhi(1997)[9397619]; Acta Med Port(1997)[9235847]; Antibiot Khimioter(1997)[9412402]; Kansenshogaku Zasshi(1997)[9339624]; Respiration(1997)[9154675]; Lancet(1997)[9130934]; Presse Med(1997)[9082404]; J Pediatr(1997)[9202614]; J Chemother(1997)[9248976]; Aust N Z J Med(1997)[9079255]; Antibiot Khimioter(1997)[9412400]; J Chemother(1997)[9248975]; Clin Infect Dis(1997)[9332511]; Jpn J Antibiot(1997)[9394237]; Diagn Microbiol Infect Dis(1997)[9458982]; Curr Opin Pulm Med(1997)[9193863]; Presse Med(1997)[9452759]; Enferm Infecc Microbiol Clin(1997)[9527370]; J Antimicrob Chemother(1997)[9145823]; Acta Med Austriaca(1997)[9206930]; Semin Perinatol(1997)[9298723]; J Med Microbiol(1997)[9379469]; J Infect(1997)[9120323]; Nihon Rinsho(1997)[9360392]; JAMA(1997)[9039868]; Enferm Infecc Microbiol Clin(1997)[9410082]; Kekkaku(1997)[9259127]; Schweiz Med Wochenschr(1997)[9446191]; Pediatr Infect Dis J(1997)[9076838]; Arch Dis Child(1997)[9166026]; Pediatr Infect Dis J(1998)[9727654]; An Esp Pediatr(1998)[9972622]; Int J Antimicrob Agents(1998)[9832282]; Chest(1998)[9596294]; Vaccine(1998)[9711807]; Eur J Clin Microbiol Infect Dis(1998)[9865980]; Intern Med(1998)[9840716]; Semin Respir Infect(1998)[9543475]; Pediatr Infect Dis J(1998)[9535250]; Med Clin (Barc)(1998)[9717161]; MMWR Morb Mortal Wkly Rep(1998)[9746431]; J Radiol(1998)[9791768]; Arch Pediatr(1998)[10223154]; Arch Pediatr(1998)[10223157]; J Commun Dis(1998)[10810561]; Med J Aust(1998)[9577450]; Presse Med(1998)[9767770]; J Antimicrob Chemother(1998)[9533462]; Arch Pediatr(1998)[10223155]; Pediatr Infect Dis J(1998)[9802641]; Antimicrob Agents Chemother(1998)[9559773]; Int J Antimicrob Agents(1998)[9716289]; J Trop Pediatr(1998)[9972076]; Med J Aust(1998)[9577446]; EPI Newsl(1998)[12293780]; Clin Infect Dis(1998)[9564484]; Presse Med(1998)[9798478]; Int J Tuberc Lung Dis(1998)[9562104]; Curr Opin Pulm Med(1998)[9675520]; Wkly Epidemiol Rec(1998)[9542461]; Pathol Biol (Paris)(1998)[9769865]; Arch Pediatr(1998)[10223104]; Pediatr Infect Dis J(1998)[9469390]; Am J Respir Crit Care Med(1998)[9603129]; Chest(1998)[9872193]; J Trop Pediatr(1998)[9972077]; Pediatr Infect Dis J(1998)[9781764]; Zh Mikrobiol Epidemiol Immunobiol(1998)[9783407]; Presse Med(1998)[9793046]; Pediatr Infect Dis J(1998)[9781754]; Pneumoftiziologia(1998)[10386145]; Chest(1998)[9631791]; Bull World Health Organ(1998)[9615503]; Pediatr Infect Dis J(1998)[9781744]; Pediatr Infect Dis J(1998)[9849979]; Pediatr Infect Dis J(1998)[9781753]; Pediatr Infect Dis J(1998)[9781761]; Pediatr Infect Dis J(1998)[9781751]; Ann Surg(1998)[9605666]; Pneumologie(1998)[9885511]; Pediatr Infect Dis J(1998)[9781758]; Trop Med Int Health(1998)[9593359]; Kansenshogaku Zasshi(1998)[9545687]; Int J Circumpolar Health(1999)[10615827]; Monaldi Arch Chest Dis(1999)[10546478]; Curr Opin Infect Dis(1999)[17035771]; Antibiot Khimioter(1999)[10095919]; Clin Infect Dis(1999)[10064229]; J Antimicrob Chemother(1999)[10590275]; Am J Trop Med Hyg(1999)[10403339]; Compr Ther(1999)[9987589]; Indian J Pediatr(1999)[10798147]; J Okla State Med Assoc(1999)[10363435]; J Microbiol Immunol Infect(1999)[10650490]; Kansenshogaku Zasshi(1999)[10077904]; Int J Tuberc Lung Dis(1999)[10488880]; Sante(1999)[10477402]; Curr Med Res Opin(1999)[10640263]; Curr Opin Infect Dis(1999)[17035770]; Infection(1999)[10885840]; Singapore Med J(1999)[10414167]; Antimicrob Agents Chemother(1999)[9925540]; Bull World Health Organ(1999)[10612883]; Paediatr Anaesth(1999)[10597562]; J Antimicrob Chemother(1999)[10225579]; Indian Pediatr(1999)[10745313]; S Afr Med J(1999)[10554623]; Semin Respir Infect(1999)[10638512]; Am J Respir Crit Care Med(1999)[10430704]; Int J Pediatr Otorhinolaryngol(1999)[10577771]; EPI Newsl(1999)[12349088]; Am J Health Syst Pharm(1999)[10580734]; J Chemother(1999)[10435684]; Can Respir J(1999)[10202230]; Zhonghua Jie He He Hu Xi Za Zhi(1999)[11775913]; Pediatr Infect Dis J(1999)[10608624]; Clin Infect Dis(1999)[10064223]; MMW Fortschr Med(1999)[10912172]; Int J Infect Dis(1999)[10460927]; Pharmacoeconomics(1999)[10345159]; Rev Esp Quimioter(1999)[10855018]; Can J Infect Dis(1999)[22346393]; Aten Primaria(1999)[10341462]; Eur J Pediatr Surg(1999)[10207695]; Can J Infect Dis(1999)[22346378]; Antibiot Khimioter(1999)[10511903]; Clin Chest Med(1999)[10516899]; Am J Med(1999)[10225239]; Chest(1999)[9925081]; Kansenshogaku Zasshi(1999)[10356891]; Pediatr Infect Dis J(1999)[10530574]; Paediatr Drugs(1999)[10937477]; Postgrad Med(1999)[10223091]; Braz J Infect Dis(1999)[11097714]; Pediatr Clin North Am(1999)[10629677]; Ryoikibetsu Shokogun Shirizu(1999)[10088420]; Med Clin (Barc)(1999)[10027178]; Eur J Clin Microbiol Infect Dis(1999)[10691195]; Schweiz Med Wochenschr(1999)[10087589]; Med Klin (Munich)(1999)[10603732]; Chest(1999)[10424501]; Rev Esp Quimioter(1999)[10878531]; Neth J Med(1999)[10509069]; Presse Med(1999)[10506878]; Semin Respir Infect(1999)[10391411]; Chemotherapy(2000)[10810209]; Chest(2000)[11083685]; Trop Med Int Health(2000)[11044266]; Am J Obstet Gynecol(2000)[10920320]; Semin Respir Infect(2000)[11052424]; Am J Trop Med Hyg(2000)[11037775]; Arch Pediatr(2000)[10941479]; Southeast Asian J Trop Med Public Health(2000)[11414406]; Infection(2000)[11139153]; Pediatr Infect Dis J(2000)[10819340]; Respir Med(2000)[10714413]; Int J Antimicrob Agents(2000)[10926447]; J Health Popul Nutr(2000)[11262765]; Clin Microbiol Infect(2000)[11168105]; J Paediatr Child Health(2000)[10760009]; Mil Med(2000)[10920635]; Am J Respir Crit Care Med(2000)[10806182]; J Chemother(2000)[11131955]; Diagn Microbiol Infect Dis(2000)[10863106]; Rev Panam Salud Publica(2000)[10893974]; Tidsskr Nor Laegeforen(2000)[10851938]; Eur J Clin Microbiol Infect Dis(2000)[11205629]; J Antimicrob Chemother(2000)[10747822]; Przegl Epidemiol(2000)[11349588]; Aten Primaria(2000)[10917693]; Tunis Med(2000)[11026821]; Rev Panam Salud Publica(2000)[11036429]; Diagn Microbiol Infect Dis(2000)[10729663]; Kansenshogaku Zasshi(2000)[11140078]; Clin Infect Dis(2000)[10913417]; Int J Antimicrob Agents(2000)[11137405]; J Antimicrob Chemother(2000)[10933659]; Semin Respir Crit Care Med(2000)[16088723]; Postgrad Med(2000)[19667546]; Emerg Infect Dis(2000)[10756150]; J Paediatr Child Health(2000)[10940162]; Chest(2000)[10767233]; J Crit Care(2000)[11011820]; Chest(2000)[11115444]; Infez Med(2000)[12709603]; Clin Microbiol Rev(2000)[10756001]; J Trop Pediatr(2000)[11191142]; Arch Bronconeumol(2000)[10932342]; Semin Respir Crit Care Med(2000)[16088722]; Paediatr Respir Rev(2000)[16263438]; Kansenshogaku Zasshi(2000)[10695294]; P N G Med J(2000)[11407623]; Rev Pneumol Clin(2000)[11033531]; Crit Care Med(2000)[11057812]; Clin Infect Dis(2000)[10875784]; Arch Intern Med(2000)[10809032]; An Esp Pediatr(2000)[11003900]; Braz J Infect Dis(2000)[11063556]; Eur J Pediatr(2000)[11014469]; Can J Infect Dis(2000)[18159274]; Acta Diabetol(2001)[11757805]; Jpn J Antibiot(2001)[12638153]; Clin Infect Dis(2001)[11320449]; Pediatr Infect Dis J(2001)[11303834]; Ther Umsch(2001)[11695094]; Braz J Infect Dis(2001)[11493414]; Rev Pneumol Clin(2001)[11593157]; J Trop Pediatr(2001)[11827304]; Ther Umsch(2001)[11695093]; Tunis Med(2001)[11771431]; Rev Pneumol Clin(2001)[11353919]; An Esp Pediatr(2001)[11181215]; Ann Pharmacother(2001)[11197583]; Microb Drug Resist(2001)[11822780]; Medicine (Baltimore)(2001)[11307588]; Ther Umsch(2001)[11695092]; ANZ J Surg(2001)[11906390]; J Health Popul Nutr(2001)[11855348]; Am J Trop Med Hyg(2001)[11442214]; Jpn J Antibiot(2001)[12638145]; Braz J Infect Dis(2001)[11980591]; Indian J Chest Dis Allied Sci(2001)[11370501]; Med J Malaysia(2001)[11732071]; Med Clin (Barc)(2001)[11412622]; Clin Infect Dis(2001)[11112673]; Manag Care Interface(2001)[11339025]; Respir Med(2001)[11419672]; Acta Med Port(2001)[11878155]; Thorax(2001)[11254821]; Am J Med(2001)[11755437]; Med Clin (Barc)(2001)[11734171]; Crit Care(2001)[11353934]; Indian J Pediatr(2001)[11563252]; Southeast Asian J Trop Med Public Health(2001)[11944709]; J Laryngol Otol(2001)[11564297]; Am J Med(2001)[11755439]; Crit Care Med(2001)[11246310]; Tunis Med(2001)[11910687]; Am J Med(2001)[11755440]; Am J Respir Crit Care Med(2001)[11254518]; Pediatr Infect Dis J(2001)[11176567]; Diagn Microbiol Infect Dis(2001)[11248523]; N Z Med J(2001)[11797872]; Indian J Chest Dis Allied Sci(2001)[11529434]; Nihon Ronen Igakkai Zasshi(2001)[11431879]; Kansenshogaku Zasshi(2001)[11218385]; Respirology(2001)[11555386]; Intern Med(2001)[11518106]; Clin Infect Dis(2001)[11283803]; Arch Pediatr(2001)[11582920]; Am J Med(2001)[11755441]; Thorax(2001)[11209098]; Arch Dis Child(2001)[11159294]; Pediatr Infect Dis J(2002)[12182396]; J Fam Pract(2002)[12184969]; J Microbiol Immunol Infect(2002)[12099332]; P N G Med J(2002)[14658827]; N Engl J Med(2002)[12181400]; Pharmacoeconomics(2002)[12109917]; Southeast Asian J Trop Med Public Health(2002)[12118460]; J Med Microbiol(2002)[11990492]; Zh Mikrobiol Epidemiol Immunobiol(2002)[12449700]; S Afr Med J(2002)[12506595]; Geriatrics(2002)[11899549]; Vaccine(2002)[12126912]; Pediatr Infect Dis J(2002)[12182397]; Pediatr Infect Dis J(2002)[12075755]; No To Shinkei(2002)[12058414]; J Chemother(2002)[12017369]; Acta Paediatr(2002)[11951998]; Rinsho Byori(2002)[12187704]; Nihon Kokyuki Gakkai Zasshi(2002)[12645113]; Curr Opin Infect Dis(2002)[11964917]; Ned Tijdschr Geneeskd(2002)[12051061]; Neth J Med(2002)[12164371]; Acta Paediatr(2002)[11951994]; Clin Infect Dis(2002)[12410480]; Ned Tijdschr Geneeskd(2002)[11802335]; Nihon Rinsho(2002)[11838187]; Diagn Microbiol Infect Dis(2002)[12376039]; J Chemother(2002)[12583551]; Ann Pharmacother(2002)[12243604]; Int J Antimicrob Agents(2002)[11850165]; Recenti Prog Med(2002)[12355983]; J Pediatr (Rio J)(2002)[14676852]; Clin Ther(2002)[12501878]; Crit Care Med(2002)[12163786]; Int J Infect Dis(2002)[12718823]; Rev Med Chil(2002)[12611238]; Ther Umsch(2002)[11851042]; Southeast Asian J Trop Med Public Health(2002)[12236437]; J Antimicrob Chemother(2002)[12556430]; Pharmacoepidemiol Drug Saf(2002)[11998547]; J Antimicrob Chemother(2002)[12239229]; Int J Antimicrob Agents(2002)[12007844]; J Clin Microbiol(2003)[12904354]; Clin Infect Dis(2003)[12955648]; Diagn Microbiol Infect Dis(2003)[12729995]; Clin Infect Dis(2003)[14689337]; Clin Cornerstone(2003)[14992415]; Braz J Infect Dis(2003)[12807692]; Semin Respir Crit Care Med(2003)[16088583]; Antimicrob Agents Chemother(2003)[12760850]; J Chemother(2003)[12868547]; Emerg Infect Dis(2003)[14718097]; Rev Panam Salud Publica(2003)[14769154]; Nihon Kokyuki Gakkai Zasshi(2003)[14727547]; Am J Respir Med(2003)[14720021]; Biomedica(2003)[12872558]; Zhonghua Yi Xue Za Zhi(2003)[12820915]; Pediatr Pulmonol(2003)[14618637]; Rev Prat(2003)[14558265]; Medicina (Kaunas)(2003)[12695633]; J Antimicrob Chemother(2003)[14613949]; J Antimicrob Chemother(2003)[14585865]; Diagn Microbiol Infect Dis(2003)[12730000]; FEMS Immunol Med Microbiol(2003)[12770762]; Ann Allergy Asthma Immunol(2003)[14692436]; East Afr Med J(2003)[12918806]; Vestn Ross Akad Med Nauk(2003)[12861708]; Eur J Intern Med(2003)[14962701]; Eur J Clin Microbiol Infect Dis(2003)[12942342]; J Microbiol Immunol Infect(2003)[12741738]; Clin Microbiol Infect(2003)[14686999]; J Trop Pediatr(2003)[12630717]; South Med J(2003)[14570341]; Arch Bronconeumol(2003)[12975069]; Braz J Infect Dis(2003)[12807691]; Nihon Rinsho(2003)[12722254]; Medicina (Kaunas)(2003)[12695638]; Pol Merkur Lekarski(2003)[12712818]; J Clin Pathol(2003)[12719453]; Diagn Microbiol Infect Dis(2003)[12944021]; J Antimicrob Chemother(2003)[12697646]; Int J Med Microbiol(2003)[14503795]; Vaccine(2003)[12706676]; Respirology(2003)[12856747]; Rev Port Pneumol(2003)[19771688]; Chest(2003)[12527619]; Respir Med(2003)[12854626]; Kansenshogaku Zasshi(2003)[14515753]; Acta Paediatr(2003)[14632332]; Pediatr Pulmonol(2003)[12772225]; Cad Saude Publica(2003)[14666208]; Chest(2003)[12853512]; Pediatr Med Chir(2003)[12916441]; Arch Bronconeumol(2003)[12890400]; Arch Pediatr Adolesc Med(2003)[12695236]; Nihon Rinsho(2003)[14619435]; Ann Fr Anesth Reanim(2004)[15030862]; Emerg Infect Dis(2004)[15324548]; Pediatr Pulmonol(2004)[15211697]; Int J Antimicrob Agents(2004)[15194123]; J Chemother(2004)[15690685]; Pac Health Dialog(2004)[18181445]; Respir Med(2004)[15481271]; Zhonghua Jie He He Hu Xi Za Zhi(2004)[14989822]; Respirology(2004)[15182283]; Semin Pediatr Infect Dis(2004)[15480964]; Clin Infect Dis(2004)[15156444]; In Vivo(2004)[15011754]; Pulm Pharmacol Ther(2004)[15219264]; J Trop Pediatr(2004)[15357563]; Vaccine(2004)[15161074]; Zh Mikrobiol Epidemiol Immunobiol(2004)[15636151]; Antibiot Khimioter(2004)[15727145]; Zhonghua Jie He He Hu Xi Za Zhi(2004)[15130324]; Eur Radiol(2004)[14749949]; Pediatr Infect Dis J(2004)[15545867]; Antibiot Khimioter(2004)[16050495]; Chemotherapy(2004)[15272227]; Int J Epidemiol(2004)[15075166]; Zhonghua Er Ke Za Zhi(2004)[15631716]; J Infect(2004)[14667792]; Scand J Infect Dis(2004)[15198183]; Int J Antimicrob Agents(2004)[15164972]; Antibiot Khimioter(2004)[15344392]; Am Fam Physician(2004)[15368729]; J Infect Chemother(2004)[15614461]; J Paediatr Child Health(2004)[15265191]; Int J Antimicrob Agents(2004)[14732312]; J Trauma(2004)[14960971]; Am J Med(2004)[15360097]; Southeast Asian J Trop Med Public Health(2004)[15691151]; Br J Gen Pract(2004)[14965401]; BMJ(2004)[15070633]; J Infect(2004)[14667791]; Am J Manag Care(2004)[15521160]; Vaccine(2004)[15519705]; Pediatr Infect Dis J(2004)[15361724]; Curr Opin Pulm Med(2004)[15071368]; Chang Gung Med J(2004)[15095957]; Lancet(2005)[15643700]; Ned Tijdschr Geneeskd(2005)[16171106]; Rev Chilena Infectol(2005)[16163415]; Scand J Infect Dis(2005)[16308233]; Rev Chilena Infectol(20 |
| *Haemophilus parainfluenzae* | Established pathogen | 53 | **established** | Am J Med Sci(1974)[4841905]; Lancet(1976)[73690]; Scand J Infect Dis(1977)[198875]; Am Rev Respir Dis(1979)[316298]; Arch Intern Med(1979)[475536]; Arch Intern Med(1980)[6966913]; Arch Intern Med(1981)[7235817]; Pathol Biol (Paris)(1983)[6341938]; Respiration(1984)[6395241]; Postgrad Med(1984)[6371752]; Nihon Kyobu Shikkan Gakkai Zasshi(1984)[6333541]; Br Med J (Clin Res Ed)(1985)[2994802]; Infection(1985)[3872845]; Jpn J Antibiot(1985)[3912526]; Chemioterapia(1987)[3308147]; Eur J Clin Microbiol Infect Dis(1988)[3134210]; J Infect Dis(1989)[2677160]; Trans R Soc Trop Med Hyg(1989)[2617633]; Rev Infect Dis(1991)[1925278]; Rev Chil Pediatr(1991)[1844925]; Postgrad Med J(1992)[1287613]; S Afr Med J(1992)[1585219]; Presse Med(1993)[8316546]; Eur Respir J(1995)[7789484]; Przegl Epidemiol(1995)[7676055]; Enferm Infecc Microbiol Clin(1996)[8695681]; Int J Antimicrob Agents(1998)[9832282]; Thorax(2000)[10856326]; J Microbiol Immunol Infect(2002)[11950124]; Recenti Prog Med(2002)[12355983]; Antimicrob Agents Chemother(2003)[14638520]; Int J Antimicrob Agents(2004)[15164972]; In Vivo(2004)[15011754]; Southeast Asian J Trop Med Public Health(2005)[16438155]; Int J Antimicrob Agents(2005)[15967638]; Pneumologia(2007)[17491203]; Zhongguo Dang Dai Er Ke Za Zhi(2008)[18947473]; Int J Tuberc Lung Dis(2012)[22236856]; J Med Case Rep(2012)[22546325]; Zhonghua Yu Fang Yi Xue Za Zhi(2013)[24113096]; Chin Med J (Engl)(2013)[23786927]; Presse Med(2014)[25451635]; PLoS One(2014)[25033402]; Can J Infect Dis Med Microbiol(2016)[27516778]; Hum Vaccin Immunother(2017)[28922613]; Zhonghua Jie He He Hu Xi Za Zhi(2019)[30955282]; Microorganisms(2019)[31600928]; Microbiol Resour Announc(2020)[32217675]; Travel Med Infect Dis(2020)[32305630]; Eur J Clin Microbiol Infect Dis(2020)[31828685]; Iowa Orthop J(2020)[32742217]; BMC Fam Pract(2021)[33957884]; Transpl Infect Dis(2021)[33523538] |
| *Klebsiella aerogenes* | Unlikely patho, no or very few evidence | 0 | **established** | NA |
| *Klebsiella michiganensis* | Unlikely patho, no or very few evidence | 0 | **established** | NA |
| *Klebsiella oxytoca* | Possible patho, intermediate # of evidence | 49 | **established** | Jpn J Antibiot(1980)[7241797]; Thorax(1983)[6857585]; Rev Infect Dis(1985)[3890094]; No To Shinkei(1988)[3293638]; Scand J Infect Dis(1991)[1957125]; Jpn J Antibiot(1991)[1920813]; South Med J(1991)[1990453]; Acta Anaesthesiol Scand(1992)[1574979]; Chest(1993)[8339664]; Dtsch Med Wochenschr(1995)[7607059]; QJM(1996)[9015487]; Presse Med(1996)[8958855]; Clin Infect Dis(1998)[9524863]; Infect Control Hosp Epidemiol(1999)[10064219]; Enferm Infecc Microbiol Clin(2001)[11709131]; Clin Ter(2004)[15700634]; J Infect Chemother(2005)[15729490]; Endoscopy(2007)[17957611]; J Clin Microbiol(2007)[17715376]; Infect Control Hosp Epidemiol(2008)[18947320]; J Pediatr Hematol Oncol(2008)[18799945]; J Infect Chemother(2011)[20862506]; Clin Microbiol Infect(2011)[20345467]; Pneumonol Alergol Pol(2012)[22370984]; Infect Control Hosp Epidemiol(2013)[23221186]; J Orthop Surg (Hong Kong)(2013)[23629998]; Diagn Microbiol Infect Dis(2014)[24582578]; Case Rep Infect Dis(2014)[25405043]; Transplantation(2014)[24162251]; Antimicrob Agents Chemother(2015)[25348541]; J Clin Diagn Res(2016)[27790429]; Transplant Proc(2016)[26915861]; Balkan Med J(2016)[27994915]; Ann Burns Fire Disasters(2017)[29983683]; Rev Peru Med Exp Salud Publica(2017)[29267766]; Zhonghua Yu Fang Yi Xue Za Zhi(2017)[29036990]; Infect Control Hosp Epidemiol(2018)[29249216]; Ann Clin Microbiol Antimicrob(2018)[29571291]; Acta Paediatr(2019)[30238492]; J Coll Physicians Surg Pak(2019)[31455486]; AIMS Public Health(2020)[33294483]; Wilderness Environ Med(2020)[32739040]; JAC Antimicrob Resist(2020)[34223023]; Mod Rheumatol Case Rep(2020)[33087003]; PLoS One(2021)[34255801]; Respir Med Case Rep(2021)[34401302]; J Nepal Health Res Counc(2021)[33510506]; J Med Case Rep(2021)[33536050]; BMJ Case Rep(2021)[34326118] |
| *Klebsiella pneumoniae* | Established pathogen | 1501 | **established** | Perm Found Med Bull(1946)[20275564]; J Am Med Assoc(1946)[20987431]; Med J Aust(1947)[20271257]; Treat Serv Bull(1947)[20261713]; Radiology(1948)[18917095]; Cinci J Med(1948)[18866043]; N Y State J Med(1948)[18872053]; Am J Dis Child (1911)(1949)[18131770]; J Pediatr(1949)[18152892]; N Y State J Med(1949)[18127866]; J Am Med Assoc(1950)[15436283]; Orv Hetil(1952)[13003342]; AMA Arch Intern Med(1952)[14902153]; Ned Tijdschr Geneeskd(1953)[13144810]; N C Med J(1953)[13111614]; Prensa Pediatr Rev Am Puericu Pediatr(1953)[13155434]; Arch Fr Pediatr(1953)[13093012]; Am J Med Sci(1954)[13180507]; Ther Hung(1954)[13247269]; Orv Hetil(1954)[13214862]; Mem Acad Chir (Paris)(1954)[13165004]; Arch Fr Pediatr(1954)[13159414]; Ann Med Intern Fenn(1954)[13158982]; N Engl J Med(1955)[13244811]; Antibiot Annu(1955)[13355364]; N Engl J Med(1955)[14383955]; Mem Acad Chir (Paris)(1955)[13369121]; Mem Acad Chir (Paris)(1955)[13369113]; Pediatr Pol(1955)[14394730]; Arch Dis Child(1956)[13314667]; Dis Chest(1956)[13356731]; Acta Med Acad Sci Hung(1956)[13394112]; Orv Hetil(1956)[13335188]; Glas Belgrad Hig Inst NR Srb(1957)[13524679]; Neurology(1957)[13451886]; Med Interna (Bucur)(1957)[13516065]; Med Interna (Bucur)(1957)[13450882]; Acta Clin Belg(1957)[13457737]; Riforma Med(1957)[13528569]; Mo Med(1957)[13430592]; Riforma Med(1959)[13840926]; Pediatrie(1959)[13633384]; Med Interna (Bucur)(1961)[13899648]; Antibiotiki(1961)[13789056]; Pediatrics(1962)[13920487]; Sem Hop(1963)[14101759]; Postgrad Med J(1963)[14053697]; Dis Chest(1963)[13950138]; Am J Surg(1963)[14017451]; Acta Chir Scand(1964)[14171727]; Boll Ist Sieroter Milan(1964)[14180470]; Arch Intern Med(1964)[14109017]; Arch Pediatr Urug(1965)[5896854]; Antibiotiki(1967)[4386255]; G Mal Infett Parassit(1967)[4387648]; Rev Ecuat Hig Med Trop(1968)[5720576]; Bull Soc Med Afr Noire Lang Fr(1968)[5713784]; Acta Paediatr Acad Sci Hung(1968)[5728707]; N Z Med J(1969)[5258884]; Ann Intern Med(1969)[4388922]; Antibiotiki(1969)[4391893]; Pediatr Pol(1970)[4992308]; Z Erkr Atmungsorgane Folia Bronchol(1970)[4399837]; Minerva Med(1970)[5425724]; J Pediatr(1971)[5539786]; Vestn Rentgenol Radiol(1971)[4949629]; Z Erkr Atmungsorgane Folia Bronchol(1971)[4399185]; Am J Med Sci(1972)[4486504]; Med J Aust(1972)[4404399]; Chest(1973)[4571978]; Can Med Assoc J(1973)[4147709]; Dtsch Med Wochenschr(1973)[4570869]; Environ Lett(1973)[4568795]; Can Med Assoc J(1973)[4586073]; Fortschr Geb Rontgenstr Nuklearmed(1974)[4369476]; Pol Tyg Lek(1974)[4822188]; Chest(1975)[1149508]; Lab Delo(1975)[45963]; Dis Mon(1975)[236155]; Jpn J Antibiot(1975)[239263]; Can Med Assoc J(1975)[1089466]; Anasthesiol Intensivmed Prax(1975)[773205]; N Y State Dent J(1976)[772504]; Vnitr Lek(1976)[4922]; Bull Int Union Tuberc(1976)[31959]; Vnitr Lek(1976)[772954]; An Esp Pediatr(1976)[193420]; JAMA(1977)[17018]; Laryngoscope(1977)[318724]; J Fam Pract(1977)[320285]; S Afr Med J(1977)[327583]; Thorax(1977)[341405]; Am J Med(1977)[871128]; J Int Med Res(1978)[357229]; Am J Med(1978)[341702]; Scand J Infect Dis(1978)[725540]; Am J Dis Child(1979)[382838]; Gastroenterology(1979)[369935]; Ned Tijdschr Geneeskd(1979)[386143]; J R Coll Physicians Lond(1979)[33268]; J Ky Med Assoc(1979)[393786]; Nurs Mirror(1979)[377248]; Prim Care(1979)[392574]; Pneumonol Pol(1979)[119951]; Clin Notes Respir Dis(1979)[394893]; J Pediatr(1979)[758385]; P N G Med J(1980)[7008428]; Radiol Med(1980)[7005983]; Pediatrics(1980)[6990374]; Jpn J Antibiot(1980)[7001088]; Lab Delo(1980)[6155526]; J Am Geriatr Soc(1980)[6993540]; ZFA (Stuttgart)(1980)[6998163]; Am J Trop Med Hyg(1980)[6986096]; Chemotherapy(1981)[7249798]; MMW Munch Med Wochenschr(1981)[6785603]; Rev Ig Bacteriol Virusol Parazitol Epidemiol Pneumoftiziol Pneumoftiziol(1981)[6278566]; Ter Arkh(1981)[7027504]; Thorax(1981)[7314031]; Klin Med (Mosk)(1981)[7024630]; Chest(1981)[7226908]; Compr Ther(1981)[7018827]; Zh Mikrobiol Epidemiol Immunobiol(1982)[6755984]; Ann Fr Anesth Reanim(1982)[6224444]; Anaesthesist(1982)[7091639]; Jpn J Antibiot(1982)[6306293]; Jpn J Antibiot(1982)[6296478]; Vox Sang(1983)[6340354]; Rev Infect Dis(1983)[6828811]; Jpn J Antibiot(1983)[6425535]; Isr J Med Sci(1983)[6662687]; Eur J Respir Dis(1983)[6825749]; Am J Infect Control(1983)[6349427]; Am Rev Respir Dis(1983)[6412607]; Clin Pediatr (Phila)(1983)[6839622]; Pneumonol Pol(1984)[6398866]; Presse Med(1984)[6231607]; Nihon Kyobu Shikkan Gakkai Zasshi(1984)[6389948]; Gerontology(1984)[6386609]; Pathol Biol (Paris)(1985)[3897966]; Drugs(1985)[3896741]; Internist (Berl)(1985)[3897096]; Pathol Biol (Paris)(1985)[3929219]; Am Fam Physician(1985)[3883718]; Jpn J Antibiot(1985)[3937915]; J Infect(1985)[3891869]; Rev Infect Dis(1985)[3890094]; Jpn J Antibiot(1985)[3912526]; N Z Med J(1985)[3859775]; Gan No Rinsho(1985)[2414484]; Kansenshogaku Zasshi(1986)[3093606]; Drugs(1986)[3488201]; Crit Care Med(1986)[3698616]; Heart Lung(1986)[3516931]; Am J Epidemiol(1986)[3940431]; Arch Intern Med(1986)[3516102]; Chest(1986)[3096644]; Med Pediatr Oncol(1986)[3023801]; Arch Intern Med(1986)[3532983]; Postgrad Med(1987)[3615308]; J Fam Pract(1987)[3546587]; Infection(1987)[3301684]; J Infect Dis(1987)[3611832]; Childs Nerv Syst(1987)[3329960]; Zhonghua Jie He He Hu Xi Za Zhi(1987)[2836098]; Drugs(1987)[3325261]; Scand J Infect Dis Suppl(1987)[3331043]; Zentralbl Bakteriol Mikrobiol Hyg A(1987)[3115004]; Zh Mikrobiol Epidemiol Immunobiol(1987)[3564780]; Clin Rheumatol(1988)[3046829]; Boll Ist Sieroter Milan(1988)[3242560]; Kansenshogaku Zasshi(1988)[3150415]; Am J Nephrol(1988)[2967033]; Infect Control(1988)[3422227]; Vrach Delo(1988)[3061163]; J Hosp Infect(1988)[2896682]; Nihon Naika Gakkai Zasshi(1988)[2457061]; Med Clin (Barc)(1988)[3050313]; Pediatrics(1988)[3261005]; Clin Pediatr (Phila)(1989)[2805557]; Enferm Infecc Microbiol Clin(1989)[2490713]; J Nucl Med(1989)[2666597]; Ann Pediatr (Paris)(1989)[2742314]; Ned Tijdschr Geneeskd(1989)[2677797]; Kansenshogaku Zasshi(1989)[2506304]; Chemotherapy(1989)[2612237]; Jpn J Antibiot(1989)[2695657]; Scand J Infect Dis(1989)[2727632]; Zhonghua Nei Ke Za Zhi(1989)[2689107]; Indian J Pediatr(1989)[2684851]; Trans R Soc Trop Med Hyg(1989)[2617633]; J Clin Microbiol(1989)[2681247]; Rev Med Chil(1989)[2519358]; J Assoc Physicians India(1989)[2693443]; Nihon Kyobu Shikkan Gakkai Zasshi(1989)[2693782]; Plucne Bolesti(1989)[2636405]; Kansenshogaku Zasshi(1989)[2614103]; J Antimicrob Chemother(1990)[2286585]; Cancer(1990)[2364370]; J Formos Med Assoc(1990)[1982535]; Pneumonol Pol(1990)[2216912]; Jpn J Antibiot(1990)[2348550]; Pneumonol Pol(1990)[2191275]; Zhonghua Jie He He Hu Xi Za Zhi(1990)[2128219]; J Med Assoc Thai(1990)[2351897]; Wiad Lek(1990)[2275197]; Jpn J Antibiot(1990)[2232147]; Digestion(1990)[2253825]; Rev Infect Dis(1990)[2201068]; Kansenshogaku Zasshi(1990)[2335751]; Schweiz Rundsch Med Prax(1990)[2185528]; J Infect(1990)[2405058]; Jpn J Antibiot(1990)[2112207]; Med J Malaysia(1990)[2152022]; Kansenshogaku Zasshi(1990)[2243193]; Kansenshogaku Zasshi(1990)[2074366]; Am Rev Respir Dis(1990)[2240849]; Kansenshogaku Zasshi(1990)[2243194]; Drugs(1991)[1724642]; Thorax(1991)[1907034]; Scand J Infect Dis(1991)[1957125]; South Med J(1991)[1990453]; Zhonghua Yi Xue Za Zhi(1991)[1650638]; Respir Med(1991)[2014354]; Arkh Patol(1991)[1772357]; Respir Med(1991)[1759010]; Respiration(1991)[1792415]; Clin Pharm(1991)[1999086]; J Formos Med Assoc(1992)[1362112]; Zhonghua Nei Ke Za Zhi(1992)[1303851]; Antimicrob Agents Chemother(1992)[1416892]; J Assoc Physicians India(1992)[1307548]; Tidsskr Nor Laegeforen(1992)[1412305]; Immun Infekt(1992)[1563756]; J Assoc Physicians India(1992)[1634456]; Can J Infect Dis(1992)[22346408]; J Am Geriatr Soc(1992)[1401689]; Kansenshogaku Zasshi(1992)[1431388]; Am Rev Respir Dis(1992)[1731595]; Chest(1992)[1729070]; Med Klin (Munich)(1993)[8437527]; Cent Afr J Med(1993)[8020088]; J Trop Pediatr(1993)[8492372]; Chest(1993)[8222797]; J Antimicrob Chemother(1993)[7755661]; J Trop Pediatr(1993)[8133568]; Zhonghua Min Guo Xiao Er Ke Yi Xue Hui Za Zhi(1993)[8368063]; Clin Ther(1993)[8458056]; Am J Respir Crit Care Med(1994)[8004324]; Indian Pediatr(1994)[7875789]; Zhonghua Nei Ke Za Zhi(1994)[7712928]; J Chemother(1994)[7983503]; West Afr J Med(1994)[7841101]; Zhonghua Jie He He Hu Xi Za Zhi(1994)[7712575]; Kansenshogaku Zasshi(1994)[8151148]; Ryoikibetsu Shokogun Shirizu(1994)[8151961]; Zhonghua Min Guo Xiao Er Ke Yi Xue Hui Za Zhi(1994)[7942036]; Acta Paediatr(1994)[7819699]; Ethiop Med J(1994)[8033877]; Acta Haematol(1994)[8171936]; South Med J(1994)[8284724]; Pol Arch Med Wewn(1994)[7854965]; Ann Surg(1994)[8203972]; Eur J Clin Microbiol Infect Dis(1994)[8070432]; Kansenshogaku Zasshi(1994)[7829904]; Chest(1994)[8306765]; Rev Pneumol Clin(1994)[7839051]; Nihon Kyobu Shikkan Gakkai Zasshi(1994)[7799554]; J Hosp Infect(1995)[8926374]; Eur Respir J(1995)[7789484]; Transplantation(1995)[7570975]; Afr J Med Med Sci(1995)[7495200]; J Antimicrob Chemother(1995)[8543488]; Chest(1995)[7813281]; Epidemiol Mikrobiol Imunol(1995)[7489132]; Pediatr Pathol Lab Med(1995)[8597814]; J Comput Assist Tomogr(1995)[7890837]; Praxis (Bern 1994)(1995)[7481320]; J Antimicrob Chemother(1996)[8858477]; J Hosp Infect(1996)[8864938]; Jpn Circ J(1996)[8902589]; Hepatology(1996)[8666323]; Chest(1996)[8620754]; J Trop Pediatr(1996)[8820615]; N Engl J Med(1996)[8875921]; Nihon Kyobu Shikkan Gakkai Zasshi(1996)[8810756]; QJM(1996)[9015487]; Clin Infect Dis(1996)[8909844]; Infect Control Hosp Epidemiol(1996)[8934243]; Int J Antimicrob Agents(1996)[18611723]; Singapore Med J(1996)[8993135]; Clin Infect Dis(1996)[8824970]; Pediatr Infect Dis J(1996)[8970218]; Pediatr Infect Dis J(1996)[8822283]; Radiol Med(1997)[9280934]; Clin Ther(1997)[9377613]; Clin Infect Dis(1997)[9145732]; Anasthesiol Intensivmed Notfallmed Schmerzther(1997)[9498890]; Burns(1997)[9177887]; Antibiot Khimioter(1997)[9412402]; Ann Acad Med Singap(1997)[9494674]; Curr Opin Pulm Med(1997)[9193863]; Heart Lung(1997)[9315470]; Arch Pediatr(1997)[9345567]; Zhonghua Jie He He Hu Xi Za Zhi(1997)[10072803]; Semin Respir Infect(1998)[9543478]; Eur J Clin Microbiol Infect Dis(1998)[9758274]; Clin Microbiol Rev(1998)[9767057]; Zhonghua Jie He He Hu Xi Za Zhi(1998)[11477872]; Eur Respir J(1998)[9701424]; Med J Aust(1998)[9577446]; Nihon Naika Gakkai Zasshi(1998)[9921217]; Diagn Microbiol Infect Dis(1998)[9934546]; J Perinatol(1998)[9730200]; Kansenshogaku Zasshi(1998)[9545687]; Chest(1998)[9872193]; Eur Respir J(1999)[10065681]; Hosp Pract (1995)(1999)[10887440]; Laryngorhinootologie(1999)[10407826]; Kansenshogaku Zasshi(1999)[10535270]; Clin Infect Dis(1999)[10476722]; Med J Aust(1999)[10376023]; QJM(1999)[10627862]; Rev Pneumol Clin(1999)[10573750]; J Infect Chemother(1999)[11810497]; J Microbiol Immunol Infect(1999)[10650491]; J Pediatr (Rio J)(1999)[14685562]; Kansenshogaku Zasshi(1999)[10535263]; Salud Publica Mex(1999)[10608173]; Singapore Med J(1999)[10572489]; Rev Esp Quimioter(1999)[10855018]; Jpn J Antibiot(1999)[10480048]; Braz J Infect Dis(1999)[11098193]; Ryoikibetsu Shokogun Shirizu(1999)[10088417]; Chest(1999)[10084487]; Clin Chest Med(1999)[10516905]; Ann Thorac Surg(1999)[10510033]; Infez Med(1999)[12748443]; West Afr J Med(1999)[10876722]; Rev Esp Quimioter(1999)[10878531]; Ann Thorac Surg(1999)[10510007]; Clin Infect Dis(1999)[10825053]; Salud Publica Mex(1999)[10608176]; Hunan Yi Ke Da Xue Xue Bao(2000)[12516407]; J Antimicrob Chemother(2000)[10719006]; Semin Respir Crit Care Med(2000)[16088719]; Am J Respir Crit Care Med(2000)[10806182]; J Hosp Infect(2000)[10833348]; Diagn Microbiol Infect Dis(2000)[10863106]; Chest(2000)[10858403]; Eur J Clin Microbiol Infect Dis(2000)[11205629]; Eye (Lond)(2000)[11584842]; J Med Assoc Thai(2000)[10808699]; Przegl Epidemiol(2000)[11349588]; Nihon Kokyuki Gakkai Zasshi(2000)[11019572]; Semin Respir Crit Care Med(2000)[16088717]; Zhonghua Yi Xue Za Zhi (Taipei)(2000)[11076428]; Indian J Pediatr(2000)[10832217]; J Crit Care(2000)[11011820]; Minn Med(2000)[11059252]; Infect Control Hosp Epidemiol(2000)[10782588]; Kansenshogaku Zasshi(2000)[10695294]; Ann Pharmacother(2000)[10981247]; Zhonghua Jie He He Hu Xi Za Zhi(2000)[11778507]; Int J Antimicrob Agents(2001)[11165120]; Nihon Kokyuki Gakkai Zasshi(2001)[11530391]; Chang Gung Med J(2001)[11820649]; Infect Control Hosp Epidemiol(2001)[11842997]; J Trop Pediatr(2001)[11827304]; Kansenshogaku Zasshi(2001)[11321779]; Eur J Clin Microbiol Infect Dis(2001)[11837636]; Antibiot Khimioter(2001)[11558449]; Swiss Med Wkly(2001)[11875753]; J Microbiol Immunol Infect(2001)[11456359]; Arch Pediatr(2001)[11582915]; Arch Bronconeumol(2001)[11412520]; Odontostomatol Trop(2001)[11544921]; J Chemother(2001)[11589485]; Med J Malaysia(2001)[11732071]; Nihon Kokyuki Gakkai Zasshi(2001)[11530388]; Indian J Chest Dis Allied Sci(2001)[11529434]; Antibiot Khimioter(2001)[11573325]; East Afr Med J(2001)[11921576]; Respirology(2001)[11555386]; Diagn Microbiol Infect Dis(2001)[11248523]; Gac Med Mex(2001)[11432088]; J Med Assoc Thai(2001)[11460948]; Pneumologia(2001)[11977486]; Indian J Pediatr(2001)[11563247]; Crit Care Med(2001)[11246310]; Int J Infect Dis(2001)[11468101]; Paediatr Drugs(2002)[11888355]; Pharmacotherapy(2002)[11794426]; Zhonghua Jie He He Hu Xi Za Zhi(2002)[12490128]; J Microbiol Immunol Infect(2002)[11950124]; Semin Perinatol(2002)[12452506]; Lancet(2002)[12383983]; Arch Pediatr(2002)[11998425]; Clin Infect Dis(2002)[12228820]; West Indian Med J(2002)[12089869]; Rinsho Byori(2002)[12187704]; Semin Respir Infect(2002)[12226801]; Emerg Infect Dis(2002)[11749750]; J Commun Dis(2002)[14710856]; Diagn Microbiol Infect Dis(2002)[12493178]; Kansenshogaku Zasshi(2002)[11974888]; Indian Pediatr(2002)[12084946]; Southeast Asian J Trop Med Public Health(2002)[12236437]; Neurosciences (Riyadh)(2002)[23978964]; S Afr Med J(2002)[12506595]; Respirology(2002)[11896903]; Am J Infect Control(2002)[12360143]; Saudi Med J(2002)[12174226]; East Afr Med J(2002)[12643233]; Crit Care Med(2003)[12626989]; Nihon Rinsho(2003)[12722247]; Diagn Microbiol Infect Dis(2003)[12730000]; Indian Pediatr(2003)[12951383]; Zhonghua Er Ke Za Zhi(2003)[14733813]; J Cardiothorac Vasc Anesth(2003)[12635056]; Salud Publica Mex(2003)[12736986]; Zhonghua Yi Xue Za Zhi(2003)[12820912]; J Clin Pathol(2003)[12719453]; Am J Infect Control(2003)[14639438]; J Commun Dis(2004)[16295682]; Eur J Clin Microbiol Infect Dis(2004)[15558346]; P R Health Sci J(2004)[15631176]; Clin Microbiol Infect(2004)[15191383]; Am J Infect Control(2004)[15175611]; Zhonghua Yi Xue Za Zhi(2004)[15569468]; Am J Kidney Dis(2004)[15558511]; Di Yi Jun Yi Da Xue Xue Bao(2004)[15604082]; Surg Neurol(2004)[15451293]; Indian J Pediatr(2004)[15053375]; J Microbiol Immunol Infect(2004)[15599466]; J Med Assoc Thai(2004)[15217174]; J Cardiol(2004)[14750412]; Nihon Kokyuki Gakkai Zasshi(2004)[14768371]; Zhong Nan Da Xue Xue Bao Yi Xue Ban(2004)[16145922]; Zhong Nan Da Xue Xue Bao Yi Xue Ban(2004)[16134598]; Nihon Kokyuki Gakkai Zasshi(2004)[14768366]; Clin Infect Dis(2004)[15356809]; Diagn Microbiol Infect Dis(2004)[15023432]; Arq Bras Cardiol(2004)[15073655]; BMC Microbiol(2004)[15320954]; Diagn Microbiol Infect Dis(2004)[15135498]; J Infect Chemother(2004)[15614461]; J Chemother(2004)[15330325]; Cancer(2004)[15042689]; Zhonghua Jie He He Hu Xi Za Zhi(2004)[14989822]; Infect Control Hosp Epidemiol(2004)[15484803]; Chest(2004)[14769717]; Saudi Med J(2004)[15083220]; J Med Assoc Thai(2004)[15521239]; Indian J Chest Dis Allied Sci(2004)[14870864]; Infect Control Hosp Epidemiol(2004)[15518024]; Southeast Asian J Trop Med Public Health(2004)[15691151]; J Infect Chemother(2005)[15729490]; Arch Pediatr(2005)[15694540]; Tuberk Toraks(2005)[16456741]; Antibiot Khimioter(2005)[17016911]; J Hosp Infect(2005)[15893850]; Infez Med(2005)[16801748]; Saudi Med J(2005)[16311663]; Int J Antimicrob Agents(2005)[16280243]; Southeast Asian J Trop Med Public Health(2005)[16124443]; J Clin Microbiol(2005)[15635033]; Antimicrob Agents Chemother(2005)[15673761]; J Commun Dis(2005)[17278658]; J Microbiol Immunol Infect(2005)[15843855]; J Hosp Infect(2005)[16198443]; Euro Surveill(2005)[29183546]; Chang Gung Med J(2005)[16013342]; Ger Med Sci(2005)[19675723]; Chin Med J (Engl)(2005)[16313842]; Respir Med(2005)[16085210]; Southeast Asian J Trop Med Public Health(2005)[15906660]; J Formos Med Assoc(2005)[16385374]; J Clin Microbiol(2005)[16207948]; Zhonghua Liu Xing Bing Xue Za Zhi(2005)[16185459]; Euro Surveill(2005)[16077213]; Diagn Microbiol Infect Dis(2005)[15808319]; Southeast Asian J Trop Med Public Health(2005)[15906659]; Panminerva Med(2005)[16489325]; Saudi Med J(2005)[16228055]; Can Respir J(2006)[16896431]; J Hosp Infect(2006)[16307822]; Diagn Microbiol Infect Dis(2006)[17084784]; Clin Infect Dis(2006)[16652304]; Eur J Clin Microbiol Infect Dis(2006)[16767484]; J Infect(2006)[16457891]; Zhongguo Dang Dai Er Ke Za Zhi(2006)[16923372]; Zhonghua Yi Xue Za Zhi(2006)[17288792]; J Hosp Infect(2006)[16307825]; Braz J Infect Dis(2006)[17293925]; Indian J Med Res(2006)[16575119]; Pacing Clin Electrophysiol(2006)[16689852]; Saudi Med J(2006)[16680246]; J Hosp Infect(2006)[16930770]; Medicina (Kaunas)(2006)[16778466]; Zhonghua Er Ke Za Zhi(2006)[16836856]; Zhongguo Dang Dai Er Ke Za Zhi(2006)[17052391]; Zhonghua Jie He He Hu Xi Za Zhi(2006)[16638292]; Saudi Med J(2006)[16758041]; Int J Tuberc Lung Dis(2006)[17131790]; Saudi Med J(2006)[16432594]; Singapore Med J(2006)[16518554]; J Pak Med Assoc(2006)[16711336]; J Chemother(2006)[17267336]; Int J Antimicrob Agents(2006)[16979877]; J Med Assoc Thai(2006)[16696387]; Paediatr Respir Rev(2007)[17419981]; J Antimicrob Chemother(2007)[17307769]; Ann Acad Med Singap(2007)[17767334]; Clin Infect Dis(2007)[17806058]; Pharmacotherapy(2007)[17594211]; J Korean Med Sci(2007)[17728528]; Infection(2007)[17721736]; Intern Med(2007)[17675765]; Scand J Infect Dis(2007)[17852950]; Hong Kong Med J(2007)[17548905]; Zhongguo Dang Dai Er Ke Za Zhi(2007)[17706048]; J Antimicrob Chemother(2007)[17884829]; Malays J Med Sci(2007)[22993489]; J Med Assoc Thai(2007)[17375629]; Am J Infect Control(2007)[17433942]; Pediatr Crit Care Med(2007)[17273116]; Allergol Immunopathol (Madr)(2007)[17594870]; Curr Opin Pulm Med(2007)[17414126]; J Antimicrob Chemother(2007)[17540673]; J Microbiol Immunol Infect(2007)[18087630]; J Crit Care(2007)[17548024]; J Clin Microbiol(2007)[17715376]; Pneumologia(2007)[17491203]; Ann Thorac Med(2007)[19727346]; Nihon Kokyuki Gakkai Zasshi(2008)[18516992]; Int J Antimicrob Agents(2008)[18358701]; Am J Infect Control(2008)[18468549]; J Natl Med Assoc(2008)[18481475]; Braz J Infect Dis(2008)[18553018]; J Microbiol Methods(2008)[18054098]; Southeast Asian J Trop Med Public Health(2008)[18567458]; Int J Antimicrob Agents(2008)[18160263]; J Paediatr Child Health(2008)[18557808]; Int J Antimicrob Agents(2008)[18162378]; Crit Care Clin(2008)[18361952]; Antimicrob Agents Chemother(2008)[18285482]; Kyobu Geka(2008)[18323184]; J Infect Dis(2008)[18260762]; Przegl Lek(2008)[18853659]; Zhonghua Er Ke Za Zhi(2008)[19099875]; Emerg Infect Dis(2008)[18826824]; Zhonghua Jie He He Hu Xi Za Zhi(2008)[19080534]; J Chin Med Assoc(2008)[19114323]; Zhongguo Yi Xue Ke Xue Yuan Xue Bao(2008)[19024381]; Infect Control Hosp Epidemiol(2008)[18947320]; J Infect Chemother(2008)[18709531]; Zhongguo Dang Dai Er Ke Za Zhi(2008)[18706158]; Eur J Clin Microbiol Infect Dis(2008)[18317821]; Zhongguo Dang Dai Er Ke Za Zhi(2008)[18289463]; Southeast Asian J Trop Med Public Health(2008)[19058610]; BMC Infect Dis(2008)[18620553]; Cent Afr J Med(2008)[21648126]; Intern Med J(2008)[19143887]; PLoS One(2008)[18813340]; J Pediatr Hematol Oncol(2008)[18799945]; South Med J(2008)[18414163]; Curr Med Res Opin(2008)[18549664]; Ann Trop Paediatr(2008)[19021940]; Nihon Kokyuki Gakkai Zasshi(2008)[18939411]; Med Sci Monit(2008)[18376347]; Zhongguo Dang Dai Er Ke Za Zhi(2008)[18947473]; Indian J Chest Dis Allied Sci(2008)[18630792]; J Thorac Cardiovasc Surg(2008)[18603075]; Zhonghua Nei Ke Za Zhi(2008)[19080136]; Pediatr Pulmonol(2008)[18085683]; Int J Antimicrob Agents(2008)[18768302]; J Infect Chemother(2009)[20012724]; Eur Radiol(2009)[19034459]; Medicine (Baltimore)(2009)[19282702]; Nihon Kokyuki Gakkai Zasshi(2009)[19882914]; Rev Mal Respir(2009)[19953020]; Int J Infect Dis(2009)[18774741]; Cases J(2009)[19829875]; Antimicrob Agents Chemother(2009)[19164142]; Pediatr Int(2009)[19400829]; Infez Med(2009)[20046108]; Mikrobiyol Bul(2009)[20084912]; J Infect Chemother(2009)[19554400]; J Int Med Res(2009)[19589276]; Respirology(2009)[19909464]; J Hosp Infect(2009)[19013676]; Transpl Infect Dis(2009)[19392733]; J Chin Med Assoc(2009)[19687002]; Clin Infect Dis(2009)[19191615]; J Manag Care Pharm(2009)[19505173]; J Paediatr Child Health(2009)[19210603]; Euro Surveill(2009)[19341609]; Pediatr Infect Dis J(2009)[19106757]; J Infect Dev Ctries(2009)[19858572]; J Microbiol Immunol Infect(2009)[19597649]; J Infect(2009)[19246099]; Spine (Phila Pa 1976)(2009)[19139655]; Rev Chilena Infectol(2009)[19621141]; Indian J Crit Care Med(2009)[20040812]; Indian Pediatr(2009)[19213982]; J Microbiol Immunol Infect(2009)[20182669]; Zhongguo Dang Dai Er Ke Za Zhi(2009)[20113598]; Trop Med Int Health(2009)[19772545]; Anestezjol Intens Ter(2009)[20201343]; Pediatr Infect Dis J(2009)[19116601]; Int J Infect Dis(2009)[18775663]; Indian J Pediatr(2010)[20859772]; J Assoc Physicians India(2010)[21563611]; J Infect Dev Ctries(2010)[20818098]; Eur J Pediatr(2010)[20119725]; J Infect Dev Ctries(2010)[20818104]; Mikrobiyol Bul(2010)[20455393]; Respirology(2010)[20546191]; Ned Tijdschr Geneeskd(2010)[21118587]; Open Microbiol J(2010)[20448814]; J Perinat Med(2010)[20297900]; Zhonghua Liu Xing Bing Xue Za Zhi(2010)[21162844]; Med Intensiva(2010)[20605269]; Burns(2010)[20080354]; Afr J Infect Dis(2010)[23878695]; Lung India(2010)[20616935]; J Infect Dev Ctries(2010)[20351452]; J Child Neurol(2010)[19574580]; J Assoc Physicians India(2010)[21568008]; Hip Int(2010)[20544658]; Clin Infect Dis(2010)[20041768]; Am J Infect Control(2010)[20176284]; West Indian Med J(2010)[21702229]; Acta Microbiol Immunol Hung(2010)[20350878]; Infection(2010)[20878457]; Eur J Clin Microbiol Infect Dis(2010)[20505967]; Burns(2010)[19765906]; Respiration(2010)[20389050]; Chemotherapy(2010)[21099220]; Mali Med(2010)[21441087]; Ned Tijdschr Geneeskd(2010)[20858323]; Mali Med(2010)[21441088]; Zhongguo Dang Dai Er Ke Za Zhi(2010)[20350426]; J Paediatr Child Health(2010)[20796185]; Med Mal Infect(2010)[20172672]; Zhonghua Er Ke Za Zhi(2010)[21055271]; Acta Neurol Taiwan(2010)[20824541]; J Med Assoc Thai(2010)[20364567]; S Afr Med J(2010)[20529438]; J Infect Dev Ctries(2010)[20440059]; J Res Health Sci(2010)[22911918]; Zhongguo Dang Dai Er Ke Za Zhi(2010)[20199716]; Chin Med J (Engl)(2010)[21034629]; Saudi Med J(2010)[21135998]; Kansenshogaku Zasshi(2011)[21861440]; J Med Microbiol(2011)[21252273]; Int J Antimicrob Agents(2011)[21683553]; Bull Soc Pathol Exot(2011)[21103965]; Intern Med(2011)[21532221]; Int J Antimicrob Agents(2011)[21880469]; J Infect Chemother(2011)[21369775]; J Microbiol Immunol Infect(2011)[21524962]; J Clin Microbiol(2011)[21677064]; Clin Microbiol Infect(2011)[20491828]; Ophthalmic Surg Lasers Imaging(2011)[21323188]; Am J Respir Crit Care Med(2011)[21920919]; Indian J Med Microbiol(2011)[21654106]; Int J Antimicrob Agents(2011)[21605960]; Open Microbiol J(2011)[21915229]; Afr J Med Med Sci(2011)[22783679]; Ann Burns Fire Disasters(2011)[22396669]; Rev Chilena Infectol(2011)[21526282]; Pediatr Infect Dis J(2011)[21245777]; J Infect Chemother(2011)[21409533]; Indian J Pediatr(2011)[20936380]; World J Emerg Med(2011)[25214995]; Malays J Med Sci(2011)[22135583]; Med Intensiva(2011)[21334104]; J Antimicrob Chemother(2011)[21393195]; Respirology(2011)[21299688]; Zhongguo Wei Zhong Bing Ji Jiu Yi Xue(2011)[22093315]; Nepal Med Coll J(2011)[22808812]; Zhongguo Dang Dai Er Ke Za Zhi(2011)[21251380]; Zhonghua Er Ke Za Zhi(2011)[22336359]; Eur J Clin Microbiol Infect Dis(2011)[20859753]; Klin Mikrobiol Infekc Lek(2011)[22052100]; Int J Clin Pharm(2011)[21984226]; Biomed Pap Med Fac Univ Palacky Olomouc Czech Repub(2011)[22336651]; Indian J Pediatr(2011)[21553207]; Respir Care(2011)[21605476]; Clin Microbiol Infect(2011)[20345467]; Indian J Crit Care Med(2011)[21814373]; Enferm Infecc Microbiol Clin(2012)[22341749]; Am J Infect Control(2012)[21908073]; J Travel Med(2012)[23379711]; J Med Microbiol(2012)[22466031]; J Int Assoc Physicians AIDS Care (Chic)(2012)[22821806]; J Infect Chemother(2012)[22460828]; Zhongguo Dang Dai Er Ke Za Zhi(2012)[23146728]; J Microbiol Immunol Infect(2012)[22154994]; FEMS Microbiol Lett(2012)[22537112]; Roum Arch Microbiol Immunol(2012)[22838220]; J Microbiol Immunol Infect(2012)[22153763]; Turk J Haematol(2012)[24744626]; J Infect Chemother(2012)[22766652]; BMC Infect Dis(2012)[22233322]; Chin Med J (Engl)(2012)[22932165]; J Infect(2012)[22101080]; Indian J Med Res(2012)[22825611]; Transpl Infect Dis(2012)[22176504]; J Hosp Infect(2012)[22382275]; J Antimicrob Chemother(2012)[22378678]; Med Mal Infect(2012)[22341663]; J Microbiol Immunol Infect(2012)[22041167]; Expert Rev Anti Infect Ther(2012)[23253318]; Pathol Biol (Paris)(2012)[21719212]; J Craniofac Surg(2012)[22627446]; Bangladesh Med Res Counc Bull(2012)[23540185]; Acta Reumatol Port(2012)[23348115]; J Microbiol Immunol Infect(2012)[22153762]; Arch Pediatr(2012)[22381664]; Zhonghua Jie He He Hu Xi Za Zhi(2012)[22455967]; Int J Antimicrob Agents(2012)[22230331]; Am J Emerg Med(2012)[22030178]; Indian J Med Res(2012)[23287138]; Clin Infect Dis(2012)[22700828]; Euro Surveill(2012)[22913976]; Chemotherapy(2012)[23548324]; Infect Drug Resist(2012)[22570555]; Pediatrics(2012)[22232311]; J Thorac Dis(2012)[22754667]; J Med Assoc Thai(2012)[22435245]; Zhonghua Er Ke Za Zhi(2012)[23158740]; Zhongguo Wei Zhong Bing Ji Jiu Yi Xue(2012)[22316533]; Am J Infect Control(2012)[22418610]; Acta Med Port(2012)[22883249]; J Med Assoc Thai(2012)[23130469]; Arch Pediatr(2012)[22381665]; Tuberc Respir Dis (Seoul)(2012)[23101022]; Support Care Cancer(2012)[22193772]; Ann Clin Microbiol Antimicrob(2012)[22449249]; PLoS Negl Trop Dis(2012)[22363829]; Roum Arch Microbiol Immunol(2012)[23210322]; Thorax(2012)[22374921]; J Pak Med Assoc(2012)[23866508]; Sultan Qaboos Univ Med J(2012)[22375258]; Pediatr Pulmonol(2012)[21901856]; Curr Opin Pulm Med(2012)[22388585]; Zhongguo Wei Zhong Bing Ji Jiu Yi Xue(2012)[23131289]; Zhonghua Xin Xue Guan Bing Za Zhi(2012)[23302668]; Mikrobiyol Bul(2012)[22951654]; Zhongguo Dang Dai Er Ke Za Zhi(2012)[23234773]; Zhonghua Jie He He Hu Xi Za Zhi(2012)[23289990]; Saudi J Anaesth(2012)[22754435]; J Clin Diagn Res(2013)[24179913]; Southeast Asian J Trop Med Public Health(2013)[24050082]; J Clin Med Res(2013)[23390479]; Int J Infect Dis(2013)[23313157]; Biomed Res Int(2013)[24455733]; J Indian Med Assoc(2013)[24592754]; Burns(2013)[22694870]; Interact Cardiovasc Thorac Surg(2013)[23416348]; MMWR Morb Mortal Wkly Rep(2013)[23407128]; ISRN Microbiol(2013)[24078895]; Rev Stomatol Chir Maxillofac Chir Orale(2013)[23827273]; Rev Pneumol Clin(2013)[23434036]; Rev Chilena Infectol(2013)[24248105]; J Formos Med Assoc(2013)[23927986]; Ann Fr Anesth Reanim(2013)[24378956]; J Formos Med Assoc(2013)[23332427]; Gac Med Mex(2013)[23435082]; Antimicrob Agents Chemother(2013)[23571536]; Case Rep Infect Dis(2013)[23984128]; Antimicrob Agents Chemother(2013)[23229478]; Exp Ther Med(2013)[23251300]; PLoS One(2013)[23349954]; Geriatr Gerontol Int(2013)[23170823]; J Clin Diagn Res(2013)[23543819]; J Family Med Prim Care(2013)[24479091]; Acta Radiol(2013)[23463859]; Bioimpacts(2013)[23878792]; J Res Pharm Pract(2013)[24991607]; Mater Sociomed(2013)[24511262]; J Infect Dev Ctries(2013)[23592640]; Mediterr J Hematol Infect Dis(2013)[24106606]; Int J Infect Dis(2013)[23474176]; Geriatr Gerontol Int(2013)[23441872]; J Infect Dev Ctries(2013)[23416657]; J Basic Clin Pharm(2013)[24808671]; Support Care Cancer(2013)[23625018]; Pediatr Infect Dis J(2013)[23411624]; Infect Control Hosp Epidemiol(2013)[23221186]; MEDICC Rev(2013)[23686252]; N Am J Med Sci(2013)[23923107]; Infect Control Hosp Epidemiol(2013)[24225613]; Zhonghua Nei Ke Za Zhi(2013)[23856111]; Zhongguo Dang Dai Er Ke Za Zhi(2013)[23336161]; Australas Med J(2013)[24133535]; Zhonghua Yi Xue Za Zhi(2013)[23660207]; Ceylon Med J(2013)[23817934]; Trans R Soc Trop Med Hyg(2013)[23418156]; Iran J Microbiol(2013)[25848499]; Zhonghua Wai Ke Za Zhi(2013)[24256585]; Eur Rev Med Pharmacol Sci(2013)[23640443]; Chin Med J (Engl)(2013)[23786927]; Indian J Otolaryngol Head Neck Surg(2013)[24427632]; Zhonghua Wei Zhong Bing Ji Jiu Yi Xue(2013)[24021044]; J Chin Med Assoc(2013)[23351417]; J Anaesthesiol Clin Pharmacol(2013)[24106362]; Int J Infect Dis(2013)[23517779]; Rev Med Chir Soc Med Nat Iasi(2013)[24502062]; Sichuan Da Xue Xue Bao Yi Xue Ban(2013)[24059113]; Am J Infect Control(2014)[24939515]; Am J Infect Control(2014)[24969124]; Zhonghua Yi Xue Za Zhi(2014)[25622962]; Infect Control Hosp Epidemiol(2014)[24602955]; Diagn Microbiol Infect Dis(2014)[24582578]; Clin Microbiol Infect(2014)[23992130]; Case Rep Infect Dis(2014)[25309763]; Am J Infect Control(2014)[25179325]; Transplant Proc(2014)[25420864]; Pulm Pharmacol Ther(2014)[24704389]; Case Rep Infect Dis(2014)[25405043]; Medicine (Baltimore)(2014)[24797169]; Jundishapur J Microbiol(2014)[25632322]; Chin Med J (Engl)(2014)[24824237]; BMJ Case Rep(2014)[24706703]; Diagn Microbiol Infect Dis(2014)[24268534]; Scand J Infect Dis(2014)[24359516]; J Chemother(2014)[24650326]; Med Mal Infect(2014)[25453362]; Scand J Infect Dis(2014)[24354959]; PLoS One(2014)[24626053]; J Trop Med(2014)[25309601]; Malays J Pathol(2014)[25194532]; New Microbiol(2014)[24858644]; J Infect Dev Ctries(2014)[25212077]; J Infect Chemother(2014)[25224765]; Korean J Intern Med(2014)[24574833]; Arch Pediatr(2014)[24993147]; Euro Surveill(2014)[25358041]; PLoS One(2014)[24466106]; Am J Infect Control(2014)[24792718]; Transplant Proc(2014)[25242796]; Scand J Infect Dis(2014)[25288384]; J Thorac Cardiovasc Surg(2014)[25240522]; Can Respir J(2014)[24791253]; Infect Drug Resist(2014)[25152627]; BMC Infect Dis(2014)[25326650]; Zhonghua Yu Fang Yi Xue Za Zhi(2014)[25619215]; Medwave(2014)[25383685]; Antimicrob Resist Infect Control(2014)[25237477]; Zhongguo Dang Dai Er Ke Za Zhi(2014)[25140768]; Med Arch(2014)[25648217]; Scand J Infect Dis(2014)[24206450]; PLoS One(2014)[25033402]; Ups J Med Sci(2014)[24724823]; Pediatrics(2014)[24420803]; Zhonghua Liu Xing Bing Xue Za Zhi(2014)[25598264]; Transplantation(2014)[24162251]; Eur J Intern Med(2014)[24814431]; Acta Med Croatica(2014)[26012149]; J Clin Diagn Res(2014)[25121014]; J Infect(2015)[25447713]; PLoS One(2015)[25799421]; Eur J Clin Microbiol Infect Dis(2015)[26239064]; J Int Med Res(2015)[25563575]; Int J Clin Exp Med(2015)[25785141]; Cell Biochem Biophys(2015)[25347986]; J Microbiol Immunol Infect(2015)[26510516]; Drug Resist Updat(2015)[26004211]; Vnitr Lek(2015)[25693612]; Zhonghua Yi Xue Za Zhi(2015)[26704158]; Front Microbiol(2015)[26257712]; Int J Bacteriol(2015)[26904750]; Transplant Proc(2015)[26518949]; PLoS One(2015)[26407326]; Adv Exp Med Biol(2015)[25310945]; J Infect Chemother(2015)[25817352]; Transpl Infect Dis(2015)[25572932]; JNMA J Nepal Med Assoc(2015)[26994036]; Int J Infect Dis(2015)[26518064]; Pan Afr Med J(2015)[27047618]; Front Microbiol(2015)[26074910]; Pathogens(2015)[25629622]; Ann Lab Med(2015)[25932453]; J Formos Med Assoc(2015)[24055339]; Klin Mikrobiol Infekc Lek(2015)[26098488]; J Hosp Infect(2015)[25623204]; Int J Clin Exp Med(2015)[26550157]; J Antimicrob Chemother(2015)[25900159]; Infection(2015)[25595510]; J Infect Public Health(2015)[26027477]; Iran Biomed J(2015)[26220641]; Epidemiol Mikrobiol Imunol(2015)[26099612]; APMIS(2015)[25257726]; Front Med(2015)[26085469]; Front Microbiol(2015)[25741336]; PLoS One(2015)[26384242]; Am J Emerg Med(2015)[25498529]; J Thorac Dis(2015)[26543615]; Am J Infect Control(2015)[26190386]; J Glob Infect Dis(2015)[25722615]; Minerva Anestesiol(2015)[25582669]; Clin Transplant(2015)[26065630]; Oncol Lett(2015)[26622822]; Front Cell Infect Microbiol(2015)[25905047]; Int J Risk Saf Med(2015)[26639714]; Niger Med J(2015)[26759518]; Pathog Glob Health(2015)[26184918]; J Glob Health(2015)[26528392]; Front Microbiol(2015)[26733952]; Zhongguo Dang Dai Er Ke Za Zhi(2015)[26014687]; Jundishapur J Microbiol(2015)[26464771]; Medicine (Baltimore)(2015)[26287432]; Pneumonol Alergol Pol(2015)[26379000]; Zhongguo Dang Dai Er Ke Za Zhi(2015)[26412173]; Antimicrob Resist Infect Control(2015)[25815166]; J Thorac Dis(2015)[26543614]; Am J Respir Crit Care Med(2015)[25585163]; Korean J Intern Med(2015)[26552458]; |
| *Klebsiella quasivariicola* | Unlikely patho, no or very few evidence | 0 | **established** | NA |
| *Klebsiella sp. M5al* | Unlikely patho, no or very few evidence | 0 | **established** | NA |
| *Listeria monocytogenes* | Established pathogen | 79 | **established** | Lyon Med(1965)[5850574]; Am J Dis Child(1966)[4958390]; J Obstet Gynaecol Br Commonw(1971)[4997696]; Geburtshilfe Frauenheilkd(1973)[4203464]; J Gynecol Obstet Biol Reprod (Paris)(1977)[903579]; Wiad Lek(1981)[7269604]; N Y State J Med(1982)[6961312]; Rev Infect Dis(1982)[6750737]; Respiration(1983)[6836191]; Pediatr Med Chir(1985)[4094923]; Am J Clin Pathol(1985)[2983527]; Am J Dis Child(1985)[4036924]; Pediatr Infect Dis J(1987)[3670948]; Acta Neurol Scand(1988)[3407390]; Neurol Clin(1988)[3047541]; Enferm Infecc Microbiol Clin(1989)[2490456]; S Afr Med J(1989)[2919343]; Eur J Pediatr(1989)[2744036]; Bol Med Hosp Infant Mex(1989)[2631740]; Enferm Infecc Microbiol Clin(1989)[2490642]; Kansenshogaku Zasshi(1990)[1981072]; Rev Clin Esp(1990)[1982570]; Chest(1990)[2361410]; Rev Chil Pediatr(1990)[2152219]; Infection(1991)[2013506]; Przegl Epidemiol(1991)[1819820]; Schweiz Med Wochenschr Suppl(1991)[1853179]; Clin Infect Dis(1993)[8507761]; Arch Pediatr Adolesc Med(1994)[8180643]; Respiration(1995)[7784707]; Rev Esp Enferm Dig(1995)[8562197]; Med Clin North Am(1997)[9093231]; Thorax(1997)[9337838]; Hematol Cell Ther(1997)[9168306]; Scott Med J(1997)[9226774]; Cancer(1998)[9708951]; An Med Interna(1999)[10507170]; An Med Interna(1999)[10609360]; Braz J Infect Dis(1999)[11097714]; An Med Interna(2000)[11213581]; Ann Acad Med Singap(2001)[11817300]; Kansenshogaku Zasshi(2002)[12607349]; Rev Neurol(2002)[12134344]; Respiration(2002)[12169753]; Ned Tijdschr Geneeskd(2003)[12894464]; Int J Clin Pract(2004)[15206516]; Enferm Infecc Microbiol Clin(2004)[14757003]; J Natl Med Assoc(2005)[16396068]; J Infect(2006)[16260041]; J Heart Lung Transplant(2006)[16730581]; J Perinat Med(2006)[16519624]; Arch Bronconeumol(2008)[19007574]; Rev Med Chil(2008)[19301777]; Medicine (Baltimore)(2009)[19282702]; Dermatol Ther(2010)[20136915]; J Microbiol Immunol Infect(2010)[21195975]; J Korean Med Sci(2011)[21286006]; Rev Argent Microbiol(2011)[21491067]; Acta Cardiol(2011)[21894816]; Rev Chilena Infectol(2013)[24248112]; Korean J Gastroenterol(2013)[24077630]; Geriatr Gerontol Int(2013)[23551348]; Neurologist(2015)[26671740]; Case Rep Infect Dis(2015)[25802774]; Epidemiol Infect(2015)[25148655]; Jundishapur J Microbiol(2015)[25969704]; Sichuan Da Xue Xue Bao Yi Xue Ban(2018)[30378347]; Clin Microbiol Infect(2018)[29549058]; East Afr Health Res J(2018)[34308168]; Clin J Am Soc Nephrol(2018)[29622669]; J Bras Pneumol(2019)[30810643]; Zhonghua Er Ke Za Zhi(2019)[31352745]; J Geriatr Oncol(2019)[30005979]; Rev Peru Med Exp Salud Publica(2020)[32876208]; Eur J Case Rep Intern Med(2020)[32665920]; Int J Infect Dis(2021)[33515776]; Pediatr Infect Dis J(2021)[34310508]; Intern Med(2021)[34024861]; Clin Infect Dis(2022)[33876229] |
| *Moraxella catarrhalis* | Established pathogen | 452 | **established** | Kansenshogaku Zasshi(1984)[6440926]; Br Med J (Clin Res Ed)(1986)[3084017]; Curr Clin Top Infect Dis(1987)[3151758]; Pediatr Infect Dis J(1987)[3615071]; Epidemiol Infect(1987)[3119360]; Br J Dis Chest(1987)[3130089]; Rev Infect Dis(1987)[3122301]; Scand J Infect Dis(1988)[3146808]; Zhonghua Yi Xue Za Zhi(1988)[3133094]; Pathol Biol (Paris)(1988)[3136431]; Am Fam Physician(1989)[2492736]; J Antimicrob Chemother(1989)[2501265]; Taiwan Yi Xue Hui Za Zhi(1989)[2507737]; Postgrad Med(1989)[2494649]; Eur J Clin Microbiol Infect Dis(1989)[2497001]; Indian Pediatr(1989)[2517124]; Eur J Clin Microbiol Infect Dis(1989)[2495944]; Rev Clin Esp(1989)[2506614]; Semin Respir Infect(1989)[2496450]; Agressologie(1989)[2508505]; J Hosp Infect(1989)[2567760]; Clin Ter(1989)[2530028]; Clin Ther(1989)[2509070]; Clin Ther(1990)[2112984]; Ann Pediatr (Paris)(1990)[2107783]; Zhonghua Jie He He Hu Xi Za Zhi(1990)[2128216]; Plucne Bolesti(1990)[2129309]; J Infect(1990)[2125625]; Pediatr Infect Dis J(1990)[2105479]; Am J Med(1990)[2111088]; Ugeskr Laeger(1990)[2106743]; Antimicrob Agents Chemother(1990)[2127342]; Arch Intern Med(1990)[2122824]; Nihon Kyobu Shikkan Gakkai Zasshi(1990)[2120497]; Med J Aust(1991)[1905385]; Infect Dis Clin North Am(1991)[1955695]; Clin Ther(1991)[1709390]; Infect Dis Clin North Am(1991)[1955698]; J Antimicrob Chemother(1991)[1904850]; J Antimicrob Chemother(1991)[1778856]; Med Clin (Barc)(1991)[1904967]; Clin Ther(1991)[1790546]; Scand J Infect Dis(1992)[1411315]; Ann Ital Med Int(1992)[1297403]; Postgrad Med J(1992)[1287613]; Am Rev Respir Dis(1992)[1416398]; Clin Ther(1992)[1525791]; Eur J Clin Microbiol Infect Dis(1992)[1597200]; Eur Respir J(1992)[1628725]; Respir Med(1992)[1470708]; Ann Saudi Med(1992)[17587050]; Int J Antimicrob Agents(1992)[18611518]; Can J Infect Dis(1992)[22514369]; Diagn Microbiol Infect Dis(1992)[1730186]; J Antimicrob Chemother(1993)[8226438]; Thorax(1993)[8434362]; Postgrad Med(1993)[8415335]; Acta Paediatr(1993)[8241643]; Singapore Med J(1993)[8153687]; Infection(1993)[8314290]; Klin Padiatr(1993)[8350587]; Drug Investig(1993)[32287509]; Kansenshogaku Zasshi(1993)[8360519]; J Antimicrob Chemother(1993)[8396086]; Ann Saudi Med(1993)[17589103]; J Gen Intern Med(1993)[8120682]; Ann Saudi Med(1993)[17589100]; Clin Infect Dis(1994)[8086559]; Ryoikibetsu Shokogun Shirizu(1994)[8152104]; Pneumonol Alergol Pol(1994)[7866327]; J Gen Intern Med(1994)[7853075]; Infection(1994)[7927815]; Enferm Infecc Microbiol Clin(1994)[7981303]; Chemotherapy(1994)[7956452]; Ned Tijdschr Geneeskd(1994)[7935949]; Scand J Infect Dis(1994)[7984970]; Scand J Infect Dis Suppl(1994)[8047855]; Antimicrob Agents Chemother(1994)[7840581]; Malays J Pathol(1994)[16329578]; Kansenshogaku Zasshi(1994)[7996025]; J Antimicrob Chemother(1995)[8543488]; Thorax(1995)[7597657]; Monaldi Arch Chest Dis(1995)[8541820]; J Antimicrob Chemother(1995)[8537282]; Am J Respir Crit Care Med(1995)[7551388]; Thorax(1995)[7660337]; J Infect Dis(1995)[7594672]; Rev Clin Esp(1995)[7784666]; Med J Aust(1995)[7565207]; Kansenshogaku Zasshi(1995)[7751728]; J Antimicrob Chemother(1996)[8858475]; Tohoku J Exp Med(1996)[8875767]; Pediatr Rev(1996)[8857197]; Jpn J Antibiot(1996)[8721076]; Diagn Microbiol Infect Dis(1996)[8937842]; Clin Microbiol Infect(1996)[11866796]; Kansenshogaku Zasshi(1996)[8699095]; Infez Med(1996)[14976433]; Diagn Microbiol Infect Dis(1996)[8937843]; Clin Infect Dis(1996)[8729201]; Thorax(1996)[8711652]; Scand J Infect Dis(1997)[9360255]; Med Dosw Mikrobiol(1997)[9411073]; J Chemother(1997)[9248976]; J Med Microbiol(1997)[9379469]; Respiration(1997)[9154675]; Acta Paediatr Jpn(1997)[9241892]; Jpn J Antibiot(1997)[9394237]; Diagn Microbiol Infect Dis(1997)[9458982]; Curr Opin Pulm Med(1997)[9193863]; J Med Microbiol(1997)[9152030]; Pediatr Infect Dis J(1997)[9076838]; Kekkaku(1997)[9259127]; Kansenshogaku Zasshi(1997)[9132426]; Pediatr Infect Dis J(1998)[9727654]; Kansenshogaku Zasshi(1998)[9884506]; Clin Infect Dis(1998)[9770140]; Pediatr Infect Dis J(1998)[9727650]; J Antimicrob Chemother(1998)[9533462]; Med Clin (Barc)(1998)[9717161]; Arch Pediatr(1998)[10223157]; Curr Opin Pulm Med(1998)[9675520]; Eur J Clin Microbiol Infect Dis(1998)[9865980]; Diagn Microbiol Infect Dis(1998)[9823538]; Int J Antimicrob Agents(1998)[9716289]; Semin Respir Infect(1998)[9543475]; Pediatr Infect Dis J(1998)[9849979]; Am J Respir Crit Care Med(1998)[9603129]; Chest(1998)[9872194]; Zh Mikrobiol Epidemiol Immunobiol(1998)[9783407]; Chest(1998)[9631791]; J Chemother(1999)[10435684]; Clin Pediatr (Phila)(1999)[10500894]; Can Respir J(1999)[10202230]; J Paediatr Child Health(1999)[10457301]; J Antimicrob Chemother(1999)[10225579]; J Infect Chemother(1999)[11810521]; Int J Pediatr Otorhinolaryngol(1999)[10577771]; APMIS Suppl(1999)[10189833]; J Antimicrob Chemother(1999)[10590275]; Am J Health Syst Pharm(1999)[10580734]; Compr Ther(1999)[9987589]; Int J Infect Dis(1999)[10460927]; Antimicrob Agents Chemother(1999)[9925540]; South Med J(1999)[10586832]; Respir Med(1999)[10653044]; Am J Respir Crit Care Med(1999)[10390424]; Ryoikibetsu Shokogun Shirizu(1999)[10088416]; Presse Med(1999)[10506875]; Kekkaku(1999)[10067052]; Braz J Infect Dis(1999)[11098193]; Acta Paediatr(1999)[10591422]; Can J Infect Dis(1999)[22346378]; Presse Med(1999)[10605468]; Med Klin (Munich)(1999)[10603732]; Can J Infect Dis(1999)[22346371]; Eur J Clin Microbiol Infect Dis(1999)[10691195]; Jpn J Antibiot(1999)[10480048]; Chest(1999)[10424501]; Jpn J Antibiot(2000)[10923284]; Int J Antimicrob Agents(2000)[11118860]; Chemotherapy(2000)[10810209]; J Antimicrob Chemother(2000)[10797089]; Pediatr Nephrol(2000)[10955911]; J Antimicrob Chemother(2000)[10747822]; Int J Antimicrob Agents(2000)[10926447]; Semin Respir Crit Care Med(2000)[16088716]; Clin Microbiol Infect(2000)[11168105]; Trop Med Int Health(2000)[11044266]; Am J Manag Care(2000)[10977480]; Fam Pract(2000)[10758078]; J Antimicrob Chemother(2000)[10719006]; Semin Respir Infect(2000)[11052424]; J Chemother(2000)[11131955]; Int J Antimicrob Agents(2000)[11137405]; Semin Respir Crit Care Med(2000)[16088723]; Postgrad Med(2000)[19667546]; Infez Med(2000)[12709603]; Arch Bronconeumol(2000)[10932342]; An Esp Pediatr(2000)[11003900]; Braz J Infect Dis(2000)[11063556]; Saudi Med J(2000)[11376364]; J Chemother(2000)[11128560]; Pediatr Infect Dis J(2000)[11001130]; Clin Infect Dis(2001)[11320449]; Braz J Infect Dis(2001)[11980591]; Med Clin (Barc)(2001)[11412622]; Respir Med(2001)[11419670]; Manag Care Interface(2001)[11339025]; Pneumologia(2001)[11977497]; Indian J Chest Dis Allied Sci(2001)[11529434]; Am J Med(2001)[11755441]; Acta Med Port(2001)[11878155]; Diagn Microbiol Infect Dis(2001)[11248523]; Diagn Microbiol Infect Dis(2001)[11502375]; Am J Med(2001)[11755440]; Clin Ther(2001)[11726004]; Thorax(2001)[11254821]; Am J Med(2001)[11755439]; Am J Med(2001)[11755437]; Clin Infect Dis(2001)[11283803]; Int J Infect Dis(2001)[11468101]; Thorax(2001)[11209098]; J Chemother(2002)[12017369]; Int J Antimicrob Agents(2002)[12297355]; Clin Infect Dis(2002)[12410480]; Rinsho Byori(2002)[12187704]; Diagn Microbiol Infect Dis(2002)[12376036]; J Chemother(2002)[12418556]; Pediatr Infect Dis J(2002)[12182397]; N Engl J Med(2002)[12181400]; Pharmacoeconomics(2002)[12109917]; Expert Opin Investig Drugs(2002)[12084002]; J Antimicrob Chemother(2002)[12239229]; Int J Infect Dis(2002)[12718823]; Int J Antimicrob Agents(2002)[11850165]; Recenti Prog Med(2002)[12355983]; Ther Umsch(2002)[11851042]; Nursing(2003)[12575688]; Clin Microbiol Infect(2003)[14686999]; Diagn Microbiol Infect Dis(2003)[12729995]; Clin Cornerstone(2003)[14992415]; Braz J Infect Dis(2003)[12807691]; J Antimicrob Chemother(2003)[14585865]; J Antimicrob Chemother(2003)[12865398]; Pol Merkur Lekarski(2003)[12712818]; Ned Tijdschr Geneeskd(2003)[12822523]; Antimicrob Agents Chemother(2003)[12760861]; Curr Infect Dis Rep(2003)[12760822]; Microbiol Immunol(2003)[12906097]; Antimicrob Agents Chemother(2003)[12760850]; South Med J(2003)[14570346]; Paediatr Drugs(2003)[14632103]; Rev Port Pneumol(2003)[19771688]; J Clin Pathol(2003)[12719453]; Chest(2003)[12853512]; Scand J Infect Dis(2003)[12685879]; Respirology(2003)[12856747]; Pediatr Pulmonol(2003)[12772225]; Chemotherapy(2004)[15272227]; Int J Antimicrob Agents(2004)[15194123]; Antibiot Khimioter(2004)[15727145]; Zhonghua Jie He He Hu Xi Za Zhi(2004)[15130324]; Scand J Infect Dis(2004)[15198183]; Int J Antimicrob Agents(2004)[15164972]; J Pediatr (Rio J)(2004)[14978548]; J Chemother(2004)[15690686]; In Vivo(2004)[15011754]; Pediatrics(2004)[14702495]; Otolaryngol Head Neck Surg(2004)[15577783]; Southeast Asian J Trop Med Public Health(2004)[15691151]; J Infect(2004)[14667791]; Int J Antimicrob Agents(2004)[14732313]; Am J Manag Care(2004)[15521160]; Ann Pharmacother(2004)[15187209]; Treat Respir Med(2005)[15846150]; Pol Merkur Lekarski(2005)[16358917]; Int J Antimicrob Agents(2005)[15664485]; Clin Chest Med(2005)[15802165]; Neth J Med(2005)[16011015]; Chang Gung Med J(2005)[16422181]; Postgrad Med(2005)[19667702]; Intern Med J(2005)[16248860]; Diagn Microbiol Infect Dis(2005)[15808319]; Acta Paediatr Taiwan(2005)[16302571]; J Antimicrob Chemother(2005)[15649996]; Respirology(2005)[15823187]; BMC Infect Dis(2005)[15927060]; Nihon Kokyuki Gakkai Zasshi(2006)[16841712]; J Infect Chemother(2006)[16506088]; Diagn Microbiol Infect Dis(2006)[16546341]; Eur J Pediatr(2006)[16133242]; J Infect Chemother(2006)[16506084]; J Chemother(2006)[17127238]; Curr Infect Dis Rep(2006)[16643773]; Eur J Pediatr(2006)[16133243]; Eur J Pediatr(2006)[16133241]; Medicina (Kaunas)(2006)[16778466]; Int J Antimicrob Agents(2006)[17046209]; Eur J Clin Microbiol Infect Dis(2006)[16767484]; Can Respir J(2006)[16896431]; Internist (Berl)(2006)[16855849]; Rev Port Pneumol(2006)[16572254]; Clin Infect Dis(2006)[16511752]; Zhonghua Jie He He Hu Xi Za Zhi(2006)[16638292]; Arch Bronconeumol(2006)[16948991]; Med Mal Infect(2006)[16839731]; J Chemother(2006)[17267337]; J Pediatr (Rio J)(2006)[17136290]; Diagn Microbiol Infect Dis(2007)[17300907]; FEMS Immunol Med Microbiol(2007)[17439541]; Int J Antimicrob Agents(2007)[17945468]; Antimicrob Agents Chemother(2007)[17908940]; Respir Med(2007)[17548187]; Libyan J Med(2007)[21503215]; Paediatr Respir Rev(2007)[17868918]; Clin Microbiol Infect(2007)[17359321]; Respirology(2007)[17298464]; Curr Med Res Opin(2007)[17261234]; J Antimicrob Chemother(2008)[18819984]; Georgian Med News(2008)[18633147]; Zhonghua Liu Xing Bing Xue Za Zhi(2008)[19103131]; J Infect Chemother(2008)[18709531]; Scand J Infect Dis(2009)[18855228]; Epidemiol Infect(2009)[18789178]; Mikrobiyol Bul(2009)[20084912]; Intern Med(2009)[19443974]; J Infect Chemother(2009)[20012724]; J Med Assoc Thai(2009)[19253807]; J Infect Chemother(2009)[19554400]; Zhonghua Yi Xue Za Zhi(2009)[20137709]; Heart Lung(2009)[19486794]; Clin Infect Dis(2009)[19191620]; Paediatr Drugs(2010)[20590169]; J Chemother(2010)[21123150]; Expert Rev Anti Infect Ther(2010)[20014898]; Wiad Lek(2010)[21612048]; Clin Infect Dis(2010)[20014950]; Ir Med J(2010)[20666070]; PLoS One(2010)[21209964]; Indian J Pathol Microbiol(2011)[22234107]; Chemotherapy(2011)[21346354]; J Antimicrob Chemother(2011)[21482567]; Australas Med J(2011)[23393539]; J Infect Chemother(2011)[21847518]; J Infect Chemother(2011)[21409533]; Int J Antimicrob Agents(2011)[21880469]; J Infect Chemother(2011)[21369775]; Scand J Infect Dis(2011)[21892897]; J Infect Chemother(2011)[20839026]; Intensive Care Med(2011)[21877210]; Scand J Infect Dis(2011)[21466255]; Pediatr Emerg Care(2011)[21975496]; Clin Infect Dis(2011)[21628484]; Respirology(2011)[21299688]; Pediatr Infect Dis J(2011)[20625346]; Clin Microbiol Infect(2012)[21851481]; Zhonghua Jie He He Hu Xi Za Zhi(2012)[22455967]; J Infect Chemother(2012)[22766652]; Mikrobiyol Bul(2012)[23188566]; Clin Infect Dis(2012)[22903951]; J Microbiol Immunol Infect(2012)[22154675]; Pediatrics(2012)[22232311]; Indian J Med Res(2012)[23287138]; J Pak Med Assoc(2013)[24392515]; Ann Fr Anesth Reanim(2013)[23669255]; PLoS One(2013)[23940582]; Scand J Infect Dis(2013)[23286738]; Zhongguo Dang Dai Er Ke Za Zhi(2013)[23336167]; Lung(2013)[23564195]; J Microbiol Immunol Infect(2013)[23757372]; Chest(2013)[22911275]; BMC Infect Dis(2013)[23815298]; Chin Med J (Engl)(2013)[23786927]; PLoS One(2013)[24312650]; J Infect(2013)[24055804]; Lancet Respir Med(2013)[24461664]; Mediterr J Hematol Infect Dis(2013)[24106606]; Diagn Microbiol Infect Dis(2014)[24582578]; Eur Respir J(2014)[24627537]; Can J Infect Dis Med Microbiol(2014)[25285112]; Presse Med(2014)[25451635]; Med Pregl(2014)[24961047]; Int J Infect Dis(2014)[24970703]; ScientificWorldJournal(2014)[24592201]; Pediatr Infect Dis J(2014)[24717966]; PLoS One(2014)[25084351]; BMC Infect Dis(2014)[25326650]; Clin Microbiol Infect(2015)[26341913]; J Infect(2015)[25917807]; J Med Microbiol(2015)[26399701]; Genet Mol Res(2015)[26662406]; Infect Drug Resist(2015)[26261422]; Front Microbiol(2015)[26074910]; Rev Esp Quimioter(2015)[26437754]; Pan Afr Med J(2015)[27047618]; J Infect Chemother(2015)[25533886]; Eur J Clin Microbiol Infect Dis(2015)[26059041]; Zhonghua Jie He He Hu Xi Za Zhi(2015)[25791651]; J Infect Chemother(2015)[25817352]; Recent Pat Inflamm Allergy Drug Discov(2015)[25706527]; Pneumonia (Nathan)(2015)[31641578]; BMC Infect Dis(2015)[25888024]; Pediatr Infect Dis J(2015)[25764097]; Epidemiol Infect(2015)[24814418]; Pediatr Infect Dis J(2015)[25923426]; Pediatr Infect Dis J(2015)[25741972]; Zhongguo Dang Dai Er Ke Za Zhi(2016)[26781412]; Zhonghua Jie He He Hu Xi Za Zhi(2016)[26792053]; J Infect Chemother(2016)[27591787]; Zhongguo Dang Dai Er Ke Za Zhi(2016)[27530786]; J Antimicrob Chemother(2016)[27048581]; PLoS One(2016)[27959904]; Balkan Med J(2016)[27994923]; Case Rep Pulmonol(2016)[26989548]; BMC Infect Dis(2016)[27776489]; Mikrobiyol Bul(2017)[28566083]; J Infect Chemother(2017)[28669567]; Rev Med Brux(2017)[28981235]; J Infect Chemother(2017)[28431934]; Int J Chron Obstruct Pulmon Dis(2017)[28790814]; Semergen(2017)[27773623]; Pediatr Pulmonol(2017)[27458795]; J Pediatr (Rio J)(2018)[28668258]; Jpn J Infect Dis(2018)[29279451]; Zhonghua Er Ke Za Zhi(2018)[29614568]; Cytokine(2018)[29158121]; Eur J Clin Microbiol Infect Dis(2018)[29959609]; Microbiol Insights(2018)[29467579]; PLoS One(2018)[30075013]; Case Rep Infect Dis(2018)[29593918]; Infect Drug Resist(2018)[30214251]; Pediatr Pulmonol(2018)[29405664]; Recent Pat Inflamm Allergy Drug Discov(2018)[29932038]; Res Rep Trop Med(2018)[30050355]; J Hosp Infect(2019)[31054937]; J Infect Chemother(2019)[31196772]; Eur J Clin Microbiol Infect Dis(2019)[30353485]; Int J Antimicrob Agents(2019)[31404620]; Zhongguo Dang Dai Er Ke Za Zhi(2019)[31874656]; BMC Infect Dis(2019)[31694565]; Open Access Maced J Med Sci(2019)[31406530]; Pediatr Infect Dis J(2019)[31738334]; Case Rep Pediatr(2019)[30729056]; Biomed Res Int(2019)[31119172]; J Microbiol Immunol Infect(2020)[31859164]; Harefuah(2020)[32186784]; New Microbiol(2020)[32596738]; J Infect Chemother(2020)[32565151]; Travel Med Infect Dis(2020)[32305630]; IDCases(2020)[32082989]; PLoS One(2020)[32497137]; Ann Intensive Care(2020)[32894364]; Influenza Other Respir Viruses(2020)[31923349]; Infection(2020)[31482316]; Ital J Pediatr(2020)[32228653]; J Clin Microbiol(2020)[31748320]; Front Microbiol(2020)[32983056]; Front Pediatr(2020)[32850546]; J Microbiol Immunol Infect(2020)[29804657]; Ear Nose Throat J(2020)[31072191]; Drugs Context(2020)[33281908]; J Infect Chemother(2021)[33277177]; Pediatr Pulmonol(2021)[33969642]; Pneumonia (Nathan)(2021)[33894778]; BMC Infect Dis(2021)[33632144]; Infection(2021)[34687426]; Clin Infect Dis(2021)[34472572]; Front Pediatr(2021)[34589455]; Infect Dis Ther(2021)[34146254]; Respir Res(2021)[33627095]; BMC Fam Pract(2021)[33957884] |
| *Neisseria meningitidis* | Established pathogen | 191 | **established** | J Infect Dis(1970)[4983357]; JAMA(1974)[4202685]; Am Rev Respir Dis(1975)[803357]; JAMA(1975)[805852]; Am J Med(1977)[404877]; Scand J Infect Dis(1979)[111345]; JAMA(1980)[6775103]; Semin Roentgenol(1980)[6766568]; South Med J(1981)[6781070]; Am J Med(1981)[7282741]; Arch Intern Med(1981)[6784686]; Arch Intern Med(1981)[6784688]; JAMA(1981)[6793741]; J Indiana State Med Assoc(1981)[6801145]; Can Med Assoc J(1981)[6780180]; Pathol Biol (Paris)(1982)[6289228]; Am Rev Respir Dis(1982)[6802047]; NIPH Ann(1983)[6413905]; Pediatr Infect Dis(1984)[6440125]; JAMA(1985)[3871869]; Acta Pathol Microbiol Immunol Scand B(1986)[3728028]; An Esp Pediatr(1986)[3706923]; J Infect(1987)[3819455]; APMIS(1988)[3126783]; Eur J Pediatr(1989)[2591401]; Pathol Biol (Paris)(1989)[2515521]; Ann Trop Paediatr(1990)[1694651]; Zhonghua Liu Xing Bing Xue Za Zhi(1990)[2125858]; Pediatr Neurol(1991)[1908680]; Pediatrics(1991)[1984618]; Schweiz Med Wochenschr Suppl(1991)[1853179]; Ir J Med Sci(1991)[1909306]; Clin Infect Dis(1992)[1554841]; Rev Clin Esp(1992)[1598431]; Indian J Pediatr(1992)[1340860]; Med Clin (Barc)(1992)[1552785]; AIDS(1993)[8442922]; J Pediatr Nurs(1993)[8410641]; Med Pregl(1993)[7869973]; Ir J Med Sci(1993)[8119785]; An Med Interna(1994)[8193241]; Arch Bronconeumol(1994)[8186915]; J Pediatr (Rio J)(1994)[14688857]; Enferm Infecc Microbiol Clin(1995)[7654835]; Rev Pneumol Clin(1995)[7569567]; Rev Pneumol Clin(1995)[8745759]; Lancet(1995)[7853983]; Ann Intern Med(1995)[7486489]; Nihon Kyobu Shikkan Gakkai Zasshi(1996)[8953907]; Am J Med Sci(1996)[8571983]; Enferm Infecc Microbiol Clin(1996)[9011216]; Epidemiol Infect(1996)[8760956]; Thorax(1997)[9404384]; Pathol Biol (Paris)(1997)[9296075]; Scand J Infect Dis(1998)[9730313]; Int J Infect Dis(1998)[9531659]; Scand J Infect Dis(1998)[9730314]; Scott Med J(1998)[9854302]; Arch Bronconeumol(1998)[9656075]; Eur J Clin Microbiol Infect Dis(1999)[10442430]; Arch Dis Child(1999)[10325716]; An Sist Sanit Navar(1999)[12886351]; Braz J Infect Dis(1999)[11097714]; N Z Med J(1999)[10340691]; An Sist Sanit Navar(2000)[12886313]; Clin Infect Dis(2000)[10619738]; Kansenshogaku Zasshi(2001)[11357326]; Arch Pediatr(2001)[11582916]; J Trop Pediatr(2001)[11827304]; MMWR Morb Mortal Wkly Rep(2001)[11428727]; Enferm Infecc Microbiol Clin(2001)[11333613]; An Sist Sanit Navar(2001)[12876601]; Indian J Pediatr(2001)[11563252]; Emerg Infect Dis(2002)[11897072]; Pediatr Infect Dis J(2002)[12150169]; An Sist Sanit Navar(2002)[12861303]; Neurosciences (Riyadh)(2002)[23978964]; Saudi Med J(2002)[12174226]; Ned Tijdschr Geneeskd(2003)[12635550]; Am Surg(2003)[12575789]; Enferm Infecc Microbiol Clin(2003)[12732123]; Curr Infect Dis Rep(2003)[12760822]; Voen Med Zh(2003)[12722363]; An Sist Sanit Navar(2003)[12759714]; Pediatr Infect Dis J(2003)[14551480]; Neth J Med(2004)[15255084]; Euro Surveill(2004)[15591689]; An Sist Sanit Navar(2004)[15146209]; Clin Infect Dis(2004)[15156455]; Presse Med(2004)[15235505]; Salud Publica Mex(2004)[15521528]; J Trop Pediatr(2004)[15233192]; J Clin Microbiol(2005)[16145146]; Pediatr Neurol(2005)[15730898]; An Sist Sanit Navar(2005)[15827582]; Ethiop Med J(2005)[16370549]; Pediatrics(2006)[16606681]; BMC Infect Dis(2006)[16420709]; Minerva Anestesiol(2006)[16570037]; Clin Infect Dis(2006)[16511752]; Pharmacoeconomics(2006)[16460135]; An Sist Sanit Navar(2007)[17898819]; Rev Inst Med Trop Sao Paulo(2007)[17625698]; Paediatr Respir Rev(2007)[17868918]; Kansenshogaku Zasshi(2007)[18095474]; Am J Med Sci(2008)[18414075]; Rev Panam Salud Publica(2008)[18764989]; Presse Med(2008)[18676110]; Ann Thorac Surg(2008)[19049748]; Clin Infect Dis(2009)[19191614]; Am J Trop Med Hyg(2009)[19346388]; Medicine (Baltimore)(2009)[19282702]; J Microbiol Immunol Infect(2009)[20182673]; BMJ(2009)[20007220]; Pediatr Infect Dis J(2009)[19165134]; Clin Infect Dis(2009)[19191620]; Heart Surg Forum(2010)[21169146]; Med Mal Infect(2010)[19959310]; Mikrobiyol Bul(2010)[21063998]; J Korean Med Sci(2011)[21286006]; Clin Lab(2012)[22582511]; Vaccine(2012)[22709955]; J Emerg Med(2012)[20655161]; Respir Med Case Rep(2012)[26057210]; Rev Inst Med Trop Sao Paulo(2012)[22499425]; Euro Surveill(2012)[23137486]; Anaesthesiol Intensive Ther(2012)[23348489]; J Infect Dev Ctries(2012)[22706195]; Emerg Infect Dis(2012)[22261040]; J Forensic Leg Med(2013)[23910860]; Indian J Pediatr(2013)[22821284]; Popul Health Metr(2013)[24016339]; MMWR Morb Mortal Wkly Rep(2014)[25503919]; An Sist Sanit Navar(2014)[25567400]; Rev Esp Quimioter(2014)[24940897]; PLoS One(2014)[25084351]; Duodecim(2014)[25158584]; Lancet Infect Dis(2015)[25728843]; Clin Infect Dis(2015)[25389259]; MMWR Morb Mortal Wkly Rep(2015)[26068563]; Arch Argent Pediatr(2015)[26593795]; Rev Chilena Infectol(2015)[26065462]; Medicine (Baltimore)(2016)[27175654]; Iran J Pharm Res(2016)[27610176]; J Infect Chemother(2016)[27591787]; Euro Surveill(2016)[27035055]; Vaccine(2016)[27443594]; JMM Case Rep(2016)[28348748]; Mil Med(2017)[28290948]; Pediatr Infect Dis J(2017)[27902653]; Epidemiol Infect(2017)[28478773]; Ned Tijdschr Geneeskd(2017)[28745253]; Emerg Infect Dis(2017)[28628448]; Ann Hematol(2017)[28213751]; Asian Pac J Trop Med(2017)[29203096]; Clin Lab(2017)[28397477]; Rev Med Suisse(2018)[30307136]; BMJ Case Rep(2018)[29804070]; Arch Argent Pediatr(2018)[30204993]; Med Clin (Barc)(2018)[29198582]; J Clin Microbiol(2018)[29343540]; Case Rep Infect Dis(2018)[30225154]; Int Med Case Rep J(2018)[29695936]; Crit Care(2018)[29855385]; EBioMedicine(2019)[30935890]; Am J Forensic Med Pathol(2019)[30663991]; Rev Inst Med Trop Sao Paulo(2019)[31411269]; Pneumonia (Nathan)(2019)[31463180]; IDCases(2019)[30911465]; J Infect(2020)[31715210]; Vnitr Lek(2020)[32942894]; J Infect(2020)[31904388]; Epidemiol Infect(2020)[31983356]; Clin Infect Dis(2020)[31556938]; Emerg Infect Dis(2020)[32568047]; IDCases(2020)[32685370]; Eur J Case Rep Intern Med(2020)[32015972]; BMJ Case Rep(2020)[32843469]; Neurol Neurochir Pol(2020)[32115677]; BMJ Open(2020)[32273315]; Turk J Pediatr(2020)[32253877]; Lancet Digit Health(2021)[34045002]; Hum Vaccin Immunother(2021)[33449835]; BMC Infect Dis(2021)[34051739]; Epidemiol Infect(2021)[33910672]; Biomedica(2021)[34669279]; Pneumonia (Nathan)(2021)[33894778]; Diagnostics (Basel)(2021)[34359348]; Mod Rheumatol Case Rep(2022)[34850098]; Am Surg(2022)[32735451]; Infect Dis (Lond)(2022)[34459329] |
| *Pseudomonas aeruginosa* | Established pathogen | 3377 | **established** | J Albert Einstein Med Cent (Phila)(1953)[13108454]; Ann Allergy(1955)[13268970]; Minerva Med(1957)[13418525]; Z Gesamte Inn Med(1957)[13570188]; Chin Med J(1958)[13573523]; Sem Med(1962)[14013915]; Riv Anat Patol Oncol(1962)[14017087]; Mars Med(1963)[14103614]; Mycopathol Mycol Appl(1963)[14083422]; Arch Kinderheilkd(1963)[14096406]; Boll Ist Sieroter Milan(1964)[14180470]; J Pediatr(1964)[14216641]; Rev Med Suisse Romande(1965)[5851765]; J Med Bord(1965)[14344505]; J Pediatr(1966)[4954932]; Masui(1966)[4960831]; Masui(1966)[4962767]; J Antibiot B(1967)[5299330]; Pediatria (Bucur)(1967)[4967299]; J Hyg (Lond)(1967)[20475882]; Arch Roum Pathol Exp Microbiol(1967)[4974212]; Rev Med Chir Soc Med Nat Iasi(1968)[4978391]; Med Klin(1968)[4305219]; G Mal Infett Parassit(1968)[5753227]; Lancet(1968)[4170754]; G Ital Chemioter(1968)[4985840]; G Mal Infett Parassit(1968)[4976934]; Tidsskr Nor Laegeforen(1969)[4979183]; J Infect Dis(1969)[4977998]; Rev Hosp Clin Fac Med Sao Paulo(1969)[4981764]; Riv Clin Pediatr(1969)[4994077]; G Ital Chemioter(1969)[4998307]; G Ital Chemioter(1969)[4998302]; G Ital Chemioter(1969)[4998304]; Antibiotiki(1969)[4391893]; Minerva Pediatr(1969)[4929967]; Tidsskr Nor Laegeforen(1969)[4986547]; Z Erkr Atmungsorgane Folia Bronchol(1969)[4983808]; Arkh Patol(1969)[4308115]; Med Klin(1970)[5001654]; Int Anesthesiol Clin(1970)[5001154]; Am J Med Sci(1970)[4990227]; Harefuah(1970)[4993203]; Can Anaesth Soc J(1970)[4990155]; Tex Med(1971)[5001166]; Pol Tyg Lek(1971)[4997246]; Arch Dis Child(1971)[5555490]; Br Med J(1971)[4997679]; Clin Pediatr (Bologna)(1971)[5001050]; Lancet(1971)[4102629]; Haematologica(1971)[5004567]; Pol Tyg Lek(1972)[4625181]; Iryo(1972)[4623892]; Ann Intern Med(1972)[4628214]; Vestn Khir Im I I Grek(1972)[4631089]; Am J Med Sci(1972)[4630662]; Am Rev Respir Dis(1972)[4622859]; Med J Aust(1972)[4404399]; Am Rev Respir Dis(1973)[4201016]; Am J Med(1973)[4198683]; J Kans Med Soc(1973)[4198546]; J Clin Pathol(1973)[4203204]; Am Rev Respir Dis(1973)[4197698]; Orv Hetil(1973)[4755876]; Am J Roentgenol Radium Ther Nucl Med(1974)[4218451]; Acta Paediatr Scand(1974)[4215280]; Vrach Delo(1974)[4155551]; J Infect Dis(1974)[4213872]; Br Med J(1974)[4206128]; Int J Clin Pharmacol Biopharm(1975)[809373]; Cleve Clin Q(1975)[806401]; Minerva Med(1975)[1113940]; Jpn J Antibiot(1975)[239263]; Thoraxchir Vask Chir(1975)[814654]; Kansenshogaku Zasshi(1975)[806639]; Nihon Kyobu Shikkan Gakkai Zasshi(1975)[813045]; J Infect Dis(1976)[823275]; Am J Med(1976)[818897]; J Infect Dis(1976)[823276]; Sem Hop(1976)[184545]; J Infect Dis(1976)[1086876]; J Infect Dis(1976)[972281]; Am J Epidemiol(1976)[952286]; J Pediatr(1977)[401521]; Lancet(1977)[65626]; Kyobu Geka(1977)[403366]; Pediatrics(1977)[408787]; S Afr Med J(1977)[416503]; Am J Med(1977)[871128]; Med J Aust(1977)[875812]; Arch Intern Med(1978)[564671]; J Infect Dis(1978)[98596]; Blood(1978)[416862]; Arch Intern Med(1978)[415674]; Zh Mikrobiol Epidemiol Immunobiol(1978)[106610]; Am J Med(1978)[707544]; Zentralbl Bakteriol Orig A(1978)[418601]; Med Klin(1978)[102910]; Eur J Pediatr(1979)[436850]; Khirurgiia (Mosk)(1979)[109696]; Jpn J Antibiot(1979)[110964]; G Ital Chemioter(1979)[122080]; Arzneimittelforschung(1979)[120747]; Pneumonol Pol(1979)[119951]; Minerva Med(1979)[492551]; Ther Drug Monit(1980)[7222190]; Grudn Khir(1980)[6768644]; Med Clin (Barc)(1980)[7366284]; Clin Pediatr (Phila)(1980)[7000410]; Orv Hetil(1980)[7194465]; Am J Med(1980)[7424954]; Chemotherapy(1980)[7363708]; Rev Fr Mal Respir(1980)[7221170]; MMW Munch Med Wochenschr(1980)[6771633]; Ann Thorac Surg(1980)[7362314]; Monatsschr Kinderheilkd(1981)[7335093]; Jpn J Antibiot(1981)[7328749]; Anaesth Intensive Care(1981)[6945063]; Arkh Patol(1981)[7030275]; MMW Munch Med Wochenschr(1981)[6785603]; Acta Paediatr Scand(1981)[6798822]; Ann Pediatr (Paris)(1981)[6784657]; J Int Med Res(1981)[7202830]; Scand J Infect Dis Suppl(1981)[6458884]; J Pediatr(1981)[7252678]; Mt Sinai J Med(1981)[6971407]; Chest(1981)[7226908]; Cancer(1981)[7226035]; J Antimicrob Chemother(1981)[19803006]; J Pediatr(1981)[7019407]; Am Rev Respir Dis(1981)[6779685]; J Chronic Dis(1982)[7119080]; Jpn J Antibiot(1982)[6820393]; Jpn Circ J(1982)[7120653]; Scand J Infect Dis(1982)[6815787]; J Antimicrob Chemother(1982)[6815155]; Am Rev Respir Dis(1982)[6816110]; Rev Clin Esp(1982)[7146546]; Antimicrob Agents Chemother(1982)[6289739]; J Pediatr(1982)[7131162]; J Antimicrob Chemother(1982)[6815156]; Pathol Biol (Paris)(1982)[6214758]; Pathol Biol (Paris)(1982)[6812007]; Arch Dis Child(1982)[6810763]; West J Med(1982)[7147933]; Arch Dis Child(1982)[6981383]; Anaesthesist(1982)[7091639]; Pediatriia(1982)[7063308]; Jpn J Antibiot(1982)[6296478]; Jpn J Antibiot(1982)[7169662]; Zh Mikrobiol Epidemiol Immunobiol(1983)[6404080]; J Antimicrob Chemother(1983)[6352638]; Acta Paediatr Scand(1983)[6340415]; Jpn J Antibiot(1983)[6425535]; J Antimicrob Chemother(1983)[6225761]; J Antimicrob Chemother(1983)[6413486]; Br Med J (Clin Res Ed)(1983)[6402090]; J Antimicrob Chemother(1983)[6352636]; Eur J Respir Dis(1983)[6825749]; Am J Hematol(1983)[6349334]; Infect Immun(1983)[6413411]; Am Rev Respir Dis(1983)[6412607]; J Antimicrob Chemother(1983)[6352603]; Acta Paediatr Scand(1983)[6673488]; Clin Pediatr (Phila)(1983)[6224623]; Lancet(1983)[6135081]; Rev Ig Bacteriol Virusol Parazitol Epidemiol Pneumoftiziol Pneumoftiziol(1983)[6318298]; Sov Med(1983)[6612481]; J Pediatr(1983)[6348228]; Dtsch Med Wochenschr(1983)[6360617]; Clin Pediatr (Phila)(1983)[6839622]; Schweiz Med Wochenschr(1983)[6658426]; Med Clin (Barc)(1983)[6418996]; Arch Dis Child(1983)[6416186]; Presse Med(1984)[6231602]; J Pediatr(1984)[6420530]; Chest(1984)[6373170]; J Infect Dis(1984)[6421943]; Rev Infect Dis(1984)[6443771]; Antimicrob Agents Chemother(1984)[6422844]; Presse Med(1984)[6231607]; S Afr Med J(1984)[6710279]; J Clin Pathol(1984)[6368604]; Rev Clin Esp(1984)[6522709]; Pathol Biol (Paris)(1984)[6739149]; J Clin Hosp Pharm(1984)[6396321]; Jpn J Antibiot(1984)[6727034]; Eur J Cancer Clin Oncol(1984)[6537916]; West J Med(1984)[6506689]; Antimicrob Agents Chemother(1985)[3875310]; Pediatr Pulmonol(1985)[3906545]; Monatsschr Kinderheilkd(1985)[3934527]; Medicine (Baltimore)(1985)[4033411]; Drugs(1985)[3896741]; Infection(1985)[4055051]; Eur J Epidemiol(1985)[3939515]; Rev Infect Dis(1985)[2934788]; Rev Infect Dis(1985)[3901211]; Rev Infect Dis(1985)[3931199]; Chemioterapia(1985)[3986938]; Drug Intell Clin Pharm(1985)[4053983]; Pediatr Infect Dis(1985)[3885181]; Am J Med(1985)[4038577]; Am J Dis Child(1985)[3927708]; Rev Infect Dis(1985)[3909322]; Rev Infect Dis(1985)[3909321]; Am J Med(1985)[3859208]; Jpn J Antibiot(1985)[3937915]; Med J Aust(1985)[4033505]; Arch Intern Med(1985)[3927867]; Am J Med(1985)[3859211]; J Antimicrob Chemother(1985)[3935639]; Lancet(1985)[2866300]; Jpn J Antibiot(1985)[3935826]; Am J Med(1985)[4025369]; J Antimicrob Chemother(1985)[3922936]; Am J Med(1985)[4073098]; Jpn J Antibiot(1985)[3912526]; Gan To Kagaku Ryoho(1985)[4004291]; G Ital Chemioter(1985)[3833592]; Am J Med(1985)[4073096]; Acta Pathol Microbiol Immunol Scand B(1985)[3893030]; Am J Dis Child(1985)[4014087]; Infection(1986)[3759253]; Drugs(1986)[3488201]; Crit Care Med(1986)[3698616]; Lancet(1986)[2878232]; Ann Emerg Med(1986)[3535586]; Am Surg(1986)[3729174]; Pediatr Infect Dis(1986)[3725655]; Am J Dis Child(1986)[3766490]; Pathol Biol (Paris)(1986)[3534721]; Scand J Infect Dis(1986)[3518048]; Sov Med(1986)[3101205]; Immun Infekt(1986)[3100428]; J Antimicrob Chemother(1986)[3100487]; Scand J Infect Dis Suppl(1986)[3103208]; Nihon Kyobu Shikkan Gakkai Zasshi(1986)[3095578]; Chirurg(1986)[3091329]; Jpn J Antibiot(1986)[3735662]; J Infect Dis(1986)[3080535]; J Antimicrob Chemother(1987)[3301785]; Pediatr Infect Dis J(1987)[3302917]; Pharm Weekbl Sci(1987)[3438152]; J Clin Immunol(1987)[3104391]; Eur J Nucl Med(1987)[3582399]; Am J Med(1987)[3555035]; Ned Tijdschr Geneeskd(1987)[3120020]; Zhonghua Yi Xue Za Zhi(1987)[3130157]; Infection(1987)[3106232]; Kansenshogaku Zasshi(1987)[3131458]; J Antimicrob Chemother(1987)[3104273]; Acta Paediatr Scand(1987)[3425313]; Infection(1987)[3301684]; Chest(1987)[3665599]; Pediatr Infect Dis J(1987)[3588112]; Semin Respir Infect(1987)[2825316]; Zh Mikrobiol Epidemiol Immunobiol(1987)[3105204]; Eur J Epidemiol(1987)[3691745]; J Infect Dis(1987)[3611832]; Infection(1987)[3692614]; J Antimicrob Chemother(1987)[3479420]; Pharm Weekbl Sci(1987)[3438151]; Am J Med(1987)[3555037]; J Pediatr(1987)[3309236]; Pharm Weekbl Sci(1987)[3325930]; Int J Clin Pharmacol Res(1987)[3583490]; Pediatr Res(1987)[3431961]; Drugs(1987)[3325261]; J Allergy Clin Immunol(1987)[3819228]; Infection(1987)[3106233]; Drug Intell Clin Pharm(1987)[3569027]; Arch Dis Child(1987)[3111389]; Nihon Rinsho(1987)[3110462]; Infection(1987)[3112023]; J Trauma(1987)[3682042]; Eur J Respir Dis(1987)[3319661]; Drug Intell Clin Pharm(1987)[3471433]; Nihon Ronen Igakkai Zasshi(1988)[3418951]; An Esp Pediatr(1988)[3056148]; Eur J Clin Microbiol Infect Dis(1988)[3132374]; Pediatr Infect Dis J(1988)[3128767]; Pathol Biol (Paris)(1988)[3043352]; Z Erkr Atmungsorgane(1988)[3135674]; Scand J Gastroenterol Suppl(1988)[3164506]; Presse Med(1988)[2973589]; Boll Ist Sieroter Milan(1988)[3242560]; Clin Pediatr (Phila)(1988)[3123114]; Am J Otolaryngol(1988)[3177762]; J Pediatr(1988)[3127569]; Pediatr Infect Dis J(1988)[3186343]; Crit Care Med(1988)[3277780]; Rev Rhum Mal Osteoartic(1988)[3149004]; Scand J Gastroenterol Suppl(1988)[3164515]; Chest(1988)[3338299]; Arch Dis Child(1988)[3389866]; Chest(1988)[3293940]; Zh Mikrobiol Epidemiol Immunobiol(1988)[3140545]; Chest(1988)[3349830]; Rev Infect Dis(1988)[3353630]; J Med Microbiol(1988)[3143012]; Am J Med(1988)[3400692]; Intensive Care Med(1988)[3230197]; Pediatrics(1988)[3261005]; Rev Argent Microbiol(1989)[2748851]; G Ital Chemioter(1989)[2488907]; Clin Pediatr (Phila)(1989)[2805557]; Kansenshogaku Zasshi(1989)[2507688]; Med Clin (Barc)(1989)[2682060]; Biochem Soc Trans(1989)[2502454]; Kansenshogaku Zasshi(1989)[2506298]; J Antimicrob Chemother(1989)[2503488]; Thorax(1989)[2588211]; Kinderarztl Prax(1989)[2786108]; Kansenshogaku Zasshi(1989)[2507686]; Zhonghua Jie He He Hu Xi Za Zhi(1989)[2507180]; Bull Chest Dis Res Inst Kyoto Univ(1989)[2620134]; Nihon Kyobu Shikkan Gakkai Zasshi(1989)[2515331]; Am J Med(1989)[2589385]; J Med Assoc Thai(1989)[2788694]; J Antimicrob Chemother(1989)[2808196]; Pediatrie(1989)[2797997]; Pediatr Infect Dis J(1989)[2649868]; Anasth Intensivther Notfallmed(1989)[2504072]; Jpn J Antibiot(1989)[2695657]; J Clin Microbiol(1989)[2502558]; Zhonghua Nei Ke Za Zhi(1989)[2689107]; Agressologie(1989)[2802049]; Arch Intern Med(1989)[2508586]; Pediatr Pulmonol(1989)[2508049]; Pediatr Infect Dis J(1989)[2682510]; J Clin Lab Immunol(1989)[2534928]; Trans R Soc Trop Med Hyg(1989)[2617633]; Eur J Pediatr(1989)[2785036]; Hosp Pharm(1989)[10292190]; Jpn J Antibiot(1989)[2810733]; Am Rev Respir Dis(1989)[2930067]; Arch Dis Child(1989)[2513779]; Eur J Clin Microbiol Infect Dis(1989)[2512129]; J Hosp Infect(1989)[2572641]; Pathol Biol (Paris)(1989)[2780107]; Kansenshogaku Zasshi(1989)[2614103]; J Med Assoc Thai(1989)[2788692]; Pediatr Med Chir(1989)[2694104]; Acta Microbiol Hung(1990)[2129258]; Thorax(1990)[2256020]; Cancer(1990)[2364370]; Vestn Dermatol Venerol(1990)[2256385]; Nihon Kyobu Shikkan Gakkai Zasshi(1990)[2232375]; Kekkaku(1990)[2077264]; J Med Microbiol(1990)[2106033]; Semin Respir Infect(1990)[2188317]; J Med Microbiol(1990)[2115590]; Pediatr Pulmonol(1990)[1697059]; Nihon Kyobu Shikkan Gakkai Zasshi(1990)[2125088]; Aust N Z J Med(1990)[2372277]; South Med J(1990)[2116669]; Jpn J Antibiot(1990)[2348550]; Rev Infect Dis(1990)[2237114]; Zhonghua Jie He He Hu Xi Za Zhi(1990)[2128219]; J Med Assoc Thai(1990)[2351897]; Semin Respir Infect(1990)[2123990]; Jpn J Antibiot(1990)[2232147]; Jpn J Antibiot(1990)[2112208]; Jpn J Antibiot(1990)[2112206]; Kansenshogaku Zasshi(1990)[2335751]; Rev Mal Respir(1990)[2114027]; Rev Mal Respir(1990)[2114028]; Arch Dis Child(1990)[2317061]; J Antimicrob Chemother(1990)[2258339]; Jpn J Antibiot(1990)[2112207]; G Batteriol Virol Immunol(1990)[2133329]; J Formos Med Assoc(1990)[1982123]; Med Clin (Barc)(1990)[2381245]; Antimicrob Agents Chemother(1990)[2183716]; J Natl Med Assoc(1990)[2332910]; Kansenshogaku Zasshi(1990)[2243193]; Zhonghua Nei Ke Za Zhi(1990)[2209236]; Kansenshogaku Zasshi(1990)[2243194]; Rev Prat(1990)[2363011]; Agressologie(1990)[2089976]; Med J Malaysia(1990)[2152022]; Clin Ter(1990)[2150364]; Rev Clin Esp(1990)[2247678]; Kansenshogaku Zasshi(1990)[2074366]; Nihon Naika Gakkai Zasshi(1991)[1908504]; Nihon Rinsho(1991)[1749091]; Singapore Med J(1991)[1775994]; Bull Soc Pathol Exot(1991)[1666982]; Thorax(1991)[2014508]; Rev Mal Respir(1991)[1907014]; Lancet(1991)[1679870]; Arch Dis Child(1991)[1929506]; J Chemother(1991)[2019860]; Thorax(1991)[1948800]; Pathol Biol (Paris)(1991)[1923590]; J Hosp Infect(1991)[1675653]; Zhonghua Yi Xue Za Zhi(1991)[1650640]; Am Rev Respir Dis(1991)[1859053]; Nihon Rinsho(1991)[1749085]; Nihon Rinsho(1991)[1749086]; Epidemiol Infect(1991)[1675610]; DICP(1991)[1877266]; Surgery(1991)[1984637]; Pediatr Infect Dis J(1991)[2041665]; APMIS(1991)[1905144]; Nihon Rinsho(1991)[1749094]; Am Rev Respir Dis(1991)[1859056]; Rev Infect Dis(1991)[1925279]; Pediatrics(1991)[2034496]; Ann Pediatr (Paris)(1991)[1746849]; Rev Infect Dis(1991)[1781856]; Pediatr Infect Dis J(1991)[1906161]; Antibiot Chemother (1971)(1991)[1801649]; Jpn J Antibiot(1991)[1652653]; Monatsschr Kinderheilkd(1991)[1903843]; Nihon Rinsho(1991)[1749098]; Clin Microbiol Rev(1991)[1906371]; Klin Wochenschr(1991)[1813715]; Nihon Rinsho(1991)[1749095]; Kansenshogaku Zasshi(1991)[1761891]; Drugs(1991)[1724642]; MMWR Morb Mortal Wkly Rep(1991)[1910146]; J Antimicrob Chemother(1991)[1761451]; Lancet(1991)[1671990]; Crit Care Med(1991)[1989753]; J Paediatr Child Health(1992)[1554515]; Semin Respir Infect(1992)[1475544]; East Afr Med J(1992)[1473506]; Hinyokika Kiyo(1992)[1561959]; Lancet(1992)[1351559]; Eur J Pediatr(1992)[1396931]; J Intern Med(1992)[1588272]; J Antimicrob Chemother(1992)[1375595]; Rinsho Ketsueki(1992)[1404860]; Med Microbiol Immunol(1992)[1287420]; West J Med(1992)[1475945]; J Heart Lung Transplant(1992)[1540605]; Zhonghua Nei Ke Za Zhi(1992)[1303851]; Infect Control Hosp Epidemiol(1992)[1545108]; Zhonghua Jie He He Hu Xi Za Zhi(1992)[1394588]; Nihon Kyobu Shikkan Gakkai Zasshi(1992)[1405100]; Nihon Kyobu Shikkan Gakkai Zasshi(1992)[1321303]; Int J STD AIDS(1992)[1571386]; Intensive Care Med(1992)[1469184]; Acta Paediatr(1992)[1606396]; J Hosp Infect(1992)[1363110]; Nihon Kyobu Shikkan Gakkai Zasshi(1992)[1318432]; Scand J Infect Dis(1992)[1589727]; Acta Paediatr(1992)[1283959]; Enferm Infecc Microbiol Clin(1992)[1498172]; J Assoc Physicians India(1992)[1634456]; Kansenshogaku Zasshi(1992)[1331264]; Kansenshogaku Zasshi(1992)[1431355]; Jpn J Antibiot(1992)[1522670]; Br Med Bull(1992)[1281036]; Kansenshogaku Zasshi(1992)[1431388]; J Clin Lab Anal(1992)[1403341]; Kansenshogaku Zasshi(1992)[1402116]; Thorax(1992)[1549817]; Kansenshogaku Zasshi(1992)[1402091]; Gan To Kagaku Ryoho(1992)[1371045]; Kansenshogaku Zasshi(1992)[1402095]; Am J Dis Child(1992)[1496945]; Gan To Kagaku Ryoho(1992)[1371046]; Chest(1992)[1729069]; Am Rev Respir Dis(1992)[1731595]; Med J Aust(1993)[8232033]; Arch Dis Child(1993)[8257181]; Pediatrics(1993)[8424026]; J Clin Microbiol(1993)[8432839]; Br J Rheumatol(1993)[8220924]; J Natl Med Assoc(1993)[8496993]; Monaldi Arch Chest Dis(1993)[8124307]; J Med Microbiol(1993)[8418289]; Intern Med(1993)[8358116]; Chest(1993)[8222797]; J R Soc Med(1993)[7684788]; Pharmacotherapy(1993)[8469624]; S Afr Med J(1993)[8424222]; Pathol Biol (Paris)(1993)[8233627]; Minerva Chir(1993)[8177440]; Respir Med(1993)[8265842]; Monatsschr Kinderheilkd(1993)[8326956]; Semin Respir Infect(1993)[8372274]; Int J Antimicrob Agents(1993)[18611584]; Ned Tijdschr Geneeskd(1993)[8272124]; Chest(1993)[8404198]; Thorax(1993)[8434358]; Zhonghua Min Guo Xiao Er Ke Yi Xue Hui Za Zhi(1993)[8368063]; Intensive Care Med(1993)[8408937]; Kansenshogaku Zasshi(1993)[8486978]; J Cardiovasc Surg (Torino)(1994)[7775519]; Am J Respir Crit Care Med(1994)[7516251]; Pneumologie(1994)[7808992]; AIDS(1994)[7818814]; Indian J Pathol Microbiol(1994)[7868174]; Am J Respir Crit Care Med(1994)[8004324]; Med Clin (Barc)(1994)[7967870]; Kansenshogaku Zasshi(1994)[8027595]; Zhonghua Jie He He Hu Xi Za Zhi(1994)[7712575]; Kansenshogaku Zasshi(1994)[8151148]; Acta Paediatr(1994)[7981562]; South Med J(1994)[8153775]; Am J Respir Crit Care Med(1994)[8049828]; Schweiz Med Wochenschr(1994)[8140404]; J Acquir Immune Defic Syndr (1988)(1994)[8021816]; J Heart Lung Transplant(1994)[7803417]; An Med Interna(1994)[7772688]; Clin Infect Dis(1994)[8086548]; Chest(1994)[7515778]; Acta Paediatr(1994)[7819699]; Ther Drug Monit(1994)[7974622]; Acta Haematol(1994)[8171936]; Antimicrob Agents Chemother(1994)[8203853]; J Antimicrob Chemother(1994)[7961201]; Clin Infect Dis(1994)[7811859]; Am J Surg(1994)[8135314]; Clin Ther(1994)[8062319]; APMIS Suppl(1994)[7811529]; Int J Pediatr Otorhinolaryngol(1994)[8045694]; Antibiot Khimioter(1994)[8060194]; Pediatr Hematol Oncol(1994)[8060811]; Immunol Invest(1994)[8144195]; Rays(1994)[7800841]; Eur J Clin Microbiol Infect Dis(1994)[7813493]; J Hosp Infect(1994)[7852734]; Infection(1994)[8002085]; Kansenshogaku Zasshi(1994)[7996025]; Arch Intern Med(1994)[8092913]; APMIS(1994)[7946270]; Radiol Med(1994)[8128034]; Nihon Rinsho(1994)[8126901]; Thorax(1994)[7522353]; Kansenshogaku Zasshi(1994)[7829904]; Intensive Care Med(1994)[8201105]; Ned Tijdschr Geneeskd(1994)[7969610]; Thorax(1994)[8153944]; Dtsch Med Wochenschr(1994)[8070330]; J Hosp Infect(1995)[7673685]; Ear Nose Throat J(1995)[7628334]; N J Med(1995)[7724059]; Neth J Med(1995)[7643944]; Genitourin Med(1995)[7490067]; Rev Mal Respir(1995)[8677357]; Rev Pneumol Clin(1995)[7569577]; Int J STD AIDS(1995)[8845410]; Transplantation(1995)[7570975]; Przegl Lek(1995)[7784613]; J Chemother(1995)[8568541]; Radiology(1995)[7892480]; Kyobu Geka(1995)[7474582]; West Afr J Med(1995)[8634230]; Kansenshogaku Zasshi(1995)[8708405]; Rev Mal Respir(1995)[7899666]; Eur J Epidemiol(1995)[7493668]; Chest(1995)[7842786]; Acta Otorhinolaryngol Belg(1995)[7484143]; Ann Thorac Surg(1995)[7847957]; Vestn Ross Akad Med Nauk(1995)[7580418]; J Antimicrob Chemother(1995)[8543488]; J Infect(1995)[8522833]; Rev Clin Esp(1995)[8532925]; Chest(1995)[7750325]; Pediatr Cardiol(1995)[8650017]; East Afr Med J(1995)[8689973]; Rinsho Ketsueki(1995)[7783323]; Nihon Naika Gakkai Zasshi(1995)[7751792]; Antimicrob Agents Chemother(1995)[7695320]; Genitourin Med(1995)[7744417]; Kansenshogaku Zasshi(1995)[7751728]; J Med Microbiol(1995)[7884805]; Pediatr Pathol Lab Med(1995)[8597814]; J Med Assoc Thai(1995)[7666027]; Int J STD AIDS(1995)[7779926]; Thorax(1995)[7597670]; Infect Control Hosp Epidemiol(1995)[7759821]; J Clin Epidemiol(1995)[7775991]; Microb Drug Resist(1995)[9158751]; Zhonghua Min Guo Wei Sheng Wu Ji Mian Yi Xue Za Zhi(1995)[9774999]; Clin Microbiol Infect(1995)[11866739]; Can J Infect Dis(1995)[22416210]; Aust N Z J Med(1995)[7605295]; Jpn J Antibiot(1995)[7563587]; Chest(1995)[7555168]; Can J Infect Dis(1995)[22550412]; J Infect Dis(1995)[7706821]; Drugs(1995)[8549294]; Genitourin Med(1995)[7490042]; S Afr J Surg(1995)[8607056]; Eur J Clin Microbiol Infect Dis(1995)[7649191]; JAMA(1995)[7637145]; Thorax(1995)[8553305]; Radiology(1995)[7617853]; Clin Chest Med(1995)[7768090]; Nihon Geka Gakkai Zasshi(1996)[8774812]; Br J Biomed Sci(1996)[8757691]; Intern Med(1996)[9030999]; East Afr Med J(1996)[8991236]; Enferm Infecc Microbiol Clin(1996)[8714157]; Respiration(1996)[8966367]; Arch Intern Med(1996)[8862105]; Lancet(1996)[8782753]; Jpn J Antibiot(1996)[8721076]; Rev Mal Respir(1996)[8650418]; J Hosp Infect(1996)[8808748]; Thorax(1996)[8882082]; Jpn J Antibiot(1996)[8752860]; Semin Respir Infect(1996)[8776781]; Eur J Pediatr(1996)[8911895]; Thorax(1996)[8733488]; Clin Infect Dis(1996)[8879776]; Nihon Kyobu Shikkan Gakkai Zasshi(1996)[8810756]; Chest(1996)[8635325]; Pediatr Pulmonol(1996)[8726151]; J Antimicrob Chemother(1996)[8836818]; Scand J Infect Dis(1996)[8893410]; Clin Infect Dis(1996)[8909844]; Antibiot Khimioter(1996)[9124979]; Infection(1996)[8852455]; Enferm Infecc Microbiol Clin(1996)[9035706]; Anaesthesist(1996)[9065253]; Acta Paediatr(1996)[9001653]; AIDS(1996)[8970683]; Thorax(1996)[8977604]; Laeknabladid(1996)[20065393]; Clin Infect Dis(1996)[8729207]; Laeknabladid(1996)[20065391]; Kansenshogaku Zasshi(1996)[8921678]; Rev Clin Esp(1996)[8768026]; Enferm Infecc Microbiol Clin(1996)[8695681]; Mil Med(1996)[8637652]; Pediatr Infect Dis J(1996)[8822283]; Rev Clin Esp(1996)[9005471]; Presse Med(1996)[8762277]; Clin Infect Dis(1996)[8824970]; Thorax(1996)[8733486]; Bone Marrow Transplant(1997)[9012932]; Pediatr Infect Dis J(1997)[9271049]; Clin Infect Dis(1997)[9332532]; J Antimicrob Chemother(1997)[9249216]; Eur Respir J(1997)[9272915]; Ugeskr Laeger(1997)[9340883]; Chest(1997)[9377929]; J Laryngol Otol(1997)[9292138]; Nihon Kyobu Geka Gakkai Zasshi(1997)[9394590]; Radiol Med(1997)[9280934]; Ned Tijdschr Geneeskd(1997)[9132613]; Enferm Infecc Microbiol Clin(1997)[9410072]; Respiration(1997)[9154675]; Kansenshogaku Zasshi(1997)[9394563]; Curr Opin Pulm Med(1997)[9193863]; Praxis (Bern 1994)(1997)[9465725]; Braz J Infect Dis(1997)[11107239]; Infect Control Hosp Epidemiol(1997)[9120243]; Nihon Kyobu Shikkan Gakkai Zasshi(1997)[9294308]; Clin Ther(1997)[9377613]; Am J Respir Crit Care Med(1997)[9154879]; Antibiot Khimioter(1997)[9412402]; Br J Biomed Sci(1997)[9499595]; Ned Tijdschr Geneeskd(1997)[9543764]; Ann Acad Med Singap(1997)[9494663]; Antibiot Khimioter(1997)[9313057]; Pneumologie(1997)[9173416]; N Z Med J(1997)[9315030]; Pediatr Infect Dis J(1997)[9002120]; Antibiot Khimioter(1997)[9480649]; Pediatr Infect Dis J(1997)[9427460]; Pneumoftiziologia(1997)[9289238]; Am J Infect Control(1997)[9437483]; Med Clin (Barc)(1997)[9580042]; Thorax(1997)[9196512]; Indian Pediatr(1997)[9332094]; Kekkaku(1997)[9259127]; Arch Dis Child(1997)[9196361]; Zhonghua Jie He He Hu Xi Za Zhi(1997)[10072803]; Eur Respir J(1998)[9701423]; Clin Infect Dis(1998)[9524833]; Am J Respir Crit Care Med(1998)[9517611]; Pediatr Pulmonol(1998)[9635931]; J Chemother(1998)[9531074]; Khirurgiia (Sofiia)(1998)[9974040]; Ann Thorac Surg(1998)[9647112]; Jpn J Antibiot(1998)[9755430]; Kansenshogaku Zasshi(1998)[9796188]; Acta Paediatr(1998)[9628311]; Zhonghua Jie He He Hu Xi Za Zhi(1998)[11477872]; Clin Infect Dis(1998)[9564484]; Hematol Cell Ther(1998)[9924926]; Ann Fr Anesth Reanim(1998)[9881193]; Clin Infect Dis(1998)[9502454]; Chest(1998)[9596294]; G Ital Cardiol(1998)[9788044]; Clin Infect Dis(1998)[9636877]; Chest(1998)[9498979]; Pathol Biol (Paris)(1998)[9769881]; Med Clin (Barc)(1998)[9922954]; Pathol Biol (Paris)(1998)[9922991]; Thorax(1998)[10319054]; Pediatr Pulmonol(1998)[9888211]; AIDS(1998)[9792377]; Int J Antimicrob Agents(1998)[9832282]; Eur J Clin Microbiol Infect Dis(1998)[9758274]; Enferm Infecc Microbiol Clin(1998)[9808882]; J Infect(1998)[9733377]; Enferm Infecc Microbiol Clin(1998)[9918992]; Radiol Med(1998)[10051868]; Antimicrob Agents Chemother(1998)[9687398]; Thorax(1998)[10195078]; Pathol Biol (Paris)(1998)[9769865]; AIDS Patient Care STDS(1998)[15462009]; Diagn Microbiol Infect Dis(1998)[9934546]; Rev Clin Esp(1998)[9844473]; Rev Clin Esp(1998)[9844477]; Chest(1998)[9596298]; Clin Genet(1998)[9660057]; Indian J Med Res(1998)[9540279]; Arch Intern Med(1998)[9570172]; BMJ(1998)[9624062]; Zhonghua Yi Xue Za Zhi (Taipei)(1998)[9830236]; Thorax(1998)[9659359]; Am J Respir Crit Care Med(1998)[9476845]; J Perinatol(1998)[9730200]; Arch Intern Med(1998)[9570185]; Am J Respir Crit Care Med(1998)[9476869]; Arch Pediatr(1998)[9759295]; Rev Clin Esp(1998)[9658909]; Arch Dis Child(1999)[10325713]; New Microbiol(1999)[10423747]; Mediators Inflamm(1999)[10704054]; Pharmacotherapy(1999)[10331825]; Pediatr Pulmonol(1999)[10587421]; Am J Respir Crit Care Med(1999)[10471620]; Clin Perform Qual Health Care(1999)[10747571]; Rev Latinoam Microbiol(1999)[10932769]; Semin Respir Infect(1999)[10638512]; Aust N Z J Med(1999)[10200808]; Curr Opin Infect Dis(1999)[17035789]; Clin Infect Dis(1999)[10530458]; Int J Hematol(1999)[10643152]; Am Surg(1999)[10432077]; J Antimicrob Chemother(1999)[10225579]; Kansenshogaku Zasshi(1999)[10535263]; Intensive Care Med(1999)[10551961]; Chest(1999)[10084454]; Pneumologie(1999)[10684242]; J Infect(1999)[10424798]; Clin Infect Dis(1999)[10452626]; QJM(1999)[10627862]; N Engl J Med(1999)[9878641]; Respiration(1999)[10461090]; J Infect Chemother(1999)[11810521]; Infection(1999)[10885841]; J Pediatr (Rio J)(1999)[14685562]; Acta Haematol(1999)[10473881]; Conn Med(1999)[10363405]; Intern Med(1999)[10526946]; Kekkaku(1999)[10067052]; Am J Respir Crit Care Med(1999)[10430736]; Am J Respir Crit Care Med(1999)[10430704]; Respir Med(1999)[10464834]; Br Dent J(1999)[10654437]; Monaldi Arch Chest Dis(1999)[10218366]; J Antimicrob Chemother(1999)[10223595]; Chest(1999)[10084457]; Ryoikibetsu Shokogun Shirizu(1999)[10088418]; Eur J Clin Microbiol Infect Dis(1999)[10482023]; Rev Esp Quimioter(1999)[10855016]; Crit Care Med(1999)[10470744]; Schweiz Med Wochenschr(1999)[10087589]; Singapore Med J(1999)[10572489]; Enferm Infecc Microbiol Clin(1999)[10439535]; Med Clin (Barc)(1999)[10220762]; Nihon Kokyuki Gakkai Zasshi(1999)[10540836]; Transplantation(1999)[10232564]; Clin Chest Med(1999)[10516905]; J Clin Invest(1999)[10449435]; Jpn J Antibiot(1999)[10480048]; Infect Control Hosp Epidemiol(1999)[9927264]; Infez Med(1999)[12748443]; Chest(1999)[10424501]; Eur J Cardiothorac Surg(1999)[10371127]; Am J Respir Crit Care Med(1999)[10390398]; Zhonghua Jie He He Hu Xi Za Zhi(1999)[11820948]; Crit Care Med(1999)[10362409]; Eur J Clin Microbiol Infect Dis(1999)[10691195]; Chest(1999)[9925081]; Pediatrics(1999)[10103331]; Chest(1999)[10084487]; Neth J Med(1999)[10509069]; Acta Paediatr(1999)[10591422]; Pediatr Infect Dis J(1999)[10530574]; Braz J Infect Dis(1999)[11098193]; N Engl J Med(1999)[10403853]; JAMA(1999)[9892453]; Salud Publica Mex(1999)[10608176]; Dtsch Med Wochenschr(1999)[10480011]; Rev Esp Quimioter(1999)[10878529]; Eur J Clin Microbiol Infect Dis(1999)[10421044]; Antibiot Khimioter(2000)[10768062]; Anesteziol Reanimatol(2000)[11013999]; Am J Respir Crit Care Med(2000)[10988092]; Am J Respir Crit Care Med(2000)[10712318]; Chemotherapy(2000)[10765030]; Semin Respir Crit Care Med(2000)[16088744]; Am J Respir Crit Care Med(2000)[10903263]; Indian J Pediatr(2000)[10832217]; Rev Mal Respir(2000)[11076385]; Diagn Microbiol Infect Dis(2000)[10794943]; Tidsskr Nor Laegeforen(2000)[10833914]; J Formos Med Assoc(2000)[10870337]; Antibiot Khimioter(2000)[10768061]; Am Surg(2000)[11149581]; Schweiz Med Wochenschr(2000)[11059027]; Antibiot Khimioter(2000)[10768064]; Pneumonol Alergol Pol(2000)[11004844]; Cochrane Database Syst Rev(2000)[10796838]; Diagn Microbiol Infect Dis(2000)[10863106]; Clin Microbiol Infect(2000)[11168087]; J Hosp Infect(2000)[11023719]; Hunan Yi Ke Da Xue Xue Bao(2000)[12516407]; J Microbiol Immunol Infect(2000)[10917881]; Respir Med(2000)[10714482]; Semin Respir Crit Care Med(2000)[16088737]; Rev Mal Respir(2000)[11076386]; Eur J Clin Microbiol Infect Dis(2000)[10834826]; Am J Surg(2000)[10802251]; Am J Surg(2000)[10874100]; Microbiology (Reading)(2000)[11021925]; Rev Esp Quimioter(2000)[10918093]; Am J Surg(2000)[10802257]; Pediatr Pulmonol(2000)[10686042]; Am J Surg(2000)[10802259]; Am J Surg(2000)[10874106]; Curr Opin Pulm Med(2000)[11100967]; Chest(2000)[11083711]; Przegl Epidemiol(2000)[11349588]; Ann Thorac Surg(2000)[10654528]; Jpn J Antibiot(2000)[10923284]; Hua Xi Yi Ke Da Xue Xue Bao(2000)[12545825]; Chest(2000)[11115467]; Crit Care Med(2000)[10966244]; Chest(2000)[10767233]; Semin Respir Crit Care Med(2000)[16088725]; Eur J Epidemiol(2000)[10845259]; Transplantation(2000)[10868641]; Science(2000)[10818002]; Cochrane Database Syst Rev(2000)[10796836]; Chest(2000)[10807834]; Thorax(2000)[10770814]; Drugs(2000)[11129122]; Can J Infect Dis(2000)[18159274]; Chest(2000)[11115444]; Cochrane Database Syst Rev(2000)[10796732]; Arch Intern Med(2000)[10695690]; An Med Interna(2000)[10859825]; Clin Ther(2000)[10688397]; Arch Bronconeumol(2000)[11004983]; J Crit Care(2000)[11011820]; Anesthesiology(2000)[10969295]; N Z Med J(2000)[10738492]; Ann Pharmacother(2000)[10981247]; Minn Med(2000)[11059252]; Transplantation(2001)[11330535]; Infect Immun(2001)[11119558]; Recenti Prog Med(2001)[11822097]; J Hosp Infect(2001)[11289777]; J Infect Dis(2001)[11133376]; Indian J Pediatr(2001)[11770243]; J Antimicrob Chemother(2001)[11581232]; Eur J Clin Microbiol Infect Dis(2001)[11347669]; Arch Pediatr(2001)[11811055]; Int J Antimicrob Agents(2001)[11165120]; Clin Infect Dis(2001)[11320454]; Kansenshogaku Zasshi(2001)[11321779]; Am J Infect Control(2001)[11287873]; Chemotherapy(2001)[11586004]; Chest(2001)[11171773]; Med J Malaysia(2001)[11732071]; Nihon Geka Gakkai Zasshi(2001)[11828709]; Nihon Kokyuki Gakkai Zasshi(2001)[11855089]; Pediatr Pulmonol(2001)[11340683]; J Bioenerg Biomembr(2001)[11804193]; Semin Respir Crit Care Med(2001)[16088683]; Int J Antimicrob Agents(2001)[11165111]; J Microbiol Immunol Infect(2001)[11456359]; Nihon Kokyuki Gakkai Zasshi(2001)[11579526]; Med Clin (Barc)(2001)[11737996]; Braz J Infect Dis(2001)[11712965]; Int J Antimicrob Agents(2001)[11574201]; Am J Respir Crit Care Med(2001)[11254518]; Clin Microbiol Infect(2001)[11422250]; Ann Fr Anesth Reanim(2001)[11234579]; Rev Med Interne(2001)[11794881]; J Infect Dis(2001)[11443551]; South Med J(2001)[11235039]; Rev Pneumol Clin(2001)[11353919]; Infect Control Hosp Epidemiol(2001)[11583207]; Antibiot Khimioter(2001)[11558449]; Rev Pneumol Clin(2001)[11353912]; Arch Intern Med(2001)[11525709]; J Med Microbiol(2001)[11232773]; Lancet(2001)[11583754]; J Chemother(2001)[11589485]; J Formos Med Assoc(2001)[11760377]; Drugs Aging(2001)[11302286]; Curr Opin Investig Drugs(2001)[11572653]; An Esp Pediatr(2001)[11262249]; Ned Tijdschr Geneeskd(2001)[11455692]; Intensive Care Med(2001)[11355117]; Diagn Microbiol Infect Dis(2001)[11248523]; An Med Interna(2001)[11766281]; Crit Care Med(2001)[11373418]; Rev Esp Quimioter(2001)[11856984]; Can Respir J(2001)[11694916]; Ir J Med Sci(2001)[11440408]; Wiad Lek(2001)[11344693]; Minerva Anestesiol(2001)[11731755]; Thorax(2001)[11312407]; New Microbiol(2001)[11346299]; Dan Med Bull(2001)[11767129]; Braz J Infect Dis(2001)[11506775]; Crit Care Med(2001)[11801813]; Med Clin (Barc)(2001)[11412696]; J Nippon Med Sch(2001)[11505282]; Crit Care(2001)[11353934]; Cochrane Database Syst Rev(2001)[11687159]; Indian J Chest Dis Allied Sci(2001)[11529434]; Cochrane Database Syst Rev(2001)[11687002]; Pediatr Pulmonol(2001)[11596160]; Respirology(2001)[11555386]; Crit Care Med(2001)[11246310]; J Pediatr(2001)[11343046]; Gac Med Mex(2001)[11432088]; Paediatr Drugs(2002)[11888355]; Chest(2002)[11796432]; J Infect Chemother(2002)[11957128]; Allergy Asthma Proc(2002)[11894730]; Rev Cubana Med Trop(2002)[15849942]; Scand J Infect Dis(2002)[12160171]; Hosp Med(2002)[12187603]; Pediatr Pulmonol(2002)[12357482]; Pediatr Pulmonol(2002)[11747258]; Zhonghua Nei Ke Za Zhi(2002)[12189118]; Pharmacotherapy(2002)[11898886]; Emerg Infect Dis(2002)[11749750]; Chest(2002)[12475862]; J Cyst Fibros(2002)[15463836]; J Cyst Fibros(2002)[15463815]; J Cyst Fibros(2002)[15463835]; Thorax(2002)[12096202]; JAMA(2002)[12052125]; Eur Respir J Suppl(2002)[12168745]; Pneumologie(2002)[12375221]; Pediatr Pulmonol(2002)[12112775]; Pediatr Ann(2002)[11862722]; J Hosp Infect(2002)[12392904]; Am J Respir Crit Care Med(2002)[12359658]; Semin Respir Infect(2002)[11891518]; Eur J Cardiothorac Surg(2002)[11932159]; Rev Esp Quimioter(2002)[12587042]; Eur Respir J(2002)[12358344]; Clin Infect Dis(2002)[11914992]; J Chemother(2002)[12583547]; Chest(2002)[11796436]; J Perinat Med(2002)[12012636]; Jpn J Antibiot(2002)[12532637]; Rinsho Byori(2002)[12187704]; Lancet(2002)[11812595]; Kansenshogaku Zasshi(2002)[12212326]; J Med Microbiol(2002)[12448673]; Am J Respir Crit Care Med(2002)[11934711]; Eur Respir J(2002)[12503708]; Chest(2002)[11796431]; Southeast Asian J Trop Med Public Health(2002)[12757224]; Hunan Yi Ke Da Xue Xue Bao(2002)[12575239]; Int J Cardiol(2002)[11959388]; Acta Paediatr Taiwan(2002)[12148967]; Am J Respir Crit Care Med(2002)[12379545]; J Hosp Infect(2002)[11886208]; South Med J(2002)[12081224]; Am J Respir Crit Care Med(2002)[12153970]; Int J Clin Pract Suppl(2002)[12014853]; Pediatr Pulmonol(2002)[12205566]; Infez Med(2002)[12702886]; |
| *Serratia marcescens* | Established pathogen | 112 | **established** | J Am Med Assoc(1957)[13438695]; Acta Pathol Microbiol Scand(1963)[14014586]; Ann Med Nancy(1965)[5322133]; Am Rev Respir Dis(1965)[5319449]; Ann Intern Med(1966)[5330144]; Presse Med (1893)(1967)[5334931]; G Mal Infett Parassit(1968)[4896745]; Arch Surg(1969)[4893857]; Arch Intern Med(1973)[4593191]; Antibiotiki(1973)[4587063]; J Infect Dis(1973)[4577976]; Anesth Analg(1974)[4606396]; Ann Thorac Surg(1975)[1093494]; Am J Med(1977)[326046]; Arch Surg(1977)[334114]; Zh Mikrobiol Epidemiol Immunobiol(1978)[369251]; Health Lab Sci(1978)[359506]; Arch Intern Med(1978)[415674]; Kansenshogaku Zasshi(1978)[103986]; Med Clin North Am(1980)[6993805]; Semin Roentgenol(1980)[6766568]; Am J Med(1981)[7304656]; Am Rev Respir Dis(1981)[7013585]; Chest(1981)[7226908]; G Ital Chemioter(1982)[6764433]; Jpn J Antibiot(1982)[6296478]; Infect Control(1983)[6354957]; Am J Infect Control(1983)[6349427]; Am J Med(1985)[4025369]; Scand J Infect Dis Suppl(1986)[3103208]; Ann Thorac Surg(1988)[3281615]; Rev Infect Dis(1988)[3353630]; Arch Belg(1989)[2700064]; Clin Pediatr (Phila)(1989)[2805557]; Pediatr Infect Dis J(1989)[2649868]; J Clin Microbiol(1989)[2681247]; Jpn J Antibiot(1990)[2232147]; Harefuah(1990)[2227673]; Enferm Infecc Microbiol Clin(1990)[2099856]; Rev Clin Esp(1990)[2247678]; Kansenshogaku Zasshi(1990)[2243194]; Kansenshogaku Zasshi(1990)[2126799]; J Hosp Infect(1992)[1358961]; Arch Intern Med(1992)[1558444]; Kansenshogaku Zasshi(1992)[1402091]; J Heart Lung Transplant(1993)[8241235]; Acta Paediatr Jpn(1995)[7645393]; Infect Control Hosp Epidemiol(1995)[7673648]; Kansenshogaku Zasshi(1995)[7751728]; J Fam Pract(1996)[8636679]; Radiol Med(1997)[9280934]; Pediatr Infect Dis J(1997)[9380455]; J Formos Med Assoc(1997)[9290280]; J Microbiol Immunol Infect(1998)[10496154]; Rev Invest Clin(1998)[9608784]; Transplantation(1999)[10232564]; Ryoikibetsu Shokogun Shirizu(1999)[10088421]; J Formos Med Assoc(1999)[10634026]; Arch Bronconeumol(1999)[10330545]; Eur J Clin Microbiol Infect Dis(2000)[10834812]; Nihon Saikingaku Zasshi(2001)[11577410]; Eur J Clin Microbiol Infect Dis(2001)[11837636]; J Microbiol Immunol Infect(2001)[11456359]; Crit Care Med(2001)[11246310]; J Perinatol(2002)[11948393]; N Engl J Med(2002)[12015392]; J Hosp Infect(2002)[12473470]; Indian J Med Microbiol(2002)[17657061]; Rinsho Byori(2003)[14743739]; Nihon Rinsho(2003)[12722248]; Diagn Microbiol Infect Dis(2004)[15023432]; Infect Control Hosp Epidemiol(2004)[15484794]; Int J Antimicrob Agents(2005)[16280243]; J Hosp Infect(2005)[16198443]; Spine (Phila Pa 1976)(2006)[16985448]; Eur J Clin Microbiol Infect Dis(2007)[17587073]; Infect Control Hosp Epidemiol(2007)[17385143]; Allergol Immunopathol (Madr)(2007)[17594870]; Arq Neuropsiquiatr(2007)[18094868]; Infect Control Hosp Epidemiol(2008)[18419363]; Eur J Clin Microbiol Infect Dis(2008)[18317821]; Przegl Lek(2008)[18853659]; Korean J Pediatr(2010)[21189945]; Bull Soc Pathol Exot(2011)[21103965]; Int J Gen Med(2011)[21941452]; Zh Mikrobiol Epidemiol Immunobiol(2012)[23163042]; Tuberc Respir Dis (Seoul)(2012)[23101022]; MEDICC Rev(2013)[23686252]; J Clin Diagn Res(2014)[25120985]; J Clin Immunol(2014)[24402618]; J Microbiol Immunol Infect(2014)[23751769]; Ther Clin Risk Manag(2014)[25258539]; Med Intensiva(2015)[26208763]; Gen Thorac Cardiovasc Surg(2015)[24091537]; Pan Afr Med J(2015)[27047618]; Scott Med J(2015)[26265740]; BMC Pharmacol Toxicol(2016)[27004519]; Cureus(2016)[27433413]; Enferm Infecc Microbiol Clin(2016)[26900002]; Eur J Dermatol(2016)[27052490]; J Med Microbiol(2017)[27911257]; Adv Exp Med Biol(2017)[27739023]; Semergen(2017)[27773623]; J Crit Care Med (Targu Mures)(2018)[29967898]; Med J Armed Forces India(2018)[30449928]; Rev Iberoam Micol(2019)[30503225]; New Microbes New Infect(2019)[31763048]; Am J Infect Control(2019)[30527282]; Am J Case Rep(2021)[33649287]; Respir Med Case Rep(2021)[34401281]; CEN Case Rep(2022)[34302598]; Infect Dis (Lond)(2022)[34382910] |
| *Staphylococcus aureus* | Established pathogen | 3055 | **established** | J Radiol Electrol Arch Electr Medicale(1953)[13097495]; J Fr Med Chir Thorac(1953)[13096683]; Arch Kinderheilkd(1954)[13208213]; J Radiol Electrol Arch Electr Medicale(1954)[13184505]; Pediatrie(1954)[13166471]; Poumon(1954)[13155306]; Poumon(1954)[13155305]; J Fr Med Chir Thorac(1954)[13211961]; Poumon(1954)[13155307]; Arch Kinderheilkd(1955)[13259604]; Alger Medicale(1955)[14398593]; Tuberkulosearzt(1955)[13291497]; Can Serv Med J(1955)[14352158]; Sem Hop(1955)[14396400]; Presse Med (1893)(1955)[14395134]; Toulouse Med(1955)[13311923]; Gyermekgyogyaszat(1956)[13318480]; Policlinico Prat(1956)[13353967]; Lancet(1956)[13320874]; Surg Forum(1956)[13391415]; Lancet(1956)[13320873]; Med J Aust(1956)[13321222]; Lyon Med(1956)[13308252]; Policlinico Prat(1956)[13310293]; J Pediatr(1957)[13417022]; Lancet(1958)[13564806]; Schweiz Med Wochenschr(1958)[13568733]; Zhonghua Nei Ke Za Zhi(1959)[13652275]; Harper Hosp Bull(1960)[13798058]; Harefuah(1960)[14435167]; Schweiz Med Wochenschr(1961)[14471377]; Orv Hetil(1967)[5634303]; N Engl J Med(1968)[4232865]; JAMA(1970)[5467891]; Am J Med Sci(1972)[4486504]; J Indian Med Assoc(1972)[4644909]; J Pediatr(1974)[4820707]; Bol Med Hosp Infant Mex(1974)[4425554]; Am J Dis Child(1975)[123704]; Nouv Presse Med(1975)[1101214]; Scand J Infect Dis(1975)[1101372]; Rev Ig Bacteriol Virusol Parazitol Epidemiol Pneumoftiziol Bacteriol Virusol and Parazitol Epidemiol(1976)[134444]; Pediatrics(1976)[934785]; Vestn Akad Med Nauk SSSR(1976)[1266374]; Am J Med(1976)[1048860]; Ann Sclavo(1976)[13744]; Srp Arh Celok Lek(1977)[616662]; J Clin Pathol(1977)[325018]; J Fam Pract(1977)[320285]; Cesk Epidemiol Mikrobiol Imunol(1977)[144030]; Am J Med(1977)[871128]; Pediatrics(1978)[32513]; J Infect Dis(1978)[24669]; West J Med(1978)[706357]; Arch Fr Pediatr(1978)[637669]; Pediatrics(1978)[634675]; Arch Dis Child(1979)[475415]; Gig Sanit(1979)[527832]; P N G Med J(1979)[299332]; Postgrad Med J(1979)[44909]; Arch Intern Med(1979)[32855]; Bol Med Hosp Infant Mex(1979)[313800]; Pneumonol Pol(1979)[119214]; Pediatrics(1980)[6990374]; Semin Roentgenol(1980)[7355303]; Mayo Clin Proc(1980)[7442320]; Sov Med(1980)[6998021]; Br Med J(1980)[7448549]; Infection(1980)[7399717]; Vrach Delo(1980)[7405165]; Grudn Khir(1980)[7372183]; Ann Intern Med(1980)[6904159]; Zhonghua Jie He He Hu Xi Xi Ji Bing Za Zhi(1980)[7227119]; Zhonghua Jie He He Hu Xi Xi Ji Bing Za Zhi(1980)[7227118]; MMW Munch Med Wochenschr(1981)[6785603]; Tijdschr Kindergeneeskd(1981)[7302937]; Monatsschr Kinderheilkd(1981)[7335093]; P N G Med J(1981)[6951351]; Ter Arkh(1981)[7027504]; Lab Delo(1981)[6170829]; Dtsch Med Wochenschr(1981)[7193562]; Thorax(1981)[7314031]; Med J Aust(1981)[7029225]; Chest(1981)[7471872]; Cancer(1981)[7226035]; Ir Med J(1981)[7319781]; Am J Dis Child(1982)[7091060]; Probl Gematol Pereliv Krovi(1982)[6953402]; Am J Trop Med Hyg(1982)[7102917]; J Thorac Cardiovasc Surg(1982)[7121040]; Ann Intern Med(1982)[7114628]; Pediatr Infect Dis(1982)[7177890]; J Infect(1982)[7185980]; Anaesthesist(1982)[7091639]; Pediatr Infect Dis(1982)[6755404]; Ann Pediatr (Paris)(1982)[7081902]; Arch Intern Med(1982)[7052006]; Rev Ig Bacteriol Virusol Parazitol Epidemiol Pneumoftiziol Pneumoftiziol(1982)[6296978]; Scand J Infect Dis(1982)[6925916]; South Med J(1983)[6823612]; Infect Control(1983)[6354957]; J Antimicrob Chemother(1983)[6352603]; Trop Doct(1983)[6679397]; Jpn J Antibiot(1983)[6348341]; Jpn J Antibiot(1983)[6425535]; Jpn J Antibiot(1983)[6348340]; Pediatr Med Chir(1983)[6647075]; Lab Delo(1983)[6194362]; Am J Infect Control(1983)[6349427]; Am J Hematol(1983)[6349334]; Clin Pediatr (Phila)(1983)[6839622]; Schweiz Med Wochenschr(1983)[6658426]; J Natl Med Assoc(1984)[6471117]; Pneumonol Pol(1984)[6398866]; Postgrad Med J(1984)[6709548]; Minerva Med(1984)[6709217]; J Clin Pathol(1984)[6368604]; J Trauma(1984)[6481828]; Monatsschr Kinderheilkd(1984)[6727895]; Am J Dis Child(1984)[6695867]; Jpn J Antibiot(1984)[6587132]; S Afr Med J(1984)[6710279]; S Afr Med J(1984)[6695248]; S Afr Med J(1984)[6495111]; Am J Med Sci(1984)[6610355]; Dis Mon(1984)[6386397]; Scand J Thorac Cardiovasc Surg(1984)[6719079]; Ann Fr Anesth Reanim(1984)[6476503]; An Esp Pediatr(1984)[6703532]; Pediatrics(1984)[6718113]; Infection(1985)[4055051]; Am Fam Physician(1985)[3883718]; Jpn J Antibiot(1985)[3937915]; J Infect(1985)[4031512]; Am J Med(1985)[3859208]; Ann Intern Med(1985)[2996410]; Ann Pediatr (Paris)(1985)[4051416]; Eur J Clin Microbiol(1985)[4018066]; Infect Control(1985)[3847402]; J Pediatr(1985)[3973782]; Am J Med(1985)[4014285]; Acta Chir Belg(1985)[4013584]; J Infect(1985)[3891869]; An Esp Pediatr(1986)[3706923]; Am J Epidemiol(1986)[3940431]; Zhonghua Jie He He Hu Xi Xi Ji Bing Za Zhi(1986)[3527607]; Infection(1986)[3759253]; South Med J(1986)[3486480]; Arch Intern Med(1986)[3516102]; Scand J Infect Dis Suppl(1986)[3103208]; N Z Med J(1986)[3456113]; Monatsschr Kinderheilkd(1986)[3748039]; Zhonghua Nei Ke Za Zhi(1986)[3743222]; J Trauma(1986)[3795301]; Pharm Weekbl Sci(1987)[3438152]; Medicine (Baltimore)(1987)[3626846]; Microbiologica(1987)[3695983]; Drug Intell Clin Pharm(1987)[3569027]; Am Rev Respir Dis(1987)[3310768]; Zhonghua Jie He He Hu Xi Za Zhi(1987)[3450416]; J Fam Pract(1987)[3546587]; Nihon Kyobu Shikkan Gakkai Zasshi(1987)[3449690]; Q J Med(1987)[3116595]; Arch Pathol Lab Med(1987)[3307685]; Infect Control(1987)[3643889]; Surg Gynecol Obstet(1987)[3660197]; Crit Care Med(1987)[3568713]; Postgrad Med(1987)[3615308]; Infect Control(1987)[3643888]; Jpn J Antibiot(1987)[3586339]; Rev Mal Respir(1987)[3671863]; Am J Dis Child(1987)[3673968]; Am J Med(1987)[3578359]; Zhong Xi Yi Jie He Za Zhi(1987)[3447764]; Pharm Weekbl Sci(1987)[3325930]; Infection(1987)[3301684]; Ugeskr Laeger(1987)[3451526]; Antimicrob Agents Chemother(1987)[3304156]; Vestn Khir Im I I Grek(1987)[3424535]; Ann Trop Paediatr(1987)[2441645]; Pediatr Radiol(1987)[2819815]; Pediatr Pulmonol(1987)[3501100]; Scand J Infect Dis(1987)[3441748]; Wiad Lek(1987)[3442018]; Semin Respir Infect(1987)[3321267]; Zentralbl Bakteriol Mikrobiol Hyg A(1987)[3115004]; Kansenshogaku Zasshi(1988)[3147308]; J Hyg Epidemiol Microbiol Immunol(1988)[3397528]; J Hosp Infect(1988)[2896723]; Am J Med Sci(1988)[3344761]; Crit Care Med(1988)[3277780]; J Hosp Infect(1988)[2896692]; Rev Ig Bacteriol Virusol Parazitol Epidemiol Pneumoftiziol Pneumoftiziol(1988)[2849190]; Scand J Infect Dis(1988)[3406669]; Arch Dis Child(1988)[3196056]; Z Erkr Atmungsorgane(1988)[3135674]; Pediatriia(1988)[3264401]; Nihon Geka Gakkai Zasshi(1988)[3146686]; Chest(1988)[3338299]; Pediatr Radiol(1988)[3387161]; Rev Mal Respir(1988)[2835800]; Southeast Asian J Trop Med Public Health(1988)[3227408]; Khirurgiia (Mosk)(1988)[3236705]; Monatsschr Kinderheilkd(1988)[3352613]; Rev Infect Dis(1988)[3353630]; Clin Chest Med(1988)[3044680]; Chest(1988)[3293940]; Am J Surg(1988)[3202266]; Zh Mikrobiol Epidemiol Immunobiol(1988)[3140545]; Med J Aust(1989)[2739613]; Med J Aust(1989)[2642592]; Zhonghua Zhong Liu Za Zhi(1989)[2550200]; Ann Pediatr (Paris)(1989)[2742314]; Jpn J Antibiot(1989)[2695657]; Klin Wochenschr(1989)[2545969]; Wiad Lek(1989)[2634313]; Trans R Soc Trop Med Hyg(1989)[2617633]; Nihon Kyobu Shikkan Gakkai Zasshi(1989)[2698422]; Rev Argent Microbiol(1989)[2748851]; J Hosp Infect(1989)[2575629]; An Med Interna(1989)[2491492]; Kinderarztl Prax(1989)[2786108]; Eur J Clin Microbiol Infect Dis(1989)[2495944]; Pediatr Infect Dis J(1989)[2594461]; Vrach Delo(1989)[2667256]; Zhonghua Nei Ke Za Zhi(1989)[2689107]; Chest(1989)[2651040]; Eur J Clin Microbiol Infect Dis(1989)[2495953]; Enferm Infecc Microbiol Clin(1989)[2490642]; CMAJ(1989)[2642395]; Int J Pediatr Otorhinolaryngol(1989)[2759784]; J Clin Lab Immunol(1989)[2534928]; Pathology(1989)[2812880]; Am Rev Respir Dis(1989)[2930067]; Medicina (B Aires)(1989)[2698435]; Pediatr Infect Dis J(1989)[2812912]; Nihon Kyobu Shikkan Gakkai Zasshi(1989)[2693782]; Ann Med Interne (Paris)(1989)[2662856]; Am J Med(1989)[2729338]; Cancer(1990)[2364370]; Semin Respir Infect(1990)[2188317]; Pediatr Pathol(1990)[2235766]; Nihon Kyobu Geka Gakkai Zasshi(1990)[2290059]; P N G Med J(1990)[2080675]; Nihon Kyobu Shikkan Gakkai Zasshi(1990)[2355700]; Kansenshogaku Zasshi(1990)[2338508]; Nihon Kyobu Shikkan Gakkai Zasshi(1990)[2355706]; Cas Lek Cesk(1990)[2369754]; Kansenshogaku Zasshi(1990)[2335751]; J Antimicrob Chemother(1990)[2312445]; Semin Respir Infect(1990)[2255806]; Rev Infect Dis(1990)[2237109]; Zhonghua Jie He He Hu Xi Za Zhi(1990)[2128219]; Pathol Biol (Paris)(1990)[2385449]; Kokyu To Junkan(1990)[2236963]; Jpn J Antibiot(1990)[2112207]; AJR Am J Roentgenol(1990)[2117371]; Kekkaku(1990)[2077264]; Pneumonol Pol(1990)[2216912]; J Assoc Physicians India(1990)[2380133]; Chest(1990)[2323247]; Pneumologie(1990)[2367437]; Am J Dis Child(1990)[2396617]; Rev Clin Esp(1990)[2247680]; Medicine (Baltimore)(1990)[2205784]; Kansenshogaku Zasshi(1990)[2243193]; Agressologie(1990)[2089979]; Respir Med(1990)[1699254]; Diabetes Care(1990)[2209323]; No Shinkei Geka(1990)[2359479]; Am J Pediatr Hematol Oncol(1990)[2240481]; Kansenshogaku Zasshi(1990)[2243194]; Nihon Hinyokika Gakkai Zasshi(1990)[2273697]; Med Klin (Munich)(1990)[2377146]; Rinsho Ketsueki(1991)[2027237]; Clin Ther(1991)[1799921]; Singapore Med J(1991)[2017709]; Rev Infect Dis(1991)[1925279]; Pediatr Emerg Care(1991)[1788120]; Thorax(1991)[1907034]; Int Orthop(1991)[1743834]; Monatsschr Kinderheilkd(1991)[2072965]; Enferm Infecc Microbiol Clin(1991)[1863618]; Eur Respir J(1991)[1889504]; Bull Soc Pathol Exot(1991)[1666982]; Clin Ther(1991)[1709390]; Am Rev Respir Dis(1991)[1859056]; Chemotherapy(1991)[1884650]; Jpn J Antibiot(1991)[1652653]; Orv Hetil(1991)[1861853]; Indian Pediatr(1991)[1748514]; Kansenshogaku Zasshi(1991)[1783810]; Kyobu Geka(1991)[1921004]; Pediatr Infect Dis J(1991)[2041665]; Chest(1991)[1864118]; Ann Pediatr (Paris)(1991)[1746849]; Pediatrics(1991)[1984618]; AJR Am J Roentgenol(1991)[2028898]; Kansenshogaku Zasshi(1991)[1761893]; Clin Ther(1991)[1790546]; J Antimicrob Chemother(1991)[1761451]; Kans Med(1991)[2002618]; Acta Paediatr Jpn(1992)[1621520]; Nihon Kyobu Shikkan Gakkai Zasshi(1992)[1405100]; Am J Med Sci(1992)[1539610]; Intern Med(1992)[1504436]; Nihon Naika Gakkai Zasshi(1992)[1453072]; Eur Respir J(1992)[1486973]; West J Med(1992)[1475945]; Respir Med(1992)[1565823]; Nihon Kyobu Geka Gakkai Zasshi(1992)[1593172]; Nihon Kyobu Shikkan Gakkai Zasshi(1992)[1318432]; Chest(1992)[1643942]; East Afr Med J(1992)[1505392]; South Med J(1992)[1411720]; Nihon Rinsho(1992)[1507430]; East Afr Med J(1992)[1473506]; Ann Pediatr (Paris)(1992)[1456675]; East Afr Med J(1992)[1505400]; Cent Afr J Med(1992)[1394392]; Bol Med Hosp Infant Mex(1992)[1449625]; Am J Dis Child(1992)[1496945]; Schweiz Med Wochenschr(1992)[1738821]; Postgrad Med J(1992)[1287613]; Ann Thorac Surg(1992)[1570995]; Zhonghua Nei Ke Za Zhi(1992)[1303851]; Kansenshogaku Zasshi(1992)[1624830]; Nihon Rinsho(1992)[1507434]; J Hosp Infect(1992)[1362749]; Burns(1992)[1418512]; S Afr Med J(1992)[1448709]; Intensive Care Med(1992)[1386615]; Kyobu Geka(1992)[1602688]; N Y State J Med(1992)[1518586]; Br Med Bull(1992)[1281036]; Chest(1992)[1729070]; J Am Geriatr Soc(1992)[1401689]; Kansenshogaku Zasshi(1992)[1431388]; Arq Bras Cardiol(1992)[1340713]; Kansenshogaku Zasshi(1992)[1402095]; Pediatr Infect Dis J(1992)[1461693]; Ann Trop Paediatr(1992)[1280041]; J Burn Care Rehabil(1992)[1429817]; Zhonghua Yi Xue Za Zhi(1992)[1338524]; Am Rev Respir Dis(1992)[1731595]; Gan To Kagaku Ryoho(1992)[1371046]; Rev Latinoam Microbiol(1993)[8209107]; Enferm Infecc Microbiol Clin(1993)[8481442]; Kansenshogaku Zasshi(1993)[7691973]; Pneumonol Alergol Pol(1993)[8148761]; J Trop Pediatr(1993)[8492372]; Respir Med(1993)[8265842]; Semin Respir Infect(1993)[8372274]; Zhonghua Yi Xue Za Zhi (Taipei)(1993)[8402370]; Arch Dis Child(1993)[8257181]; Kansenshogaku Zasshi(1993)[8294769]; Intern Med(1993)[8358116]; Nihon Kyobu Shikkan Gakkai Zasshi(1993)[8255025]; Ryumachi(1993)[8316908]; Clin Infect Dis(1993)[8286635]; Clin Rheumatol(1993)[8258248]; Chest(1993)[8252964]; Pediatrics(1993)[8424026]; J Trauma(1993)[8355313]; Chest(1993)[8222797]; J Hosp Infect(1993)[7905892]; Ann Fr Anesth Reanim(1993)[8338270]; Med J Aust(1993)[8487685]; Thorax(1993)[8236076]; Rev Pneumol Clin(1993)[8296141]; Drug Investig(1993)[32287509]; Clin Infect Dis(1993)[8218696]; Anaesthesist(1993)[8250207]; Intensive Care Med(1993)[8408937]; AJR Am J Roentgenol(1993)[8352125]; Clin Invest Med(1993)[8467579]; West Afr J Med(1994)[8080838]; Am J Infect Control(1994)[7695112]; Am J Respir Crit Care Med(1994)[8004324]; Am J Respir Crit Care Med(1994)[7952612]; Kansenshogaku Zasshi(1994)[8151148]; Scand J Infect Dis Suppl(1994)[8047855]; Nihon Jinzo Gakkai Shi(1994)[8139151]; West Afr J Med(1994)[7841101]; Lung Cancer(1994)[7812701]; An Med Interna(1994)[7772688]; Ethiop Med J(1994)[8033877]; Med Clin North Am(1994)[8078378]; Chest(1994)[8131547]; Semin Respir Infect(1994)[7831542]; Semin Respir Infect(1994)[7831538]; Acta Paediatr(1994)[7981562]; South Med J(1994)[8153775]; Ryoikibetsu Shokogun Shirizu(1994)[8152085]; Kansenshogaku Zasshi(1994)[8051440]; Semin Respir Infect(1994)[7831539]; Am J Surg(1994)[8135314]; Postgrad Med J(1994)[8183783]; Pneumologie(1994)[8183862]; J Gen Intern Med(1994)[7853075]; Infection(1994)[7927832]; Clin Infect Dis(1994)[8086544]; Can J Infect Dis(1994)[22346488]; Eur Respir J(1994)[7511540]; Kansenshogaku Zasshi(1994)[7996025]; Arch Bronconeumol(1994)[8025784]; Nihon Kyobu Shikkan Gakkai Zasshi(1994)[7799554]; Intensive Care Med(1994)[8201105]; Nihon Rinsho(1994)[8126901]; Arch Intern Med(1994)[7979856]; J Chemother(1994)[7799056]; Arch Intern Med(1994)[8092913]; Dtsch Med Wochenschr(1994)[8070330]; Thorax(1994)[8153944]; Kansenshogaku Zasshi(1994)[7829904]; Intern Med(1994)[7949649]; Scand J Infect Dis(1995)[8685635]; Eur Respir J(1995)[8620965]; J Chemother(1995)[8568541]; Vestn Ross Akad Med Nauk(1995)[7580418]; J Obstet Gynecol Neonatal Nurs(1995)[7562139]; Pediatr Pulmonol(1995)[8570307]; Enferm Infecc Microbiol Clin(1995)[7654835]; Clin Infect Dis(1995)[7548560]; Neth J Med(1995)[7643944]; Clin Pediatr (Phila)(1995)[7720324]; Kansenshogaku Zasshi(1995)[8708405]; J Antimicrob Chemother(1995)[8543488]; East Afr Med J(1995)[8689973]; Jpn J Antibiot(1995)[7745805]; Kansenshogaku Zasshi(1995)[7751746]; Rev Rhum Engl Ed(1995)[7788320]; Arch Intern Med(1995)[7763121]; Neth J Med(1995)[8569936]; J Trop Pediatr(1995)[7636941]; J Chemother(1995)[8568539]; Kyobu Geka(1995)[7474582]; Malays J Pathol(1995)[8907000]; Kansenshogaku Zasshi(1995)[7561255]; Acta Otorhinolaryngol Belg(1995)[7484143]; Nihon Naibunpi Gakkai Zasshi(1995)[7750628]; Chest(1995)[7750325]; Kyobu Geka(1995)[7745871]; South Med J(1995)[7732454]; Crit Care Med(1995)[7600827]; Microb Drug Resist(1995)[9158751]; Zhonghua Min Guo Wei Sheng Wu Ji Mian Yi Xue Za Zhi(1995)[9774999]; J Clin Forensic Med(1995)[15335635]; Eur J Emerg Med(1995)[9422174]; Can J Infect Dis(1995)[22416210]; Thorax(1995)[8553294]; JAMA(1995)[7637145]; Can J Infect Dis(1995)[22550412]; Gan To Kagaku Ryoho(1995)[7661584]; Med J Aust(1995)[7565207]; Eur J Surg(1995)[7772634]; Chest(1995)[7656634]; Arch Intern Med(1995)[7618989]; Infect Dis Clin North Am(1995)[7769221]; Pneumonol Alergol Pol(1995)[8520552]; Intensive Care Med(1996)[8844235]; Clin Infect Dis(1996)[8783710]; Pediatr Infect Dis J(1996)[8970218]; Pneumologie(1996)[9019751]; Presse Med(1996)[8958873]; Chemotherapy(1996)[8861533]; Nihon Geka Gakkai Zasshi(1996)[8774812]; Semin Respir Infect(1996)[8776779]; Semin Respir Infect(1996)[8776781]; Pediatr Emerg Care(1996)[8858655]; Rev Mal Respir(1996)[8711237]; Intern Med(1996)[9030999]; Respir Med(1996)[8984527]; Eur J Clin Microbiol Infect Dis(1996)[8839645]; Harefuah(1996)[8794638]; Arch Bronconeumol(1996)[8948878]; Monaldi Arch Chest Dis(1996)[9009626]; Br J Dermatol(1996)[8736347]; Pediatr Pulmonol(1996)[8905884]; Nihon Ronen Igakkai Zasshi(1996)[8868121]; Clin Infect Dis(1996)[8842271]; Int J Antimicrob Agents(1996)[18611723]; Radiol Clin North Am(1996)[8539353]; Laeknabladid(1996)[20065391]; Clin Infect Dis(1996)[8824970]; Arch Pediatr(1996)[8762954]; Postgrad Med J(1996)[8944211]; Aust N Z J Surg(1996)[8634044]; Pediatrics(1996)[8784356]; AIDS(1996)[8970683]; Bacteriol Virusol Parazitol Epidemiol(1996)[8963117]; Enferm Infecc Microbiol Clin(1996)[8695681]; HNO(1996)[8852801]; Pathol Biol (Paris)(1996)[8758486]; Arch Pediatr(1996)[8952771]; Am J Respir Crit Care Med(1997)[9001306]; Infect Control Hosp Epidemiol(1997)[9247830]; Kansenshogaku Zasshi(1997)[9339624]; Curr Opin Pulm Med(1997)[9193863]; Med Clin (Barc)(1997)[9333692]; Acta Med Austriaca(1997)[9206930]; Ugeskr Laeger(1997)[9340883]; East Afr Med J(1997)[9185416]; Clin Infect Dis(1997)[9332511]; Nihon Kyobu Shikkan Gakkai Zasshi(1997)[9396255]; Ann Ital Med Int(1997)[9409949]; Ann Acad Med Singap(1997)[9494663]; Scand J Infect Dis(1997)[9181654]; Rev Med Chir Soc Med Nat Iasi(1997)[10756729]; Ann Acad Med Singap(1997)[9494674]; Radiol Med(1997)[9280934]; Kansenshogaku Zasshi(1997)[9248265]; Kansenshogaku Zasshi(1997)[9339633]; Radiol Med(1997)[9280932]; Respiration(1997)[9154675]; J Okla State Med Assoc(1997)[9299893]; Ann Dermatol Venereol(1997)[9740863]; Antibiot Khimioter(1997)[9412402]; Pneumonol Alergol Pol(1997)[9760790]; Mem Inst Oswaldo Cruz(1997)[9566230]; Pneumologie(1997)[9173416]; Head Neck(1997)[9243263]; Kansenshogaku Zasshi(1997)[9394563]; Can J Infect Dis(1997)[22346528]; N Z Med J(1997)[9315030]; Eur J Epidemiol(1997)[9324219]; Thorax(1997)[9039234]; Nihon Rinsho(1997)[9360392]; Infect Control Hosp Epidemiol(1997)[9397368]; Rev Clin Esp(1997)[9558601]; Infect Dis Clin North Am(1998)[9779381]; Antimicrob Agents Chemother(1998)[9559773]; Br J Theatre Nurs(1998)[9677888]; Crit Care Clin(1998)[9561819]; Drugs Today (Barc)(1998)[15094862]; N Engl J Med(1998)[9882209]; Clin Infect Dis(1998)[9524833]; Chest(1998)[9792588]; Nihon Naika Gakkai Zasshi(1998)[9549312]; Pediatr Infect Dis J(1998)[9535250]; Kansenshogaku Zasshi(1998)[9545687]; J Chemother(1998)[9531074]; J Thorac Imaging(1998)[9799134]; Emerg Infect Dis(1998)[9716961]; Eur J Clin Microbiol Infect Dis(1998)[9832263]; Medicine (Baltimore)(1998)[9465863]; J Hosp Infect(1998)[9868618]; Intensive Care Med(1998)[9885888]; Eur Respir J(1998)[9701424]; Pathol Biol (Paris)(1998)[9769865]; Am J Respir Crit Care Med(1998)[9563735]; Arch Intern Med(1998)[9818796]; Clin Exp Obstet Gynecol(1998)[9987566]; Dakar Med(1998)[10797962]; Clin Infect Dis(1998)[9770140]; Pediatr Pulmonol(1998)[9888211]; QJM(1998)[9519211]; J Heart Lung Transplant(1998)[9588587]; Hinyokika Kiyo(1998)[9546132]; Scand Cardiovasc J(1998)[9802144]; Arch Bronconeumol(1998)[9803276]; Arch Pediatr(1998)[10223157]; Kansenshogaku Zasshi(1998)[9796188]; Med Clin (Barc)(1998)[9717164]; Minerva Anestesiol(1998)[9951272]; Am J Cardiol(1998)[9761092]; Eur J Clin Microbiol Infect Dis(1998)[9758274]; Am J Med(1998)[9684654]; Eur J Surg(1998)[9696441]; Infect Control Hosp Epidemiol(1998)[9475347]; J Clin Microbiol(1998)[9466751]; Int J STD AIDS(1998)[9874118]; J Cardiovasc Surg (Torino)(1998)[9537546]; Ann Thorac Surg(1998)[9456102]; Intensive Care Med(1998)[9840237]; Pneumoftiziologia(1998)[10386145]; Presse Med(1998)[9819592]; J Hosp Infect(1998)[9602976]; Am J Respir Crit Care Med(1998)[9476869]; Chest(1998)[9596298]; Am J Respir Crit Care Med(1998)[9731019]; Presse Med(1998)[9793046]; Chest(1998)[9580099]; Zhonghua Yi Xue Za Zhi (Taipei)(1998)[9830236]; Pneumologie(1998)[9885511]; Zh Mikrobiol Epidemiol Immunobiol(1998)[9783407]; Med Clin (Barc)(1998)[9922954]; Intensive Care Med(1998)[9539080]; Diagn Microbiol Infect Dis(1998)[9934546]; Clin Chest Med(1999)[10516899]; Emerg Infect Dis(1999)[10341185]; Semin Respir Infect(1999)[10638512]; Jpn J Thorac Cardiovasc Surg(1999)[10496060]; J Trop Pediatr(1999)[10467836]; Jpn J Clin Oncol(1999)[10073152]; QJM(1999)[10627862]; J Med Assoc Thai(1999)[10730534]; Clin Infect Dis(1999)[10589891]; Semin Respir Infect(1999)[10197394]; Chest(1999)[10084458]; Sante(1999)[10477402]; Am Surg(1999)[10432077]; Compr Ther(1999)[9987589]; Clin Infect Dis(1999)[10524959]; Aust N Z J Med(1999)[10200815]; J Microbiol Immunol Infect(1999)[10650491]; Kansenshogaku Zasshi(1999)[10535263]; Am J Perinatol(1999)[10614699]; Diagn Microbiol Infect Dis(1999)[10091036]; J Pediatr (Rio J)(1999)[14685562]; Enferm Infecc Microbiol Clin(1999)[10396086]; Int J Pediatr Otorhinolaryngol(1999)[10577771]; Antibiot Khimioter(1999)[10095919]; Intensive Care Med(1999)[10551961]; Indian J Pediatr(1999)[10798147]; Acta Paediatr Taiwan(1999)[10910624]; Am J Respir Crit Care Med(1999)[10430736]; Crit Care Med(1999)[10321654]; Rev Clin Esp(1999)[10216394]; Chest(1999)[10084452]; J Infect Dis(1999)[10558935]; Kansenshogaku Zasshi(1999)[10423947]; Eur J Cardiothorac Surg(1999)[10371127]; Infez Med(1999)[12748443]; Presse Med(1999)[10506875]; RN(1999)[10205564]; Semin Thromb Hemost(1999)[10357089]; Medicina (B Aires)(1999)[10752217]; Crit Care Med(1999)[10470744]; Singapore Med J(1999)[10572489]; Schweiz Med Wochenschr(1999)[10568234]; Singapore Med J(1999)[10487085]; Crit Care Med(1999)[10362409]; Pneumonol Alergol Pol(1999)[10481526]; Pediatr Infect Dis J(1999)[10530574]; Chest(1999)[9925081]; Ryoikibetsu Shokogun Shirizu(1999)[10088411]; Transplantation(1999)[10232564]; Postgrad Med(1999)[10223091]; Acta Radiol(1999)[10080731]; Eur J Clin Microbiol Infect Dis(1999)[10691195]; Pathol Biol (Paris)(1999)[10418017]; Chest(1999)[10084487]; Braz J Infect Dis(1999)[11097714]; Am J Respir Crit Care Med(2000)[10988092]; Leg Med (Tokyo)(2000)[12935447]; Am J Perinatol(2000)[11142393]; Am Surg(2000)[11149581]; Diagn Microbiol Infect Dis(2000)[10863106]; Tidsskr Nor Laegeforen(2000)[10851938]; Nihon Kokyuki Gakkai Zasshi(2000)[10774175]; Ugeskr Laeger(2000)[10860428]; Eur J Pediatr(2000)[11014470]; Am J Surg(2000)[10802251]; Am J Surg(2000)[10874100]; Am J Surg(2000)[10802257]; Tohoku J Exp Med(2000)[11211316]; Eur J Clin Microbiol Infect Dis(2000)[11205629]; Med Oncol(2000)[10871816]; Clin Infect Dis(2000)[11073748]; Semin Respir Crit Care Med(2000)[16088716]; Semin Respir Crit Care Med(2000)[16088717]; Sante(2000)[10960805]; Ned Tijdschr Geneeskd(2000)[11048555]; Clin Infect Dis(2000)[11073777]; Curr Opin Pulm Med(2000)[11100967]; Kansenshogaku Zasshi(2000)[10835838]; Eur Respir J(2000)[11292126]; Clin Infect Dis(2000)[10913417]; Chemotherapy(2000)[10765030]; Zhonghua Jie He He Hu Xi Za Zhi(2000)[11778507]; Pediatrics(2000)[11099630]; Am J Surg(2000)[10802259]; Am J Respir Crit Care Med(2000)[10712318]; Diagn Microbiol Infect Dis(2000)[10794943]; Rev Med Chil(2000)[11008357]; Am J Health Syst Pharm(2000)[11057360]; Am J Manag Care(2000)[10977480]; Nihon Rinsho(2000)[11225327]; Am J Surg(2000)[10874106]; Int J Antimicrob Agents(2000)[11137406]; Przegl Epidemiol(2000)[11349588]; East Afr Med J(2000)[12862109]; Eur J Pediatr(2000)[11014469]; Pediatr Int(2000)[10881583]; Chest(2000)[10767233]; J Crit Care(2000)[11011820]; Can J Infect Dis(2000)[18159274]; Transplantation(2000)[10868641]; Crit Care Med(2000)[11057812]; Crit Care Med(2000)[10966244]; N Z Med J(2000)[10909937]; Chest(2000)[11115444]; N Z Med J(2000)[10894342]; Minerva Med(2000)[11084845]; Cochrane Database Syst Rev(2000)[10796836]; Infect Control Hosp Epidemiol(2000)[10782588]; Ann Transplant(2000)[11147024]; Curr Infect Dis Rep(2000)[11095858]; Arch Bronconeumol(2000)[11004983]; J Formos Med Assoc(2000)[11155749]; Chest(2000)[11115467]; J Trop Pediatr(2000)[11191152]; Chest(2000)[10807834]; AJR Am J Roentgenol(2000)[10882278]; J Assoc Physicians India(2000)[11310380]; Infect Control Hosp Epidemiol(2000)[10968716]; J Chemother(2001)[11936369]; Antibiot Khimioter(2001)[11573325]; Arch Pediatr(2001)[11582921]; J Dermatol(2001)[11732724]; Rev Esp Quimioter(2001)[11856984]; Med J Malaysia(2001)[11732071]; Pediatr Crit Care Med(2001)[12797868]; J Chemother(2001)[11589485]; J Hosp Infect(2001)[11289777]; East Mediterr Health J(2001)[15332774]; Nihon Kokyuki Gakkai Zasshi(2001)[11729685]; Chest(2001)[11171773]; Ther Umsch(2001)[11695094]; Clin Infect Dis(2001)[11320452]; Semin Respir Crit Care Med(2001)[16088683]; Diagn Microbiol Infect Dis(2001)[11687309]; Rev Pneumol Clin(2001)[11353919]; Arch Intern Med(2001)[11525709]; Fortschr Med Orig(2001)[11935653]; J Am Geriatr Soc(2001)[11380747]; Altern Ther Health Med(2001)[11347281]; Drugs Aging(2001)[11302286]; Unfallchirurg(2001)[11381766]; Ther Umsch(2001)[11695092]; Indian J Pediatr(2001)[11770243]; Heart(2001)[11711482]; Burns(2001)[11451607]; Acta Diabetol(2001)[11757805]; Am J Respir Crit Care Med(2001)[11254518]; Intern Med J(2001)[11480485]; Can Assoc Radiol J(2001)[11780550]; Eur J Clin Microbiol Infect Dis(2001)[11837636]; J Med Liban(2001)[12243418]; Kansenshogaku Zasshi(2001)[11357318]; Int J Antimicrob Agents(2001)[11165120]; Pol Merkur Lekarski(2001)[11770318]; Am J Epidemiol(2001)[11207152]; Crit Care Med(2001)[11292880]; Postgrad Med(2001)[19667559]; Am J Med(2001)[11755439]; Clin Exp Dermatol(2001)[11722449]; Arch Pediatr(2001)[11582915]; Am J Infect Control(2001)[11172313]; East Afr Med J(2001)[11921576]; Cochrane Database Syst Rev(2001)[11687002]; Crit Care Med(2001)[11246310]; Chest(2001)[11399696]; Indian J Chest Dis Allied Sci(2001)[11529434]; Acta Paediatr(2001)[11236037]; Am J Med(2001)[11755441]; Crit Care Med(2001)[11801813]; Semin Respir Infect(2001)[11562899]; Indian J Pediatr(2001)[11563247]; CMAJ(2001)[11468949]; Crit Care(2001)[11353934]; J Pediatr Surg(2001)[11227003]; J Neurol(2001)[11757959]; Thorax(2001)[11312407]; Drug Ther Bull(2001)[11471517]; Swiss Med Wkly(2001)[11875753]; Diagn Microbiol Infect Dis(2001)[11248523]; Crit Care Med(2001)[11373418]; Braz J Infect Dis(2001)[11506775]; Pediatr Infect Dis J(2001)[11368105]; Arch Dis Child(2001)[11159295]; Minerva Anestesiol(2001)[11731755]; Rev Cubana Med Trop(2002)[15849942]; J Hosp Infect(2002)[12392904]; Semin Respir Infect(2002)[11891518]; J Chemother(2002)[12583547]; Ann Fr Anesth Reanim(2002)[12078429]; Rinsho Byori(2002)[12187704]; J Dermatol(2002)[11837574]; J Fam Pract(2002)[12184969]; Arch Dis Child(2002)[12244005]; Emerg Infect Dis(2002)[11749750]; Infect Control Hosp Epidemiol(2002)[11918125]; Jpn J Thorac Cardiovasc Surg(2002)[12166265]; Diabetes Metab(2002)[12442071]; Pediatr Infect Dis J(2002)[12182396]; J Microbiol Immunol Infect(2002)[12099332]; Am J Respir Crit Care Med(2002)[12379545]; Paediatr Drugs(2002)[11888355]; Southeast Asian J Trop Med Public Health(2002)[12118460]; Geriatrics(2002)[11899549]; Semin Respir Infect(2002)[11891522]; Lancet(2002)[11888586]; Clin Infect Dis(2002)[12228818]; N Engl J Med(2002)[12181400]; Pediatr Pulmonol(2002)[11836802]; Eur Radiol(2002)[11870440]; Am J Respir Crit Care Med(2002)[11934711]; Nihon Geka Gakkai Zasshi(2002)[12599923]; J Cardiovasc Surg (Torino)(2002)[12483175]; J Am Geriatr Soc(2002)[12121517]; J Infect Chemother(2002)[11957128]; Scand J Infect Dis(2002)[12030399]; Postgrad Med J(2002)[12151652]; Unfallchirurg(2002)[11995227]; Acta Paediatr Taiwan(2002)[12632784]; Otol Neurotol(2002)[12170160]; Scand J Infect Dis(2002)[12477329]; Southeast Asian J Trop Med Public Health(2002)[12757224]; Pediatr Infect Dis J(2002)[12182377]; Int J Clin Pract Suppl(2002)[12014853]; Diagn Microbiol Infect Dis(2002)[12493178]; J Pediatr(2002)[12006957]; Int J Infect Dis(2002)[12044296]; Rev Med Chil(2002)[12611238]; Arch Pediatr(2002)[12108316]; J Antimicrob Chemother(2002)[12205063]; Int J Infect Dis(2002)[12718823]; East Afr Med J(2002)[12643233]; Kansenshogaku Zasshi(2002)[11974888]; Recenti Prog Med(2002)[12355983]; Gerodontology(2002)[12542215]; Tidsskr Nor Laegeforen(2002)[12448252]; Crit Care Med(2002)[12163786]; Inflamm Res(2002)[12056515]; West Indian Med J(2002)[12089869]; Southeast Asian J Trop Med Public Health(2002)[12236437]; Clin Ther(2002)[12117077]; Chest(2002)[12171848]; J Pediatr Surg(2002)[12378443]; Infection(2003)[14556063]; Rev Prat(2003)[14558265]; Anaesthesist(2003)[14504808]; Am J Respir Med(2003)[14720021]; Presse Med(2003)[12754444]; Arch Dis Child(2003)[14612371]; Clin Infect Dis(2003)[14523769]; Pediatr Infect Dis J(2003)[12613460]; Braz J Infect Dis(2003)[12807691]; Kyobu Geka(2003)[12607257]; Intensive Care Med(2003)[12677369]; Am J Respir Crit Care Med(2003)[12689848]; Kyobu Geka(2003)[12854472]; Diagn Microbiol Infect Dis(2003)[12730000]; Ren Fail(2003)[12803514]; Rev Mal Respir(2003)[12910140]; Arch Pediatr(2003)[12907071]; J Trop Pediatr(2003)[12729288]; Wien Klin Wochenschr(2003)[12674685]; Nurs Stand(2003)[14649189]; Am J Infect Control(2003)[12762292]; Yonsei Med J(2003)[12619189]; Semin Respir Crit Care Med(2003)[16088523]; Rev Mal Respir(2003)[12910141]; Infect Control Hosp Epidemiol(2003)[14510249]; Przegl Lek(2003)[14575024]; Int J Med Microbiol(2003)[14503795]; Braz J Infect Dis(2003)[14499046]; Acta Microbiol Pol(2003)[14594401]; Saudi Med J(2003)[14710292]; Medicina (Kaunas)(2003)[12695638]; Am J Respir Crit Care Med(2003)[12433668]; Infect Control Hosp Epidemiol(2003)[14649777]; Cochrane Database Syst Rev(2003)[12917916]; Metabolism(2003)[14577062]; Eur Respir J(2003)[14680072]; Chest(2003)[14605050]; Int J Infect Dis(2003)[12839710]; Asian Cardiovasc Thorac Ann(2003)[12692031]; Diagn Microbiol Infect Dis(2003)[12729995]; Semin Respir Crit Care Med(2003)[16088583]; Intensive Care Med(2003)[12904849]; Pneumonol Alergol Pol(2003)[12959021]; Curr Opin Infect Dis(2003)[12821819]; Arch Bronconeumol(2003)[12890400]; Chest(2003)[12853512]; Arch Pediatr(2003)[12907073]; J Pediatr Hematol Oncol(2003)[12544773]; Arch Bronconeumol(2003)[12975070]; Sao Paulo Med J(2003)[12920475]; Ned Tijdschr Geneeskd(2003)[12814021]; J Hosp Infect(2003)[12495679]; Antimicrob Agents Chemother(2003)[12878545]; Harefuah(2003)[12754875]; Med Princ Pract(2003)[12634471]; Intensive Care Med(2003)[12898002]; Surg Infect (Larchmt)(2003)[12744764]; Pediatr Pulmonol(2003)[12772225]; Intern Med(2003)[12583614]; Hunan Yi Ke Da Xue Xue Bao(2003)[14653088]; Zhonghua Er Ke Za Zhi(2003)[14746685]; Crit Care Med(2003)[14530765]; Turk J Pediatr(2003)[14696807]; Nihon Rinsho(2003)[14619435]; Rev Hosp Clin Fac Med Sao Paulo(2003)[14666322]; Pediatr Infect Dis J(2003)[12913766]; Postgrad Med(2003)[12647477]; Chest(2003)[12628886]; J Microbiol Immunol Infect(2004)[15060682]; Clin Genet(2004)[15151509]; Curr Opin Pulm Med(2004)[15514493]; J Trop Pediatr(2004)[15357563]; Clin Infect Dis(2004)[15356814]; Di Yi Jun Yi Da Xue Xue Bao(2004)[15604082]; Kyobu Geka(2004)[15366570]; Recenti Prog Med(2004)[15032335]; Anesthesiol Clin North Am(2004)[15325711]; Eur Respir J(2004)[15358692]; J Int Med Res(2004)[14997711]; Scand J Infect Dis(2004)[15061671]; Antibiot Khimioter(2004)[15344392]; J Microbiol Immunol Infect(2004)[15060683]; Clin Infect Dis(2004)[15095210]; Chest(2004)[15249482]; Kansenshogaku Zasshi(2004)[15628531]; Clin Lab Med(2004)[15177847]; Pediatr Pulmonol(2004)[15334503]; Clin Microbiol Infect(2004)[15191383]; Neurosurg Focus(2004)[15636567]; J Cyst Fibros(2004)[15463902]; In Vivo(2004)[15011754]; Assist Inferm Ric(2004)[15152377]; Eur J Clin Microbiol Infect Dis(2004)[15558346]; Pathol Biol (Paris)(2004)[15465267]; Clin Microbiol Rev(2004)[14726455]; Acta Paediatr(2004)[15244228]; Scand J Infect Dis(2004)[15198183]; Am J Respir Crit Care Med(2004)[15242840]; Interact Cardiovasc Thorac Surg(2004)[17670247]; Med Mal Infect(2004)[15612359]; Orv Hetil(2004)[15384746]; Korean J Intern Med(2004)[15053046]; Med J Aust(2004)[15310264]; Zhonghua Lao Dong Wei Sheng Zhi Ye Bing Za Zhi(2004)[15033031]; BMC Pulm Med(2004)[15109397]; Indian J Chest Dis Allied Sci(2004)[14870864]; Infect Control Hosp Epidemiol(2004)[15484803]; Crit Care Med(2004)[15071391]; P R Health Sci J(2004)[15125215]; J Infect Chemother(2004)[15614461]; Southeast Asian J Trop Med Public Health(2004)[15691151]; Kyobu Geka(2004)[15202275]; Can J Infect Dis(2004)[18159439]; J Paediatr Child Health(2004)[15265191]; Eur J Cardiothorac Surg(2004)[15145012]; Acta Paediatr(2004)[15918230]; Kyobu Geka(2004)[15553037]; Zhonghua Jie He He Hu Xi Za Zhi(2004)[15130324]; Int J Pediatr Otorhinolaryngol(2004)[15126016]; Resuscitation(2004)[14987786]; Anasthesiol Intensivmed Notfallmed Schmerzther(2004)[14740311]; Acta Paediatr Taiwan(2004)[15868807]; Curr Opin Pulm Med(2004)[15071367]; Probl Tuberk Bolezn Legk(2004)[15315123]; J Trauma(2004)[14960971]; J Ayub Med Coll Abbottabad(2004)[15762062]; Infect Control Hosp Epidemiol(2004)[15484800]; Chest(2004)[15249447]; Medicina (B Aires)(2004)[15338978]; Otolaryngol Head Neck Surg(2004)[15577783]; Zhongguo Wei Zhong Bing Ji Jiu Yi Xue(2004)[15355618]; Intensive Care Med(2004)[14714 |
| *Staphylococcus aureus (MRSA)* | Established pathogen | 3055 | **established** | J Radiol Electrol Arch Electr Medicale(1953)[13097495]; J Fr Med Chir Thorac(1953)[13096683]; Arch Kinderheilkd(1954)[13208213]; J Radiol Electrol Arch Electr Medicale(1954)[13184505]; Pediatrie(1954)[13166471]; Poumon(1954)[13155306]; Poumon(1954)[13155305]; J Fr Med Chir Thorac(1954)[13211961]; Poumon(1954)[13155307]; Arch Kinderheilkd(1955)[13259604]; Alger Medicale(1955)[14398593]; Tuberkulosearzt(1955)[13291497]; Can Serv Med J(1955)[14352158]; Sem Hop(1955)[14396400]; Presse Med (1893)(1955)[14395134]; Toulouse Med(1955)[13311923]; Gyermekgyogyaszat(1956)[13318480]; Policlinico Prat(1956)[13353967]; Lancet(1956)[13320874]; Surg Forum(1956)[13391415]; Lancet(1956)[13320873]; Med J Aust(1956)[13321222]; Lyon Med(1956)[13308252]; Policlinico Prat(1956)[13310293]; J Pediatr(1957)[13417022]; Lancet(1958)[13564806]; Schweiz Med Wochenschr(1958)[13568733]; Zhonghua Nei Ke Za Zhi(1959)[13652275]; Harper Hosp Bull(1960)[13798058]; Harefuah(1960)[14435167]; Schweiz Med Wochenschr(1961)[14471377]; Orv Hetil(1967)[5634303]; N Engl J Med(1968)[4232865]; JAMA(1970)[5467891]; Am J Med Sci(1972)[4486504]; J Indian Med Assoc(1972)[4644909]; J Pediatr(1974)[4820707]; Bol Med Hosp Infant Mex(1974)[4425554]; Am J Dis Child(1975)[123704]; Nouv Presse Med(1975)[1101214]; Scand J Infect Dis(1975)[1101372]; Rev Ig Bacteriol Virusol Parazitol Epidemiol Pneumoftiziol Bacteriol Virusol and Parazitol Epidemiol(1976)[134444]; Pediatrics(1976)[934785]; Vestn Akad Med Nauk SSSR(1976)[1266374]; Am J Med(1976)[1048860]; Ann Sclavo(1976)[13744]; Srp Arh Celok Lek(1977)[616662]; J Clin Pathol(1977)[325018]; J Fam Pract(1977)[320285]; Cesk Epidemiol Mikrobiol Imunol(1977)[144030]; Am J Med(1977)[871128]; Pediatrics(1978)[32513]; J Infect Dis(1978)[24669]; West J Med(1978)[706357]; Arch Fr Pediatr(1978)[637669]; Pediatrics(1978)[634675]; Arch Dis Child(1979)[475415]; Gig Sanit(1979)[527832]; P N G Med J(1979)[299332]; Postgrad Med J(1979)[44909]; Arch Intern Med(1979)[32855]; Bol Med Hosp Infant Mex(1979)[313800]; Pneumonol Pol(1979)[119214]; Pediatrics(1980)[6990374]; Semin Roentgenol(1980)[7355303]; Mayo Clin Proc(1980)[7442320]; Sov Med(1980)[6998021]; Br Med J(1980)[7448549]; Infection(1980)[7399717]; Vrach Delo(1980)[7405165]; Grudn Khir(1980)[7372183]; Ann Intern Med(1980)[6904159]; Zhonghua Jie He He Hu Xi Xi Ji Bing Za Zhi(1980)[7227119]; Zhonghua Jie He He Hu Xi Xi Ji Bing Za Zhi(1980)[7227118]; MMW Munch Med Wochenschr(1981)[6785603]; Tijdschr Kindergeneeskd(1981)[7302937]; Monatsschr Kinderheilkd(1981)[7335093]; P N G Med J(1981)[6951351]; Ter Arkh(1981)[7027504]; Lab Delo(1981)[6170829]; Dtsch Med Wochenschr(1981)[7193562]; Thorax(1981)[7314031]; Med J Aust(1981)[7029225]; Chest(1981)[7471872]; Cancer(1981)[7226035]; Ir Med J(1981)[7319781]; Am J Dis Child(1982)[7091060]; Probl Gematol Pereliv Krovi(1982)[6953402]; Am J Trop Med Hyg(1982)[7102917]; J Thorac Cardiovasc Surg(1982)[7121040]; Ann Intern Med(1982)[7114628]; Pediatr Infect Dis(1982)[7177890]; J Infect(1982)[7185980]; Anaesthesist(1982)[7091639]; Pediatr Infect Dis(1982)[6755404]; Ann Pediatr (Paris)(1982)[7081902]; Arch Intern Med(1982)[7052006]; Rev Ig Bacteriol Virusol Parazitol Epidemiol Pneumoftiziol Pneumoftiziol(1982)[6296978]; Scand J Infect Dis(1982)[6925916]; South Med J(1983)[6823612]; Infect Control(1983)[6354957]; J Antimicrob Chemother(1983)[6352603]; Trop Doct(1983)[6679397]; Jpn J Antibiot(1983)[6348341]; Jpn J Antibiot(1983)[6425535]; Jpn J Antibiot(1983)[6348340]; Pediatr Med Chir(1983)[6647075]; Lab Delo(1983)[6194362]; Am J Infect Control(1983)[6349427]; Am J Hematol(1983)[6349334]; Clin Pediatr (Phila)(1983)[6839622]; Schweiz Med Wochenschr(1983)[6658426]; J Natl Med Assoc(1984)[6471117]; Pneumonol Pol(1984)[6398866]; Postgrad Med J(1984)[6709548]; Minerva Med(1984)[6709217]; J Clin Pathol(1984)[6368604]; J Trauma(1984)[6481828]; Monatsschr Kinderheilkd(1984)[6727895]; Am J Dis Child(1984)[6695867]; Jpn J Antibiot(1984)[6587132]; S Afr Med J(1984)[6710279]; S Afr Med J(1984)[6695248]; S Afr Med J(1984)[6495111]; Am J Med Sci(1984)[6610355]; Dis Mon(1984)[6386397]; Scand J Thorac Cardiovasc Surg(1984)[6719079]; Ann Fr Anesth Reanim(1984)[6476503]; An Esp Pediatr(1984)[6703532]; Pediatrics(1984)[6718113]; Infection(1985)[4055051]; Am Fam Physician(1985)[3883718]; Jpn J Antibiot(1985)[3937915]; J Infect(1985)[4031512]; Am J Med(1985)[3859208]; Ann Intern Med(1985)[2996410]; Ann Pediatr (Paris)(1985)[4051416]; Eur J Clin Microbiol(1985)[4018066]; Infect Control(1985)[3847402]; J Pediatr(1985)[3973782]; Am J Med(1985)[4014285]; Acta Chir Belg(1985)[4013584]; J Infect(1985)[3891869]; An Esp Pediatr(1986)[3706923]; Am J Epidemiol(1986)[3940431]; Zhonghua Jie He He Hu Xi Xi Ji Bing Za Zhi(1986)[3527607]; Infection(1986)[3759253]; South Med J(1986)[3486480]; Arch Intern Med(1986)[3516102]; Scand J Infect Dis Suppl(1986)[3103208]; N Z Med J(1986)[3456113]; Monatsschr Kinderheilkd(1986)[3748039]; Zhonghua Nei Ke Za Zhi(1986)[3743222]; J Trauma(1986)[3795301]; Pharm Weekbl Sci(1987)[3438152]; Medicine (Baltimore)(1987)[3626846]; Microbiologica(1987)[3695983]; Drug Intell Clin Pharm(1987)[3569027]; Am Rev Respir Dis(1987)[3310768]; Zhonghua Jie He He Hu Xi Za Zhi(1987)[3450416]; J Fam Pract(1987)[3546587]; Nihon Kyobu Shikkan Gakkai Zasshi(1987)[3449690]; Q J Med(1987)[3116595]; Arch Pathol Lab Med(1987)[3307685]; Infect Control(1987)[3643889]; Surg Gynecol Obstet(1987)[3660197]; Crit Care Med(1987)[3568713]; Postgrad Med(1987)[3615308]; Infect Control(1987)[3643888]; Jpn J Antibiot(1987)[3586339]; Rev Mal Respir(1987)[3671863]; Am J Dis Child(1987)[3673968]; Am J Med(1987)[3578359]; Zhong Xi Yi Jie He Za Zhi(1987)[3447764]; Pharm Weekbl Sci(1987)[3325930]; Infection(1987)[3301684]; Ugeskr Laeger(1987)[3451526]; Antimicrob Agents Chemother(1987)[3304156]; Vestn Khir Im I I Grek(1987)[3424535]; Ann Trop Paediatr(1987)[2441645]; Pediatr Radiol(1987)[2819815]; Pediatr Pulmonol(1987)[3501100]; Scand J Infect Dis(1987)[3441748]; Wiad Lek(1987)[3442018]; Semin Respir Infect(1987)[3321267]; Zentralbl Bakteriol Mikrobiol Hyg A(1987)[3115004]; Kansenshogaku Zasshi(1988)[3147308]; J Hyg Epidemiol Microbiol Immunol(1988)[3397528]; J Hosp Infect(1988)[2896723]; Am J Med Sci(1988)[3344761]; Crit Care Med(1988)[3277780]; J Hosp Infect(1988)[2896692]; Rev Ig Bacteriol Virusol Parazitol Epidemiol Pneumoftiziol Pneumoftiziol(1988)[2849190]; Scand J Infect Dis(1988)[3406669]; Arch Dis Child(1988)[3196056]; Z Erkr Atmungsorgane(1988)[3135674]; Pediatriia(1988)[3264401]; Nihon Geka Gakkai Zasshi(1988)[3146686]; Chest(1988)[3338299]; Pediatr Radiol(1988)[3387161]; Rev Mal Respir(1988)[2835800]; Southeast Asian J Trop Med Public Health(1988)[3227408]; Khirurgiia (Mosk)(1988)[3236705]; Monatsschr Kinderheilkd(1988)[3352613]; Rev Infect Dis(1988)[3353630]; Clin Chest Med(1988)[3044680]; Chest(1988)[3293940]; Am J Surg(1988)[3202266]; Zh Mikrobiol Epidemiol Immunobiol(1988)[3140545]; Med J Aust(1989)[2739613]; Med J Aust(1989)[2642592]; Zhonghua Zhong Liu Za Zhi(1989)[2550200]; Ann Pediatr (Paris)(1989)[2742314]; Jpn J Antibiot(1989)[2695657]; Klin Wochenschr(1989)[2545969]; Wiad Lek(1989)[2634313]; Trans R Soc Trop Med Hyg(1989)[2617633]; Nihon Kyobu Shikkan Gakkai Zasshi(1989)[2698422]; Rev Argent Microbiol(1989)[2748851]; J Hosp Infect(1989)[2575629]; An Med Interna(1989)[2491492]; Kinderarztl Prax(1989)[2786108]; Eur J Clin Microbiol Infect Dis(1989)[2495944]; Pediatr Infect Dis J(1989)[2594461]; Vrach Delo(1989)[2667256]; Zhonghua Nei Ke Za Zhi(1989)[2689107]; Chest(1989)[2651040]; Eur J Clin Microbiol Infect Dis(1989)[2495953]; Enferm Infecc Microbiol Clin(1989)[2490642]; CMAJ(1989)[2642395]; Int J Pediatr Otorhinolaryngol(1989)[2759784]; J Clin Lab Immunol(1989)[2534928]; Pathology(1989)[2812880]; Am Rev Respir Dis(1989)[2930067]; Medicina (B Aires)(1989)[2698435]; Pediatr Infect Dis J(1989)[2812912]; Nihon Kyobu Shikkan Gakkai Zasshi(1989)[2693782]; Ann Med Interne (Paris)(1989)[2662856]; Am J Med(1989)[2729338]; Cancer(1990)[2364370]; Semin Respir Infect(1990)[2188317]; Pediatr Pathol(1990)[2235766]; Nihon Kyobu Geka Gakkai Zasshi(1990)[2290059]; P N G Med J(1990)[2080675]; Nihon Kyobu Shikkan Gakkai Zasshi(1990)[2355700]; Kansenshogaku Zasshi(1990)[2338508]; Nihon Kyobu Shikkan Gakkai Zasshi(1990)[2355706]; Cas Lek Cesk(1990)[2369754]; Kansenshogaku Zasshi(1990)[2335751]; J Antimicrob Chemother(1990)[2312445]; Semin Respir Infect(1990)[2255806]; Rev Infect Dis(1990)[2237109]; Zhonghua Jie He He Hu Xi Za Zhi(1990)[2128219]; Pathol Biol (Paris)(1990)[2385449]; Kokyu To Junkan(1990)[2236963]; Jpn J Antibiot(1990)[2112207]; AJR Am J Roentgenol(1990)[2117371]; Kekkaku(1990)[2077264]; Pneumonol Pol(1990)[2216912]; J Assoc Physicians India(1990)[2380133]; Chest(1990)[2323247]; Pneumologie(1990)[2367437]; Am J Dis Child(1990)[2396617]; Rev Clin Esp(1990)[2247680]; Medicine (Baltimore)(1990)[2205784]; Kansenshogaku Zasshi(1990)[2243193]; Agressologie(1990)[2089979]; Respir Med(1990)[1699254]; Diabetes Care(1990)[2209323]; No Shinkei Geka(1990)[2359479]; Am J Pediatr Hematol Oncol(1990)[2240481]; Kansenshogaku Zasshi(1990)[2243194]; Nihon Hinyokika Gakkai Zasshi(1990)[2273697]; Med Klin (Munich)(1990)[2377146]; Rinsho Ketsueki(1991)[2027237]; Clin Ther(1991)[1799921]; Singapore Med J(1991)[2017709]; Rev Infect Dis(1991)[1925279]; Pediatr Emerg Care(1991)[1788120]; Thorax(1991)[1907034]; Int Orthop(1991)[1743834]; Monatsschr Kinderheilkd(1991)[2072965]; Enferm Infecc Microbiol Clin(1991)[1863618]; Eur Respir J(1991)[1889504]; Bull Soc Pathol Exot(1991)[1666982]; Clin Ther(1991)[1709390]; Am Rev Respir Dis(1991)[1859056]; Chemotherapy(1991)[1884650]; Jpn J Antibiot(1991)[1652653]; Orv Hetil(1991)[1861853]; Indian Pediatr(1991)[1748514]; Kansenshogaku Zasshi(1991)[1783810]; Kyobu Geka(1991)[1921004]; Pediatr Infect Dis J(1991)[2041665]; Chest(1991)[1864118]; Ann Pediatr (Paris)(1991)[1746849]; Pediatrics(1991)[1984618]; AJR Am J Roentgenol(1991)[2028898]; Kansenshogaku Zasshi(1991)[1761893]; Clin Ther(1991)[1790546]; J Antimicrob Chemother(1991)[1761451]; Kans Med(1991)[2002618]; Acta Paediatr Jpn(1992)[1621520]; Nihon Kyobu Shikkan Gakkai Zasshi(1992)[1405100]; Am J Med Sci(1992)[1539610]; Intern Med(1992)[1504436]; Nihon Naika Gakkai Zasshi(1992)[1453072]; Eur Respir J(1992)[1486973]; West J Med(1992)[1475945]; Respir Med(1992)[1565823]; Nihon Kyobu Geka Gakkai Zasshi(1992)[1593172]; Nihon Kyobu Shikkan Gakkai Zasshi(1992)[1318432]; Chest(1992)[1643942]; East Afr Med J(1992)[1505392]; South Med J(1992)[1411720]; Nihon Rinsho(1992)[1507430]; East Afr Med J(1992)[1473506]; Ann Pediatr (Paris)(1992)[1456675]; East Afr Med J(1992)[1505400]; Cent Afr J Med(1992)[1394392]; Bol Med Hosp Infant Mex(1992)[1449625]; Am J Dis Child(1992)[1496945]; Schweiz Med Wochenschr(1992)[1738821]; Postgrad Med J(1992)[1287613]; Ann Thorac Surg(1992)[1570995]; Zhonghua Nei Ke Za Zhi(1992)[1303851]; Kansenshogaku Zasshi(1992)[1624830]; Nihon Rinsho(1992)[1507434]; J Hosp Infect(1992)[1362749]; Burns(1992)[1418512]; S Afr Med J(1992)[1448709]; Intensive Care Med(1992)[1386615]; Kyobu Geka(1992)[1602688]; N Y State J Med(1992)[1518586]; Br Med Bull(1992)[1281036]; Chest(1992)[1729070]; J Am Geriatr Soc(1992)[1401689]; Kansenshogaku Zasshi(1992)[1431388]; Arq Bras Cardiol(1992)[1340713]; Kansenshogaku Zasshi(1992)[1402095]; Pediatr Infect Dis J(1992)[1461693]; Ann Trop Paediatr(1992)[1280041]; J Burn Care Rehabil(1992)[1429817]; Zhonghua Yi Xue Za Zhi(1992)[1338524]; Am Rev Respir Dis(1992)[1731595]; Gan To Kagaku Ryoho(1992)[1371046]; Rev Latinoam Microbiol(1993)[8209107]; Enferm Infecc Microbiol Clin(1993)[8481442]; Kansenshogaku Zasshi(1993)[7691973]; Pneumonol Alergol Pol(1993)[8148761]; J Trop Pediatr(1993)[8492372]; Respir Med(1993)[8265842]; Semin Respir Infect(1993)[8372274]; Zhonghua Yi Xue Za Zhi (Taipei)(1993)[8402370]; Arch Dis Child(1993)[8257181]; Kansenshogaku Zasshi(1993)[8294769]; Intern Med(1993)[8358116]; Nihon Kyobu Shikkan Gakkai Zasshi(1993)[8255025]; Ryumachi(1993)[8316908]; Clin Infect Dis(1993)[8286635]; Clin Rheumatol(1993)[8258248]; Chest(1993)[8252964]; Pediatrics(1993)[8424026]; J Trauma(1993)[8355313]; Chest(1993)[8222797]; J Hosp Infect(1993)[7905892]; Ann Fr Anesth Reanim(1993)[8338270]; Med J Aust(1993)[8487685]; Thorax(1993)[8236076]; Rev Pneumol Clin(1993)[8296141]; Drug Investig(1993)[32287509]; Clin Infect Dis(1993)[8218696]; Anaesthesist(1993)[8250207]; Intensive Care Med(1993)[8408937]; AJR Am J Roentgenol(1993)[8352125]; Clin Invest Med(1993)[8467579]; West Afr J Med(1994)[8080838]; Am J Infect Control(1994)[7695112]; Am J Respir Crit Care Med(1994)[8004324]; Am J Respir Crit Care Med(1994)[7952612]; Kansenshogaku Zasshi(1994)[8151148]; Scand J Infect Dis Suppl(1994)[8047855]; Nihon Jinzo Gakkai Shi(1994)[8139151]; West Afr J Med(1994)[7841101]; Lung Cancer(1994)[7812701]; An Med Interna(1994)[7772688]; Ethiop Med J(1994)[8033877]; Med Clin North Am(1994)[8078378]; Chest(1994)[8131547]; Semin Respir Infect(1994)[7831542]; Semin Respir Infect(1994)[7831538]; Acta Paediatr(1994)[7981562]; South Med J(1994)[8153775]; Ryoikibetsu Shokogun Shirizu(1994)[8152085]; Kansenshogaku Zasshi(1994)[8051440]; Semin Respir Infect(1994)[7831539]; Am J Surg(1994)[8135314]; Postgrad Med J(1994)[8183783]; Pneumologie(1994)[8183862]; J Gen Intern Med(1994)[7853075]; Infection(1994)[7927832]; Clin Infect Dis(1994)[8086544]; Can J Infect Dis(1994)[22346488]; Eur Respir J(1994)[7511540]; Kansenshogaku Zasshi(1994)[7996025]; Arch Bronconeumol(1994)[8025784]; Nihon Kyobu Shikkan Gakkai Zasshi(1994)[7799554]; Intensive Care Med(1994)[8201105]; Nihon Rinsho(1994)[8126901]; Arch Intern Med(1994)[7979856]; J Chemother(1994)[7799056]; Arch Intern Med(1994)[8092913]; Dtsch Med Wochenschr(1994)[8070330]; Thorax(1994)[8153944]; Kansenshogaku Zasshi(1994)[7829904]; Intern Med(1994)[7949649]; Scand J Infect Dis(1995)[8685635]; Eur Respir J(1995)[8620965]; J Chemother(1995)[8568541]; Vestn Ross Akad Med Nauk(1995)[7580418]; J Obstet Gynecol Neonatal Nurs(1995)[7562139]; Pediatr Pulmonol(1995)[8570307]; Enferm Infecc Microbiol Clin(1995)[7654835]; Clin Infect Dis(1995)[7548560]; Neth J Med(1995)[7643944]; Clin Pediatr (Phila)(1995)[7720324]; Kansenshogaku Zasshi(1995)[8708405]; J Antimicrob Chemother(1995)[8543488]; East Afr Med J(1995)[8689973]; Jpn J Antibiot(1995)[7745805]; Kansenshogaku Zasshi(1995)[7751746]; Rev Rhum Engl Ed(1995)[7788320]; Arch Intern Med(1995)[7763121]; Neth J Med(1995)[8569936]; J Trop Pediatr(1995)[7636941]; J Chemother(1995)[8568539]; Kyobu Geka(1995)[7474582]; Malays J Pathol(1995)[8907000]; Kansenshogaku Zasshi(1995)[7561255]; Acta Otorhinolaryngol Belg(1995)[7484143]; Nihon Naibunpi Gakkai Zasshi(1995)[7750628]; Chest(1995)[7750325]; Kyobu Geka(1995)[7745871]; South Med J(1995)[7732454]; Crit Care Med(1995)[7600827]; Microb Drug Resist(1995)[9158751]; Zhonghua Min Guo Wei Sheng Wu Ji Mian Yi Xue Za Zhi(1995)[9774999]; J Clin Forensic Med(1995)[15335635]; Eur J Emerg Med(1995)[9422174]; Can J Infect Dis(1995)[22416210]; Thorax(1995)[8553294]; JAMA(1995)[7637145]; Can J Infect Dis(1995)[22550412]; Gan To Kagaku Ryoho(1995)[7661584]; Med J Aust(1995)[7565207]; Eur J Surg(1995)[7772634]; Chest(1995)[7656634]; Arch Intern Med(1995)[7618989]; Infect Dis Clin North Am(1995)[7769221]; Pneumonol Alergol Pol(1995)[8520552]; Intensive Care Med(1996)[8844235]; Clin Infect Dis(1996)[8783710]; Pediatr Infect Dis J(1996)[8970218]; Pneumologie(1996)[9019751]; Presse Med(1996)[8958873]; Chemotherapy(1996)[8861533]; Nihon Geka Gakkai Zasshi(1996)[8774812]; Semin Respir Infect(1996)[8776779]; Semin Respir Infect(1996)[8776781]; Pediatr Emerg Care(1996)[8858655]; Rev Mal Respir(1996)[8711237]; Intern Med(1996)[9030999]; Respir Med(1996)[8984527]; Eur J Clin Microbiol Infect Dis(1996)[8839645]; Harefuah(1996)[8794638]; Arch Bronconeumol(1996)[8948878]; Monaldi Arch Chest Dis(1996)[9009626]; Br J Dermatol(1996)[8736347]; Pediatr Pulmonol(1996)[8905884]; Nihon Ronen Igakkai Zasshi(1996)[8868121]; Clin Infect Dis(1996)[8842271]; Int J Antimicrob Agents(1996)[18611723]; Radiol Clin North Am(1996)[8539353]; Laeknabladid(1996)[20065391]; Clin Infect Dis(1996)[8824970]; Arch Pediatr(1996)[8762954]; Postgrad Med J(1996)[8944211]; Aust N Z J Surg(1996)[8634044]; Pediatrics(1996)[8784356]; AIDS(1996)[8970683]; Bacteriol Virusol Parazitol Epidemiol(1996)[8963117]; Enferm Infecc Microbiol Clin(1996)[8695681]; HNO(1996)[8852801]; Pathol Biol (Paris)(1996)[8758486]; Arch Pediatr(1996)[8952771]; Am J Respir Crit Care Med(1997)[9001306]; Infect Control Hosp Epidemiol(1997)[9247830]; Kansenshogaku Zasshi(1997)[9339624]; Curr Opin Pulm Med(1997)[9193863]; Med Clin (Barc)(1997)[9333692]; Acta Med Austriaca(1997)[9206930]; Ugeskr Laeger(1997)[9340883]; East Afr Med J(1997)[9185416]; Clin Infect Dis(1997)[9332511]; Nihon Kyobu Shikkan Gakkai Zasshi(1997)[9396255]; Ann Ital Med Int(1997)[9409949]; Ann Acad Med Singap(1997)[9494663]; Scand J Infect Dis(1997)[9181654]; Rev Med Chir Soc Med Nat Iasi(1997)[10756729]; Ann Acad Med Singap(1997)[9494674]; Radiol Med(1997)[9280934]; Kansenshogaku Zasshi(1997)[9248265]; Kansenshogaku Zasshi(1997)[9339633]; Radiol Med(1997)[9280932]; Respiration(1997)[9154675]; J Okla State Med Assoc(1997)[9299893]; Ann Dermatol Venereol(1997)[9740863]; Antibiot Khimioter(1997)[9412402]; Pneumonol Alergol Pol(1997)[9760790]; Mem Inst Oswaldo Cruz(1997)[9566230]; Pneumologie(1997)[9173416]; Head Neck(1997)[9243263]; Kansenshogaku Zasshi(1997)[9394563]; Can J Infect Dis(1997)[22346528]; N Z Med J(1997)[9315030]; Eur J Epidemiol(1997)[9324219]; Thorax(1997)[9039234]; Nihon Rinsho(1997)[9360392]; Infect Control Hosp Epidemiol(1997)[9397368]; Rev Clin Esp(1997)[9558601]; Infect Dis Clin North Am(1998)[9779381]; Antimicrob Agents Chemother(1998)[9559773]; Br J Theatre Nurs(1998)[9677888]; Crit Care Clin(1998)[9561819]; Drugs Today (Barc)(1998)[15094862]; N Engl J Med(1998)[9882209]; Clin Infect Dis(1998)[9524833]; Chest(1998)[9792588]; Nihon Naika Gakkai Zasshi(1998)[9549312]; Pediatr Infect Dis J(1998)[9535250]; Kansenshogaku Zasshi(1998)[9545687]; J Chemother(1998)[9531074]; J Thorac Imaging(1998)[9799134]; Emerg Infect Dis(1998)[9716961]; Eur J Clin Microbiol Infect Dis(1998)[9832263]; Medicine (Baltimore)(1998)[9465863]; J Hosp Infect(1998)[9868618]; Intensive Care Med(1998)[9885888]; Eur Respir J(1998)[9701424]; Pathol Biol (Paris)(1998)[9769865]; Am J Respir Crit Care Med(1998)[9563735]; Arch Intern Med(1998)[9818796]; Clin Exp Obstet Gynecol(1998)[9987566]; Dakar Med(1998)[10797962]; Clin Infect Dis(1998)[9770140]; Pediatr Pulmonol(1998)[9888211]; QJM(1998)[9519211]; J Heart Lung Transplant(1998)[9588587]; Hinyokika Kiyo(1998)[9546132]; Scand Cardiovasc J(1998)[9802144]; Arch Bronconeumol(1998)[9803276]; Arch Pediatr(1998)[10223157]; Kansenshogaku Zasshi(1998)[9796188]; Med Clin (Barc)(1998)[9717164]; Minerva Anestesiol(1998)[9951272]; Am J Cardiol(1998)[9761092]; Eur J Clin Microbiol Infect Dis(1998)[9758274]; Am J Med(1998)[9684654]; Eur J Surg(1998)[9696441]; Infect Control Hosp Epidemiol(1998)[9475347]; J Clin Microbiol(1998)[9466751]; Int J STD AIDS(1998)[9874118]; J Cardiovasc Surg (Torino)(1998)[9537546]; Ann Thorac Surg(1998)[9456102]; Intensive Care Med(1998)[9840237]; Pneumoftiziologia(1998)[10386145]; Presse Med(1998)[9819592]; J Hosp Infect(1998)[9602976]; Am J Respir Crit Care Med(1998)[9476869]; Chest(1998)[9596298]; Am J Respir Crit Care Med(1998)[9731019]; Presse Med(1998)[9793046]; Chest(1998)[9580099]; Zhonghua Yi Xue Za Zhi (Taipei)(1998)[9830236]; Pneumologie(1998)[9885511]; Zh Mikrobiol Epidemiol Immunobiol(1998)[9783407]; Med Clin (Barc)(1998)[9922954]; Intensive Care Med(1998)[9539080]; Diagn Microbiol Infect Dis(1998)[9934546]; Clin Chest Med(1999)[10516899]; Emerg Infect Dis(1999)[10341185]; Semin Respir Infect(1999)[10638512]; Jpn J Thorac Cardiovasc Surg(1999)[10496060]; J Trop Pediatr(1999)[10467836]; Jpn J Clin Oncol(1999)[10073152]; QJM(1999)[10627862]; J Med Assoc Thai(1999)[10730534]; Clin Infect Dis(1999)[10589891]; Semin Respir Infect(1999)[10197394]; Chest(1999)[10084458]; Sante(1999)[10477402]; Am Surg(1999)[10432077]; Compr Ther(1999)[9987589]; Clin Infect Dis(1999)[10524959]; Aust N Z J Med(1999)[10200815]; J Microbiol Immunol Infect(1999)[10650491]; Kansenshogaku Zasshi(1999)[10535263]; Am J Perinatol(1999)[10614699]; Diagn Microbiol Infect Dis(1999)[10091036]; J Pediatr (Rio J)(1999)[14685562]; Enferm Infecc Microbiol Clin(1999)[10396086]; Int J Pediatr Otorhinolaryngol(1999)[10577771]; Antibiot Khimioter(1999)[10095919]; Intensive Care Med(1999)[10551961]; Indian J Pediatr(1999)[10798147]; Acta Paediatr Taiwan(1999)[10910624]; Am J Respir Crit Care Med(1999)[10430736]; Crit Care Med(1999)[10321654]; Rev Clin Esp(1999)[10216394]; Chest(1999)[10084452]; J Infect Dis(1999)[10558935]; Kansenshogaku Zasshi(1999)[10423947]; Eur J Cardiothorac Surg(1999)[10371127]; Infez Med(1999)[12748443]; Presse Med(1999)[10506875]; RN(1999)[10205564]; Semin Thromb Hemost(1999)[10357089]; Medicina (B Aires)(1999)[10752217]; Crit Care Med(1999)[10470744]; Singapore Med J(1999)[10572489]; Schweiz Med Wochenschr(1999)[10568234]; Singapore Med J(1999)[10487085]; Crit Care Med(1999)[10362409]; Pneumonol Alergol Pol(1999)[10481526]; Pediatr Infect Dis J(1999)[10530574]; Chest(1999)[9925081]; Ryoikibetsu Shokogun Shirizu(1999)[10088411]; Transplantation(1999)[10232564]; Postgrad Med(1999)[10223091]; Acta Radiol(1999)[10080731]; Eur J Clin Microbiol Infect Dis(1999)[10691195]; Pathol Biol (Paris)(1999)[10418017]; Chest(1999)[10084487]; Braz J Infect Dis(1999)[11097714]; Am J Respir Crit Care Med(2000)[10988092]; Leg Med (Tokyo)(2000)[12935447]; Am J Perinatol(2000)[11142393]; Am Surg(2000)[11149581]; Diagn Microbiol Infect Dis(2000)[10863106]; Tidsskr Nor Laegeforen(2000)[10851938]; Nihon Kokyuki Gakkai Zasshi(2000)[10774175]; Ugeskr Laeger(2000)[10860428]; Eur J Pediatr(2000)[11014470]; Am J Surg(2000)[10802251]; Am J Surg(2000)[10874100]; Am J Surg(2000)[10802257]; Tohoku J Exp Med(2000)[11211316]; Eur J Clin Microbiol Infect Dis(2000)[11205629]; Med Oncol(2000)[10871816]; Clin Infect Dis(2000)[11073748]; Semin Respir Crit Care Med(2000)[16088716]; Semin Respir Crit Care Med(2000)[16088717]; Sante(2000)[10960805]; Ned Tijdschr Geneeskd(2000)[11048555]; Clin Infect Dis(2000)[11073777]; Curr Opin Pulm Med(2000)[11100967]; Kansenshogaku Zasshi(2000)[10835838]; Eur Respir J(2000)[11292126]; Clin Infect Dis(2000)[10913417]; Chemotherapy(2000)[10765030]; Zhonghua Jie He He Hu Xi Za Zhi(2000)[11778507]; Pediatrics(2000)[11099630]; Am J Surg(2000)[10802259]; Am J Respir Crit Care Med(2000)[10712318]; Diagn Microbiol Infect Dis(2000)[10794943]; Rev Med Chil(2000)[11008357]; Am J Health Syst Pharm(2000)[11057360]; Am J Manag Care(2000)[10977480]; Nihon Rinsho(2000)[11225327]; Am J Surg(2000)[10874106]; Int J Antimicrob Agents(2000)[11137406]; Przegl Epidemiol(2000)[11349588]; East Afr Med J(2000)[12862109]; Eur J Pediatr(2000)[11014469]; Pediatr Int(2000)[10881583]; Chest(2000)[10767233]; J Crit Care(2000)[11011820]; Can J Infect Dis(2000)[18159274]; Transplantation(2000)[10868641]; Crit Care Med(2000)[11057812]; Crit Care Med(2000)[10966244]; N Z Med J(2000)[10909937]; Chest(2000)[11115444]; N Z Med J(2000)[10894342]; Minerva Med(2000)[11084845]; Cochrane Database Syst Rev(2000)[10796836]; Infect Control Hosp Epidemiol(2000)[10782588]; Ann Transplant(2000)[11147024]; Curr Infect Dis Rep(2000)[11095858]; Arch Bronconeumol(2000)[11004983]; J Formos Med Assoc(2000)[11155749]; Chest(2000)[11115467]; J Trop Pediatr(2000)[11191152]; Chest(2000)[10807834]; AJR Am J Roentgenol(2000)[10882278]; J Assoc Physicians India(2000)[11310380]; Infect Control Hosp Epidemiol(2000)[10968716]; J Chemother(2001)[11936369]; Antibiot Khimioter(2001)[11573325]; Arch Pediatr(2001)[11582921]; J Dermatol(2001)[11732724]; Rev Esp Quimioter(2001)[11856984]; Med J Malaysia(2001)[11732071]; Pediatr Crit Care Med(2001)[12797868]; J Chemother(2001)[11589485]; J Hosp Infect(2001)[11289777]; East Mediterr Health J(2001)[15332774]; Nihon Kokyuki Gakkai Zasshi(2001)[11729685]; Chest(2001)[11171773]; Ther Umsch(2001)[11695094]; Clin Infect Dis(2001)[11320452]; Semin Respir Crit Care Med(2001)[16088683]; Diagn Microbiol Infect Dis(2001)[11687309]; Rev Pneumol Clin(2001)[11353919]; Arch Intern Med(2001)[11525709]; Fortschr Med Orig(2001)[11935653]; J Am Geriatr Soc(2001)[11380747]; Altern Ther Health Med(2001)[11347281]; Drugs Aging(2001)[11302286]; Unfallchirurg(2001)[11381766]; Ther Umsch(2001)[11695092]; Indian J Pediatr(2001)[11770243]; Heart(2001)[11711482]; Burns(2001)[11451607]; Acta Diabetol(2001)[11757805]; Am J Respir Crit Care Med(2001)[11254518]; Intern Med J(2001)[11480485]; Can Assoc Radiol J(2001)[11780550]; Eur J Clin Microbiol Infect Dis(2001)[11837636]; J Med Liban(2001)[12243418]; Kansenshogaku Zasshi(2001)[11357318]; Int J Antimicrob Agents(2001)[11165120]; Pol Merkur Lekarski(2001)[11770318]; Am J Epidemiol(2001)[11207152]; Crit Care Med(2001)[11292880]; Postgrad Med(2001)[19667559]; Am J Med(2001)[11755439]; Clin Exp Dermatol(2001)[11722449]; Arch Pediatr(2001)[11582915]; Am J Infect Control(2001)[11172313]; East Afr Med J(2001)[11921576]; Cochrane Database Syst Rev(2001)[11687002]; Crit Care Med(2001)[11246310]; Chest(2001)[11399696]; Indian J Chest Dis Allied Sci(2001)[11529434]; Acta Paediatr(2001)[11236037]; Am J Med(2001)[11755441]; Crit Care Med(2001)[11801813]; Semin Respir Infect(2001)[11562899]; Indian J Pediatr(2001)[11563247]; CMAJ(2001)[11468949]; Crit Care(2001)[11353934]; J Pediatr Surg(2001)[11227003]; J Neurol(2001)[11757959]; Thorax(2001)[11312407]; Drug Ther Bull(2001)[11471517]; Swiss Med Wkly(2001)[11875753]; Diagn Microbiol Infect Dis(2001)[11248523]; Crit Care Med(2001)[11373418]; Braz J Infect Dis(2001)[11506775]; Pediatr Infect Dis J(2001)[11368105]; Arch Dis Child(2001)[11159295]; Minerva Anestesiol(2001)[11731755]; Rev Cubana Med Trop(2002)[15849942]; J Hosp Infect(2002)[12392904]; Semin Respir Infect(2002)[11891518]; J Chemother(2002)[12583547]; Ann Fr Anesth Reanim(2002)[12078429]; Rinsho Byori(2002)[12187704]; J Dermatol(2002)[11837574]; J Fam Pract(2002)[12184969]; Arch Dis Child(2002)[12244005]; Emerg Infect Dis(2002)[11749750]; Infect Control Hosp Epidemiol(2002)[11918125]; Jpn J Thorac Cardiovasc Surg(2002)[12166265]; Diabetes Metab(2002)[12442071]; Pediatr Infect Dis J(2002)[12182396]; J Microbiol Immunol Infect(2002)[12099332]; Am J Respir Crit Care Med(2002)[12379545]; Paediatr Drugs(2002)[11888355]; Southeast Asian J Trop Med Public Health(2002)[12118460]; Geriatrics(2002)[11899549]; Semin Respir Infect(2002)[11891522]; Lancet(2002)[11888586]; Clin Infect Dis(2002)[12228818]; N Engl J Med(2002)[12181400]; Pediatr Pulmonol(2002)[11836802]; Eur Radiol(2002)[11870440]; Am J Respir Crit Care Med(2002)[11934711]; Nihon Geka Gakkai Zasshi(2002)[12599923]; J Cardiovasc Surg (Torino)(2002)[12483175]; J Am Geriatr Soc(2002)[12121517]; J Infect Chemother(2002)[11957128]; Scand J Infect Dis(2002)[12030399]; Postgrad Med J(2002)[12151652]; Unfallchirurg(2002)[11995227]; Acta Paediatr Taiwan(2002)[12632784]; Otol Neurotol(2002)[12170160]; Scand J Infect Dis(2002)[12477329]; Southeast Asian J Trop Med Public Health(2002)[12757224]; Pediatr Infect Dis J(2002)[12182377]; Int J Clin Pract Suppl(2002)[12014853]; Diagn Microbiol Infect Dis(2002)[12493178]; J Pediatr(2002)[12006957]; Int J Infect Dis(2002)[12044296]; Rev Med Chil(2002)[12611238]; Arch Pediatr(2002)[12108316]; J Antimicrob Chemother(2002)[12205063]; Int J Infect Dis(2002)[12718823]; East Afr Med J(2002)[12643233]; Kansenshogaku Zasshi(2002)[11974888]; Recenti Prog Med(2002)[12355983]; Gerodontology(2002)[12542215]; Tidsskr Nor Laegeforen(2002)[12448252]; Crit Care Med(2002)[12163786]; Inflamm Res(2002)[12056515]; West Indian Med J(2002)[12089869]; Southeast Asian J Trop Med Public Health(2002)[12236437]; Clin Ther(2002)[12117077]; Chest(2002)[12171848]; J Pediatr Surg(2002)[12378443]; Infection(2003)[14556063]; Rev Prat(2003)[14558265]; Anaesthesist(2003)[14504808]; Am J Respir Med(2003)[14720021]; Presse Med(2003)[12754444]; Arch Dis Child(2003)[14612371]; Clin Infect Dis(2003)[14523769]; Pediatr Infect Dis J(2003)[12613460]; Braz J Infect Dis(2003)[12807691]; Kyobu Geka(2003)[12607257]; Intensive Care Med(2003)[12677369]; Am J Respir Crit Care Med(2003)[12689848]; Kyobu Geka(2003)[12854472]; Diagn Microbiol Infect Dis(2003)[12730000]; Ren Fail(2003)[12803514]; Rev Mal Respir(2003)[12910140]; Arch Pediatr(2003)[12907071]; J Trop Pediatr(2003)[12729288]; Wien Klin Wochenschr(2003)[12674685]; Nurs Stand(2003)[14649189]; Am J Infect Control(2003)[12762292]; Yonsei Med J(2003)[12619189]; Semin Respir Crit Care Med(2003)[16088523]; Rev Mal Respir(2003)[12910141]; Infect Control Hosp Epidemiol(2003)[14510249]; Przegl Lek(2003)[14575024]; Int J Med Microbiol(2003)[14503795]; Braz J Infect Dis(2003)[14499046]; Acta Microbiol Pol(2003)[14594401]; Saudi Med J(2003)[14710292]; Medicina (Kaunas)(2003)[12695638]; Am J Respir Crit Care Med(2003)[12433668]; Infect Control Hosp Epidemiol(2003)[14649777]; Cochrane Database Syst Rev(2003)[12917916]; Metabolism(2003)[14577062]; Eur Respir J(2003)[14680072]; Chest(2003)[14605050]; Int J Infect Dis(2003)[12839710]; Asian Cardiovasc Thorac Ann(2003)[12692031]; Diagn Microbiol Infect Dis(2003)[12729995]; Semin Respir Crit Care Med(2003)[16088583]; Intensive Care Med(2003)[12904849]; Pneumonol Alergol Pol(2003)[12959021]; Curr Opin Infect Dis(2003)[12821819]; Arch Bronconeumol(2003)[12890400]; Chest(2003)[12853512]; Arch Pediatr(2003)[12907073]; J Pediatr Hematol Oncol(2003)[12544773]; Arch Bronconeumol(2003)[12975070]; Sao Paulo Med J(2003)[12920475]; Ned Tijdschr Geneeskd(2003)[12814021]; J Hosp Infect(2003)[12495679]; Antimicrob Agents Chemother(2003)[12878545]; Harefuah(2003)[12754875]; Med Princ Pract(2003)[12634471]; Intensive Care Med(2003)[12898002]; Surg Infect (Larchmt)(2003)[12744764]; Pediatr Pulmonol(2003)[12772225]; Intern Med(2003)[12583614]; Hunan Yi Ke Da Xue Xue Bao(2003)[14653088]; Zhonghua Er Ke Za Zhi(2003)[14746685]; Crit Care Med(2003)[14530765]; Turk J Pediatr(2003)[14696807]; Nihon Rinsho(2003)[14619435]; Rev Hosp Clin Fac Med Sao Paulo(2003)[14666322]; Pediatr Infect Dis J(2003)[12913766]; Postgrad Med(2003)[12647477]; Chest(2003)[12628886]; J Microbiol Immunol Infect(2004)[15060682]; Clin Genet(2004)[15151509]; Curr Opin Pulm Med(2004)[15514493]; J Trop Pediatr(2004)[15357563]; Clin Infect Dis(2004)[15356814]; Di Yi Jun Yi Da Xue Xue Bao(2004)[15604082]; Kyobu Geka(2004)[15366570]; Recenti Prog Med(2004)[15032335]; Anesthesiol Clin North Am(2004)[15325711]; Eur Respir J(2004)[15358692]; J Int Med Res(2004)[14997711]; Scand J Infect Dis(2004)[15061671]; Antibiot Khimioter(2004)[15344392]; J Microbiol Immunol Infect(2004)[15060683]; Clin Infect Dis(2004)[15095210]; Chest(2004)[15249482]; Kansenshogaku Zasshi(2004)[15628531]; Clin Lab Med(2004)[15177847]; Pediatr Pulmonol(2004)[15334503]; Clin Microbiol Infect(2004)[15191383]; Neurosurg Focus(2004)[15636567]; J Cyst Fibros(2004)[15463902]; In Vivo(2004)[15011754]; Assist Inferm Ric(2004)[15152377]; Eur J Clin Microbiol Infect Dis(2004)[15558346]; Pathol Biol (Paris)(2004)[15465267]; Clin Microbiol Rev(2004)[14726455]; Acta Paediatr(2004)[15244228]; Scand J Infect Dis(2004)[15198183]; Am J Respir Crit Care Med(2004)[15242840]; Interact Cardiovasc Thorac Surg(2004)[17670247]; Med Mal Infect(2004)[15612359]; Orv Hetil(2004)[15384746]; Korean J Intern Med(2004)[15053046]; Med J Aust(2004)[15310264]; Zhonghua Lao Dong Wei Sheng Zhi Ye Bing Za Zhi(2004)[15033031]; BMC Pulm Med(2004)[15109397]; Indian J Chest Dis Allied Sci(2004)[14870864]; Infect Control Hosp Epidemiol(2004)[15484803]; Crit Care Med(2004)[15071391]; P R Health Sci J(2004)[15125215]; J Infect Chemother(2004)[15614461]; Southeast Asian J Trop Med Public Health(2004)[15691151]; Kyobu Geka(2004)[15202275]; Can J Infect Dis(2004)[18159439]; J Paediatr Child Health(2004)[15265191]; Eur J Cardiothorac Surg(2004)[15145012]; Acta Paediatr(2004)[15918230]; Kyobu Geka(2004)[15553037]; Zhonghua Jie He He Hu Xi Za Zhi(2004)[15130324]; Int J Pediatr Otorhinolaryngol(2004)[15126016]; Resuscitation(2004)[14987786]; Anasthesiol Intensivmed Notfallmed Schmerzther(2004)[14740311]; Acta Paediatr Taiwan(2004)[15868807]; Curr Opin Pulm Med(2004)[15071367]; Probl Tuberk Bolezn Legk(2004)[15315123]; J Trauma(2004)[14960971]; J Ayub Med Coll Abbottabad(2004)[15762062]; Infect Control Hosp Epidemiol(2004)[15484800]; Chest(2004)[15249447]; Medicina (B Aires)(2004)[15338978]; Otolaryngol Head Neck Surg(2004)[15577783]; Zhongguo Wei Zhong Bing Ji Jiu Yi Xue(2004)[15355618]; Intensive Care Med(2004)[14714 |
[truncated: 174,426 more chars]
